# Monoallelic *PSMB8* variants cause PRAAS with immunodeficiency through impaired immunoproteasome assembly

**DOI:** 10.1016/j.ajhg.2026.04.015

**Published:** 2026-05-21

**Authors:** Robin Wijngaard, Caspar I. van der Made, Sema Kalkan Uçar, Gayatri Ramakrishnan, Man Wang, Johannes Brand, Jill A. Rosenfeld, Tiphanie P. Vogel, Sarah K. Nicholas, Monika Weisz-Hubshman, Undiagnosed Diseases Network, Clara D.M. van Karnebeek, Eric J. Allenspach, Taylor E. Gardiner, Sumudu Perera Kimmantudawage, Zornitza Stark, Ruth K. Armstrong, Janine Campbell, Stefano Volpi, Enrico Drago, Marco Gattorno, Alice Grossi, Isabella Ceccherini, Alfredo Cabrera-Orefice, Bente Siebels, Thomas Mair, Hartmut Schlüter, Ruben L. Smeets, Ronald van Beek, Ingrid Goebel, Katrin Küchler, Søren W. Gersting, Alexander Hoischen, Lisenka E.L.M. Vissers, Ron A. Wevers, Catherine Meyer-Schwesinger, Saskia B. Wortmann, Machteld M. Oud, Sergio Guerrero-Castillo

**Affiliations:** 1 Department of Human Genetics, Radboud University Medical Center, Nijmegen, the Netherlands; 2 Department of Internal Medicine and Radboud Center for Infectious Diseases (RCI), Radboud University Medical Center, Nijmegen, the Netherlands; 3 Division of Pediatric Nutrition and Metabolism, Department of Pediatrics, Ege University Faculty of Medicine, İzmir, Turkey; 4 Bangalore, India; 5 Institute of Cellular and Integrative Physiology, University Medical Center Hamburg-Eppendorf, Hamburg, Germany; 6 Department of Molecular and Human Genetics, Baylor College of Medicine, Houston, TX, USA; 7 Division of Rheumatology, Department of Pediatrics, Baylor College of Medicine and Center for Human Immunobiology, Texas Children’s Hospital, Houston, TX, USA; 8 Division of Immunology, Allergy, and Retrovirology, Department of Pediatrics, Baylor College of Medicine, Houston, TX, USA; 9 Genetics Department, Texas Children’s Hospital, Houston, TX, USA; 10 Departments of Pediatrics and Human Genetics, Emma Center for Personalized Medicine, Amsterdam University Medical Centers, Amsterdam, the Netherlands; 11 Department of Pediatrics, Divisions of Immunology and Pediatric Rheumatology, University of Washington, Seattle, WA, USA; 12 Seattle Children’s Hospital, Division of Immunology, Seattle, WA, USA; 13 Victorian Clinical Genetics Services, Murdoch Children’s Research Institute, Melbourne, VIC, Australia; 14 Department of Paediatrics, University of Melbourne, Melbourne, VIC, Australia; 15 Department of Neonatal Medicine, The Royal Children’s Hospital, Melbourne, VIC, Australia; 16 Department of Clinical Haematology, The Royal Children’s Hospital, Melbourne, VIC, Australia; 17 UOC Clinical and Experimental Immunology, IRCCS Istituto Giannina Gaslini, Genoa, Italy; 18 Department of Neuroscience, Rehabilitation, Ophthalmology, Genetics, Maternal and Child Health (DINOGMI), Università Degli Studi di Genova, Genova, Italy; 19 Gene Therapy Program, Dana Farber/Boston Children’s Cancer and Blood Disorders Center, Harvard Medical School, Boston, MA, USA; 20 UOSD Area Aggregazione Laboratori Della Ricerca, IRCCS Istituto Giannina Gaslini, Genoa, Italy; 21 Research Institute for Medical Innovation, Radboud University Medical Center, Nijmegen, the Netherlands; 22 Section Mass Spectrometry and Proteomics, University Medical Center Hamburg-Eppendorf, Hamburg, Germany; 23 Department of Laboratory Medicine, Laboratory of Medical Immunology, Radboud University Medical Center, Nijmegen, the Netherlands; 24 Department of Laboratory Medicine, Radboudumc Laboratory for Diagnostics, Radboud University Medical Center, Nijmegen, the Netherlands; 25 University Children’s Research@Kinder-UKE, University Medical Center Hamburg-Eppendorf, Hamburg, Germany; 26 German Center for Child and Adolescent Health (DZKJ), Partner Site Hamburg, Hamburg, Germany; 27 Translational Metabolic Laboratory, Department of Human Genetics, Radboud University Medical Center, Nijmegen, the Netherlands; 28 University Children’s Hospital, Paracelsus Medical University, Salzburg, Austria

**Keywords:** immunoproteasome, immunodeficiency, proteasome-associated autoinflammatory syndrome, complexome profiling, monoallelic *PSMB8* variants, proteasome biogenesis, proteasome dysfunction, PRAAS, PRAAS-ID, dominant-negative

## Abstract

Monoallelic variants in catalytic immunoproteasome subunits have recently been linked to proteasome-associated autoinflammatory syndromes with immunodeficiency (PRAAS-ID), yet their molecular mechanisms and clinical spectra are not fully defined. In this study, seven individuals from five unrelated families carrying five distinct monoallelic *PSMB8* variants were identified. Individuals presented with neonatal-onset immunodeficiency characterized by recurrent infections, B cell lymphopenia, and hypogammaglobulinemia requiring immunoglobulin replacement. Inflammatory manifestations of variable severity included enteropathy, hepatitis, myositis, and inflammatory lung disease. Additional findings included leukocyte vacuolization in blood and bone marrow. Pathogenic variants in immunoproteasome subunits were analyzed to identify structural features associated with dominant-negative behavior. Immunoproteasome assembly and activity were investigated using complexome profiling, immunoblotting, and in-gel activity assays in proband-derived fibroblasts and transfected HEK293T cells**,** with downstream effects assessed by proteomic and RT-qPCR analyses. Mutant PSMB8 subunits were inefficiently incorporated into immunoproteasome complexes, leading to impaired assembly, including reduced fully assembled complexes and accumulation of assembly intermediates. This defect was accompanied by activation of the integrated stress response alongside impaired immune signaling. Monoallelic pathogenic variants in *PSMB8*, *PSMB9*, and *PSMB10* associated with PRAAS-ID affected residues that are highly conserved and biophysically similar between the three immunoproteasome catalytic subunits. These shared structural features may help identify additional variants with similar disruptive effects on immunoproteasome assembly. Together, our data show that monoallelic *PSMB8* variants disrupt immunoproteasome assembly, resulting in clinically variable disease with immunodeficiency and systemic inflammation. Our findings support immunoproteasome assembly disruption as a unifying dominant-negative mechanism underlying PRAAS-ID.

## Graphical abstract

**Figure:**
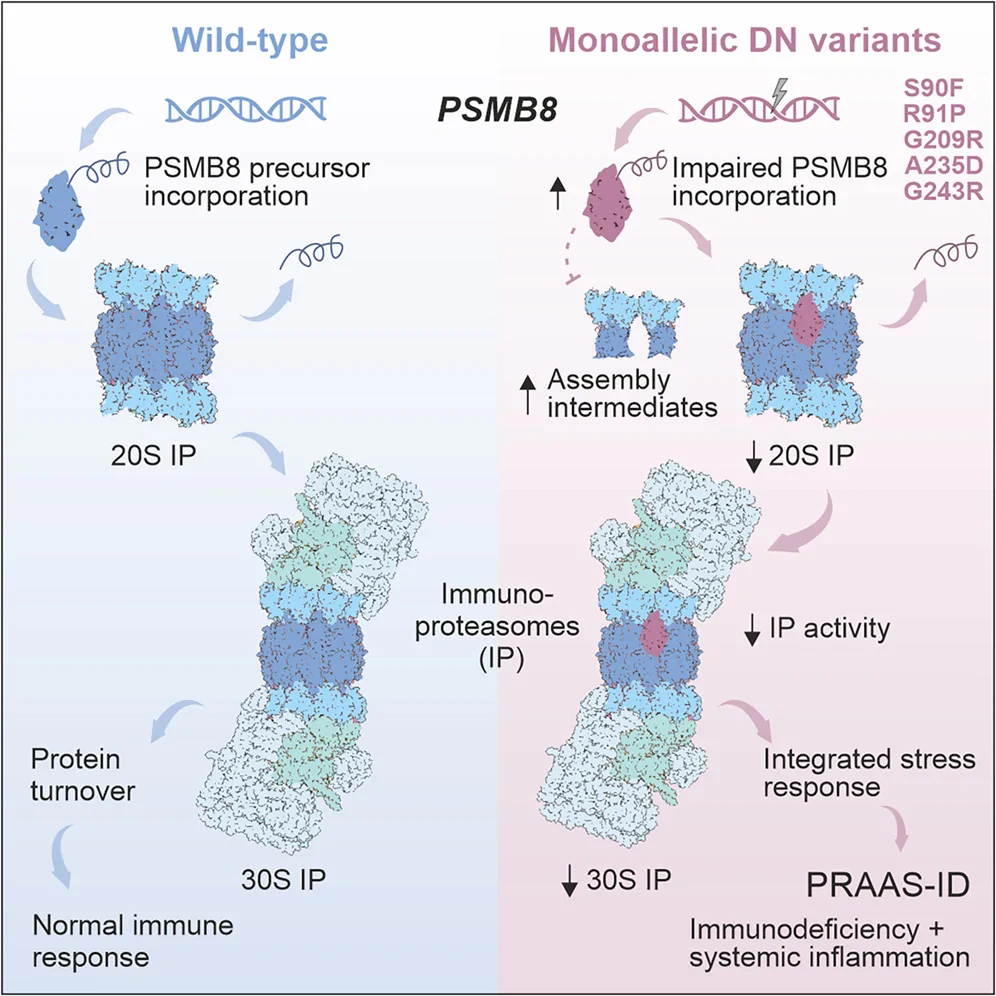

## Teaser

Seven individuals from five families harboring monoallelic *PSMB8* variants presented with immunodeficiency and systemic inflammation. Mutant PSMB8 proteins are inefficiently incorporated into immunoproteasome complexes, impairing assembly and triggering cellular stress responses. This dominant-negative mechanism unifies PRAAS-IDs and highlights structural vulnerabilities in catalytic subunits.

## Introduction

Proteasomes are large multiprotein complexes that mediate protein degradation within cells, primarily targeting ubiquitinated proteins but also processing non-ubiquitinated substrates.^1^ The proteasome is composed of a catalytic 20S core particle combined with one or two regulatory subcomplexes that modulate its activity, including 19S or PA700, which, together with the 20S, form the 26S or 30S proteasome, the 11S or PA28, and PA200.^1^^,^^2^^,^^3^ The 20S core particle is a cylindrical structure composed of four stacked rings: two outer rings of seven α subunits and two inner rings of seven β subunits.^4^ In the constitutively expressed standard proteasome (SP), the β rings contain three catalytic subunits, PSMB6 (β1), PSMB7 (β2), and PSMB5 (β5), with caspase-, trypsin-, and chymotrypsin-like activity, respectively.^5^ These subunits are synthesized as precursors and activated through N-terminal propeptide cleavage. In addition, tissue-specific forms exist, including the immunoproteasome (IP), the thymoproteasome, and the spermatoproteasome. In the IP, expressed in immune cells or in other cell types in response to pro-inflammatory cytokines such as interferon-γ (IFNγ), the SP catalytic subunits are replaced by PSMB9 (β1i), PSMB10 (β2i), and PSMB8 (β5i).^6^^,^^7^^,^^8^ These specialized subunits provide the IP with peptide-cleavage properties optimized for antigen presentation via major histocompatibility complex (MHC) class I, supporting adaptive immunity.^9^^,^^10^^,^^11^

Assembly of the 20S proteasome core is a highly ordered and regulated process.^4^^,^^12^^,^^13^^,^^14^ Formation begins with an α ring scaffold, followed by stepwise incorporation of β subunits to generate a half-mer or 13S intermediate, which subsequently dimerizes to form the mature 20S particle.^3^^,^^13^^,^^15^ The assembly process is tightly regulated by assembly chaperones, such as PSMG1-4 or POMP, which disassociate as assembly progresses and are absent in the mature complexes.^13^ Notably, the incorporation order of certain β subunits differs between the IP and the SP; for example, the inducible subunit PSMB9 can enter precursor complexes earlier than its constitutive homolog PSMB6.^3^^,^^8^^,^^15^^,^^16^ These distinct incorporation kinetics promote preferential formation of homogeneous SP or IP particles, although mixed proteasomes containing combinations of inducible and constitutive catalytic subunits have also been demonstrated.^12^^,^^17^^,^^18^

Dysfunction of the IP gives rise to a group of inborn errors of immunity known as proteasome-associated autoinflammatory syndromes (PRAAS). PRAAS are characterized by sustained pathological expression of type I IFN cytokines and multisystemic hyperinflammation, including rash, fevers, and organ dysfunction.^19^^,^^20^ Classical PRAAS follows autosomal-recessive or oligogenic inheritance involving two or more variants in IP catalytic subunits, assembly chaperones, or shared subunits between the SP and the IP (MIM: 256040, 617591, 619183, and 619175).^21^^,^^22^^,^^23^^,^^24^^,^^25^^,^^26^ However, autosomal-dominant inheritance has been described for some subunits (MIM: 618048, 620796*,* and 620807). These disorders, often referred to as PRAAS with immunodeficiency (PRAAS-ID), are thought to act via dominant-negative mechanisms that disrupt proteasome assembly.^27^^,^^28^^,^^29^^,^^30^

Here, we show that multiple monoallelic *PSMB8* (MIM: 177046) variants impair IP assembly, leading to clinically variable immunodeficiency and inflammatory disease, with leukocyte vacuolization and inclusions observed in some individuals. The variants share structural, biophysical, and functional consequences with previously described monoallelic variants in *PSMB9* (MIM: 177045) and *PSMB10* (MIM: 176847), providing a unifying molecular explanation for PRAAS-ID and expanding its clinical and genetic spectrum.

## Material and methods

### Inclusion and ethical considerations

Seven individuals from five families were identified through research projects at their respective centers and connected via GeneMatcher.^31^ All studies were approved by the relevant local institutional review boards or ethics committees, and written informed consent was obtained from participants or their caregivers, including consent for publication of clinical images if appropriate. Detailed information on recruitment and ethics approvals is provided in the supplemental material and methods.

### Data collection and genetic analysis

Clinical and laboratory data were obtained during routine diagnostic evaluations at each center. Sequencing was performed locally using standard exome or genome sequencing protocols on DNA extracted from blood, buccal swabs, or frozen brain tissue. Additional family members were sequenced to determine the variant’s mode of inheritance. Family-specific details are provided in the supplemental material and methods.

### Structural biology analysis

We modeled the *PSMB8* variants in the crystal structures of the human 20S IP containing PSMB8 (PDB: 6E5B) and the human 26S SP using the paralogous PSMB5 subunit (PDB: 6MSB, 60.3% sequence identity), to model the PSMB8 variants.^32^^,^^33^ Structural models were generated using RepairPDB and BuildModel functions, with five iterations of side-chain rotamer adjustments. Changes in protein stability were estimated using the FoldX energy function (v.5.0), with average free energy differences between wild-type (WT) and variant structures in kcal/mol.^34^ Frustration index (FI) analysis was computed to account for shifts in energetic distributions in the variant environment using Frustratometer2.^35^ Mutational and configurational FIs and residue contact interactions were calculated for residues within 5 Å centered at Cα of the variant site. A contact was considered minimally frustrated if scores were >0.78, highly frustrated if they were <−1.00, and neutral in between.

To investigate whether paralogous positions across proteasome subunits show similar structural and evolutionary properties, 21 described pathogenic missense variants affecting the mature regions of PSMB8, PSMB9, and PSMB10 were manually curated from the literature and ClinVar (including those identified in this study) (Table S1). Variants were classified according to disease association as classical PRAAS for variants reported in the context of recessive disease and PRAAS-ID for variants reported in the context of dominant disease. The variants were modeled across all 17 paralogous proteasome subunits of the 20S core particle (PSMA1–7 and PSMB1–10) by introducing equivalent amino acid substitutions at aligned positions. Evolutionary conservation was assessed using Rate4Site scores derived from multiple sequence alignments built from protein sequences with ≥70% identity retrieved from UniProt.^36^ The distribution of variant locations within the protein structure was assessed by calculating their relative solvent accessibility (RSA) using FreeSASA.^37^ For each residue, RSA was calculated as the ratio of its absolute solvent-accessible surface area (in Å^2^) to its corresponding maximum allowed solvent accessibilities. Residues were categorized as exposed when RSA was ≥20% and buried when RSA was <20%.

### Cell lines

Human osteosarcoma 143B and human monocytic THP-1 cells, used as non-immune and immune cell models, respectively, as well as human skin fibroblasts from healthy control subjects and from individuals 1 and 5 carrying the p.Ser90Phe (c.269C>T) and p.Ala235Asp (c.704C>A) variants, were cultured under standard conditions (supplemental material and methods).

### Transient expression of WT and mutant *PSMB8* constructs in HEK293T cells

The pcDNA3.1/V5-His TOPO vector encoding human WT *PSMB8*, kindly provided by Elke Krüger, University Medicine Greifswald, Germany, was used as a template to generate nine *PSMB8* variant constructs by site-directed mutagenesis (Table S2).^30^ HEK293T cells were transiently transfected for 24 h using Lipofectamine 2000 (Thermo Fisher Scientific, Carlsbad, CA, USA) following the supplier’s instructions.

### RNA isolation and quantitative reverse-transcription PCR

RNA from six biological replicates from fibroblasts from individual 5 and eight biological replicates from unaffected family members was extracted using the NucleoSpin RNA kit (Macherey-Nagel, Düren, Germany) according to the manufacturer’s instructions. cDNA synthesis was performed using the iScript cDNA synthesis kit (Bio-Rad, Hercules, CA, USA). Relative expression levels of *HSPA5*, *ATF4*, *DDIT3*, and spliced *XBP1* (*sXBP1*) with *SDHA* as a reference gene were determined by RT-qPCR using GoTaq Green Master Mix (Promega, Madison, WI, USA). Primers are listed in Table S3.

### Protein extraction and immunoblotting

To maintain the activity of proteasomes, fibroblasts were lysed by freeze-thaw cycles, and the cytosolic fraction was used for immunoblotting and proteasome activity assays (supplemental material and methods).

### In-gel proteasome proteolytic activity assay and determination of active proteasome subunit abundance

The chymotrypsin-like activity of the proteasome was assessed with an in-gel fluorescence technique (supplemental material and methods). Briefly, lysates were separated by native gel electrophoresis. Gels were incubated in the presence of proteasomal substrate Ac-ANW-AMC, releasing aminomethylcumarin (AMC), which becomes fluorescent after substrate cleavage. Additionally, activity-based probes (ABPs) were used to assess the amount of active proteasome catalytic subunits (supplemental material and methods).

### Complexome profiling

Briefly, cells were mechanically disrupted, and homogenates were solubilized with digitonin and separated by blue native gel electrophoresis. Triplicate gel lanes from 143B and THP-1 cells were further processed. Fibroblasts stimulated with IFNγ from 2 healthy control subjects (one gel lane from each individual) and two gel lanes from individual 5 carrying the p.Ala235Asp variant were analyzed by complexome profiling.^38^^,^^39^ In addition, fibroblasts from individual 1 carrying the p.Ser90Phe variant were analyzed in a separate batch for qualitative comparison of migration patterns and were therefore not included in quantitative analyses. Entire gel lanes were cut into 60 fractions and digested in gel with trypsin, and peptides were subjected to liquid chromatography-tandem mass spectrometry (LC-MS/MS) to analyze the protein composition and abundance in each gel fraction. Protein migration profiles were grouped together based on their similarities using agglomerative hierarchical clustering analysis and visualized as abundance heatmaps. A full description of the technique can be found in the supplemental material and methods.

### Statistics

Protein complex quantifications from complexome profiling data were calculated as the mean protein abundance of fibroblasts from two healthy control subjects and from two replicate cultures from individuals 1 and 5. The sum of all protein abundance values across the 60 fractions was used to calculate the total protein intensities. Relative gene expression from quantitative reverse-transcription PCR (RT-qPCR) was calculated using the comparative Ct (2^−ΔΔCt^) method. Differences between groups were analyzed using a two-sided Student’s *t* test or Wilcoxon test, as appropriate, and corrected for multiple testing. Graph Prism 6 and R were used to visualize the results. Error bars represent the standard deviation.

## Results

### Characterization of individuals with early-onset immunodeficiency and variable systemic inflammation

**Figure 1:**
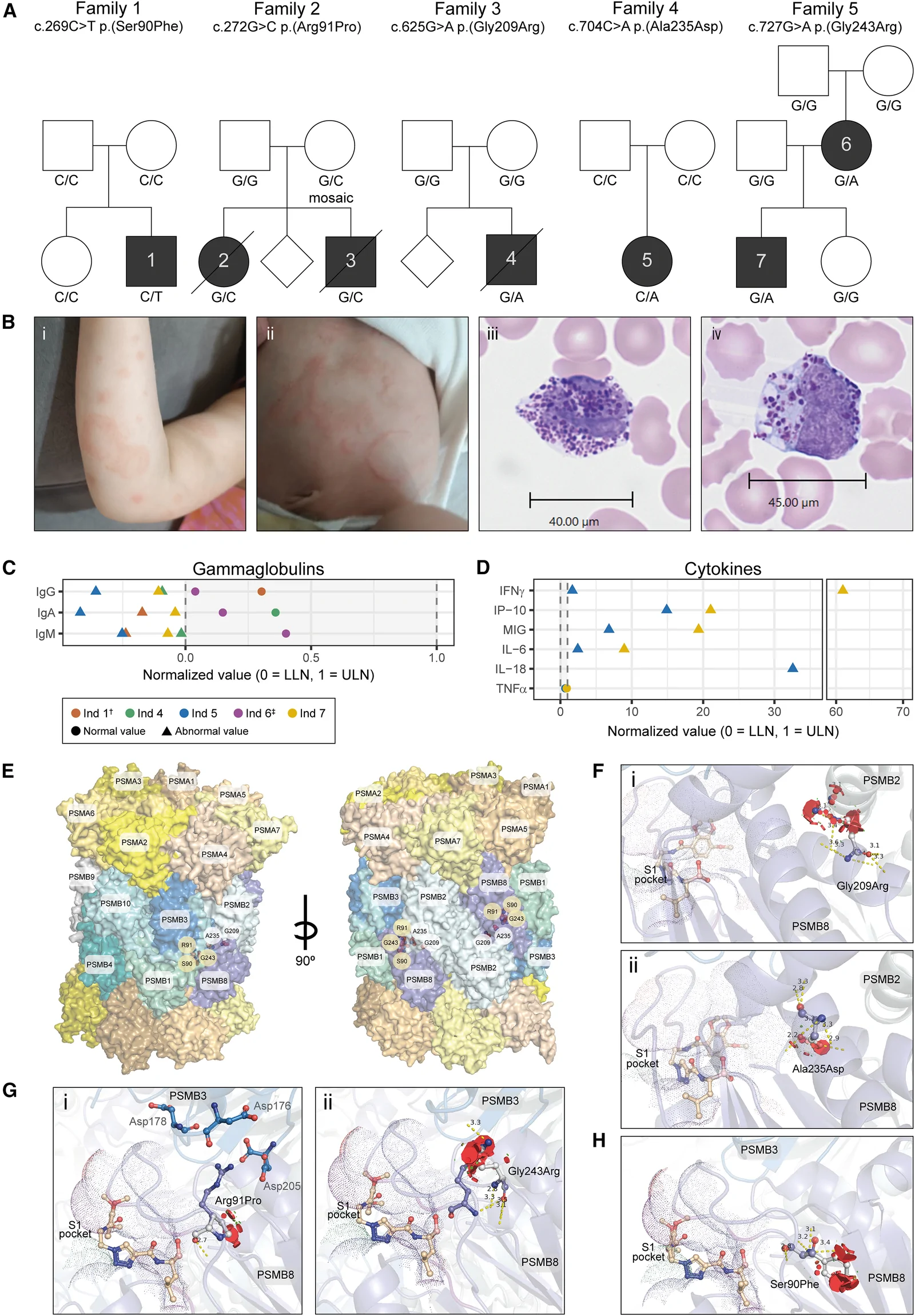
Clinical findings and structural impact of *PSMB8* variants (A) Pedigrees of the five included families with monoallelic variants in *PSMB8* (GenBank: NM_148919.4). Affected individuals are numbered 1–7. (B) Clinical signs and symptoms seen in individuals 4, 5, and 7. Erythematous, edematous plaques on the arm of individual 5 at age 3 years (i). Rash of individual 7 (ii). Peripheral blood cells from individual 4 showing a neutrophil (iii) and a monocyte (iv) with abnormal coarse, deep pink to purple staining inclusions. (C and D) Normalized gammaglobulin and cytokine values. The gray area between the dashed lines represents the reference range, with 0 corresponding to the lower limit of normal (LLN) and 1 to the upper limit of normal (ULN). †, values measured during IVIG treatment; ‡, values of low IgG recorded during childhood. (E) Crystal structure of the 20S immunoproteasome assembly with the variants indicated. (F) Close-up view of the p.Gly209Arg (i) and the p.Ala235Asp (ii) variants located at the PSMB8-PSMB2 interface. (G) Close-up view of the p.Arg91Pro (i) and p.Gly243Arg (ii) variants located at the PSMB8-PSMB3 interface. (H) Close-up view of the p.Ser90Phe variant located near the S1 pocket of the active site. Variants that introduce steric clashes in the neighborhood are indicated as red discs.

We identified seven individuals from five unrelated families with multisystemic inflammation and signs of immunodeficiency from the first year of life (Figure 1A; Table 1). Detailed clinical descriptions are provided in the supplemental note (case reports) and Table S4. The phenotypic presentations of the affected individuals can be placed in a clinical spectrum ranging from mild episodic infections to severe continuous multiorgan inflammation. Individual 6 presented with only skin eruption and did not develop febrile episodes, progressive interstitial lung disease, or bronchiectasis before adolescence. In contrast, individual 4 presented with very early-onset hydrops fetalis and severe pulmonary hypertension necessitating emergency Caesarian section and advanced respiratory support, leading to fatal multiorgan failure at just 2 months of age. The most prominent clinical findings were (inflammatory) lung disease (5/6), lymphopenia (5/5), enteropathy (5/7), transaminitis (4/7), myositis (4/7), and rhabdomyolysis (3/7). Infections were most often caused by viruses, including adenovirus, rhinovirus, enterovirus, parainfluenza virus, and respiratory syncytial virus (RSV), and bacteria, including *Streptococcus pneumoniae*, *Haemophilus influenzae*, and *Haemophilus haemolyticus*. Several individuals (4/7) suffered from periodic exacerbations with fever, recurrent infections with or without skin rash, and transaminitis (Figure 1B). Both severely affected children from family 2 died during infancy due to pneumonia and meningitis, respectively. Postmortem examination revealed basal ganglia calcification in both individuals. Individual 1 underwent lung transplantation due to devastating lung damage caused by necrotizing acute bronchitis and bronchiectasis.

**Table 1:** Demographic, genetic, clinical, and immunological laboratory findings of included individuals

	**Family 1, I1**	**Family 2, I2**	**Family 2, I3**	**Family 3, I4**	**Family 4, I5**	**Family 5, I6**	**Family 5, I7**
**Demographics and genetics**							

Sex	male	female	male	male	female	female	male
*PSMB8* variant (GenBank: NM_148919.4)	c.269C>T (p.Ser90Phe)	c.272G>C (p.Arg91Pro)	c.272G>C (p.Arg91Pro)	c.625G>A (p.Gly209Arg)	c.704C>A (p.Ala235Asp)	c.727G>A (p.Gly243Arg)	c.727G>A (p.Gly243Arg)
Inheritance	*de novo*	inherited from mother (mosaic 3%)	inherited from mother (mosaic 3%)	*de novo*	*de novo*	*de novo*	inherited from mother (I6)
CADD	29.6	29.1	29.1	29.1	29.7	29.7	29.7
AlphaMissense	0.973 (LP)	0.9864 (LP)	0.9864 (LP)	0.842 (LP)	0.987 (LP)	0.897 (LP)	0.897 (LP)
gnomAD AF v.4.1.0	absent	absent	absent	absent	absent	absent	absent
ACMG classification	LP (PS2, PM2_Supporting, PP3_Moderate)	VUS (PM2_Supporting, PP1_Moderate, PP3_Moderate)	VUS (PM2_Supporting, PP1_Moderate, PP3_Moderate)	LP (PS2, PM2_Supporting, PP3_Moderate)	LP (PS2, PM2_Supporting, PP3_Moderate)	LP (PS2, PM2_Supporting, PP1_Moderate, PP3_Moderate)	LP (PS2, PM2_Supporting, PP1_Moderate, PP3_Moderate)

**Clinical features**							

Age at							
Presentation	3 months	7 months	6 months	congenital	3 months	adolescence	2 days
Current	12 years	16 months^∗^	11 months^∗^	68 days^∗^	4 years	34 years	4 years
Recurrent fever	–	+	+	–	+	+	–
Recurrent infections	+	+	+	–	+	+/−	–
Bronchitis (upper respiratory tract)	+	+	–	–	–	–	–
Otitis	+	+	+	–	–	–	–
Skin rash	–	–	–	+	+	+^∗∗^	+
Systemic inflammation	–	+	+	+	+	–	–
Inflammatory lung disease	+	+	+	N/D	+	+	–
Pulmonary hypertension	–	–	–	+	+	–	–
Enteropathy	+	+	–	+	+	–	+
Liver dysfunction	+	–	–	+	+	–	+
Myositis/muscle atrophy	+	–	–	–	+	+	+
Rhabdomyolysis	–	–	–	–	+	+	+
Basal ganglia calcification	–	+	+	N/D	–	–	–
Poor growth/short stature	+	–	–	+	–	+	+
Other	lung transplantation at age 10 years, bilateral conductive hearing loss, multiple skin warts	focal seizures (11 months)	–	prematurity (28 completed weeks’ gestation), hydrops fetalis, cardiac hypertrophy, metaphyseal dysplasia (moth-eaten) long bones	hypothyroidism	late preterm, hypothyroidism	acute myo-pericarditis, hypothyroidism, multiple allergies

**Laboratory results**							

Dyslipidemia	+	N/D	N/D	N/D	+	N/D	–
Autoantibodies	N/D	N/D	N/D	–	–	–	–
Immunoglobulins	↓	N/D	N/D	↓	↓	↓ (transient)^∗∗^	↓
Anemia	+	+	–	+	+	–	+
Thrombocytopenia	+	+	+	+	+	–	–
Lymphopenia	+	N/D	N/D	+	+	+	+
B cells	↓	N/D	N/D	↓	↓	↓	↓
T cells	normal	N/D	N/D	normal	normal	normal	normal
NK cells	↓	N/D	N/D	normal	variable	normal	normal
Eosinophilia	–	–	N/D	–	–	–	+
Leukocyte inclusions	+	N/D	N/D	+	–	N/D	N/D
IFN signature	↑	N/D	N/D	↑	↑	N/D	normal

ACMG, American College of Medical Genetics and Genomics; CADD, Combined Annotation Dependent Depletion; d, days; gnomAD AF, Genome Aggregation Database allele frequency; IFN, interferon; LP, likely pathogenic; N/D, not determined; VUS, variant of uncertain significance; y, years; +, present; –, absent; ↑, increased; ↓, decreased; ^∗^, deceased; ^∗∗^, started during the first year of life.

Immunophenotyping in individuals 1, 4, 5, 6, and 7 revealed significant and repeated reductions in B cell percentages, accompanied by a reduced relative number of memory B cells in individuals 1 and 7 (Table S5). Natural killer (NK) cell percentages were variable across individuals, while T cell percentages remained normal. Additional immunological analyses demonstrated hypogammaglobulinemia (Figure 1C), necessitating ongoing intravenous immunoglobulin (IVIG) therapy. IFN scores were markedly elevated in individuals 4 and 5, mildly elevated in individual 1 (measurement was performed during immunosuppressive treatment following lung transplantation), and normal in individual 7 (supplemental material and methods; Figure S1). Cytokine profiling of individuals 5 and 7 during follow-up suggested marked activation of the IFN pathway, along with innate immune activation, including elevated serum levels of IL-6 and IL-18 (Figure 1D; supplemental material and methods; Table S6).

Hematological abnormalities, other than lymphopenia, included (intermittent) thrombocytopenia (5/7) and anemia (5/7). Strikingly, a bone marrow examination of individual 4 demonstrated moderate dyserythropoiesis, near-absent megakaryocytes, and occasional leukocytes in different developmental stages with vacuoles and coarse, deep pink to purple cytoplasmic inclusions (Figure 1B). Leukocyte vacuoles were also demonstrated in the peripheral blood of individual 1. Furthermore, individual 5 developed a combination of clinical features and laboratory findings that were suggestive of hemophagocytic lymphohistiocytosis, including fever, bicytopenia, hyperferritinemia, splenomegaly, and hypertriglyceridemia, and was treated with corticosteroids.

### Genetic analysis identified heterozygous missense variants in *PSMB8*

Monoallelic missense variants in *PSMB8* were prioritized in all families: c.269C>T (p.Ser90Phe), c.272G>C (p.Arg91Pro), c.625G>A (p.Gly209Arg), c.704C>A (p.Ala235Asp), and c.727G>A (p.Gly243Arg) (Table 1; Figure S2). The variants were confirmed *de novo* in individuals 1, 4, and 5. Individuals 2 and 3 inherited the variant from the unaffected mother, in whom low-level mosaicism was detected in a buccal swab (4 of 143 reads; 3%). In family 5, the variant was confirmed *de novo* in the affected mother (individual 6) and transmitted to the affected child (individual 7). All variants were absent from gnomAD v.4.1.0, predicted to be deleterious by multiple *in silico* predictors, and classified as variants of uncertain significance or likely pathogenic following the ACMG criteria (Table 1).^40^

Given the possibility of digenic inheritance, all other proteasome subunit genes were screened for second hit variants. No rare exonic variants were identified in any family, and in those with genome sequencing, possible pathogenic intronic variants were also excluded. Other genetic findings in the families are included in Table S7. Based on the consistent identification of strong *PSMB8* variants and clinical overlap across families, *PSMB8* was considered the most likely genetic candidate in all cases.

### The identified *PSMB8* variants destabilize the 20S and 26S complexes *in silico*

We investigated the structural impact of *PSMB8* variants within the 20S IP and 26S SP complexes through *in silico* modeling. All residues were retained in the mature protein and were highly conserved, as indicated by ConSurf scores (Figure S3).^41^ Moreover, all variants exhibited increased free energy (ΔΔG) values compared with the WT, indicating a destabilizing effect on both the 20S and 26S complexes (Table S8).

Residues Gly209 and Ala235 are located near or at the PSMB8-PSMB2 β ring interface. Both residues are substituted by larger, charged amino acids, causing steric clashes that likely disrupt interactions with PSMB2 and are incompatible with the surrounding hydrophobic environment (Figures 1E and 1F). In contrast, Ser90, Arg91, and Gly243 are close to the S1 pocket of the active site and the interface with PSMB3 (Figure 1E). Arg91 forms stable salt bridges with aspartates in PSMB3, which are lost upon mutation (Figure 1G). The p.Gly243Arg substitution produces substantial shifts due to the long side chain of the Arg residue that may interfere with residue interactions at the PSMB8-PSMB3 interface (Figures 1G and S4). p.Ser90Phe is likely to exert allosteric effects on the S1 pocket, with the bulky, hydrophobic phenylalanine increasing rigidity of the active site loop, thereby altering local structure and function (Figure 1H).

Moreover, protein frustration analyses revealed that all variants induced changes in neighboring residues by altering local energy frustrations and/or changing local contact densities, rewiring the surrounding structure (Table S8; Figure S5). Together, these data suggest that the monoallelic variants exert their pathogenic effect through broad structural destabilization of the complex, in line with a dominant-negative disease mechanism.

### Monoallelic variants lead to defective IP biogenesis with buildup of assembly intermediates

Complexome profiling, a technique that separates protein complexes under native conditions to analyze their composition and abundance by MS, was employed to assess proteasome assembly. Using a non-immune (143B) and an immune (THP-1) cell line, we first validated the specificity and sensitivity of this method to distinguish IP from SP through clear detection and quantification of IP-specific subunits (PSMB8-10) and SP-specific subunits (PSMB5-7) (supplemental note: additional complexome profiling findings; Figure S6). To enhance IP expression in fibroblasts, IFNγ stimulation was applied, which markedly increased the IP fraction from ∼15% to ∼65% of total proteasome abundance (Figure S7).

**Figure 2:**
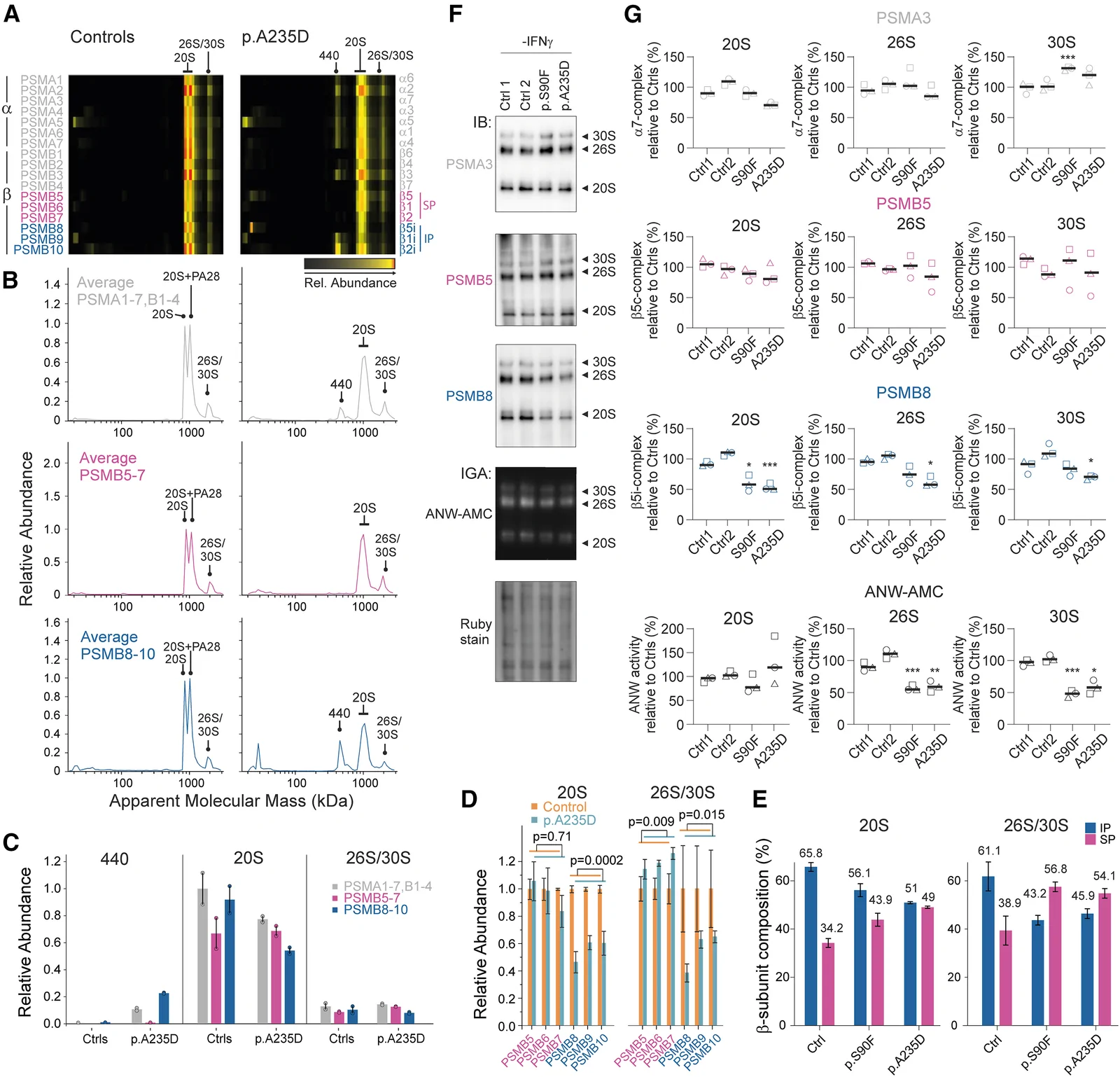
Impaired assembly of immunoproteasomes in fibroblasts carrying *PSMB8* variants (A) Heatmap representation of the migration profiles of α and β subunits showing signals for 20S and 26S+30S proteasome complexes in control fibroblasts and in fibroblasts carrying variant p.Ala235Asp. An additional signal at ∼440 kDa is observed in the variants. For each individual, two lanes of a blue native gel were analyzed. (B) Average migration patterns of α and β subunits shared between the SP and IP (gray), SP-specific β subunits (dark pink), and IP-specific β subunits (blue). (C) Abundance of SP-specific, IP-specific, and shared subunits at the 440-kDa intermediate and 20S and 26S proteasomes∗ relative to the abundance of the 20S complex in the controls (mean ± standard deviation, *n* = 2). (D) Relative quantification of SP- and IP-β subunits at 20S and 26S (mean ± standard deviation, *n* = 2). (E) Percentage of SP and IP content in 20S and 26S proteasome complexes. (F) Immunoblots (IBs) of PSMA3, PSMB5, and PSMB8 and in-gel activity (IGA), followed by cleavage of ANW-AMC, after native separation of fibroblast lysates. This is a representative image of three independent experiments. (G) Quantification of the activity and the abundance in 20S, 26S, and 30S complexes of PSMA3, PSMB5, and PSMB8, as representatives of shared and SP- and IP-specific subunits, respectively (*n* = 3). Quantification of ANW-AMC activity at the 20S, 26S, and 30S proteasome complexes was normalized to total proteasome abundance measured as PSMA3 content. Significance levels are indicated as ns, not significant, ^∗^*p* ≤ 0.05, ^∗∗^*p* ≤ 0.01, and ^∗∗∗^*p* ≤ 0.001. Note: quantification of the 20S and 26S+30S proteasomes also includes assemblies with PA28 and PA200. Mass ranges used for quantification were as follows: 440-kDa peak, 403–514 kDa; 20S proteasome, 774–1,262 kDa; and 26S+30S proteasome, 1,898–2,059 kDa.

Under IFNγ stimulation conditions, fibroblasts carrying p.Ala235Asp showed an approximately 50% decrease in the abundance of the IP-specific 20S and 26S complexes compared with controls, accompanied by a significant reduction of all IP-specific catalytic subunits in both complexes (Figures 2A–2D). In contrast, SP-specific complexes and subunits remained unchanged (Figures 2A–2D). Consequently, the relative abundance of IP compared with SP was lower in the p.Ala235Asp line than in controls (1.4 versus 0.8) (Figure 2E). Fibroblasts carrying p.Ser90Phe showed similarly low relative IP levels (0.7), most likely due to a reduced IP in this sample (Figures 2E and S8). Native immunoblotting under basal conditions confirmed an approximately 50% reduction in PSMB8-containing IP complexes in both variants, whereas PSMB5-containing SP assemblies were not significantly altered (Figures 2F and 2G). Upon IFNγ stimulation, IP formation was partially restored, with a more pronounced compensatory effect in the p.Ser90Phe line (Figure S9). This assembly defect resulted in reduced IP-dependent proteasome activity, with a ∼40% decrease in ANW-AMC activity of 26S and 30S proteasomes after normalization to total proteasome abundance, whereas 20S-associated activity remained preserved (Figures 2F and 2G). Despite reduced activity, no accumulation of total ubiquitinated proteins was detected (Figures S9E and S9F).

**Figure 3:**
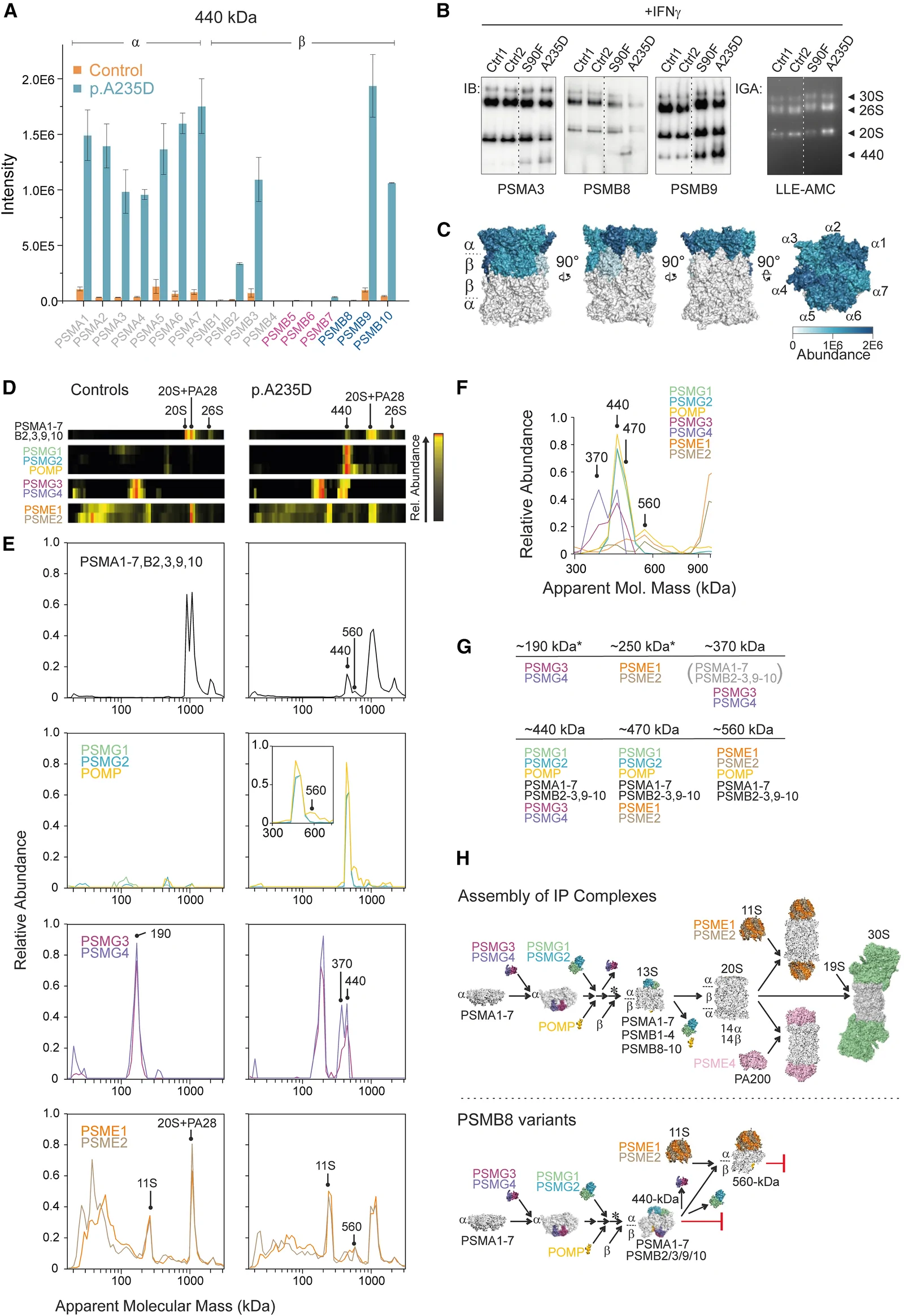
Assembly factors scaffold the accumulated IP intermediate (A) Quantification of α and β subunits at the ∼440-kDa intermediate. (B) Immunoblot (IB) of PSMA3, PSMB8, and PSMB9, showing the accumulation of PSMA3 and PSMB9 at ∼440 kDa, and proteasomal in-gel activity (IGA) developed with the β1 and β1i-specific substrate LLE-AMC. (C) Cartoon representation of the 20S IP based on the cryoelectron microscopy (cryo-EM) structure of the human 20S IP (PDB: 6E5B^32^), where the upper half of the structure, comprising one α and one β ring, is colored by the average protein intensity values observed in the proband-derived samples (*n* = 2). (D) Heatmap representation of the average of α and β subunits that form the ∼440-kDa intermediate (PSMA1–7 and PSMB2–3/9–10), proteasome assembly chaperones PSMG1–4 and POMP, and the components of the 11S heptameric complex, PSME1 and PSME2. The average of two independent experiments is shown. (E) Plots of the average migration profiles of the proteins from (A). (F) Zoom-in of the protein migration profiles around ∼440 kDa showing the stepwise incorporation and release of assembly chaperones. (G) Composition of the accumulated (sub)complexes observed in control subject- and proband-derived fibroblasts. (H) Schematic representation of the IP assembly pathway illustrating differences between controls and variants. In the controls, all β subunits are efficiently incorporated into assembly intermediates, leading to the formation of the 20S core particle and, subsequently, fully assembled proteasomes containing regulatory particles. In contrast, in the variants, defective incorporation of PSMB8 and of β subunits that are incorporated later stalls the assembly process. This leads to the accumulation of an aberrant ∼440-kDa assembly intermediate that retains the assembly factors. In the variant p.Ala235Asp, a portion of this intermediate progresses to form a ∼560-kDa complex, in which the assembly factors, except for POMP, have been replaced with the 11S regulatory particle. This model highlights impaired proteasome biogenesis as a potential pathogenic mechanism.

Consistent with impaired IP assembly, mutant fibroblasts showed a pronounced ∼440-kDa IP-associated peak that was nearly absent in controls, comprising all α subunits together with PSMB2, PSMB3, PSMB9, and PSMB10 (Figures 2A–2C, S8, 3A, and S10). Accumulation of an assembly intermediate was further supported by native immunoblotting of IFNγ-stimulated cells, revealing a band migrating below the 20S complex in mutant lines that contained PSMA3 and PSMB9 but lacked PSMB8 (marked as 440; Figure 3B). Despite the incorporation of PSMB9, no caspase-like activity was detected, indicating that the complex is likely catalytically inactive (Figure 3B). In the complexome profiling data, assembly chaperones PSMG1–4 and POMP co-migrated with the ∼440-kDa intermediate in both mutants (Figures 3D, 3E, and S10). Moreover, an additional peak at ∼560 kDa was detected in p.Ala235Asp, most likely representing a transitional assembly state in which the PSMG1–4 chaperones are displaced by the PA28 heptameric regulator (Figures 3D–3G).

Collectively, these findings indicate impaired IP biogenesis, including reduced formation of mature IP complexes and accumulation of assembly intermediates (Figure 3H). Regulatory particle subassemblies formed independently of the 20S core showed no alterations (Figure S11).

### Accumulation of immature PSMB8 indicates defective proteasome incorporation

**Figure 4:**
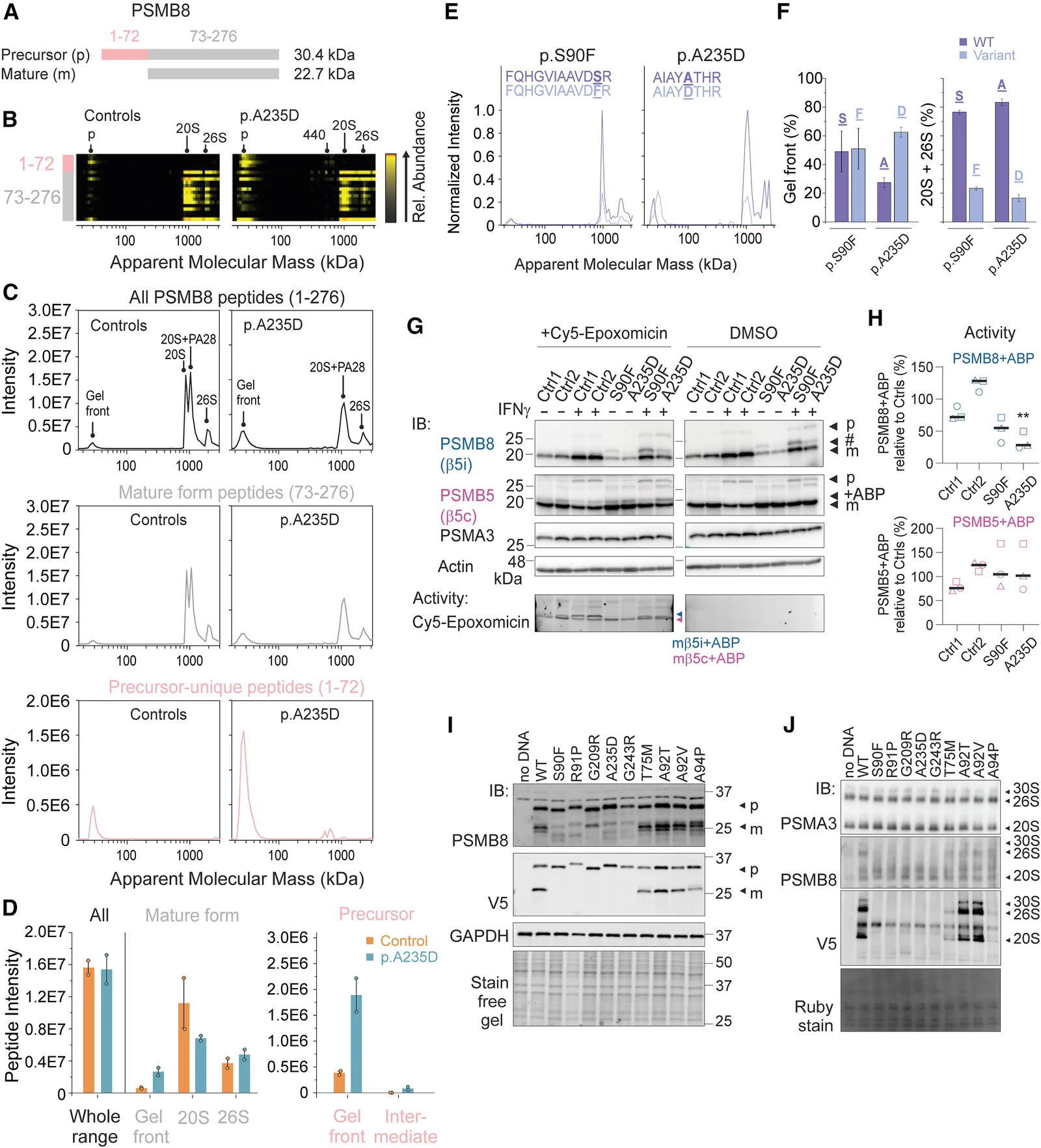
Peptide analysis reveals inefficient maturation and decreased incorporation of p.Alla235Asp variant to 20S and 26S complexes (A) Schematic representation of PSMB8 cleavage site. Theoretical masses were calculated with the Expasy tool compute pI/Mw. (B) Heatmap representation of average migration profiles of all PSMB8 peptides identified (Figures S12A and S12B). (C) Average migration profiles based on the intensity values of all peptides (top), peptides of the mature form (middle), or precursor-specific peptides (bottom). (D) Quantification of all peptides in the whole molecular mass range, mature-form peptide intensities across the gel front and 20S and 26S proteasome fractions, and of precursor-unique peptides at the gel front and at the 440-kDa intermediate. (E) Quantification of wild-type and mutant-specific peptide intensities in controls and in fibroblasts carrying variants p.Ser90Phe and p.Ala235Asp. (F) Percentage of free (migrating at the gel front) and assembled wild-type and mutant PSMB8 into 20S+26S complexes. (G) Immunoblot (IB) analysis of fibroblasts with or without IFNγ stimulation using antibodies against PSMB8, PSMB5, PSMA3, and actin and proteasomal subunit activity. #, this band may reflect an incomplete maturation step. (H) Quantification of PSMB8 and PSMB5 specific activity in samples without IFNγ stimulation. (I) Immunoblot of PSMB8, V5-tag, and GAPDH in HEK293T cells transiently expressing *PSMB8* variants associated with PRAAS-ID and classical PRAAS. (J) Native immunoblot analysis of transfected HEK293T cells from (I), using antibodies against PSMA3, PSMB8, and V5-tag. Data represent the average of two independent experiments. Significance levels are indicated as ns, not significant, ^∗^*p* ≤ 0.05, ^∗∗^*p* ≤ 0.01, and ^∗∗∗^*p* ≤ 0.001.

To further define the molecular basis of the assembly defect, we examined PSMB8 processing and incorporation. Complexome profiling showed similar overall PSMB8 abundance but a 4.5-fold enrichment of PSMB8-derived peptides at the gel front, including precursor peptides, and a 30% reduced incorporation into assembled complexes (Figures 4A–4D and S12). At ∼440 kDa, a minor signal of PSMB8 precursor peptides suggested limited incorporation into this assembly intermediate (Figures 4A–4D). Notably, precursor peptide signals for PSMB9 and PSMB10 were also detected at this position, consistent with the presence of immature catalytic subunits at this assembly stage (Figure S13). Comparison of WT and mutant equivalent peptides showed that the abnormal migration pattern was attributable to mutant PSMB8, which was 4-fold reduced in 20S/26S complexes and accumulated at the gel front in the p.Ala235Asp line (Figures 4E and 4F). High-resolution SDS-PAGE confirmed the accumulation of the PSMB8 precursor with reduced mature protein and revealed an additional band slightly above the mature form detected only in mutant samples (Figure 4G). Accordingly, activity-based probing with Cy5-epoxomicin showed a ∼50% reduction in PSMB8-associated catalytic activity, while PSMB5 activity remained unchanged (Figure 4H). To assess the effect of the remaining variants, we transiently expressed them in HEK293T cells, alongside four structurally proximal variants associated with classical PRAAS. WT and variants associated with classical PRAAS were normally assembled, as indicated by the presence of mature PSMB8 and their detection in proteasome complexes, whereas all monoallelic mutants lacked mature PSMB8, which was also absent in the complexes (Figures 4I and 4J).

Collectively, these data support defective incorporation of mutant PSMB8 into assembling proteasomes, leading to accumulation of free precursor subunits, a mechanism distinct from that observed for the tested classical PRAAS-associated variants.

### Proteasome assembly factors, inflammatory markers, and ISR-related genes are dysregulated in affected fibroblasts

**Figure 5:**
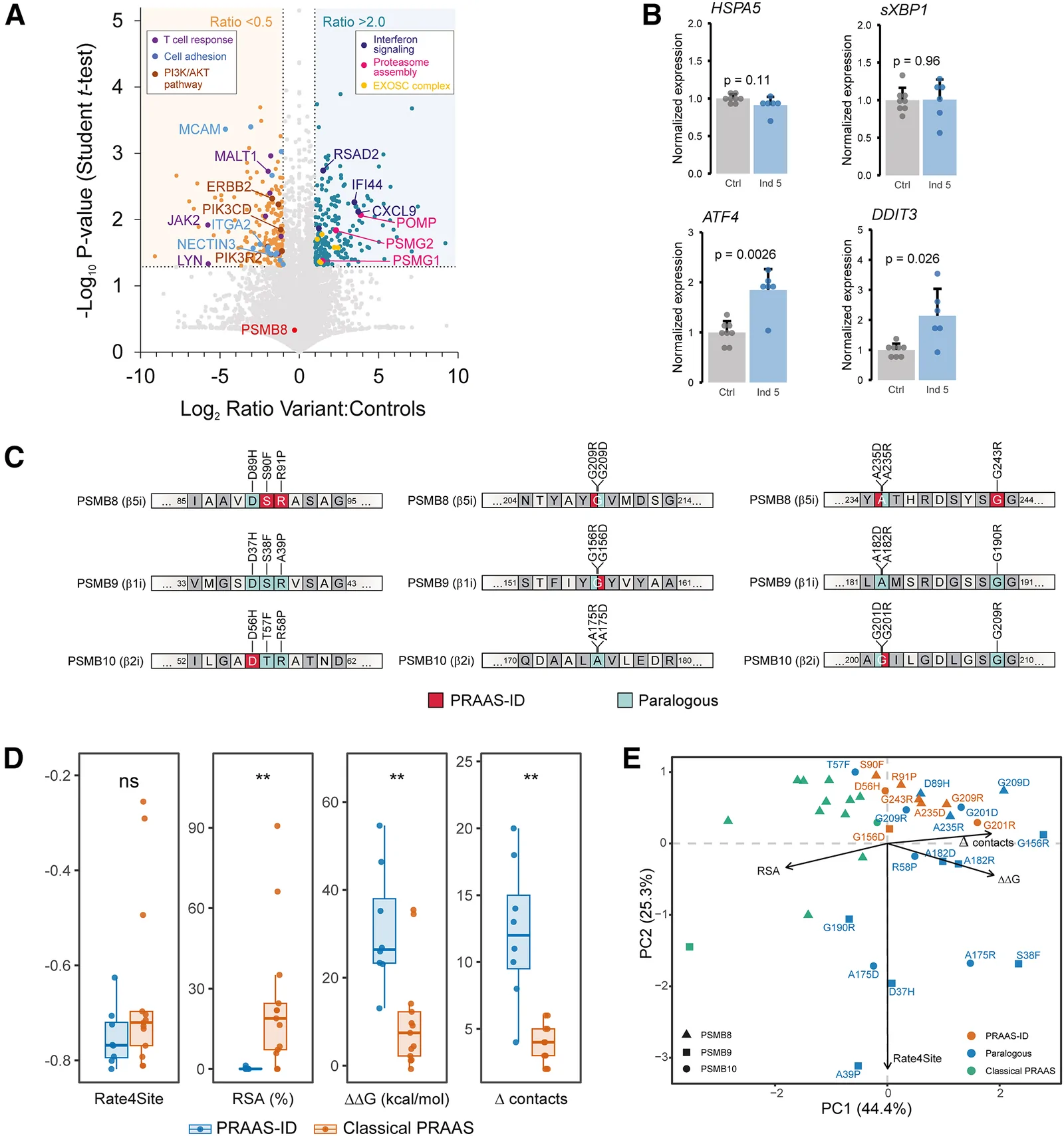
Expression analysis and *in silico* characterization of *PSMB8*, *PSMB9*, and *PSMB10* variants (A) Volcano plot of differential protein abundance in fibroblast expressing the p.Ser90Phe and p.Ala235Asp variants compared with controls (log_2_ fold change versus –log_10_ Student’s *t* test *p* value). Dashed vertical lines indicate expression ratios <0.33 and >3.0. Selected significantly dysregulated genes are highlighted and colored by functional category. (B) RT-qPCR analysis of integrated stress response (ISR) and endoplasmic reticulum stress markers (*HSPA5*, *sXBP1*, *ATF4*, and *DDIT3*) in IFNγ-stimulated fibroblasts from control subjects and individual 5 (Ind 5). Bars show the mean ± SD; dots represent individual measurements. *p* values are shown (two-tailed *t* test). (C) Sequence context of PRAAS-ID-associated variants and evaluated paralogous variants (equivalent amino acid substitutions at aligned positions) in *PSMB8*, *PSMB9*, and *PSMB10*. (D) Comparison of *in silico* scores between PRAAS-ID-associated and classical PRAAS-associated variants for Rate4Site (evolutionary conservation), ΔΔG (predicted destabilization), RSA (relative solvent accessibility), and Δ contacts (number of contacts with changed frustration energies). (E) Principal-component analysis (PCA) of structural and evolutionary features of PRAAS-ID and classical PRAAS variants and PRAAS-ID-derived paralogous variants. Symbols indicate the proteasome subunit, and colors indicate variant class. Arrows represent the contribution of structural and evolutionary variables (RSA, Δ contacts, ΔΔG, and Rate4Site). Significance levels are indicated as ns, not significant, ^∗^*p* ≤ 0.05, ^∗∗^*p* ≤ 0.01, and ^∗∗∗^*p* ≤ 0.001.

Differential protein abundance in fibroblasts with the p.Ala235Asp variant revealed a total of 182 significantly upregulated (fold change ≥ 2) and 157 significantly downregulated (fold change ≤ 0.5) proteins (Table S9). Enrichment analysis indicated upregulation of proteasome assembly factors (PSMG1, PSMG2, and POMP) and stress-related pathways, including components of the RNA exosome complex (EXOSC), as well as immune response genes predominantly related to type I/II IFN signaling (Figures 5A and S14; Table S10). Downregulated proteins were enriched for cell motility and adhesion, T cell responses, and the PI3K/AKT pathway. Previous studies have linked cellular stress to activation of the integrated stress response (ISR) through eIF2α phosphorylation mediated by PKR or GCN2 kinases.^30^^,^^42^^,^^43^ In line with this, and consistent with the proteomics findings, significant upregulation of the downstream ISR targets *ATF4* and *DDIT3* was observed by RT-qPCR. In contrast, expression levels of endoplasmic reticulum stress-associated genes, *HSPA5* and *sXBP1*, remained unchanged (Figure 5B).

### Variants at aligned positions across *PSMB8*, *PSMB9*, and *PSMB10* may exert similar functional effects

Because *PSMB8*, *PSMB9*, and *PSMB10* originated from paralogous gene copies, we aligned their sequences to compare positions of nine reported monoallelic variants (Table S1).^44^ Interestingly, two of the *PSMB8* variants mapped to the same secondary structural positions as described variants in *PSMB9* and *PSMB10*, whereas the remaining variants localized to positions largely conserved among the three subunits (Figures 5C and S15).

To identify features underlying potentially dominant-negative behavior, we assessed structural and biophysical properties of monoallelic variants and compared them with thirteen missense variants associated with classical PRAAS. In general, all pathogenic variants affected evolutionarily conserved residues (Rate4Site score < 0). However, PRAAS-ID-associated variants showed significantly larger predicted energy shifts (ΔΔG), preferential localization to buried sites (RSA < 5%), and disruption of a greater number of contacts showing differences in frustration energies (Δ contacts) (Figure 5D). These features were subsequently evaluated across paralogous variants (equivalent amino acid substitutions at aligned positions) in the 19 subunits constituting the 20S SP and IP. Notably, the structural and evolutionary characteristics were largely conserved across the IP β subunits but substantially weaker across other β and α subunits (Figure S16). Thus, most modeled paralogous variants displayed similar structural and evolutionary properties, suggesting they may have similar functional impacts (Figures 5E and S17; Table S11). However, selected positions deviated from this pattern, including p.Gly190Arg in PSMB9, which is present in gnomAD v.4.1.0, consistent with an expected limited impact on IP assembly. Together, these findings suggest that variants at paralogous positions are susceptible to similar functionally relevant assembly disruption, with structural properties improving predictions of their impact (Figure 5E).

## Discussion

Classical PRAAS caused by *PSMB8* variants is inherited in a recessive manner. Here, we expand this observation by describing five different monoallelic variants in seven individuals, presenting with variable degrees of immunodeficiency and multisystemic inflammation. These variants act through a dominant-negative disease mechanism, causing impaired PSMB8 incorporation, abnormal IP biogenesis, accumulation of dysfunctional assembly intermediates, and cellular stress. Our findings are in agreement with a recent publication that independently described a single dominant-negative *PSMB8* variant causing PRAAS.^30^

By modeling the variants into the IP structure, we found that all variants were associated with structural destabilization and altered residue contacts, supporting a shared mechanism of defective proteasome assembly underlying dominant-negative effects in multimeric protein complexes.^45^ This prediction was experimentally validated, revealing defective IP biogenesis with reduced abundance of fully assembled complexes and impaired maturation characterized by accumulation of stalled assembly intermediates. The primary defect appears to be inefficient incorporation of mutant PSMB8, leading to accumulation of unincorporated protein. Residual incorporation of mutant subunits may further perturb assembly by generating complexes that fail to mature properly or that retain compromised proteasome function. For example, the p.Ser90Phe variant is predicted to perturb the interaction network near the catalytic center and may therefore additionally affect catalytic activity. Several additional observations were consistent with current models of IP assembly. First, the reduction of fully assembled complexes was accompanied by a coordinated decrease of all three IP catalytic subunits, supporting the cooperative incorporation model favoring homogeneous IP formation over mixed complexes.^15^^,^^46^^,^^47^^,^^48^ Second, the composition of the stalled assembly intermediate aligned with the reported sequential order of IP subunit incorporation. This intermediate was also detectable at low levels in controls, indicating a transient physiological assembly step that remains catalytically inactive with uncleaved precursor forms of PSMB9 and PSMB10.^6^^,^^47^ Finally, compensatory SP upregulation, typically observed in knockout models, was not detected despite impaired IP assembly.^46^^,^^49^^,^^50^ This likely reflects preserved PSMB8 expression, with the defect resulting from impaired subunit incorporation rather than its absence.

The mechanism we observed here parallels that proposed for reported monoallelic variants in genes encoding the IP catalytic subunits PSMB8, PSMB9, and PSMB10 and the proteasome assembly factor POMP.^27^^,^^28^^,^^29^^,^^30^ Moreover, assembly defects have been independently demonstrated for the p.Gly209Arg variant in PSMB8 and the p.Gly156Asp variant in PSMB9, which similarly impair incorporation of the catalytic subunits and lead to accumulation of abnormal assembly intermediates.^28^^,^^30^ More broadly, reported monoallelic variants in these catalytic subunits are missense changes that predominantly cluster at the β ring interface and share similar predicted structural consequences, including destabilization and perturbation of residue interactions. Several variants affect equivalent residues across these paralogous proteins, supporting observations that variants at paralogous positions can support the pathogenicity of newly identified variants.^51^^,^^52^ However, some paralogous substitutions showed milder or divergent predicted structural effects, indicating that computational analyses can help refine variant interpretation and guide prioritization for experimental validation. In contrast, classical PRAAS-associated variants were predicted to induce more localized effects, consistent with the normal incorporation of the mutant proteins observed in our transfection assays. Finally, variants affecting *POMP* represent a related but mechanistically broader category. These variants are frameshifts that escape nonsense-mediated decay, resulting in the production of a truncated protein that disrupts both SP and IP assembly pathways.^27^ Taken together, monoallelic variants affecting either catalytic subunits or *POMP* likely act through a shared dominant-negative mechanism by interfering with proteasome assembly.

Clinically, the seven individuals show substantial overlap with PRAAS-ID caused by monoallelic variants in *PSMB8*, *PSMB9*, or *PSMB10*, characterized by neonatal-onset immunodeficiency with recurrent infections and systemic inflammation. In contrast, lipodystrophy or joint abnormalities typical of recessive forms of PRAAS are generally absent (Table 2).^28^^,^^29^^,^^30^^,^^53^^,^^54^ The postmortem basal ganglia calcification in individuals 2 and 3 and the elevated IFN signatures and/or increased serum IFNγ levels in all tested individuals suggested IFN pathway activation, as observed in PRAAS, although less prominent and without clear accumulation of polyubiquitinated substrates.^55^ Immunodeficiency, particularly B cell lymphopenia and hypogammaglobulinemia, is consistent across all monoallelic forms.^28^^,^^29^^,^^30^ In contrast to reports of T cell abnormalities in individuals with monoallelic variants in *PSMB9* and *PSMB10*, T cell populations and mitogen responses were normal in our affected individuals. This difference may in part reflect the distinct composition of the thymoproteasome, in which PSMB8 is replaced by PSMB11 (β5t), the catalytic subunit mediating CD8^+^ T cell selection, whereas PSMB9 and PSMB10 are components of both the IP and the thymoproteasome.^56^^,^^57^ However, T cell lymphopenia and hampered function have also been described for the p.Gly209Arg monoallelic *PSMB8* variant, suggesting a thymus-independent mechanism. The recurrent viral infections in several individuals in our study might also indicate some degree of impaired T cell function. Additional studies are needed to further delineate specific lymphocyte effects. T and B cell abnormalities have also been described in individuals with variants in the proteasome assembly factor *POMP*, who additionally exhibit signs of immune dysregulation such as circulating autoantibodies and hypergammaglobulinemia.^27^

**Table 2:** Comparison of clinical features between monoallelic *PSMB8*-related disorder and other PRAAS syndromes

**Disease**	**Monoallelic**					**Biallelic, oligogenic**	
**Gene(s)**	***PSMB8***	***PSMB8***	***PSMB9***	***PSMB10***	***POMP***	***PSMB8***	***PSMB8*, *PSMB10*, *PSMB4*, *PSMG2*, *PSMA3*, *PSMA5*, *PSMC5***
Ref.	this study	Wolfgramm et al.^30^	Kanazawa et al.,^28^ Kataoka et al.^53^	van der Made et al.^29^	Brehm et al.,^24^ Poli et al.,^27^ Meinhardt et al.^58^	Papendorf et al.,^25^ Arima et al.,^59^ Liu et al.,^60^ Patel et al.^61^	de Jesus et al.,^22^ Brehm et al.,^24^ Papendorf et al.,^25^ Arima et al.,^59^ Liu et al.,^60^ Verhoeven et al.^62^
Total individuals	7	8	3	6	4	20	13

**Clinical features**							

Growth deficiency	+	+	N/R	+	+/−	+	+
Recurrent fever	+	+	+	–	+/−	+	+
Skin rash	+	+	+	+	+	+	+
Systemic inflammation	+/−	+	+	+/−	+/−	+	+
Inflammatory lung disease	+	+/−	+	+/−	+/−	+/−	+/−
Pulmonary hypertension	+/−	N/R	+	–	N/R	N/R	N/R
Enteropathy	+	+/−	N/R	+	+/−	N/R	N/R
Liver dysfunction	+	+	+	+	–	+/−	+
Myositis/muscle atrophy	+	+/−	+	–	–	+/−	+
Rhabdomyolysis	+/−	N/R	+	–	–	+/−	+/−
Basal ganglia calcification	+/−	N/R	+	–	N/R	+/−	+/−
Lipodystrophy	–	+/−	–	–	+/−	+	+
Joint contractures	–	N/R	N/R	–	–	+/−	+

**Laboratory results**							

Dyslipidemia	+	+/−	–	N/R	N/R	+	+
Autoantibodies	–	N/R	–	N/R	+	+/−	+/−
Immunoglobulins	↓	↓	↓	↓	↑	↑ (most)	↑ (most), ↓ (some)
Anemia	+	+	+	N/R	+	+	+
Thrombocytopenia	+	+	+	N/R	+	+ (some ↑)	+ (some ↑)
B cells	↓	↓	↓ or normal	↓	↓	normal or N/R	normal or N/R
T cells	normal	↓ CD4 or normal	↓ or normal	↓, ↑ CD4/CD8	↑ CD4, ↑ CD4/CD8	normal or N/R	normal or N/R
NK cells	variable	N/R	variable	variable	↓	normal or N/R	normal or N/R
IFN signature	↑	↑	↑	↑	↑	↑	↑
Other	hypothyroidism, eosinophilia	thyroid gland agenesis, eosinophilia, nephropathy	abnormal coagulation	eosinophilia	eosinophilia	low TSH	–

+, observed in most individuals; +/−, observed in some individuals; –, not observed; ↓, decreased; ↑, increased; N/R, not reported; normal, within reference range; TSH, thyroid-stimulating hormone.

Of interest are the leukocyte vacuolization and inclusions seen in the blood and/or bone marrow of individuals 1 and 4. These inclusions might reflect accumulation of protein aggregates due to increased ISR activation and proteotoxic stress, reminiscent of lysosomal storage disorders and perhaps similar to the ubiquitin-rich inclusions observed in keratinocytes from individuals with PRAAS.^24^ More studies are needed to unravel the origin and relevance of these leukocyte inclusions in individuals with monoallelic *PSMB8* variants. Nevertheless, vacuolization and the presence of inclusions in leukocytes could be sought as a supportive diagnostic feature.

Our results underscore the added value of complexome profiling relative to conventional methods in the evaluation of the assembly process. Complexome profiling provides high-resolution and comprehensive detail, enabling precise visualization of proteasome assembly intermediates, stoichiometric relationships between subunits, and variant-specific disruptions, especially relevant for assessing the clinical significance of missense variants. Moreover, our findings emphasize that alternative inheritance patterns should be considered when missense variants occur in components of multiprotein complexes, as dominant-negative effects may be involved.

To conclude, we describe seven individuals carrying monoallelic *PSMB8* variants that impair IP assembly, leading to clinically variable immunodeficiency and inflammatory disease, with leukocyte vacuolization and inclusions observed in some individuals. The overlap between clinical features of PRAAS-ID and the shared structural and functional effects caused by monoallelic variants supports IP assembly disruption as a unifying dominant-negative mechanism underlying this group of disorders.

## Data and code availability

The MS data from IFNγ-stimulated fibroblasts are available at the ProteomeXchange Consortium via the PRIDE partner repository. The accession number for the MS data reported in this paper is PRIDE:PXD064505 (https://www.ebi.ac.uk/pride/archive). The complexome profiling dataset is available at the Complexome Profiling Data Resource (CEDAR). The accession number for the complexome profiling data reported in this paper is CEDAR:CRX49 (https://www3.cmbi.umcn.nl/cedar/browse).

## Acknowledgments

The members of the Undiagnosed Diseases Network are listed in supplemental information. We thank the families for participating and for allowing this study to be conducted. We would like to thank Wim A. Dik, Sandra J. Posthumus-van Sluijs, Esther van Rijssen, Evelien Sprenkeler, Laura Lubbers, Laura Batlle Masó, and the Core Facility Mass Spectrometric Proteomics as part of the Technology Platform Mass Spectrometry (TPMS) at the University of Hamburg (UHH) and University Medical Center Hamburg-Eppendorf (UKE) for technical assistance. S.B.W. is a member of the European Reference Network for Rare Hereditary Metabolic Disorders (MetabERN). This work was supported by MetaKids and the United for Metabolic Diseases consortium (UMD-ZOE-2022-012); the 10.13039/501100001659Deutsche Forschungsgemeinschaft (DFG; INST 337/15-1, INST 337/16-1, INST 152/837-1, INST 152/947-1, FOR 5705, 523862973, and 518551069); the BMFTR (German Federal Ministry of Research, Technology, and Space) through the German Center for Child and Adolescent Health (DZKJ) (01GL2404A); the Ricerca Corrente Ministeriale (PerSAIDs project; ERAPerMed2021-262); the 10.13039/100000065National Institute of Neurological Disorders and Stroke of the National Institutes of Health (U01HG007709 and U01HG007942); and the Clinical Translational Core of the Baylor College of Medicine IDDRC (P50HD103555) from the 10.13039/100000071Eunice Kennedy Shriver National Institute of Child Health and Human Development. The content is solely the responsibility of the authors and does not necessarily represent the official views of the National Institutes of Health.

## Author contributions

R.W., C.I.v.d.M., S.B.W., C.M.-S., R.A.W., M.M.O., and S.G.-C. contributed to the conception and design of the study. R.W., M.W.-H., E.J.A., A.G., I.C., Z.S., and M.M.O. performed the genetic data analysis. R.W., C.I.v.d.M., S.K.U., J.A.R., T.P.V., S.K.N., E.J.A., T.E.G., S.V., S.P.K., R.K.A., J.C., and E.D. followed up with the families and collected the clinical data and images. R.W., J.B., M.W., T.P.V., A.C.-O., B.S., T.M., I.G., K.K., R.v.B., and S.G.-C. performed the experimental work. G.R. performed structural biology analysis. R.L.S. performed the cytokine analysis. C.D.M.v.K., M.G., and L.E.L.M.V. provided funding. S.V., M.G., A.H., H.S., S.W.G., L.E.L.M.V., S.B.W., C.M.-S., R.A.W., and M.M.O. provided supervision, either of the study as a whole or of specific aspects such as experiments, clinical follow-up, or data analysis. R.W. and S.G.-C. wrote the initial draft of the manuscript. S.B.W., R.A.W., M.M.O., and S.G.-C. provided critical feedback and assisted with manuscript revisions. All authors read and approved the final version of the manuscript.

## Declaration of interests

The Department of Molecular and Human Genetics at Baylor College of Medicine receives revenue from clinical genetic testing completed at Baylor Genetics Laboratories.

## Web resources

CEDAR, https://www3.cmbi.umcn.nl/cedar/browse
GenBank, https://www.ncbi.nlm.nih.gov/genbank/
OMIM, https://www.omim.org
PDB, https://www.rcsb.org
PRIDE, https://www.ebi.ac.uk/pride/archive

## Supplemental information

Document S1. Figures S1–S18, Tables S1–S3, S6, S7, and S11, supplemental notes, and supplemental material and methods

Document S2. Tables S4, S5, S8–S10, and S12

Document S3. Article plus supplemental information
