# Supplementary material for: Monoallelic PSMB8 variants cause PRAAS with immunodeficiency through impaired immunoproteasome assembly
Source: Am J Hum Genet. 2026 May 21;113(6):1214–32. doi: 10.1016/j.ajhg.2026.04.015 (PMC13277692; doi:10.1016/j.ajhg.2026.04.015)
Supplement: Document S1. Figures S1–S18, Tables S1–S3, S6, S7, and S11, supplemental notes, and supplemental material and methods [file mmc1.pdf]

The American Journal of Human Genetics, Volume *113*

Supplemental information

Monoallelic *PSMB8* variants

cause PRAAS with immunodeficiency

through impaired immunoproteasome assembly

Robin Wijngaard, Caspar I. van der Made, Sema Kalkan Uçar, Gayatri Ramakrishnan, Man Wang, Johannes Brand, Jill A. Rosenfeld, Tiphanie P. Vogel, Sarah K. Nicholas, Monika Weisz-Hubshman, Undiagnosed Diseases Network, Clara D.M. van Karnebeek, Eric J. Allenspach, Taylor E. Gardiner, Sumudu Perera Kimmantudawage, Zornitza Stark, Ruth K. Armstrong, Janine Campbell, Stefano Volpi, Enrico Drago, Marco Gattorno, Alice Grossi, Isabella Ceccherini, Alfredo Cabrera-Orefice, Bente Siebels, Thomas Mair, Hartmut Schlüter, Ruben L. Smeets, Ronald van Beek, Ingrid Goebel, Katrin Küchler, Søren W. Gersting, Alexander Hoischen, Lisenka E.L.M. Vissers, Ron A. Wevers, Catherine Meyer-Schwesinger, Saskia B. Wortmann, Machteld M. Oud, and Sergio Guerrero-Castillo

Supplemental Note: Case Reports

Supplemental Note: Additional complexome profiling findings

Supplemental Figures

- Figure S1. Interferon signature score plots
- Figure S2. Genetic evidence of identified variants
- Figure S3. Variant positions and conservation along the linear protein sequence
- Figure S4. Conformational changes due to p.Gly243Arg
- Figure S5. Structural context of monoallelic variants identified in this study and their associated local frustration changes
- Figure S6. Characterization of standard and immunoproteasome subunits in human osteosarcoma 143B cells and in macrophages derived from THP-1 cells
- Figure S7. Enhanced expression of immunoproteasome-specific subunits in cytokine-stimulated human skin fibroblasts
- Figure S8. Heatmaps and migration profiles of variant p.Ser90Phe.
- Figure S9. Enhanced expression of immunoproteasome-specific subunits in fibroblasts from controls and variants p.S90F and p.SA235D after IFNg-stimulation.
- Figure S10. Accumulation of 440-kDa intermediate in variant p.Ser90Phe.
- Figure S11. Heatmaps of migration profiles of proteasome subunits and assembly factors in fibroblasts
- Figure S12. PSMB8 peptide profiles
- Figure S13. PSMB9 and PSMB10 peptide profiles
- Figure S14. Gene ontology (GO) and Reactome enrichment analysis of differentially expressed proteins.
- Figure S15. Clustal multiple sequence alignment of PSMB8, PSMB9, and PSMB10 protein sequences.
- Figure S16. Comparison of structural and evolutionary features across paralogous positions in proteasome subunits for the same amino acid substitution.
- Figure S17. Structural and evolutionary properties of monoallelic variants and at their paralogous positions.
- Figure S18. Blue native electrophoresis of fibroblasts and molecular mass calibration

Supplemental Tables

- Table S1. Variant curation for *PSMB8*, *PSMB9* and *PSMB10*
- Table S2. Site-directed mutagenesis oligonucleotides
- Table S3. Quantitative reverse transcription PCR primer sequences
- Table S4. Clinical characteristics of included individuals
- Table S5. Laboratory findings of included individuals

Table S6. Quantification of soluble serum factors in individual 5 and 7.

Table S7. Candidate gene variants and rare variants in proteasome subunits

Table S8. Frustration index and proteasomal contacts across wild-type and mutant variants

Table S9. List of significantly upregulated or downregulated proteins in the p.Ala235Asp cell line

Table S10. Enriched Gene Ontology (GO) terms and Reactome pathways in upregulated and downregulated protein sets

Table S11. Predicted structural and biophysical effects of paralogous variants in immunoproteasome β-subunits (*PSMB8*, *PSMB9* and *PSMB10*).

Table S12. Identified proteasome subunits and associated subcomplexes detected in control samples from THP1, 143B, and fibroblast cell lines

Supplemental Methods

Recruitment and ethics approvals

Cell culturing

Protein extraction and immunoblotting

In-gel proteasome proteolytic activity assay

Active proteasome subunit abundance assay

Complexome profiling

Genome sequencing and variant analysis

Cytokine measurements

Supplemental References

Members of the Undiagnosed Diseases Network (Version 3.31.25)

Supplemental Note: Case Reports

Family 1

The proband (individual 1) is the second child of non-consanguineous healthy Egyptian parents. He was born at 39 weeks' gestation following an uncomplicated pregnancy, and his neonatal course was unremarkable. His respiratory illnesses started at 3 months of age, requiring hospitalization. He subsequently had multiple episodes of pneumonia, at times at a monthly frequency, also requiring hospitalization. During early hospitalizations, there was concern for possible myopathy with hypotonia and elevated CK levels but normal EMG. Biopsy findings were suggestive of a muscular dystrophy (with focal loss and disarray of myofilaments and regenerating fibers with large/prominent nuclei). However, he does not currently have hypotonia, weakness, nor elevated CK levels. Growth/differentiation factor 15 (GDF15) was mildly elevated, which could be due to myopathy or chronic inflammation. Developmentally, there were early motor delays due to hypotonia, but he does not currently have developmental issues, only academic concerns likely related to multiple hospitalizations and ADHD. At 2-3 years of age, he presented with bloody diarrhea and was diagnosed with dysentery. Colonoscopy demonstrated ulcerative colitis. He has not required medications for symptom control since the age of 5 years. Poor growth remains a feature, requiring a G-tube for nighttime feeds. He was diagnosed with growth hormone deficiency at age 7 years. He has had intermittent elevations of AST/ALT/GGT. Given his multiple infections, he underwent immunology evaluations starting at age 5 years, which showed low IgG and poor vaccine responses, suggesting common variable immune deficiency (CVID). Further workup has shown a severe antibody defect with pan-hypogammaglobulinemia, low B cells with no switched memory cells, low NK cell numbers, and intermittent mild cytopenias. His NK cell subset distribution is roughly within the normal range, and he has normal NK cell cytotoxicity. He has normal T cells with normal function. A blood smear revealed vacuolated neutrophils. He does not have any history of malignancy, granulomatous-lymphocytic interstitial lung disease, or autoimmune disease. His cardiac evaluation has shown evidence of elevated right-sided pressures in the past, but these have normalized on repeat echocardiography assessments. By age 7 years, his lung damage was significant, with three lobes (right middle, right lower, and left lower) being nonfunctional. Administration of IVIG helped stabilize his lung disease. His airways were diffusely ectatic with mucosal irregularity. He underwent lung transplantation at age 10 years. Pathology evaluation of the explanted tissue revealed severe, variably necrotizing acute bronchitis and bronchiectasis of several medium and small airways, bronchiolectasis without inflammation, and very rare microscopic foci of small airway obstruction reminiscent of bronchiolitis obliterans, with no follicular bronchiolitis.

His history is also significant for bilateral conductive hearing loss and bilateral tympanic membrane perforations. Around 9 years of age he developed multiple warts on the face, neck, and torso. His lung transplant was complicated by respiratory failure requiring two days on extracorporeal membrane oxygenation (ECMO), with a possible stroke event identified on cerebral MRI. At age 11 years, 9 months, his height was 130.6cm (-2.39SD), weight was 29.4kg (-1.69SD); he is normocephalic. The interferon score was mildly elevated, although this measurement was performed during immunosuppressive treatment (Supplemental Methods, Figure S1A).

Family 2

Individual 2 is the first child of non-consanguineous parents of European descent in family 2. The father had no significant medical history. The mother of this family has a history of recurrent ear infections as a child and migraines but has otherwise been healthy with no invasive infection history, growth issues, or significant autoimmune manifestations. The female child (individual 2) was born at term gestation with an uncomplicated pregnancy and unremarkable neonatal course. At 7 months of age, she started to get recurrent ear infections and chronic diarrhea. At 11 months of age, she was admitted to the hospital for a seizure in the setting of viral URI symptoms and acute otitis media (AOM) following 3 days of Augmentin. She was afebrile with eye deviation to the left, clenched fists, and intermittent stiffening of the upper extremities. Infectious workup was unremarkable other than the AOM. Head CT demonstrated bilateral mastoid and middle ear effusions without bone destruction or soft tissue abscess. There was no evidence of intracranial infection, acute intracranial hemorrhage, transcortical infarction, or mass lesion. These findings were confirmed on cerebral MRI. EEG was consistent with focal seizures with central midline interictal discharges. Levitiracetam treatment was initiated, and she received ceftriaxone x2 days for the ear infection with clinical improvement. At 12 months of age, she underwent bilateral myringotomy with tube placement but continued to have persistent otorrhea with cultures including *Haemophilus influenza*. At the age of 16 months, she presented with dehydration, lethargy, tachycardia after one day of diarrhea, rash, fever to 102F and emesis. She rapidly decompensated with multiple seizures, depressed mental status, respiratory depression, and large variations in her heart rate and blood pressure. She required intubation, and despite active resuscitation, her neurological condition deteriorated, resulting in brain death. Life support was withdrawn with the provision of comfort care. At the time of death, respiratory virus PCR panel was positive for adenovirus and rhinovirus. Blood culture was positive *for Streptococcus pneumoniae*. Autopsy revealed acute neutrophilic meningitis suggestive of a bacterial infection. She had no evidence of pneumonia. She was noted to have bilateral basal ganglia calcifications.

Individual 3 is the third child of family 2. He was born at term gestation after an uncomplicated pregnancy. He started to get recurrent ear infections and frequent viral respiratory infections starting at 6 months of age and underwent bilateral myringotomy with ear tube placement at 9 months of age. However, he continued to have chronic otorrhea. Ear culture was positive for *Haemophilus haemolyticus* and treatment with amoxicillin resulted in resolution of the otorrhea. At 11 months of age, he presented with cough, rhinorrhea, fever, respiratory distress and an ear infection for which he had been taking amoxicillin. Respiratory viral PCR panel was positive for rhinovirus/enterovirus and adenovirus. Progressive increase in work of breathing and hypoxia prompted CXR which demonstrated vague opacities in the medial lung bases bilaterally. Emergency intubation occurred when he became unresponsive with agonal breathing following a seizure. He subsequently went into cardiac arrest and despite active resuscitation spontaneous circulation was unable to be achieved and he died. Postmortem bronchoalveolar lavage PCR was positive for adenovirus, rhinovirus, parainfluenza virus, *Streptococcus pneumoniae*, and *Haemophilus influenzae*. Autopsy revealed diffuse, bilateral, panlobar bronchopneumonia, likely bacterial with no evidence of infection elsewhere. Bilateral basal ganglia calcifications were also noted.

<u>Family 3</u>

The proband (individual 4) is the second child to non-consanguineous parents of European descent, delivered at 28+1 weeks' gestation by emergency caesarean section due to abnormal cardiotocography and oligohydramnios, birth weight 864g (9th percentile). The infant had hydrops fetalis and significant persistent pulmonary hypertension requiring advanced ventilation techniques in the early neonatal period. The neonatal period was further complicated by conjugated hyperbilirubinemia with evolving cholestasis and fluctuating transaminitis; hepatosplenomegaly with stable splenic infarct and ascites; biventricular cardiac hypertrophy; and strikingly, 'moth-eaten' long bone changes and metaphyseal splaying on X-ray imaging prompting an extensive (though unrevealing) search for congenital infections. The blood film showed infrequent leukocytes with coarse deep pink to purple staining cytoplasmic inclusions. Persistent cytopenias required near-daily platelet transfusions with more intermittent red cell transfusion. Immunological investigations revealed lymphopenia with a very low percentage of B-cells and low immunoglobulins. The infant was treated empirically with benzylpenicillin on radiographic grounds (though maternal syphilis serology was negative). Bone marrow aspirate demonstrated moderate dyserythropoiesis, near-absent megakaryocytes, and occasional leukocytes with coarse deep pink to purple cytoplasmic inclusions. He died having received the genetic diagnosis, aged 68 days (35+6 weeks' gestation) from multisystem organ failure, with acute cardiac decompensation, having received high dose steroid

therapy (modest improvement in thrombocytopenia) and shortly after starting immunomodulating agents in line with recommendations for treatment of interferonopathy.

Family 4

The proband (individual 5) is the only child of non-consanguineous Caucasian parents, the father has Multiple Sclerosis. The pregnancy, birth, anthropometric birth data, and postnatal adaptation were uneventful. No skin lesions were noted at birth. At the age of 3 months, following the live-attenuated rotavirus vaccination, she developed a gastroenteritis and was admitted to the pediatric intensive care unit with lactic acidosis, elevated transaminases, high triglycerides, increased ferritin, and pancytopenia as well as hypogammaglobulinemia. Hemophagocytic lymphohistiocytosis (HLH) was suspected and she was consequently started on steroid therapy with good response. Blood and urine amino acid analyses were additionally performed to investigate potential inherited metabolic diseases, showing mildly elevated lysine levels, and urinary organic acid analysis revealed dicarboxylic aciduria. Cranial MRI and MR spectroscopy showed no abnormalities. At 5 months of age, she was hospitalized with respiratory distress resulting from Respiratory Syncytial Virus (RSV) infection complicated by pneumonia. She additionally developed rhabdomyolysis and elevated liver transaminases without liver insufficiency. Due to persisting respiratory distress, a high-resolution chest CT and echocardiography were performed at the age of 6 months, detecting bronchiolitis obliterans and pulmonary arterial hypertension. She was started on monthly corticosteroids, intravenous immunoglobulin suppletion therapy (IVIG), and the PDE-5 inhibitor tildenafil. Moreover, ultrasonography and biopsy of the liver showed mild hepatomegaly with mild perisinusoidal and periductal fibrosis. At the age of 19 months she experienced a *Klebsiella pneumoniae* urinary tract infection, again complicated by rhabdomyolysis. Over the subsequent years, she experienced recurrent episodes of elevated transaminases, thrombocytopenia, and rhabdomyolysis, sometimes with diarrhea, during numerous milder episodes often triggered by respiratory tract infections. She had persistently low IgG levels requiring ongoing (IVIG) treatment. These episodes were often accompanied by skin lesions of the face, extremities or the whole body appearing as erythematous, edematous plaques with central blanching that resolved with brown discoloration and subsequent complete disappearance. Skin lesions resembling livedo reticularis were noted recurrently. Additionally, hypothyroidism was diagnosed at the age of 10 months and treated. Currently, at 4 years of age, she shows age adequate growth and development. She has received all vaccinations following the Turkish vaccination program and notably, has not suffered from invasive infections.

Immunological investigations starting from the first year of life showed neutropenia and mild lymphopenia with persistently low B cell and normal T and NK cell numbers. There was a reduction in

IgG, IgA and IgM production. No autoantibodies were detected. T cell populations were not notably different from controls and T cell proliferation in response to PHA stimulation was normal. A diagnostic interferon signature and soluble serum factors were additionally requested as a type I interferonopathy was suspected. Both showed evidence of an interferon type I immune response (Table S1).

<u>Family 5</u>

Family 5 includes two affected family members, mother (individual 6) and child (individual 7). During the first year of life the mother presented with recurrent pruritic polymorphous erythematous eruptions clinically described as erythema marginatum–like, with post-inflammatory hyperpigmentation. Hypogammaglobulinemia in the absence of recurrent infections was reported in the first year of life and diagnosed as transient hypogammaglobulinemia of infancy. In adolescence she developed progressive interstitial lung disease with basal bronchiectasis. Spirometry showed a very severe mixed ventilatory defect. Febrile, antibiotic-related episodes were associated with myalgia, tendinopathy, and marked creatine kinase (CK) elevations up to rhabdomyolysis. She exhibits adult short stature (<−2 SDS) and non-autoimmune primary hypothyroidism treated with levothyroxine.

The affected child was born late-preterm and small for gestational age (SGA) after intrauterine growth restriction (IUGR) and oligohydramnios. At 2 days of age, he developed a pruritic, serpiginous annular eruption consistent with erythema marginatum (Figure 1B), diarrhea and peripheral eosinophilia. Neuroimaging, chest imaging, and abdominal ultrasound were unremarkable.

During the first years, failure to thrive (FTT) persisted, affecting weight (≤−2 SDS) and height (≤-2 SDS), and a hypereosinophilic syndrome emerged with absolute eosinophil counts typically 1.5–4.0×10^3/µL (peak 8.4×10^3/µL), involving the gastrointestinal tract, liver, and skin. Gastrointestinal disease manifested as chronic diarrhea, malabsorption, and iron-deficiency anemia, supported by endoscopic–histologic evidence of an eosinophil-predominant inflammatory colopathy (28 E/HPF). Hepatic involvement was characterized by eosinophilic hepatopathy with fluctuating transaminases. Liver biopsy demonstrated diffuse macrovesicular steatosis with portal eosinophils and reactive hepatocellular changes. Transient elastography showed no fibrosis, and flares were corticosteroid-responsive. Cutaneous disease comprised recurrent pruritic serpiginous annular erythema beginning in the neonatal period; dermatopathology reported spongiotic dermatitis with a dense eosinophilic infiltrate. In view of persistent eosinophilia and multiorgan involvement, mepolizumab (anti–IL-5) was started and associated with an early decline in eosinophils and aminotransferases, followed by later

transaminase rebounds without signs of liver failure. A 3-month course of oral steroid therapy only partially controlled liver enzymes.

At approximately 3½ years of age, after amoxicillin exposure, he developed a drug reaction compatible with eosinophilia and systemic symptoms (DRESS) but without eosinophilia (on mepolizumab treatment) complicated by acute myopericarditis with junctional rhythm, troponin elevation, and cardiac MRI consistent with myocardial–pericardial inflammation requiring admittance to our intensive care unit. He received intravenous immunoglobulin (IVIG) 2 g/kg, high-dose methylprednisolone, and milrinone with full clinical recovery and normalization of the electrocardiogram (EKG) and echocardiography. Across follow-up he also exhibited recurrent hyper–creatine kinase (CK) elevations, mostly during intercurrent infectious (of note these were not more severe or more frequent than in healthy peers of the same age) or inflammatory events.

The immunologic profile indicates a primary humoral immunodeficiency with persistent hypogammaglobulinemia, reduced IgG subclasses 1–3, and poor responses to T-dependent protein antigens; B-cell immunophenotyping shows marked memory reduction—low un-switched memory ($CD27^+IgD^+IgM^+$) and low switched memory ($CD27^+IgD^-IgM^-$)—with expansion of naïve cells and a low CD21low fraction for age (Table S2). Type I interferon signature was negative on several occasions.

Supplemental Note: Additional complexome profiling findings

<u>Complexome profiling analysis on 143B and THP-1 cell lines</u>

To determine whether IP 20S and 26S complexes were properly assembled in fibroblasts from individuals harboring a variant in *PSMB8*, complexome profiling was employed. This mass spectrometric technique separates protein complexes under native conditions to analyze their composition and abundance within a biological sample .

The ability of this technique to detect all proteasome subunits and to differentiate between the SP and the IP was first assessed using a non-immune cell line (143B) and an immune cell line (THP-1). The migration profiles in these cell lines showed two predominant peaks at ~750 kDa and ~2000 kDa, corresponding to the 20S and 26S complexes, respectively (Figure S6A). At the 20S peak, seven α and ten β-subunits were detected, including PSMB5-7 (SP-specific) and PSMB8-10 (IP-specific), indicating the presence of both proteasome types in immune and non-immune cell lines. The 26S complex additionally included ATPase and non-ATPase regulatory subunits from the 19S regulatory particle. A peak around ~1000 kDa, close to the 20S proteasome, was also detected, representing the 20S complex associated with the 11S or PA200 regulatory subunits. Additional peaks corresponding to proteasome subcomplexes were observed and are further detailed below ("Other proteasome assemblies detected in 143B, THP-1, and control fibroblast cell lines") and Table S11.

To differentiate the SP from the IP, we used abundances of PSMB5-7 and PSMB8-10 as SP-specific and IP-specific markers, respectively (Figure S6B). In the 143B cells, 95% of the proteasome corresponded to the SP, whereas in the THP-1 cells, the ratio was inverted, with 80% of the proteasome corresponding to the IP (Figure S6C). Based on these findings, although the apparent molecular masses of the 20S and 26S complexes of SP and IP were similar, we were able to distinguish between these two based on the quantification of the respective specific β-subunits.

<u>Other proteasome assemblies detected in 143B, THP-1, and control fibroblast cell lines</u>

To gain insight into the whole proteasome assembly process, additional patterns observed in the migration profiles and heatmaps were assessed.

In the complexome profiling data, we observed an intermediate of the 20S proteasome assembly in THP-1 and stimulated fibroblast cell lines at very low abundances. This structure contained a complete α-ring and included subunits PSMB2, PSMB3, PSMB9 and PSMB10. The presence of immunoproteasome (IP)-specific subunits PSMB9-10, and the absence of standard proteasome (SP)-specific subunits PSMB5–7, indicated that this intermediate is specific to the IP. The 20S assembly factors POMP and PSMG1–2 were also detected within this intermediate, consistent with their

known role early in proteasome biogenesis and their dissociation later in the assembly process. The abundance of this intermediate was substantially lower than that of the mature 20S and 26S complexes, and it was not detected in the 143B cell line, which is in line with the generally low expression of the IP in this non-immunological cell type.

Regarding the 19S regulatory particles, three distinct subassemblies were detected. Two of these corresponded to base subcomplexes: one composed of PSMC3, PSMC6, and PSMD9, and the other formed by PSMC1, PSMC2, PSMD2, and PSMD5. The third intermediate was part of the 19S lid and comprised subunits PSMD3, PSMD6, PSMD7, PSMD11, PSMD12, PSMD13 and PSMD14. Migration profiles of subunits PSMC1-6 and PSMD2 also showed another peak slightly smaller than the 26S, indicating that these subunits of the base were associated first to the 20S core particle. The complete 19S structure was only observed as part of the assembled 26S proteasome complex. This complex contained the PSMC1-6 subunits forming the ATPase module, as well as non-ATPase subunits previously identified in the subassemblies, along with PSMD1, PSMD4, and PSMD8. Notably, PSMD5, PSMD9 and PSMD10 were absent from the fully assembled 26S complex, consistent with their known roles as assembly chaperones rather than structural components. The only subunit known to be part of the human 26S complex (PDB: 6MSB) that was not identified by LC-MS/MS was SEM1.[1] This protein was not efficiently detected by mass spectrometry due to its small size and the lack of tryptic peptides of a detectable length.

Finally, we also detected subassembly structures of the 11S regulatory particles. At a similar molecular mass, we observed the presence of PSME1, PSME2, and PSME3. These signals corresponded to two distinct 11S complexes: the 11S αβ heteroheptamer formed by PSME1 and PSME2, and the 11S γ homoheptamer formed by PSME3. At the same molecular mass, we also detected the PA200 regulatory particle, formed by a single molecule of PSME4. Both the 11S αβ complex and PA200 were also observed in association with the 20S core particle, alone or as part of hybrid proteasomes incorporating the 20S core particle with the 19S and either 11S αβ or PA200. In contrast, the 11S γ complex was not detected in association with the 20S core particle.

All proteasome structures identified in this study through complexome profiling are presented in Figure S11B and summarized in Table S12.

Supplemental Figures

Figure S1. Interferon signature score plots

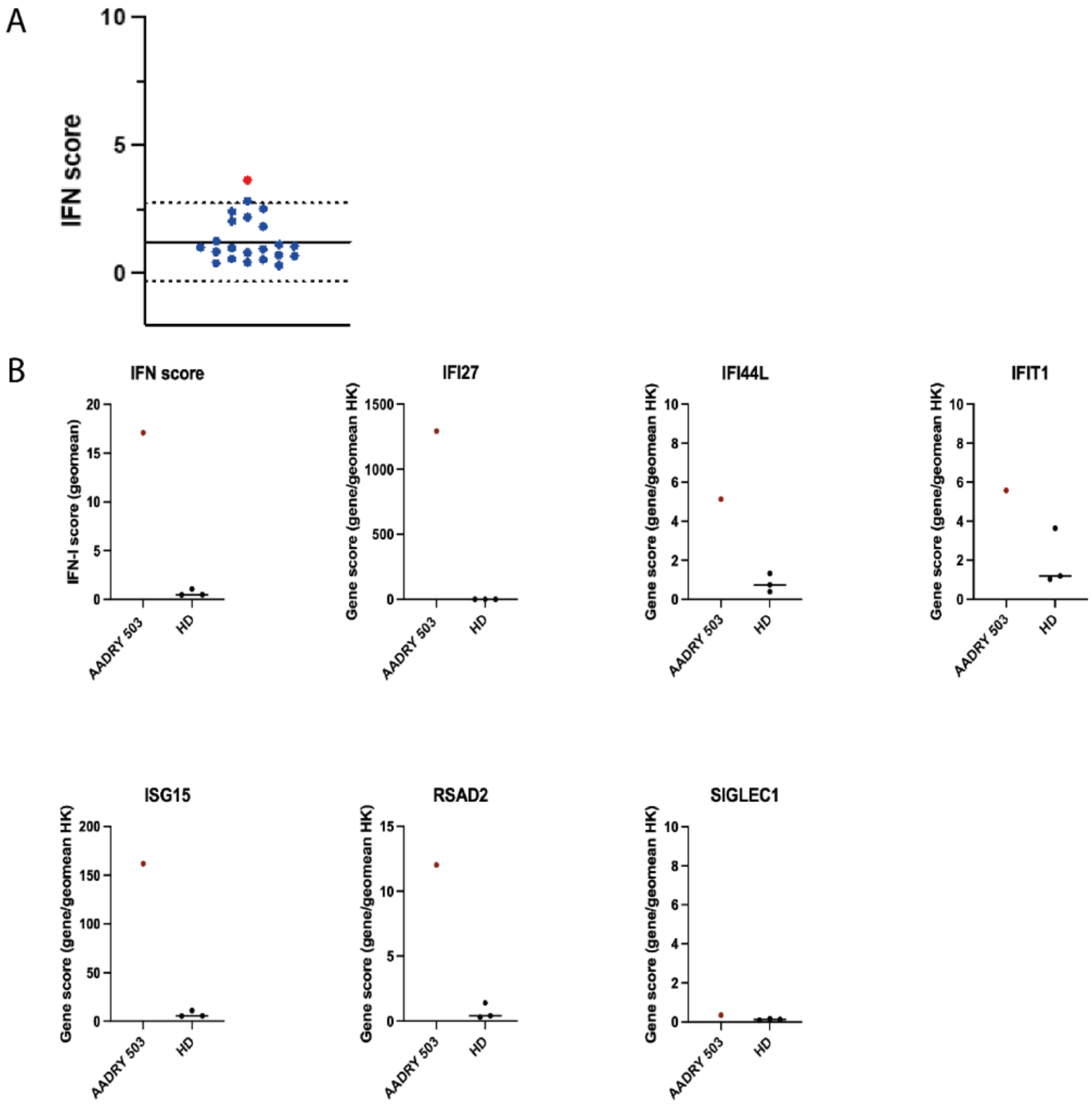

A: Blue are interferon (IFN) scores from healthy controls and red is the IFN score from individual 1, from a post-lung transplant specimen (pre-transplant samples were not available). Solid line represents the mean of healthy controls, dotted lines represent 2 standard deviations from the mean. **B**: IFN score and individual gene scores for individual 4.

Figure S2. Genetic evidence of identified variants

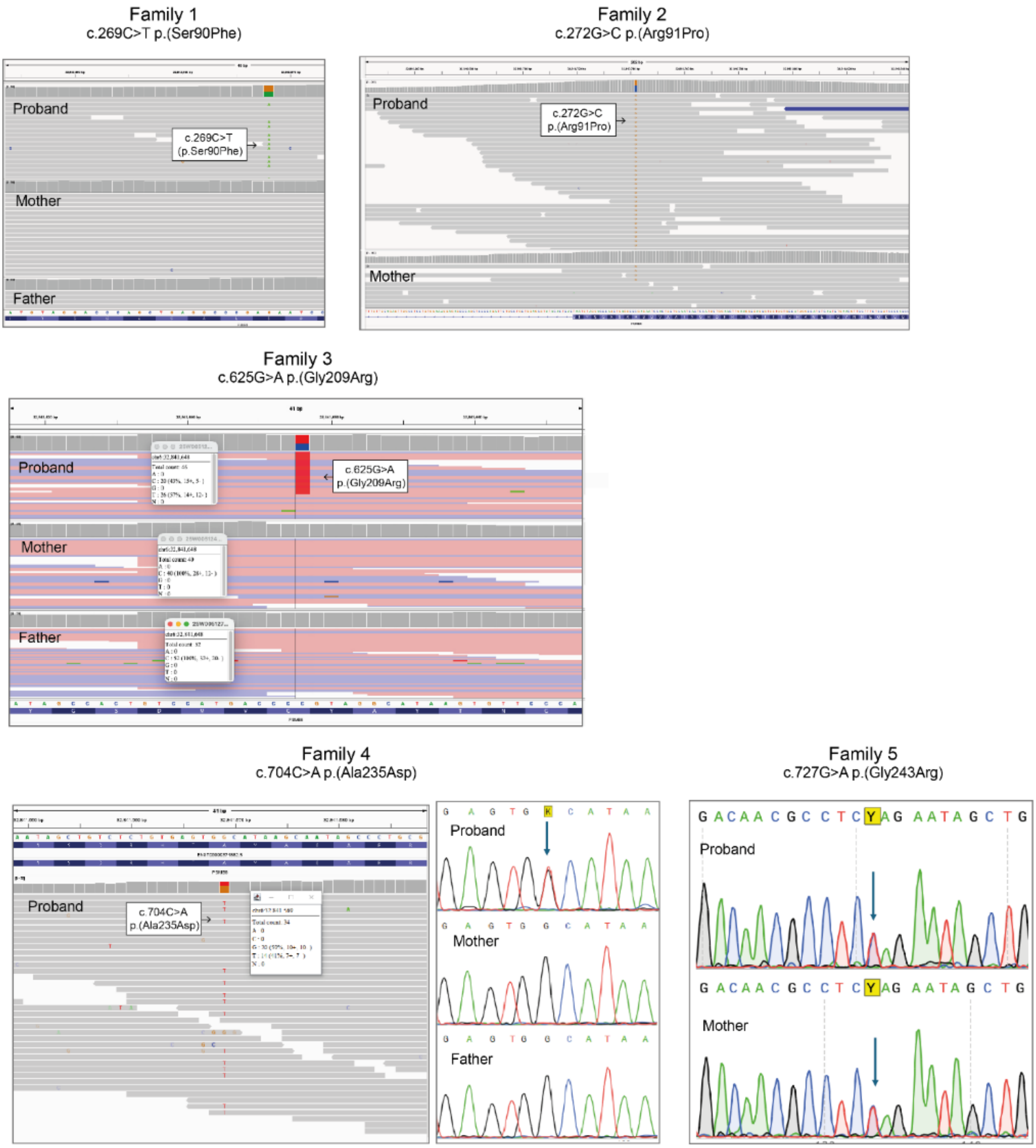

Visualization of the variant in genome sequencing data and/or Sanger sequencing traces in the proband and parents in the five included families.

Figure S3. Variant positions and conservation along the linear protein sequence

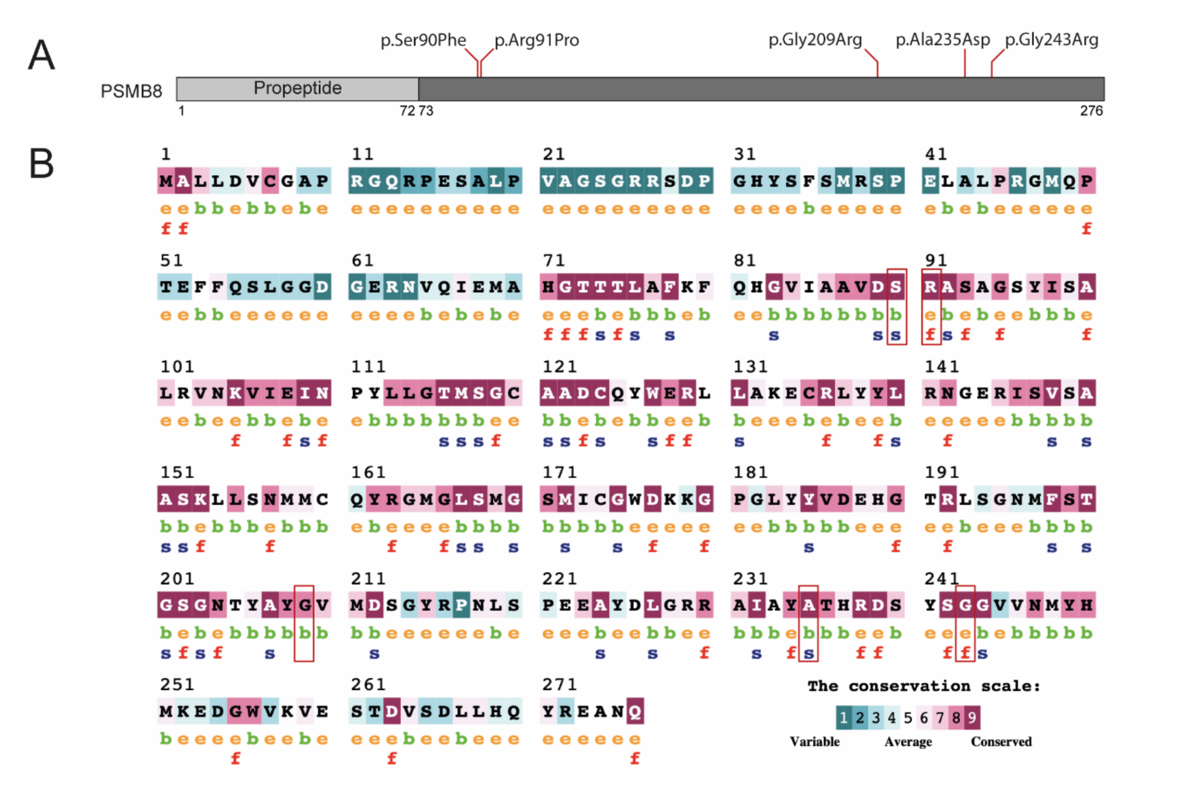

A: Linear protein structure with variant positions. **B**: ConSurf conservation scores with variants highlighted (red boxes). Letters below amino acids indicate: e = solvent-exposed; b = buried; f = functional residue (highly conserved and exposed); s = structural residue (highly conserved and buried).

Figure S4. Conformational changes due to p.Gly243Arg

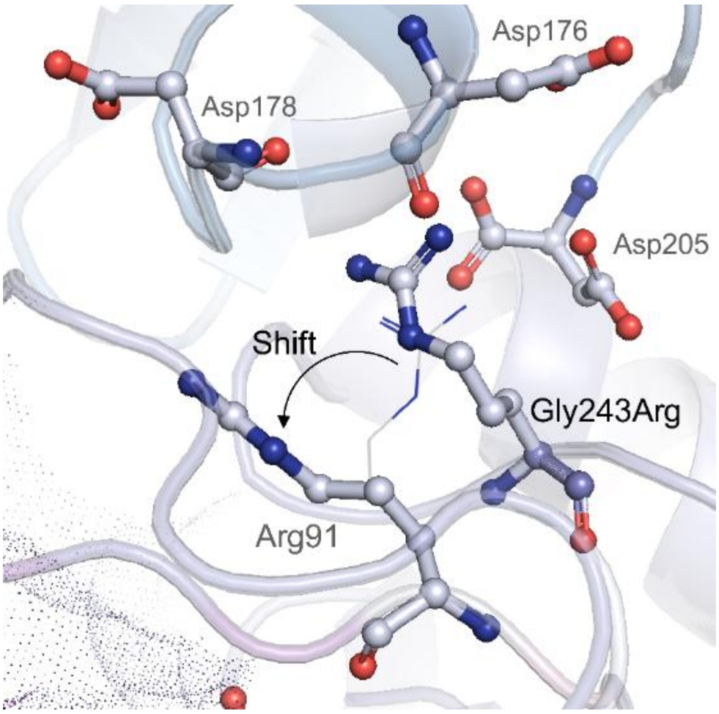

The p.Gly243Arg substitution causes substantial conformational changes in the surrounding residues, resulting in the loss of interactions between Arg91 and the PSMB3 subunit. In place of Arg91, the variant Arg243 establishes salt-bridges with aspartates in PSMB3, while repelling the positively charged sidechain of Arg91. This necessitates structural rewiring in the neighborhood which may be insufficient to fully accommodate the extra positive charge, resulting in significant protein instability.

Figure S5. Structural context of monoallelic variants identified in this study and their associated local frustration changes

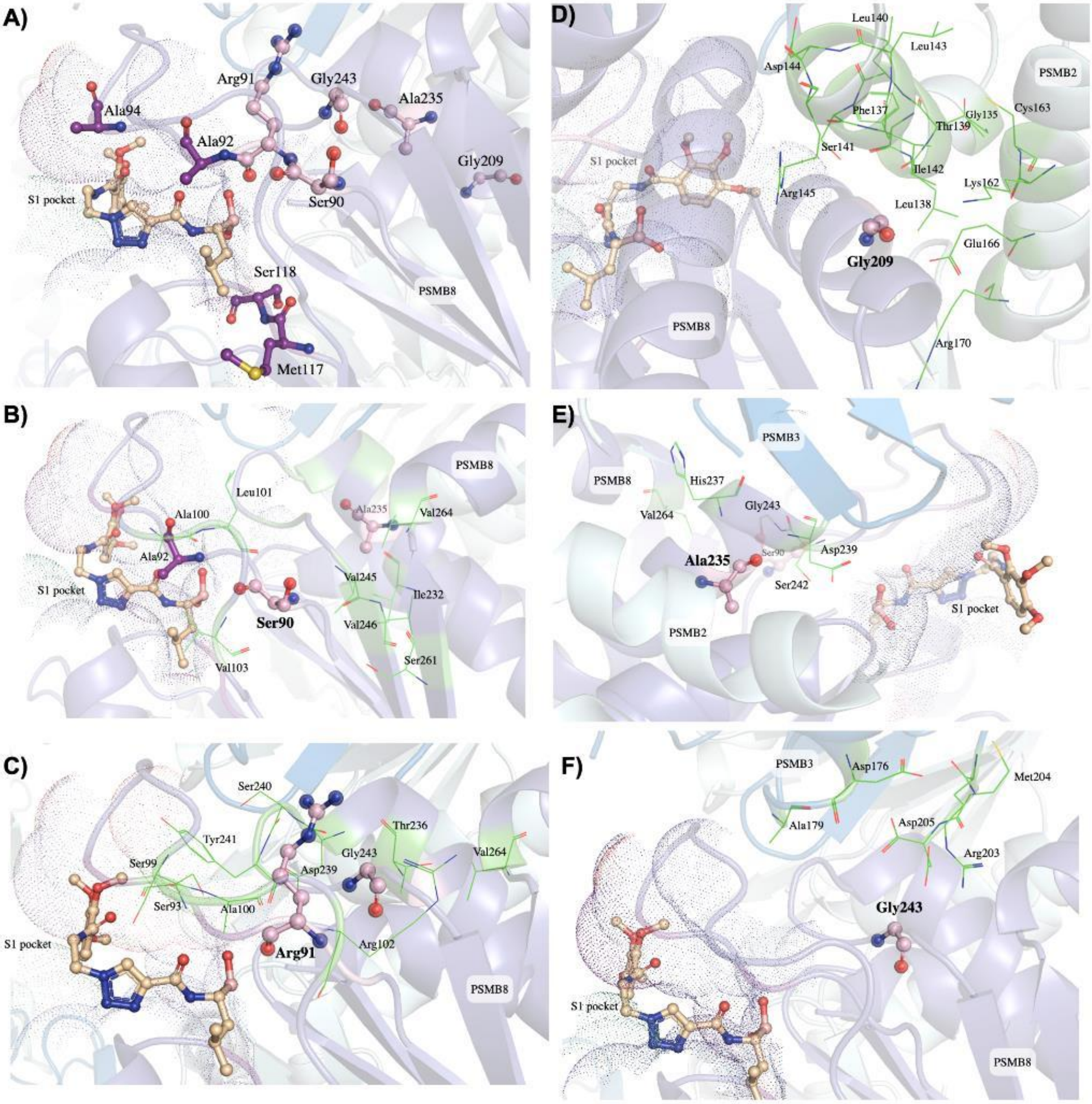

A: Variants assessed in this study are highlighted as ball and sticks. PRAAS-ID-associated variants are colored pink while classical PRAAS-associated variants are shown in violet. Immunoproteasome inhibitor (PDB ID: 6E5B) at S1 pocket is shown and retained in all panels as a reference location point.[2] **B**: Residues that undergo change in frustration energies in the variant p.(Ser90Phe) (neutral/high to minimally frustrated) located 5Å around Ser90 are shown in green. This set also includes the autosomal classical PRAAS-associated variant of interest Ala92 shown in purple. **C-F:** Similarly, residues in the neighborhood around p.(Arg91Pro), p.(Gly209Arg), p.(Ala235Asp), and p.(Gly243Arg) that exhibit changes in frustration energies are shown in green. Table S6 lists the residues in each of the variants' neighborhood.

Figure S6. Characterization of standard and immunoproteasome subunits in human osteosarcoma 143B cells and in macrophages derived from THP-1 cells

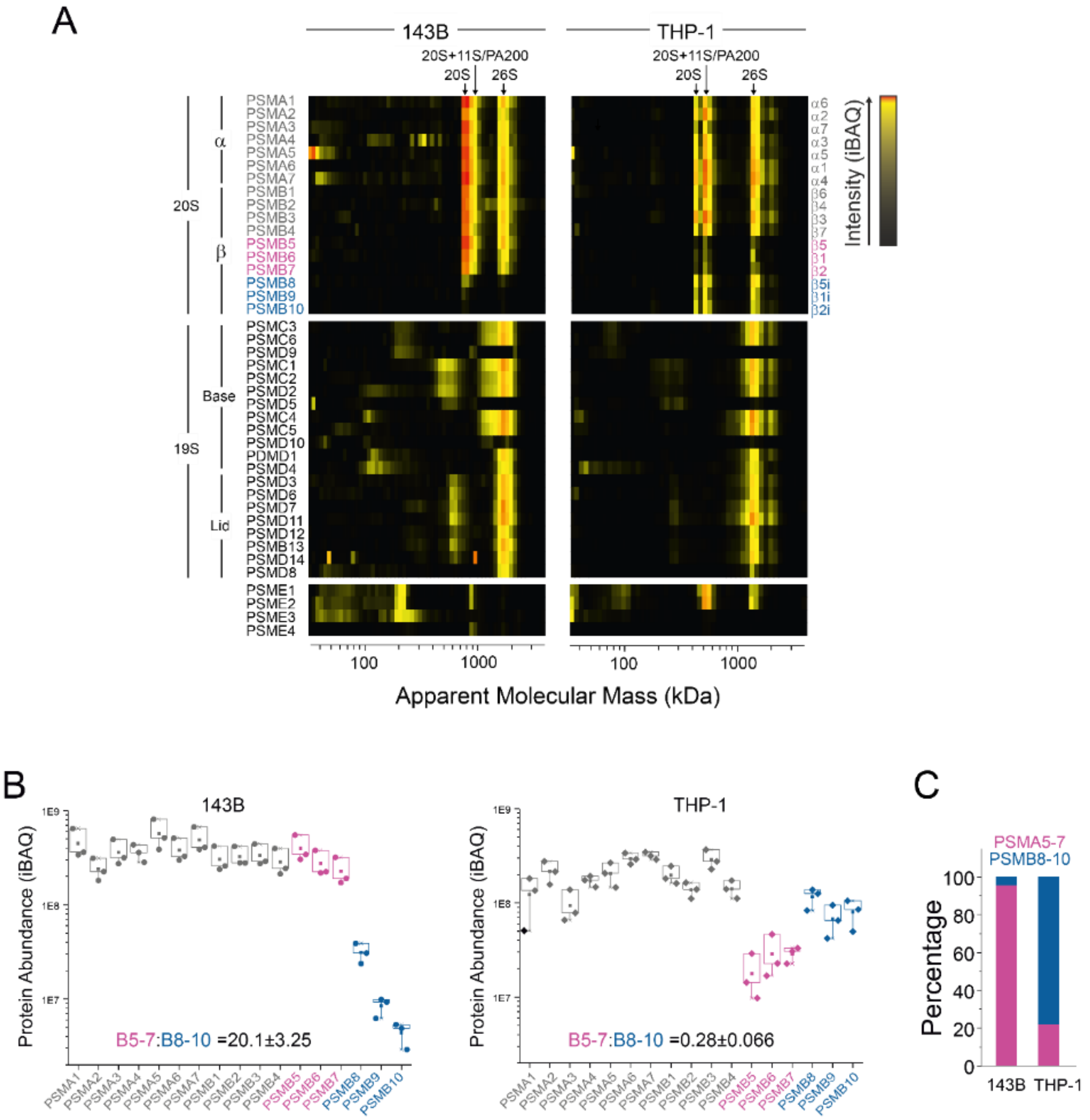

A: Heatmap representation of the migration profiles of 20S proteasome α- and β-subunits and 19S, 11S (PSME1-2) and PA200 (PSME4) proteasome regulatory proteins reflecting total abundances. Average of three independent experiments. **B:** Quantification of α- and β-subunits. Standard proteasomes predominated in 143B cells whereas in THP-1, immunoproteasomes were more prevalent. iBAQ, intensity-based abundance quantification. **C:** Quantification of the percentage of immunoproteasome content in both cell lines.

Figure S7. Enhanced expression of immunoproteasome-specific subunits in cytokine-stimulated human skin fibroblasts

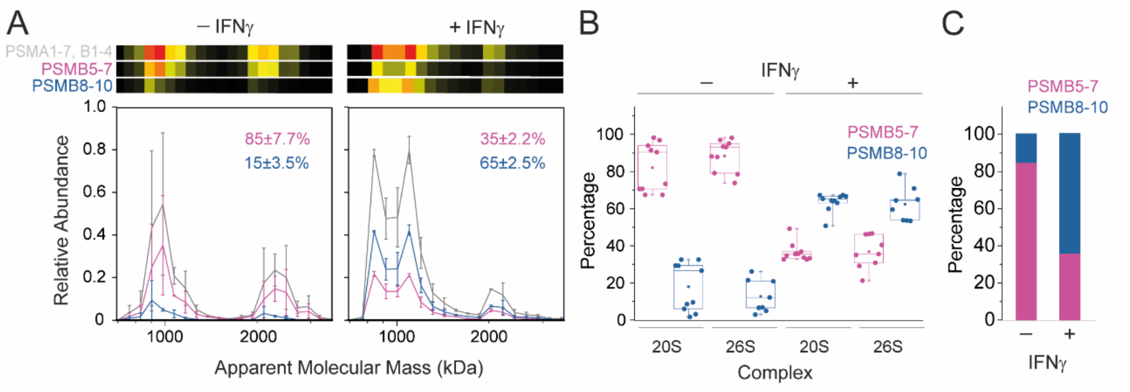

A: Heatmaps of the average migration profiles of shared (PSMA1-7, PSMB1-4), standard proteasome-specific (PSMB5-7) and immunoproteasome-specific (PSMB8-10) subunits in control fibroblasts without and after 48 h incubation with 250 U/mL human recombinant IFNγ prior to harvesting. Average of two independent experiments. **B:** Quantification of the immunoproteasome content in 20S and 26S inferred from SP-specific subunits:IP-specific subunits abundance ratios in gel fractions with the highest intensity values of 20S and 26S complexes. **C**: Quantification of the percentage of immunoproteasome content after IFNγ stimulation.

Figure S8. Heatmaps and migration profiles of variant p.Ser90Phe.

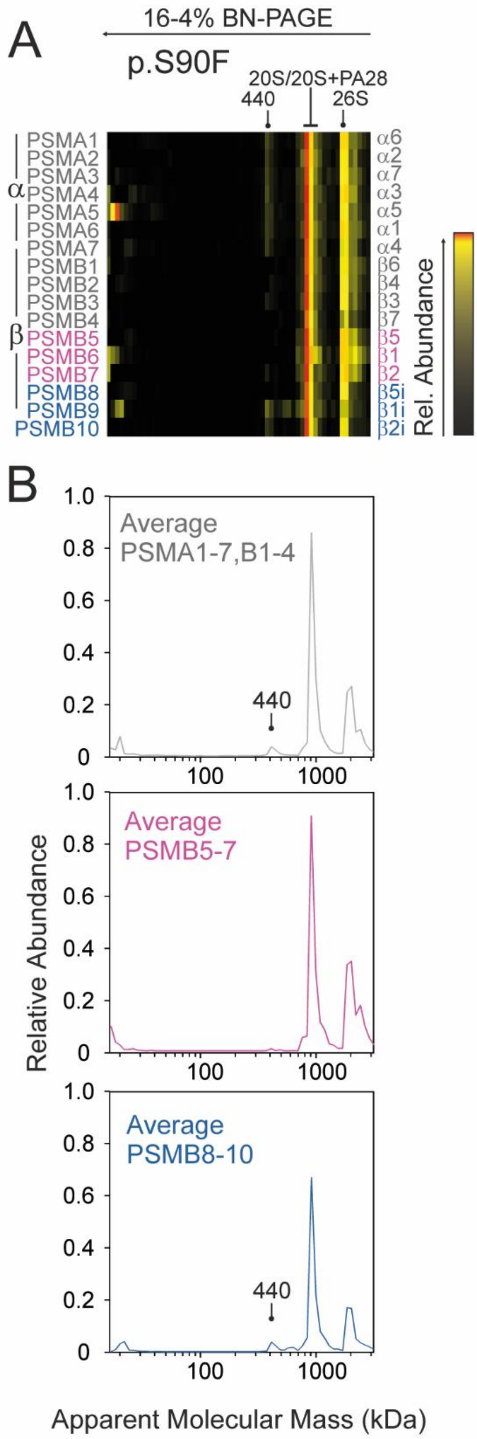

A: heatmap representation of migration profiles of α- and β-subunits showing signals for the 440-kDa intermediate, 20S+PA28 and 26S proteasome complexes in fibroblasts carrying *PSMB8* variant p.(S90F). **B**: Average migration profiles of shared subunits (gray), SP-specific β-subunits (pink), and IP-specific β-subunits (blue).

Figure S9. Enhanced expression of immunoproteasome-specific subunits in fibroblasts from controls and variants p.S90F and p.SA235D after IFNγ-stimulation.

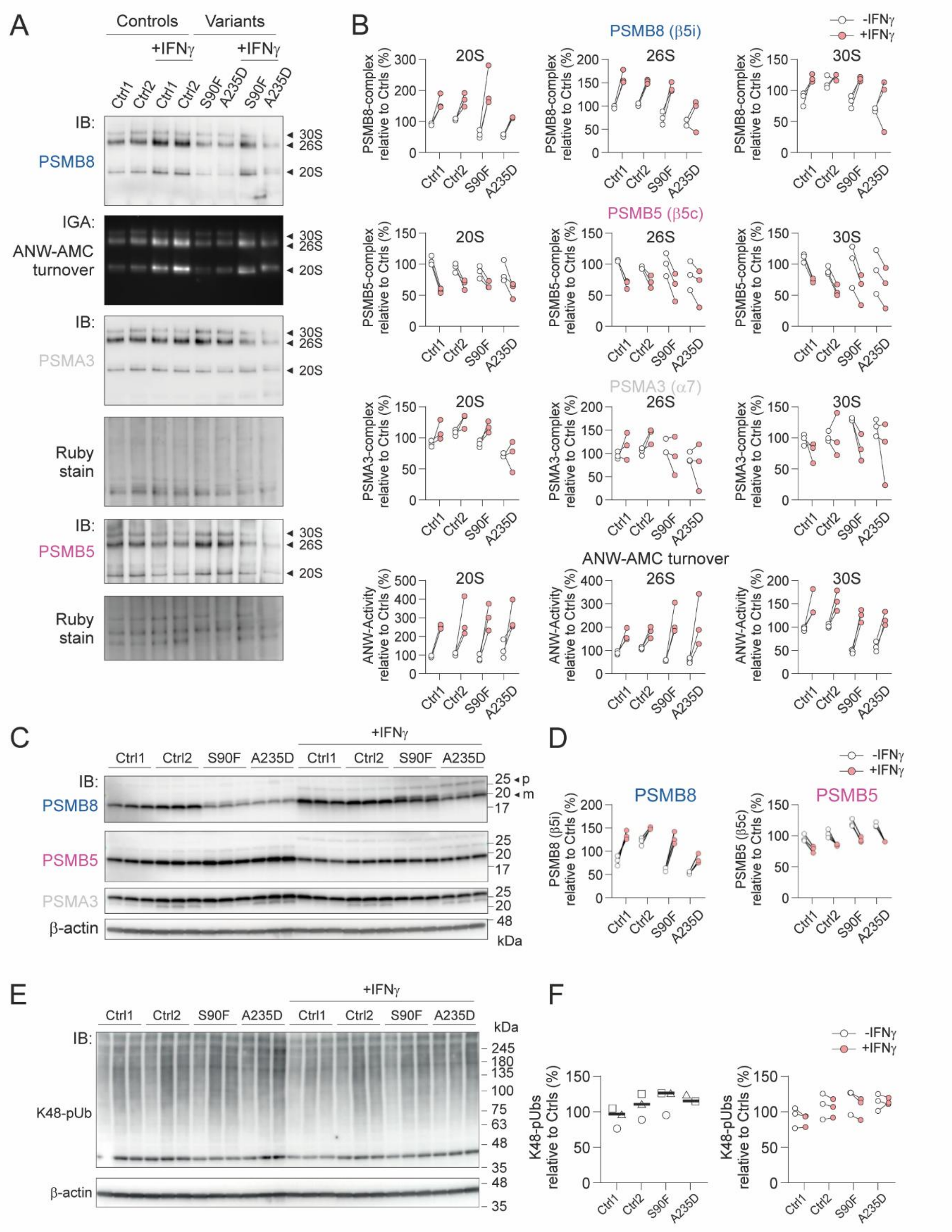

A: Immunoblots (IB) of PSMB8, PSMA3 and PSMB5 and in-gel activity (IGA) after native separation of non-induced and induced fibroblasts with IFNγ for 48 h. Representative blot (n=3). **B**: densitometric quantification of PSMB8 and PSMB5 signals at 20S and 26S complexes relative to non-induced

control fibroblasts. **C**: Immunoblots of PSMB8, PSMB5, PSMA3 and actin after denaturing separation of non-induced and induced fibroblasts with IFNγ for 48 h. Three independent cultures of each individual were induced or not with IFNγ. Precursor (p) and mature (m) PSMB8 forms are indicated. **D**: densitometric quantification of mature PSMB8 and PSMB5 after IFNγ induction for 48 h, relative to non-induced control fibroblasts. E: Protein polyubiquitination assessed by immunoblotting K48-polyubiquitin. **F:** Quantification of K48-poly-ubiquitin across variants and after IFNγ treatment.

Figure S10. Accumulation of 440-kDa intermediate in variant p.Ser90Phe.

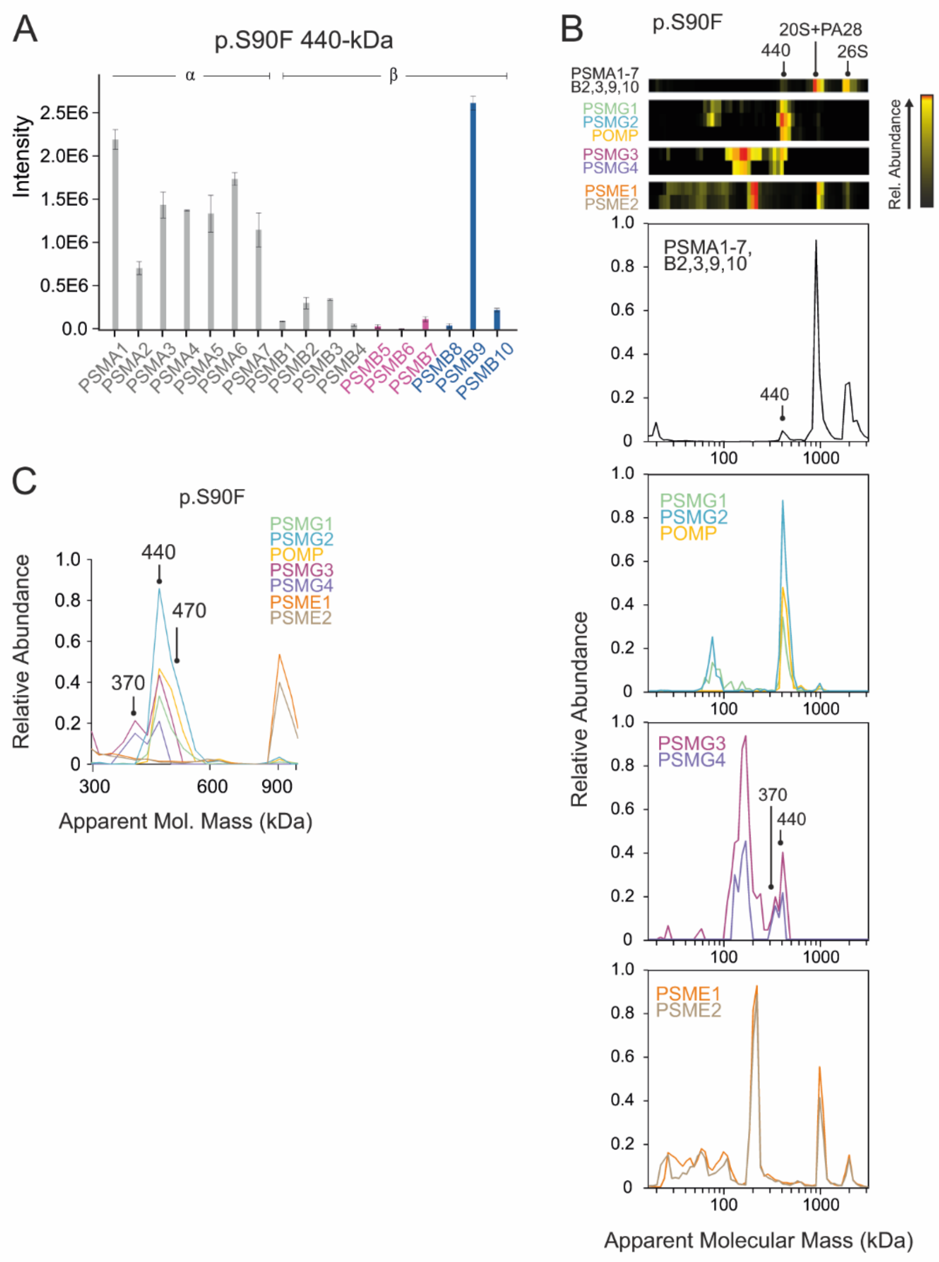

A: Quantification of α- and β-subunits at 440 kDa. **B**: Heatmap representation and migration plots of the average of α- and β-subunits integrating the ~440-kDa intermediate (PSMA1-7, PSMB2-3/9-10), proteasome assembly chaperones PSMG1-4 and POMP, and components of the 11S regulatory particle, PSME1 and PSME2. Average of two independent experiments. **C**: Zoom-in of the protein migration profiles around ~440 kDa showing the stepwise incorporation and release of assembly chaperones.

Figure S11. Heatmaps of migration profiles of proteasome subunits and assembly factors in fibroblasts

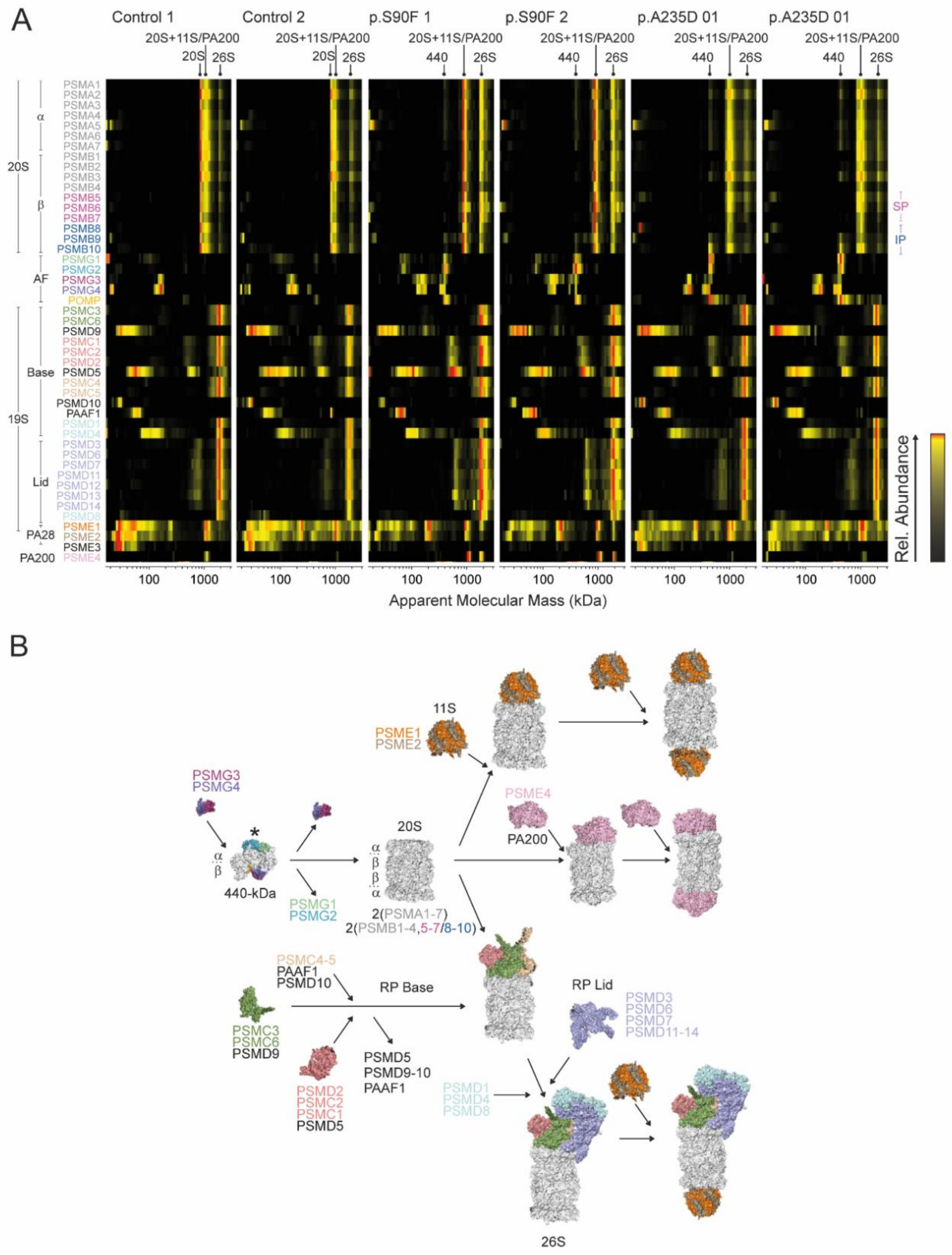

A: Heatmap representation of the migration profiles of proteasome subunits and assembly factors in control fibroblast and from individuals carrying variants p.S90F and p.A235D. Each protein's values were normalized independently, with 0 representing the lowest and 1 the highest value observed for that protein. **B**: Model of proteasome assembly based on intermediates and fully assembled proteasome complexes evidenced by complexome profiling. Subunits forming assembly intermediates are represented as cartoon illustrations, based on cryo-EM structures (PDB: 5GJR, 6E5B and 8QYJ).[1-3] AF: assembly factors.

Figure S12. PSMB8 peptide profiles

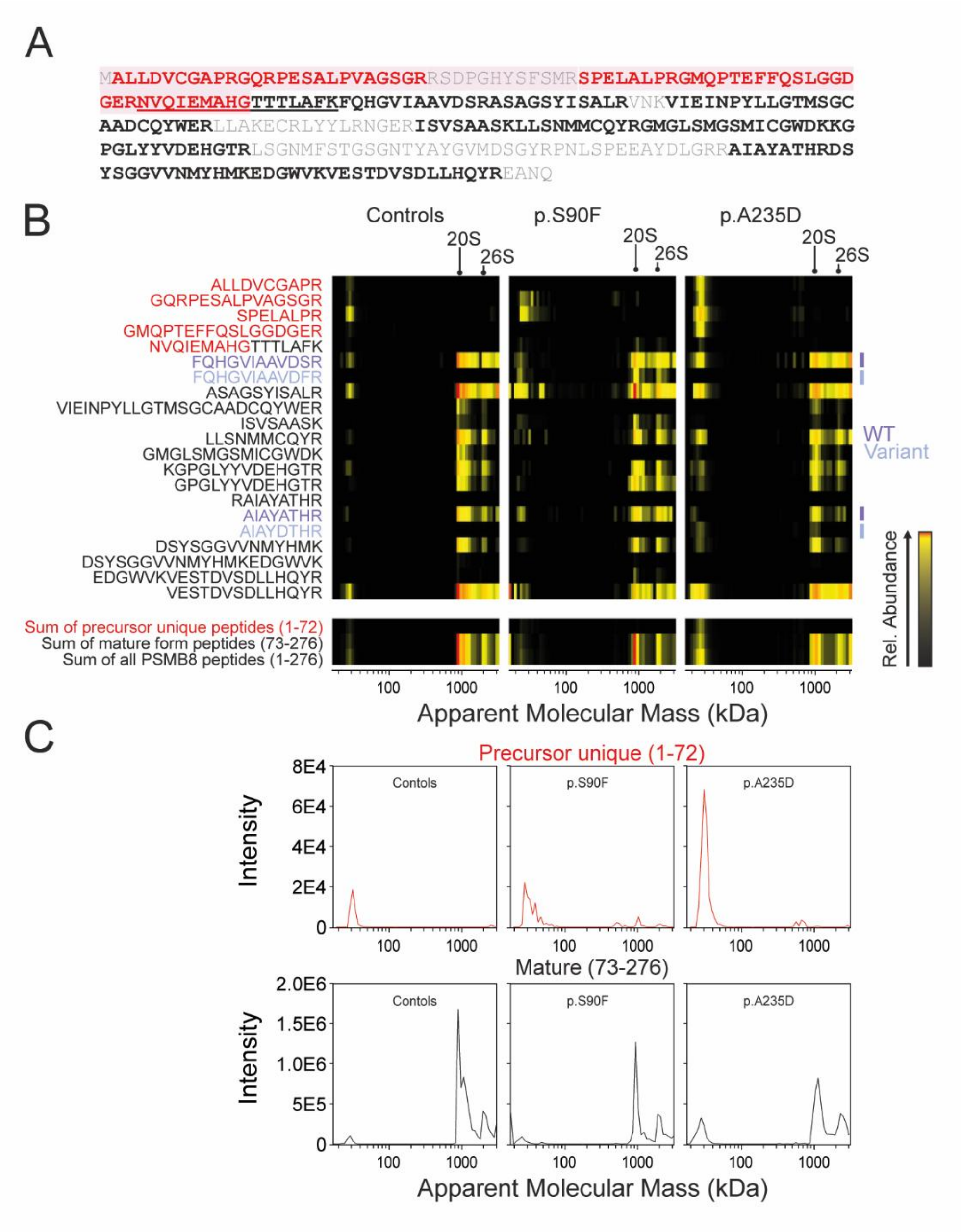

A: PSMB8 protein sequence with the propeptide sequence highlighted in light pink. Detected peptides corresponding to the propeptide are shown in red, while those from the mature form are shown in black. Identified peptide containing the cleavage site is underscored. **B**: Heatmap representation of the peptides identified in the controls and in variants p.Ser90Phe and p.Ala235Asp. **C:** Average migration profiles of precursor-unique peptides and of peptides covering the mature form of PSMB8. Average of two independent experiments.

Figure S13. PSMB9 and PSMB10 peptide profiles

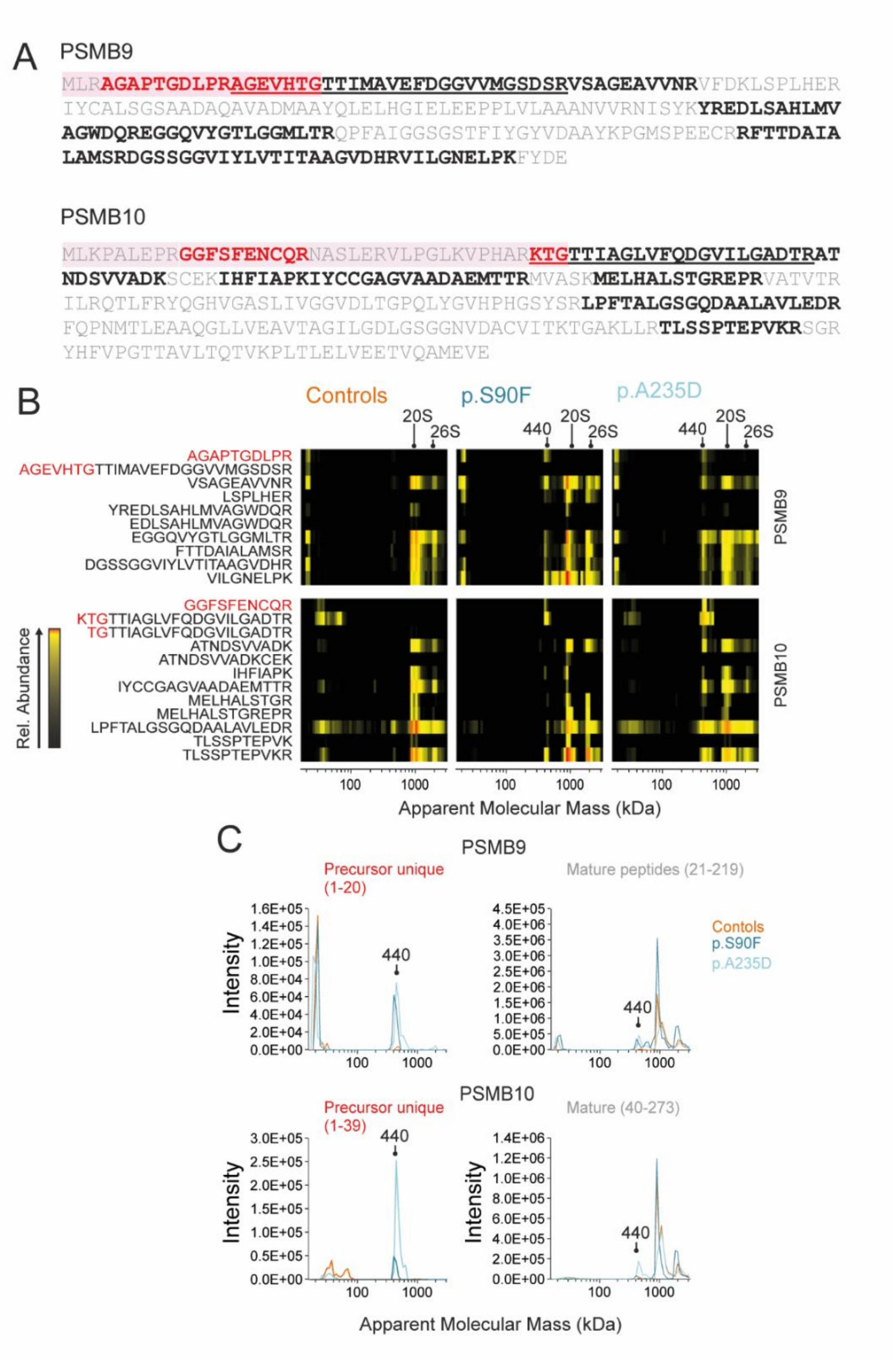

A: PSMB9 and PSMB10 protein sequences with the propeptide sequences highlighted in light pink. Detected premature-unique peptides are shown in red, while those from the mature proteins are shown in black. **B**: Heatmap representation of the PSMB9 and PSMB10 peptides present in the control and in variants p.Ser90Phe and p.Ala235Asp. Average of two independent experiments. **C**: Average migration profiles from precursor-unique peptides and mature PSMB9 and PSMB10 peptides.

Figure S14. Gene ontology (GO) and Reactome enrichment analysis of differentially expressed proteins.

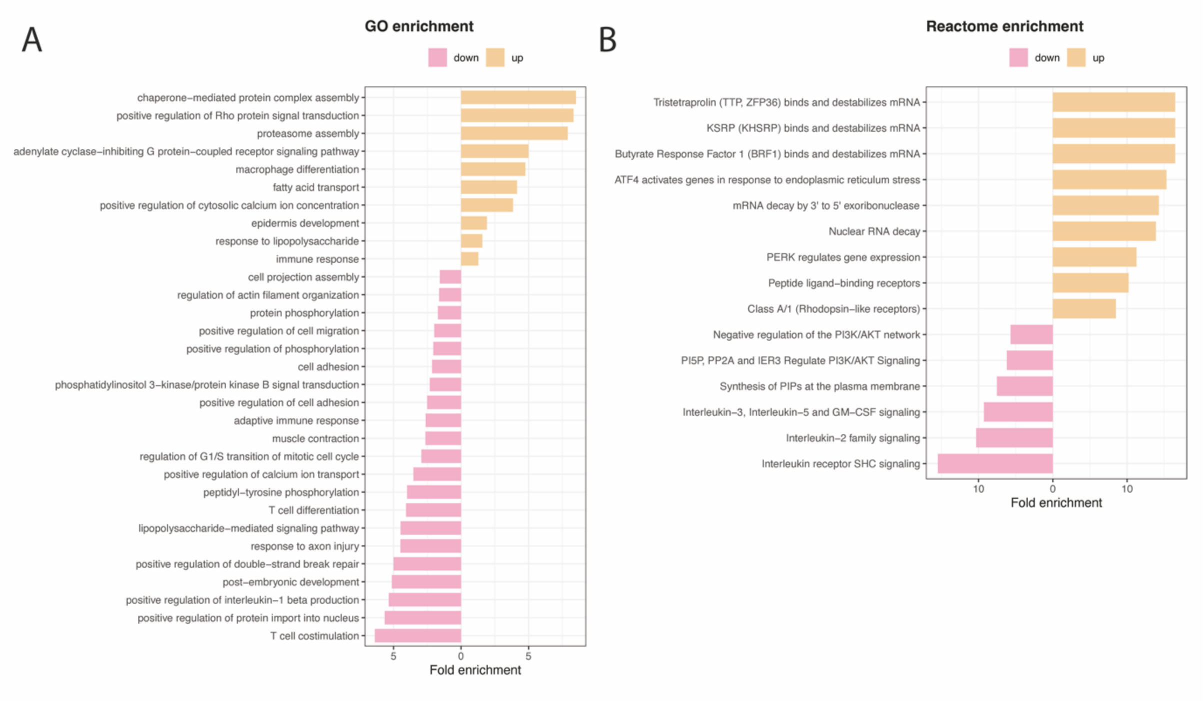

A: GO terms and **B:** Reactome pathways enriched among upregulated and downregulated proteins. Bar length represents fold enrichment (observed/expected).

Figure S15. Clustal multiple sequence alignment of PSMB8, PSMB9, and PSMB10 protein sequences.

```
PSMB10      ------------------------------------MLKPALEPRGGFSFENCQRNASLE      24
PSMB8       MALLDVCGAPRGQRPESALPVAGSGRRSDPGHYSFSMRSPELALPRGMQPTEFFQSLG--      58
PSMB9       ----------------------------------------------------MLRAGA--      6
                                                                  :  .

PSMB10      RVLPGLKVPHARKTGTTIAGLVFQDGVILGADTRATNDSVVADKSCEKIHFIAPKIYCCG      84
PSMB8       -GDGERNVQIEMAHGTTTLAFKFQHGVIAAVDSRASAGSYISALRVNKVIEINPYLLGTM      117
PSMB9       -PTGDLPRAGEVHTGTTIMAVEFDGGVVMGSDSRVSAGEAVVNRVFDKLSPLHERIYCAL      65
                          ***  .. *: **: . *:*.: .. :     :*:  :   :

PSMB10      AGVAADAEMTTRMVASKMELHALSTGREPRVATVTRILRQTLFRYQG-HVGASLIVGGVD      143
PSMB8       SGCAADCQYWERLLAKECRLYYLRNGERISVSAASKLLSNMMCQYRGMGLSMGSMICGWD      177
PSMB9       SGSAADAQAVADMAAYQLELHGIELEEPPLVLAAANVVRNISYKYR-EDLSAHLMVAGWD      124
            :* ***.:    : * : .*: :   .   * :.:.:: :   :*:   :.   :: * *

PSMB10      LTGPQLYGVHPHGSYSRLPFTALGSGQDAALAVLEDRFQPNMTLEAAQGLLVEAVTAGIL      203
PSMB8       KKGPGLYYVDEHGTRLSGNMFSTGSGNTYAYGVMDSGYRPNLSPEEAYDLGRRAIAYATH      237
PSMB9       QREGGQVYGTLGGMLTRQPFAIGGSGSTFIYGYVDAAYKPGMSPEECRRFTTDAIALAMS      184
                        *      :   ***.    . ::  ::*.:: * .  :   *:: .

PSMB10      GDLGSGGNVDACVITKTGAKLLRTLSSPTEPVKRSGRYHFVPGTTAVLTQTVKPLTLELV      263
PSMB8       RDSYSGGVVNMYHMKEDGWVKVESTDVSDL-------LHQYREANQ--------------      276
PSMB9       RDGSSGGVIYLVTITAAGVDHRVI-LGNEL-------PKFYDE-----------------      219
             *  *** :    :.  *                    :

PSMB10      EETVQAMEVE       273
PSMB8       ----------       276
PSMB9       ----------       219
```

Residues reported to harbor PRAAS-ID-associated variants are highlighted in blue. Highlighted positions correspond to Ser90, Arg91, Gly209, Ala235, and Gly243 in PSMB8, Gly156 in PSMB9, and Asp56 and Gly201 in PSMB10.

Figure S16. Comparison of structural and evolutionary features across paralogous positions in proteasome subunits for the same amino acid substitution.

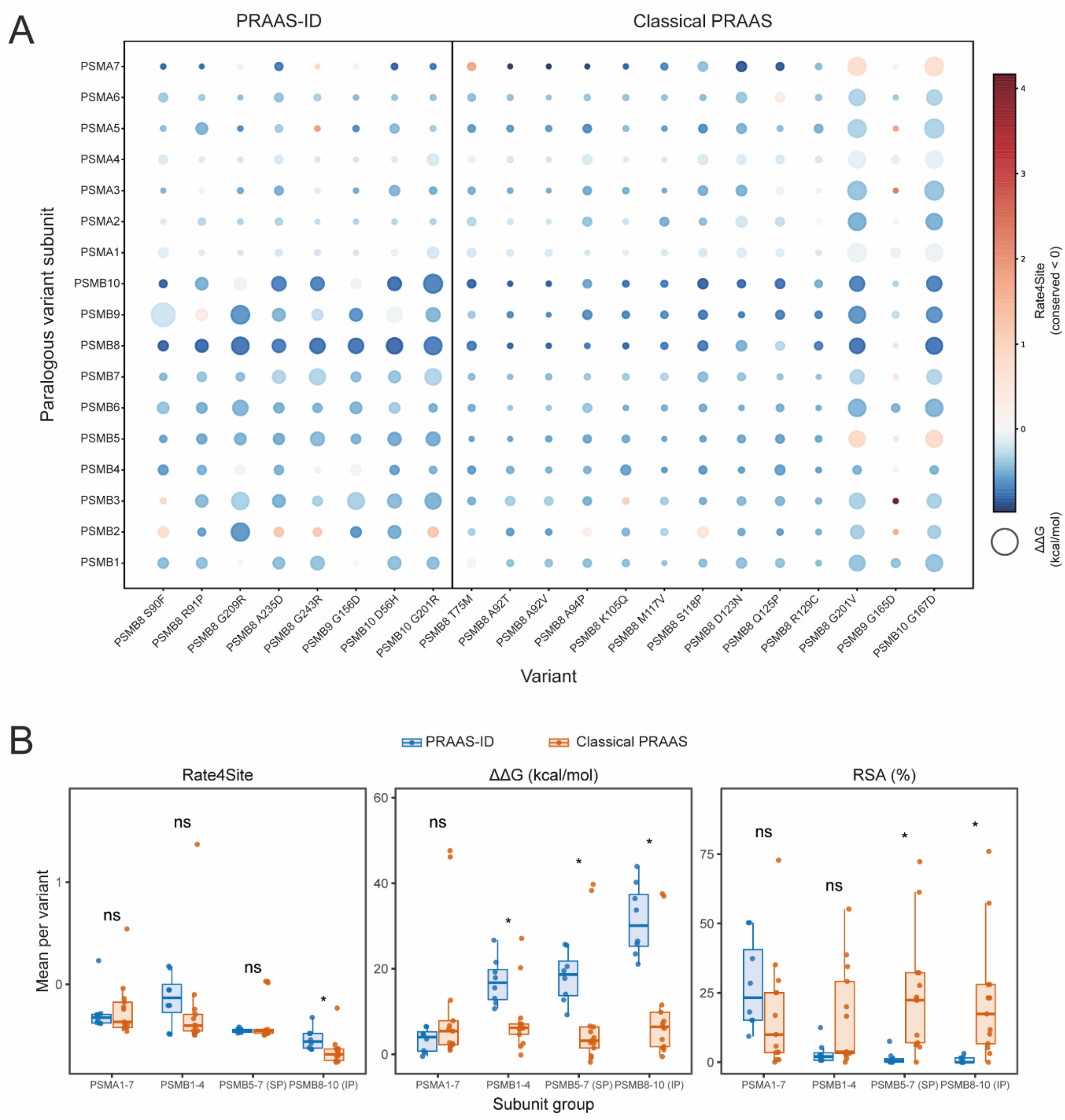

A: Bubble plot of variants, classified as PRAAS-ID or classical PRAAS, showing evolutionary conservation and predicted structural impact of the same amino acid substitution at paralogous positions across proteasome subunits. Bubble color indicates the Rate4Site score (lower values reflect greater conservation), and bubble size indicates the predicted FoldX ΔΔG (kcal/mol). **B:** Distribution of Rate4Site, FoldX ΔΔG (kcal/mol), and RSA (%) values per variant, averaged across subunits within each subunit group.

Significance levels are indicated as ns = non-significant, * = p ≤ 0.05, ** = p ≤ 0.01, and *** = p ≤ 0.001

Figure S17. Structural and evolutionary properties of monoallelic variants and at their paralogous positions.

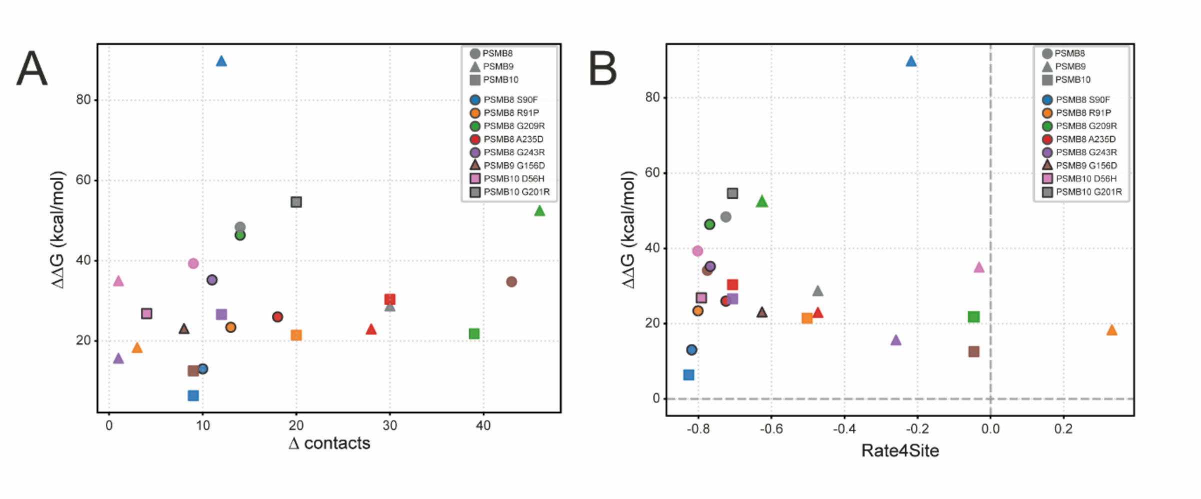

Scatter plots showing predicted structural effects of PRAAS-ID variants and paralogous immunoproteasome β-subunits (*PSMB8*, *PSMB9*, *PSMB10*). Points are colored by variant position. Variants reported in patients are highlighted with a black outline, whereas paralogous variants carrying the same amino acid substitution at equivalent positions lack the outline. **A:** Relationship between predicted destabilization (FoldX ΔΔG) and intermolecular contact perturbation (Δ contacts). **B:** Relationship between predicted destabilization (FoldX ΔΔG) and evolutionary conservation (Rate4Site).

Figure S18. Blue native electrophoresis of fibroblasts and molecular mass calibration

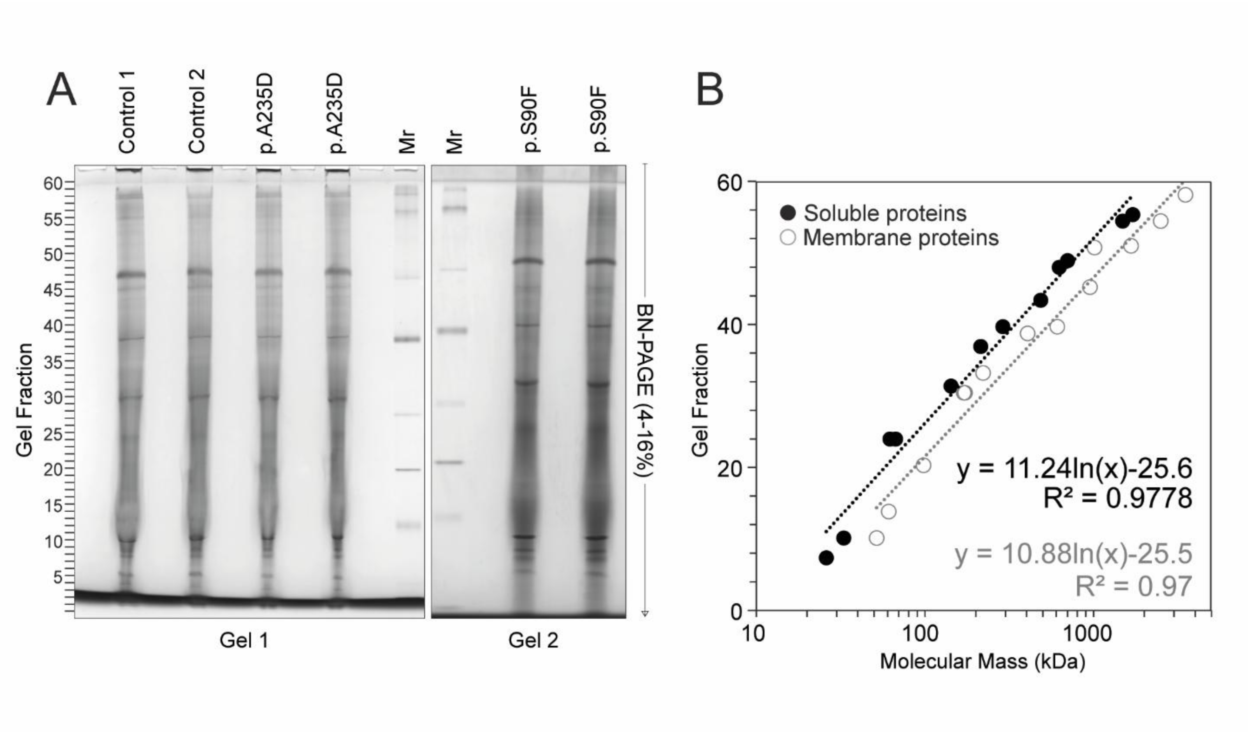

A: Gel images showing protein separation by blue native electrophoresis and Coomassie staining. **B:** Molecular mass calibration using a reference set of soluble and membrane protein complexes with known molecular masses and stoichiometries. The following proteins and complexes were used to calculate the apparent molecular masses of globular (hydrophilic) protein complexes in each gel fraction: monomeric ATP synthase subunit beta (ATP5F1B, 51.7 kDa), tetrameric single-stranded DNA-binding protein (SSBP1, 15.2×4=61 kDa), dimeric citrate synthase (CS, 49×2=98 kDa), complex I (CI) Q-module (NDUFS2, NDUFS3, NDUFS7, NDUFS8, NDUFA5, NDUFAF3, NDUFAF4, 170 kDa), UDP-glucose:glycoprotein glucosyltransferase 1 (UGGT1, 173 kDa), aldehyde dehydrogenase X tetramer (ALDH1B1, 55.3×4=221 kDa), 60 kDa heat shock protein heptamer (HSPD1, 58×7=406 kDa), isocitrate dehydrogenase [NAD] (IDH3A, IDH3B, IDH3G heterooctamer 300 kDa), propionyl-CoA carboxylase (PCCA, PCCB heterododecamer, 832 kDa), TRiC chaperonin (TCP1, CCT2-5, CCT6A/B, CCT7-8 heterohexadecamer, 944 kDa), alpha ketoglutarate dehydrogenase complex (OGDH, DLST, DLD, MRPS36, ca. 3500 kDa). The following transmembrane protein complexes were used as standards for hydrophobic protein complexes: MIC27 monomer (26 kDa), SLC25A5 (33 kDa), VDAC1-2 dimer (62 kDa), TOMM70 (67 kDa), succinate dehydrogense complex (143 kDa), cytochrome *c* oxidase (CIV, 214 kDa), VDAC1-3 nonamers (290 kDa), cytochrome bc_1 complex dimer ($CIII_2$, 485 kDa), ATP synthase (626 kDa), supercomplex $CIII_2$-CIV (700 kDa), supercomplex CI-$CIII_2$ (1485 kDa), respirasome CI-$CIII_2$-CIV (1708 kDa).

Supplemental Tables

Table S1. Variant curation for *PSMB8*, *PSMB9* and *PSMB10*

Gene	gDNA	cDNA	Protein	ClinVar[a]	PMID	Phenotype
PSMB8	chr6:g.32843013G>A	NM_148919.4:c.224C>T	p.(Thr75Met)	Pathogenic 2* (Variation ID: 659832)	21129723; 21953331; 26524591; 28895430; 29115062; 31874111; 37600812	Classical PRAAS
PSMB8	chr6:g.32842968G>A	NM_148919.4:c.269C>T	p.(Ser90Phe)	VUS 2* (Variation ID: 1308452)	This study	PRAAS-ID
PSMB8	chr6:g.32842965C>G	NM_148919.4:c.272G>C	p.(Arg91Pro)	VUS 1* (Variation ID: 2581896)	This study	PRAAS-ID
PSMB8	chr6:g.32842963C>T	NM_148919.4:c.274G>A	p.(Ala92Thr)	-	26524591; 28895430	Classical PRAAS
PSMB8	chr6:g.32842962G>A	NM_148919.4:c.275C>T	p.(Ala92Val)	-	31046790	Classical PRAAS
PSMB8	chr6:g.32842957C>G	NM_148919.4:c.280G>C	p.(Ala94Pro)	-	26567544	Classical PRAAS
PSMB8	chr6:g.32842766T>G	NM_148919.4:c.313A>C	p.(Lys105Gln)	Pathogenic 0* (Variation ID: 548954)	26524591; 31046790	Classical PRAAS
PSMB8	chr6:g.32842730T>C	NM_148919.4:c.349A>G	p.(Met117Val)	-	24001180; 26524591	Classical PRAAS
PSMB8	chr6:g.32842727A>G	NM_148919.4:c.352T>C	p.(Ser118Pro)	Pathogenic 0* (Variation ID: 3220941)	31874111; 33512037; 37600812	Classical PRAAS
PSMB8	chr6:g.32842712C>T	NM_148919.4:c.367G>A	p.(Asp123Asn)	VUS 1* (Variation ID: 870500)	32513120	Classical PRAAS
PSMB8	chr6:g.32842705T>G	NM_148919.4:c.374A>C	p.(Gln125Pro)	-	30387862	Classical PRAAS
PSMB8	chr6:g.32842706G>A	NM_148919.4:c.385C>T	p.(Arg129Cys)	VUS 2* (Variation ID: 870499)	32513120	Classical PRAAS
PSMB8	chr6:g.32841671C>A	NM_148919.4:c.602G>T	p.(Gly201Val)	Pathogenic 0* (Variation ID: 29860)	21852578; 21881205; 23942189; 23942189; 26524591; 36211342	Classical PRAAS
PSMB8	chr6:g.32841648C>G	NM_148919.4:c.625G>C	p.(Gly209Arg)	VUS 2* (Variation ID: 1034913)	This study; 41253591	PRAAS-ID
PSMB8	chr6:g.32841569G>T	NM_148919.4:c.704C>A	p.(Ala235Asp)	-	This study	PRAAS-ID
PSMB8	chr6:g.32841546C>T	NM_148919.4:c.727G>A	p.(Gly243Arg)	VUS 1* (Variation ID: 1431256)	This study	PRAAS-ID
PSMB9	chr6:g.32858440G>A	NM_002800.5:c.467G>A	p.(Gly156Asp)	Pathogenic 0* (Variation ID: 1299361)	34819510	PRAAS-ID
PSMB9	chr6:g.32858467G>A	NM_002800.5:c.494G>A	p.(Gly165Asp)	Conflicting classifications of pathogenicity 1* (Variation ID: 548995)	26524591	Classical PRAAS
PSMB10	chr16:g.67936291C>G	NM_002801.4:c.166G>C	p.(Asp56His)	Pathogenic 0* (Variation ID: 3241970)	38503300	PRAAS-ID
PSMB10	chr16:g.67935478C>T	NM_002801.4:c.500G>A	p.(Gly167Asp)	Pathogenic 0* (Variation ID: 3241965)	37600812	Classical PRAAS
PSMB10	chr16g.67934906C>G	NM_002801.4:c.601G>C	p.(Gly201Arg)	Pathogenic 0* (Variation ID: 3241969)	38503300	PRAAS-ID

a: ClinVar: assessed on 24 October 2025

Table S2. Site-directed mutagenesis oligonucleotides

	Name	Sequence	Length bp	Tm °C
1	1PSMB8_c.269C>T_S90F_for	GCA GTG GAT TTT CGG GCC TCA G	22	67.2
	1PSMB8_c.269C>T_S90F-rev	CTG AGG CCC GAA AAT CCA CTG C	22	67.2
2	2PSMB8_c.272G>C_R91P-for	GTG GAT TCT CCG GCC TCA GCT G	22	68.2
	2PSMB8_c.272G>C_R91P-rev	CAG CTG AGG CCG GAG AAT CCA C	22	68.2
3	3PSMB8_c.625G>A_G209R-for	CTT ATG CCT ACA GGG TCA TGG AC	23	64
	3PSMB8_c.625G>A_G209R-rev	GTC CAT GAC CCT GTA GGC ATA AG	23	64
4	4PSMB8_c.704C>A_A235D-for	CTA TTG CTT ATG ACA CTC ACA GAG AC	26	62.9
	4PSMB8_c.704C>A_A235D-rev	GTC TCT GTG AGT GTC ATA AGC AAT AG	26	62.9
5	5PSMB8_c.727G>A_G243R-for	GAC AGC TAT TCT AGA GGC GTT GTC	24	64.2
	5PSMB8_c.727G>A_G243R_rev	GAC AAC GCC TCT AGA ATA GCT GTC	24	64.2
6	6PSMB8_c.224C>T_T75M-for	CATGGCACCACCATGCTCGCCTTCAAG	27	69.5
	6PSMB8_c.224C>T_T75M-rev	CTTGAAGGCGAGCATGGTGGTGCCATG	27	69.5
7	7PSMB8_c.274G>A_A92T-for	GTGGATTCTCGG**A**CCTCAGCTG	22	64
	7PSMB8_c.274G>A_A92T-rev	CAGCTGAGG**T**CCGAGAATCCAC	22	64
8	8PSMB8_c.275C>T_A92V-for	GTGGATTCTCGGG**T**CTCAGCTG	22	64
	8PSMB8_c.275C>T_A92V-rev	CAGCTGAG**A**CCCGAGAATCCAC	22	64
9	9PSMB8_c.280G>C_A94P-for	GATTCTCGGGCCTCA**C**CTGGGTCC	24	69.6
	9PSMB8_c.280G>C_A94P-rev	GGACCCAG**G**TGAGGCCCGAGAATC	24	69.6

Table S3. Quantitative reverse transcription PCR primer sequences

Target	Alternative names	Forward primer	Reverse primer
SDHA		CTGTCTTCATACGCTTCTGCACTC	CCAGCCACTAGGTGCCAATC
HSPA5	BiP, GRP78	TGTTCAACCAATTATCAGCAAACTC	TTCTGCTGTATCCTCTTCACCAGT
sXBP1	Spliced XBP1	CTGAGTCCGAATCAGGTGCAG	ATCCATGGGGAGATGTTCTGG
ATF4		GTTCTCCAGCGACAAGGCTA	ATCCTGCTTGCTGTTGTTGG
DDIT3	CHOP	AGAACCAGGAAACGGAAACAGA	TCTCCTTCATGCGCTGCTTT
HSP90B1	GRP94	GAAACGGATGCCTGGTGG	GCCCCTTCTTCCTGGGTC

Table S4. Clinical characteristics of included individuals

In separate file

Table S5. Laboratory findings of included individuals

In separate file

Table S6. Quantification of soluble serum factors in individual 5 and 7.

Analyte	Individual 5				Individual 7	
	Detection range (pg/mL)	Healthy controls median (pg/mL)	Healthy controls range (pg/mL)	Value (pg/mL)	Reference range (pg/mL)	Value (pg/ml)
BCA1 (CXCL13)	0.64-7236	12.9	5.37-20.8	**81.6**	–	–
MIP-4 (CCL18)	1.58-29290	5751	2820-13276	**>29290**	–	–
GM-CSF	1.20-21428	3.39	<1.20-59.9	2.46	–	–
IFNγ	1.98-43870	38.7	2.20-152	**252**	0-0.2	**12.2**
IL-1β	1.88-25072	<1.88	<1.88-25.6	<1.88	0-5.7	**8.6**
IL-2	0.57-9931	<0.57	<0.57-61.0	<0.57	–	–
sIL-2Rα	31.9-499286	285	141-671	**3256**	–	–
IL-4	0.70-10000	27.3	21.2-83.1	24.9	–	–
IL-5	0.64-9961	1.52	<0.64-20.1	1.52	0-0.3	**4**
IL-6	0.67-9988	<0.67	<0.67-8.93	**20.8**	0.5-2.2	**15.7**
IL-7	0.64-10249	2.85	<0.64-11.2	4.18	–	–
IL-8	0.70-9298	5.38	1.68-10.2	**51.2**	–	–
sVEGFR-2	156-2526362	6010	4763-7791	**10012**	–	–
sVCAM-1	7.72-124288	31252	22808-51553	**>124288**	–	–
IL-17A	3.44-10010	<3.44	<3.44	<3.44	–	–
IL-21	60.8-999925	154	<60.8-12874	182	–	–
sTNFR-2	16.0-216001	2618	1488-4492	**7245**	–	–
IL-18	0.59-10027	19.3	8.94-46.0	**1214**	–	–
VEGF-D	45.4-25000	235	47.0-343	82.8	–	–
IP-10 (CXCL10)	1.26-3953	29.9	13.8-63.4	**752**	0-104	**2190**
MIP-3 (CCL23)	0.30-4685	35.0	16.0-80.0	**>4685**	–	–
MIG (CXCL9)	1.62-19669	66.1	22.2-151	**897**	23-220	**3839**
RANTES (CCL5)	0.53-9707	1917	843-4136	933	–	–
TNFα	3.21-48196	13.3	5.58-33.2	24.5	0.2-5.6	5
PF4 (CXCL4)	1480-1057120	9615	2388-17091	5279	–	–
Endostatin	1.00-4997	103	85-159	100	–	–
TIMP-1	239-181547	94292	76761-148602	90945	–	–
Eotaxin	–	–	–	–	55-220	50.5

Substantial differences of soluble factors in the serum are presented as >1.5x upper limit of normal and highlighted in bold.

[a]Serum of healthy controls was used as reference (n=13). Quantification is expressed as mean value (pg/mL) and range of the lower and upper limit of normal (min-max).

Table S7. Candidate gene variants and rare variants in proteasome subunits

Family	Individual	Gene	HGVSg (hg38)	HGVSc	HGVSp	Zygosity	Segregation	CADD v1.6	AF GnomAD v4.0.0	Comment
Family 1	Individual 1	*PSMB8*	chr6:g.32842968G>T	NM_148919.4:c.269C>T	p.(Ser90Phe)	Heterozygous	*De novo*	28.2	Absent	
		IL25	chr14:g.23375644C>T	NM_022789.3:c.298C>T	p.(Arg100Trp)	Homozygous	PV MV	30	0.00056322 (1x homozygote)	
		GMPPB	chr3:g.49722056C>T	NM_013334.3:c.860G>A	p.(Arg287Gln)	Heterozygous	PV	20.9	0.00017912 (0x homozygote)	No second variant on other allele
		HS6ST2	chrX:g.132958353C>G	NM_001077188.1:c.250G>C	p.(Ala84Pro)	Hemizygous	MV	21.7	Absent	Does not fit phenotype
Family 2	Individual 2 and 3	*PSMB8*	chr6:g.32842965C>G	NM_148919.4:c.272G>C	p.(Arg91Pro)	Heterozygous	MV (mosaic)	32	Absent	
Family 3	Individual 4	*PSMB8*	chr6:g.32841648G>A	NM_148919.4:c.625G>A	p.(Gly209Arg)	Heterozygous	*De novo*	29.1	Absent	
Family 4	Individual 5	*PSMB8*	chr6:g.32841569G>T	NM_148919.4:c.704C>A	p.(Ala235Asp)	Heterozygous	*De novo*	29.7	Absent	
		CLPB	chr11:g.72372971C>T	NM_030813.6:c.690G>A	p.(Trp230*)	Heterozygous	PV	38	0.00001115 (0x homozygote)	No second variant on other allele
Family 5	Individual 6	*PSMB8*	chr6:g.32841546C>T	NM_148919.2:c.727G>A	p.(Gly243Arg)	Heterozygous	*De novo*	31	Absent	
		PAPPA	chr9:g.116187628G>C	NM_002581.5:c.890G>C	p.(Trp297Ser)	Heterozygous	*De novo*	29.2	0.0000006195	Present in unaffected child
	Individual 7	*PSMB8*	chr6:g.32841546C>T	NM_148919.2:c.727G>A	p.(Gly243Arg)	Heterozygous	MV	31	Absent	
		PAPPA	chr9:g.116187628G>C	NM_002581.5:c.890G>C	p.(Trp297Ser)	Heterozygous	MV	29.2	0.0000006195	Present in unaffected sibling

LP: likely pathogenic; MV: maternal variant; P: pathogenic; PV: paternal variant

Table S8. Frustration index and proteasomal contacts across wild-type and mutant variants

In separate file

Table S9. List of significantly upregulated or downregulated proteins in the p.Ala235Asp cell line

In separate file

Table S10. Enriched Gene Ontology (GO) terms and Reactome pathways in upregulated and downregulated protein sets

In separate file

Table S11. Predicted structural and biophysical effects of paralogous variants in immunoproteasome β-subunits (*PSMB8*, *PSMB9* and *PSMB10*).

Gene	Variant	Described pathogenic	gnomAD v4.1.0	RSA	Rate4Site	ΔΔG	Δ contacts	FI switch
PSMB8	**p.Asp89His**	No	Absent	0	-0.802	39.3	9	Neutral
PSMB9	**p.Asp37His**	No	Absent	0	-0.032	35.0	1	Neutral
PSMB10	**p.Asp56His**	Yes	Absent	0	-0.792	26.8	4	High to neutral
PSMB8	**p.Ser90Phe**	Yes	Absent	0.4	-0.818	13.1	10	Neutral to minimal
PSMB9	**p.Ser38Phe**	No	Absent	0	-0.217	89.8	12	Neutral to minimal
PSMB10	**p.Thr57Phe**	No	Absent	4.3	-0.826	6.4	9	Neutral to minimal
PSMB8	**p.Arg91Pro**	Yes	Absent	1.3	-0.801	23.4	13	Neutral to high
PSMB9	**p.Arg39Pro**	No	Absent	5.9	0.332	18.4	3	Neutral to high
PSMB10	**p.Arg58Pro**	No	Absent	2.3	-0.502	21.4	20	Neutral to high
PSMB8	**p.Gly209Arg**	Yes	Absent	0	-0.769	46.4	14	Neutral
PSMB9	**p.Gly156Arg**	No	Absent	0	-0.626	52.5	46	Neutral
PSMB10	**p.Ala175Arg**	No	Absent	0	-0.046	21.8	39	Neutral to minimal
PSMB8	**p.Gly209Asp**	No	Absent	0	-0.769	34.8	43	Neutral to high
PSMB9	**p.Gly156Asp**	Yes	Absent	0	-0.626	23.1	8	Neutral to high
PSMB10	**p.Ala175Asp**	No	Absent	0	-0.046	12.6	9	Neutral to high
PSMB8	**p.Ala235Asp**	Yes	Absent	0	-0.725	26.0	18	Neutral to high
PSMB9	**p.Ala182Asp**	No	Absent	0	-0.473	23.0	28	Neutral to high
PSMB10	**p.Gly201Asp**	No	Absent	0	-0.706	30.4	30	Neutral to high
PSMB8	**p.Ala235Arg**	No	Absent	0	-0.725	48.4	14	Neutral
PSMB9	**p.Ala182Arg**	No	Absent	0	-0.473	28.7	30	Neutral
PSMB10	**p.Gly201Arg**	Yes	Absent	0	-0.706	54.6	20	Neutral
PSMB8	**p.Gly243Arg**	Yes	Absent	0	-0.767	35.2	11	Neutral
PSMB9	**p.Gly190Arg**	No	74x	5.1	-0.259	15.7	1	Neutral
PSMB10	**p.Gly209Arg**	No	Absent	0	-0.706	26.6	12	Neutral

RSA, relative solvent accessibility; Rate4Site, evolutionary conservation score; ΔΔG, predicted change in protein stability (FoldX); Δ contacts, change in residue interaction network; FI, frustration index; FI switch, change in local frustration state; gnomAD v4.1.0, Genome Aggregation Database.

Table S12. Identified proteasome subunits and associated subcomplexes detected in control samples from THP1, 143B, and fibroblast cell lines

In separate file

Supplemental Methods

Recruitment and ethics approvals

Family 1 was identified in the Undiagnosed Diseases Network (UDN) study, a multicenter study approved by the National Institutes of Health IRB (15HG0130).

Family 2 was enrolled under the Immunology Biorepository #667 protocol for research genomic sequencing and formal case reporting of their medical course which was approved by Seattle Children's Hospital Institutional Review board.

Family 3 was tested clinically through the Victorian Clinical Genetics Services, Melbourne, Victoria.

Family 4 was part of the Radboud Data- and Biobank for genetics and rare diseases and was enrolled in an international research study to diagnose the undiagnosed at the Radboud University Medical Center (Radboudumc) under a protocol approved by the Institutional Review Board of CMO Radboudumc and METC East Nijmegen, the Netherlands (2018-4985 and 2019-5554).

Family 5 was recruited under the whole exome and whole genome sequencing program for undiagnosed diseases of Gaslini Children's Hospital and Italian Institute of Technology under the protocol approved by the Regione Liguria Institutional Review Board.

Cell culturing

Human osteosarcoma 143B cells (cell line GM05887, Coriell Institute for Medical Research) were cultured in Dulbecco's Modified Eagle's Medium (DMEM, Gibco 41-965-039) supplemented with 10% fetal bovine serum (FBS-11A, Capricorn scientific) and 1% antibiotic/antimycotic solution (30-004-CL, Corning) in a humidified, 5% CO2 atmosphere at 37°C.

Human monocytic cell line THP-1 (ATCC® TIB202™) was cultured in RPMI-1640 supplemented with 10% FBS-11A, 100 U/ml penicillin, 100 µg/ml streptomycin, 250 n/ml amphotericin B and 0.05 mM β-mercaptoethanol in a humidified, 5% CO2 atmosphere at 37°C. THP-1 monocytes were differentiated with 10 ng/ml phorbol 12-myristate 13-acetate for 48 h and macrophages were harvested by trypsinization.

Human skin fibroblasts were cultured in DMEM (Gibco) supplemented with 10% FCS (Capricorn scientific) and antibiotic/antimycotic solution in a humidified, 5% CO2 atmosphere at 37 °C. To enhance the expression of immunoproteaseome-specific subunits, cells were stimulated for 48 h with 250 U/ml human recombinant IFNγ (ImmunoTools, Friesoythe, Germany). Cells from four 175-cm2 flasks were harvested by trypsinization, washed with PBS and centrifuged at 1000 *g* for 5 min at 4°C.

Protein extraction and immunoblotting

To maintain the activity of proteasomes, cells were lysed in TSDG buffer (10 mM Tris pH7.5, 10 mM NaCl, 25 mM KCl, 1 mM $MgCl_2$, 0.1 mM EDTA, 10% glycerol, 1 mM DTT, 2mM ATP in H_2O) with seven freeze-thaw cycles consisting of rapid freezing in an ethanol/dry ice bath for 3 minute followed by thawing in a 37 °C water bath for 3 minutes, ensuring complete thawing in each cycle. Afterwards, a centrifugation for 10 minutes with 16000 *g* at 4 °C was performed, and supernatants were used for immunoblotting and proteasome activity assays.

Cell lysates containing 10 µg proteins were denatured in 5x reducing loading buffer (250 mM Tris pH6.8, 0.5 M DTT, 10% SDS, 50% glycerol, 0.25% bromophenol blue in H_2O) for 10 minutes at 95 °C. Afterwards, samples were separated on 4-15% TGX stain-free gels (Bio-Rad, 5678085) in SDS-PAGE running buffer (25 mM Tris, 192 mM glycine, 0.1 % SDS in H_2O, pH8.6). After electrophoresis, proteins were visualized by stain-free staining under UV-light as loading control. Proteins were then transferred on ethanol-activated PVDF membranes with the TransBlot Turbo System. The transfer was carried out at constant 25 V for 30 minutes in transfer buffer (25 mM Tris, 192 mM glycine, 20% ethanol in H_2O, pH8.6). After the transfer, total protein staining was performed by staining membranes in 0.2% Ponceau-S staining solution for 10 minutes. Membranes were then blocked in 3% non-fat dry milk (NFDM)/TBS-T for 1 hour at RT, followed by primary antibody incubation overnight at 4 °C. Antibodies against PSMB5 (Thermo Fisher Scientific PA1-977), PSMB8 (Abcam ab3329), PSMA3 (Santa Cruz Biotechnology Inc. Sc-166205), PSMB9 (Abcam ab184172), Ubiquitin (linkage-specific K48; Abcam 140601), V5 tag (Thermo Fisher Scientific, R960-25) and GAPDH (Santa Cruz Biotechnology, sc-47724) were used. Subsequent incubation with HRP-coupled secondary antibodies (1:10,000) in 3% NFDM/TBS-T was performed at RT for 1 hour. Membranes were then incubated in ECL SuperSignal for 5 minutes at RT, and the signal was developed with an imager (Fusion FX7, Vilber). When reprobing was needed, HRP activity of the former secondary antibody was inhibited by incubating membranes in 0.01 M para-toluene sulfonic acid (PTSA) twice for 5 minutes before the next primary antibody incubation.

In-gel proteasome proteolytic activity assay

The chymotrypsin-like activity of proteasome was assessed with an in-gel fluorescence technique. Native cell lysates with 15 µg protein were mixed with 5x native loading buffer (250 mM BisTris pH6.5, 250 mM NaCl, 50% glycerol, 0.25% bromophenol blue in H_2O). Without heating, the mixture was resolved using a 3-12% Bis-Tris native gel (Thermo Fisher, #BN1003BOX). After 3-hour electrophoresis run at constantly 150-200 V in native running buffer (50 mM BisTris, 50 mM Tricin, 0.4 mM ATP, 2 mM $MgCl_2$, 0.5 mM DTT in H_2O), gels were incubated with proteasome activity assay buffer (20 mM Tris pH7.4, 5 mM $MgCl_2$, 2 mM ATP, 100 µM Ac-ANW-AMC in H_2O) for 20 minutes at

37 °C. The chymotrypsin-like activity of proteasomal complexes is reflected by the cleavage of the Suc-LLVY-AMC substrate and the release of fluorescent free aminomethylcumarin (AMC), which could be measured by an imager (Fusion FX7, Vilber) using excitation at 365 nm and detection at 450 nm. The gel was subsequently blotted on a PVDF membrane, which was further stained with RubyStain total protein stain (Thermo Fisher Scientific, V10309) for loading control. The proteasome α7 subunit (PSMA3) was detected as a measure of proteasome abundance for normalization of proteolytic activity. PSMB5 and PSMB8 were detected as a measure of constitutive and immunoproteasome abundance, respectively.

Active proteasome subunit abundance assay

The amount of active proteasome catalytic subunits was assessed with activity-based probes (ABPs), which are composed of a reactive group, a recognition element, and a reporter tag. ABPs covalently attach to the active sites of proteasomal catalytic subunits, enabling their detection via the reporter. Pan-ABPs (cy5-epoxomicin) targeting all proteolytic β-subunits (kindly provided by Dr. B.I. Florea) were used in this study. Specifically, 10 µg native cell lysates were incubated with 0.5 µM cy5-epoxomicin for 1 hour at 37 °C. All reactions were performed in TSDG buffer. After incubation with ABPs, samples were solubilized with the 5x loading buffer (250 mM Tris pH6.8, 0.5 M DTT, 10% SDS, 50% glycerol, 0.25% bromophenol blue in H_2O) for 10 minutes at 70 °C. Samples were subsequently resolved by SDS-PAGE on 12.5% tris-glycine gels with 3.3% crosslinker (acrylamide:bisacrylamide 29:1). Electrophoresis was carried out at 120-150 V until the 17 kDa band of the protein ladder reached the gel bottom to get the optimal separation of proteasome catalytic subunits. After separation, fluorescence detection was performed at at Ex/Em = 650/670 nm for cy5-epoxomicin using the imager (Fusion FX7, Vilber) and was followed by subsequent blotting of the gel to determine total expression of proteasome subunits of interest for normalization.

Complexome profiling

Cell fractionation

Fibroblast, 143B and THP-1 cell pellets were resuspended in 4 ml ice-cold homogenization buffer (250 mM sucrose, 1 mM EDTA, 20 mM Tris/HCl, pH 7.4) and disrupted mechanically by 15 strokes, passing the cell suspension through a 20 G needle fixed to a 5-ml syringe on ice.

Fibroblasts cell homogenates were centrifuged at 1000 *g* for 10 min at 4°C and the supernatants were centrifuged at 21000 *g* for 10 min at 4°C. Pellets were resuspended in homogenization buffer and the protein concentration was determined by Lowry. Suspension aliquots containing 200 µg protein were centrifuged at 21000 *g* for 20 min at 4°C. Supernatants were discarded and the pellets were shock frozen in liquid nitrogen and stored at -80°C.

143B cell homogenates were centrifuged at 1000 *g* for 10 min at 4°C and the supernatant was centrifuged at 6000 *g* for 10 min at 4°C. Pellets were resuspended in homogenization buffer and the protein concentration was determined by Lowry.

THP-1 cell homogenates were centrifuged at 1000 *g* for 10 min at 4°C and the protein concentration was determined from the supernatants.

<u>Blue-Native PAGE and cutting</u>

Samples containing 200 µg of protein were thawed and resuspended at 10 mg protein/ml in solubilization buffer (0.5 M 6-aminohexanoic acid, 1 mM EDTA, 50 mM imidazole/HCl, pH 7.0) and solubilized with 6 g digitonin/g protein. Protein solutions were centrifuged at 22000 *g* for 20 min at 4°C and protein concentration of the supernatant was determined by Lowry. An aliquot containing 0.1 mg protein was mixed with 5% Coomassie blue (Serva Blue G) in 0.5 M aminohexanoic acid and loaded on a 4-16% polyacrylamide gradient gel. Proteins were separated by gel electrophoresis at 4°C as described previously.[4] After electrophoresis, gels were fixed in 50% methanol, 10% acetic acid, 10 mM ammonium acetate and stained with Coomassie blue (Figure S18A). Gels were destained in 10% acetic acid, washed with water, documented and each lane was cut into 60 even pieces and transferred to a 96-well MultiScreen-BV. 1.2 µm filter plate (Millipore).

<u>In-gel digestion</u>

Gel pieces were destained for 30 min in 50% methanol, 50 mM ammonium bicarbonate (ABC) at room temperature and centrifuged at 600 *g* for 2 min. This step was repeated 3-4 times until the Coomassie blue dye was removed entirely. Then, gel pieces were incubated for 1 h in 5 mM dithiothreitol, 50 mM ABC followed by 45 min incubation with 15 mM 2-chloroacetamide, 50 mM ABC. After washing once with 50% methanol, 50 mM ABC, gel pieces were let dry at room temperature for 30 min and rehydrated with 20 µl of 5 µg trypsin/ml in 50 mM ABC for 30 min at 4°C. After adding 50 µl 50 mM ABC, plates were sealed and incubated overnight at 37°C. Peptide solutions were transferred to a PCR plate by centrifugation at 600 *g* for 2 min. The gel pieces were washed once with 50% acetonitrile, 5% formic acid and the filtrates were collected into the same PCR plate. Peptide solutions were dried for 2.5 h at 45°C in a centrifuge concentrator (Concentrator plus, Eppendorf). Peptides were dissolved in 20 µl 0.1% formic acid.

<u>LC-MS/MS</u>

From fibroblasts samples, 2 µl were injected and subjected to liquid chromatography tandem mass spectrometry (LC-MS/MS) on a quadrupole-orbitrap hybrid orbitrap mass spectrometer (Exploris 480, Thermo Fisher Scientific) coupled at the front end to an ultra-high pressure liquid

chromatography system (Vanquish neo UHPLC System, Thermo Fisher). Attached to the UHPLC was a peptide trap (100 µm x 20 mm, 100 Å pore size, 5 µm particle size, C18, Nano Viper, Thermo Fisher) for online desalting and purification, followed by a 25 cm C18 reversed-phase column (75 µm x 250 mm, 130 Å pore size, 1.7 µm particle size, peptide BEH C18, nanoEase, Waters). Peptides were separated using a 35 min method with linearly increasing ACN concentration from 2% to 30% ACN over 25 minutes.

MS/MS measurements from fibroblasts samples were performed on a quadrupole-orbitrap hybrid mass spectrometer (Exploris 480, Thermo Fisher Scientific). Eluting peptides were ionized using a nano-electrospray ionization source (nano-ESI) with a spray voltage of 1,800 V and analyzed in data-independent acquisition (DIA) mode. For each MS1 scan, ions were accumulated for a maximum of 240 ms or until a charge density of 3 x 10^6 ions (AGC Target) was reached. Fourier-transformation based mass analysis of the data from the orbitrap mass analyzer was performed covering a mass range of m/z 400 – 1,400 with a resolution of 120,000 at m/z 200. Within a precursor mass range of m/z 380-980 fragmentation in DIA-mode with m/z 12 isolation windows and m/z 1 window overlaps was performed. Fragmentation was performed at normalized collision energy of 28% using higher energy collisional dissociation (HCD). An AGC target of 2 x 10^6 ions or a maximum of 54 ms was set. Orbitrap resolution was set to 30,000 with a scan range from m/z 350-2000.

From 143B and THP-1 samples, 5 µl of the peptide solutions were analyzed by liquid chromatography electrospray ionization tandem mass spectrometry (LC-ESI-MS/MS) in a Q-Exactive mass spectrometer (Thermo Fisher Scientific) equipped with an Easy nLC1000 nano-flow high-performance liquid chromatography system at the front end. Peptide separation was performed with an emitter column (15cm L x 100 µm ID x 360 µm OD x 15 µm orifice; MSWil, CoAnn Technologies, LLC) filled with ReproSil-Pur C18-AQ reverse phase beads (3 µm particle size, 120 Å pore size; Dr. Maisch GmbH) using a 30 min linear gradient of 5 to 35% acetonitrile with 0.1% formic acid. The mass spectrometer operated in positive ion switching automatically between MS and data dependent MS/MS, fragmenting the twenty most intense ions per precursor scan. Full scan MS mode (400 to 1400 m/z) was operated with automatic gain control target of 1 × 10^6 ions, 70000 resolution and a maximum ion transfer of 20 ms. Selected ions for MS/MS were analyzed using the following parameters: resolution 17,500; AGC target of 1 × 10^5; maximum ion transfer of 50 ms; 4.0 m/z isolation window and dynamic exclusion of 30.0 s was used.

Proteomics data analysis

LC-MS/MS data from fibroblasts were searched with the CHIMERYS DIA algorithm integrated into the Proteome Discoverer software (v3.1.0.638, Thermo Fisher Scientific) against a reviewed human

Swissprot database (obtained November 2023) using Inferys 3.0 fragmentation as prediction model. Carbamidomethylation was set as a fixed modification for cysteine residues. The oxidation of methionine was allowed as a variable modification. A maximum number of one missing tryptic cleavage was set. Peptides between 7 and 30 amino acids were considered. A strict cutoff (FDR < 0.01) was set for peptide identification. Quantification was performed by CHIMERYS based on fragment ions. The mass spectrometry proteomics data have been deposited to the ProteomeXchange Consortium via the PRIDE partner repository with the dataset identifier PXD064505.[5]

A new database search was performed to identify the PSMB8 variant A235D and S90F. To this end, the raw data were searched with the DIA-NN algorithm (Version 1.9.1) against a human database (obtained November 2023) which included the PSMB8 variants.[6] The oxidation of methionine, the N-terminal methionine excision, and the acetylation of the protein N-terminus were allowed as variable modifications. A maximum number of two missing tryptic cleavages was set. Peptides between 7 and 30 amino acids were considered. A strict cutoff (FDR < 0.01) was set for peptide identification in double pass mode. Match between runs was activated.

LC-MS/MS raw files from 143B and THP-1 samples were analyzed using MaxQuant 1.5.0.25, and 1.6.17.0, respectively. 143B spectra were matched against the human NCBI Reference Sequence Database release 55 with reverse decoy and a false discovery rate of 0.01. THP-1 spectra were marched against the Uniprot database of canonical isoforms downloaded on May 2021.

Protein groups text files containing the abundance values of each protein across all gel fractions were subjected to hierarchical clustering analysis by uncentered Pearson correlation with average linkage using Cluster 3.0.[7] Protein migration profiles were visualized using NOVA v0.5.7 and the differences of the protein migration profiles between controls and patient fibroblasts were scored by Hausdorff distance calculations using COPAL as described previously.[8,9] Protein migration profiles of control and patient fibroblasts were uploaded to the complexome profiling data resource, CEDAR, accession number (CRX49).[10] A set of soluble and membrane protein complexes with established molecular mass and stoichiometry were used as standards to estimate the molecular masses of globular (hydrophilic) and transmembrane (hydrophobic) protein complexes in each gel fraction, respectively (Figure S18B).

Genome sequencing and variant analysis

Family 1

Family 1 underwent trio genome sequencing through the UDN Sequencing Core at Baylor Genetics as previously described, and research reanalysis was performed at the BCM UDN site.[11] The research genome reanalyzes prioritized rare, *de novo* and biallelic variants that had an allele frequency of < 1% in gnomAD v4.0.0 and in the BCM UDN internal sequencing database (> 1200 samples). Codified Genomics was used for variant filtering and prioritization.

Family 2

The exome sequencing was clinically performed using enrichment capture kit from IDT xGen Exome v1 and v2 from extracted genomic DNA isolated from frozen brain tissue for individual 4 and buccal swabs for the unaffected parents. The enriched targets were simultaneously sequenced with paired-end reads on an Illumina platform. Bi-directional sequence reads were assembled and aligned to reference sequences based on NCBI RefSeq transcripts and human genome build GRCh37/UCSC hg19. Initial analysis was performed using custom-developed proprietary analysis tool GeneDx's XomeAnalyzer (a variant annotation, filtering, and viewing interface for WES data). Further analysis of the proteosome genes were also performed manually using the Integrative Genomics Viewer (IGV).

Family 3

Clinically accredited trio genome sequencing was performed at the Victorian Clinical Genetics Services, Melbourne, Australia as previously described and identified a *de novo* heterozygous missense variant in *PSMB8*, NM_148919.4(PSMB8): c.625G>A; p.(Gly209Arg).[12]

Family 4

Genome sequencing for the proband was outsourced to the Beijing Genomics Institute (BGI) and performed on a BGISeq500 platform. Paired-end 100 bp reads were generated, achieving a median coverage of 30-fold. Data processing was carried out at the Radboud University Medical Center. Reads were aligned to the GRCh38 reference genome using Bwa-mem2 v2.2.1, and quality control was performed with Qualimap v2.2.1. Single nucleotide variants (SNVs) were called using GATK HaplotypeCaller v3.8. Structural variants (SVs) were detected using Manta v1.1.0 (Illumina), and copy number variants (CNVs) were identified with Canvas v1.40.0 (Illumina). Short tandem repeats (STRs) were analyzed using ExpansionHunter v3.1.2 with default parameters. All variant types (SNVs, SVs, CNVs) were annotated using an in-house pipeline. Rare candidate SNVs were prioritized based on a gnomAD v3.1 and internal frequency of <1%. CNVs and SVs were filtered using a <1% frequency

threshold in the 1000 Genomes database and an internal reference set, requiring a minimum reciprocal overlap of 90%. Additional annotations, including CADD scores, SpliceAI, phyloP, and AlphaMissense, along with the patient's phenotype, were used to prioritize potentially disease-causing variants. Candidate variants were segregated in parental DNA with Sanger sequencing, allowing *de novo* assessment.

Family 5

Genome sequencing was generated on an Illumina NovaSeq 6000 at the Genomics Facility of the Istituto Italiano di Tecnologia (Genoa, Italy) and analyzed by the Clinical Bioinformatics Unit, Istituto Giannina Gaslini (Genoa, Italy). We first interrogated the coding regions of all known autoinflammatory disease (AID) genes (~60, IUIS 2024). No pathogenic variants were identified. Variant filtering used standard criteria: high quality and depth, rarity in population databases (e.g., gnomAD <1%), predicted protein-altering or canonical splice-site effect, inheritance consistency, and exclusion of known benign/common variants.

Interferon (IFN) type I signature

Family 1

The IFN score calculation was adapted with the following modifications.[13] RNA was extracted from cryopreserved peripheral blood mononuclear cells (PBMCs) using the Quick RNA Miniprep Kit (Zymo Research) per manufacturer's instructions, and 33ng converted to cDNA using the GoScript Reverse Transcription System (Promega). Real time PCR was performed using TaqMan Fast Advanced Master Mix and commercially available primer/probe sets (both Applied Biosystems) on a Roche LightCycler 96. The relative abundance of each target transcript was normalized to GAPDH, and data are expressed relative to a single control patient.

Family 3

An IFN-score was calculated based on six IFN-related genes: IF127, IF144L, IFIT1, ISG15, RSAD2, SIGLEC1.

Family 4

Based on the expression levels quantified from PAXgene Blood RNA tubes by RT-PCR of five IFN-related genes (IFI44, IFI44L, IFIT1, IFIT3, and MX1) and a reference gene (ABL) an interferon-type 1 (IFN-1) gene signature score was calculated for each sample, as previously described.[14,15] A value ≥ 9.4 was considered positive.

Family 5

IFN induced gene expression analysis was performed as described by Tesser et al.[16]

Cytokine measurements

Family 4

For cytokine, chemokine and soluble receptor production measurements, serum was collected. Concentrations (pg/mL) of human BCA-1, MIP-3, MIP-4, GM-CSF, IFNγ, IL-1β, IL-2, sIL-2Rα, IL-4, IL-5, IL-6, IL-7, IL-8, sVEGFR-2, sVCAM-1, IL-17A, IL-21, sTNFR2, IL-18, VEGF-D, IP-10, MIG, RANTES, TNFα, PF4, Endostatin, and TIMP-1 were measured according to the manufacturer's instructions. For all factors, measured values below the lower limit of detection are represented by this lowest detection value. Serum analysis was performed using MILLIPLEX® Multiplex Assays (Merck Millipore) using a Flexmap 3D system.

Supplemental References

1. Dong, Y., Zhang, S., Wu, Z., Li, X., Wang, W.L., Zhu, Y., Stoilova-McPhie, S., Lu, Y., Finley, D., and Mao, Y. (2019). Cryo-EM structures and dynamics of substrate-engaged human 26S proteasome. Nature *565*, 49-55. 10.1038/s41586-018-0736-4.
2. Ladi, E., Everett, C., Stivala, C.E., Daniels, B.E., Durk, M.R., Harris, S.F., Huestis, M.P., Purkey, H.E., Staben, S.T., Augustin, M., et al. (2019). Design and Evaluation of Highly Selective Human Immunoproteasome Inhibitors Reveal a Compensatory Process That Preserves Immune Cell Viability. J Med Chem *62*, 7032-7041. 10.1021/acs.jmedchem.9b00509.
3. Huang, X., Luan, B., Wu, J., and Shi, Y. (2016). An atomic structure of the human 26S proteasome. Nat Struct Mol Biol *23*, 778-785. 10.1038/nsmb.3273.
4. Wittig, I., Braun, H.P., and Schagger, H. (2006). Blue native PAGE. Nat Protoc *1*, 418-428. 10.1038/nprot.2006.62.
5. Perez-Riverol, Y., Bandla, C., Kundu, D.J., Kamatchinathan, S., Bai, J., Hewapathirana, S., John, N.S., Prakash, A., Walzer, M., Wang, S., and Vizcaino, J.A. (2025). The PRIDE database at 20 years: 2025 update. Nucleic Acids Res *53*, D543-D553. 10.1093/nar/gkae1011.
6. Demichev, V., Messner, C.B., Vernardis, S.I., Lilley, K.S., and Ralser, M. (2020). DIA-NN: neural networks and interference correction enable deep proteome coverage in high throughput. Nat Methods *17*, 41-44. 10.1038/s41592-019-0638-x.
7. de Hoon, M.J., Imoto, S., Nolan, J., and Miyano, S. (2004). Open source clustering software. Bioinformatics *20*, 1453-1454. 10.1093/bioinformatics/bth078.
8. Giese, K.P., Aziz, W., Kraev, I., and Stewart, M.G. (2015). Generation of multi-innervated dendritic spines as a novel mechanism of long-term memory formation. Neurobiol Learn Mem *124*, 48-51. 10.1016/j.nlm.2015.04.009.
9. Van Strien, J., Guerrero-Castillo, S., Chatzispyrou, I.A., Houtkooper, R.H., Brandt, U., and Huynen, M.A. (2019). COmplexome Profiling ALignment (COPAL) reveals remodeling of mitochondrial protein complexes in Barth syndrome. Bioinformatics *35*, 3083-3091. 10.1093/bioinformatics/btz025.
10. van Strien, J., Haupt, A., Schulte, U., Braun, H.P., Cabrera-Orefice, A., Choudhary, J.S., Evers, F., Fernandez-Vizarra, E., Guerrero-Castillo, S., Kooij, T.W.A., et al. (2021). CEDAR, an online resource for the reporting and exploration of complexome profiling data. Biochim Biophys Acta Bioenerg *1862*, 148411. 10.1016/j.bbabio.2021.148411.
11. Keehan, L., Jiang, M.M., Li, X., Marom, R., Dai, H., Murdock, D., Liu, P., Hunter, J.V., Heaney, J.D., Robak, L., et al. (2021). A novel de novo intronic variant in ITPR1 causes Gillespie syndrome. Am J Med Genet A *185*, 2315-2324. 10.1002/ajmg.a.62232.
12. Lunke, S., Bouffler, S.E., Patel, C.V., Sandaradura, S.A., Wilson, M., Pinner, J., Hunter, M.F., Barnett, C.P., Wallis, M., Kamien, B., et al. (2023). Integrated multi-omics for rapid rare disease diagnosis on a national scale. Nat Med *29*, 1681-1691. 10.1038/s41591-023-02401-9.
13. Rice, G.I., Forte, G.M., Szynkiewicz, M., Chase, D.S., Aeby, A., Abdel-Hamid, M.S., Ackroyd, S., Allcock, R., Bailey, K.M., Balottin, U., et al. (2013). Assessment of interferon-related biomarkers in Aicardi-Goutieres syndrome associated with mutations in TREX1, RNASEH2A, RNASEH2B, RNASEH2C, SAMHD1, and ADAR: a case-control study. Lancet Neurol *12*, 1159-1169. 10.1016/S1474-4422(13)70258-8.
14. Huijser, E., Bodewes, I.L.A., Lourens, M.S., van Helden-Meeuwsen, C.G., van den Bosch, T.P.P., Grashof, D.G.B., van de Werken, H.J.G., Lopes, A.P., van Roon, J.A.G., van Daele, P.L.A., et al. (2022). Hyperresponsive cytosolic DNA-sensing pathway in monocytes from primary Sjogren's syndrome. Rheumatology (Oxford) *61*, 3491-3496. 10.1093/rheumatology/keac016.
15. Bodewes, I.L.A., Al-Ali, S., van Helden-Meeuwsen, C.G., Maria, N.I., Tarn, J., Lendrem, D.W., Schreurs, M.W.J., Steenwijk, E.C., van Daele, P.L.A., Both, T., et al. (2018). Systemic interferon

type I and type II signatures in primary Sjogren's syndrome reveal differences in biological disease activity. Rheumatology (Oxford) *57*, 921-930. 10.1093/rheumatology/kex490.
16. Tesser, A., Bocca, P., Ulivi, M., Pin, A., Pastorino, C., Cangelosi, D., Santori, E., Drago, E., Caorsi, R., Candotti, F., et al. (2025). Type I interferon signature: a quantitative standardized method for clinical application. Clin Exp Immunol *219*. 10.1093/cei/uxaf018.

Members of the Undiagnosed Diseases Network (Version 3.31.25)

Alyssa A. Tran, Arjun Tarakad, Ashok Balasubramanyam, Brendan H. Lee, Carlos A. Bacino, Daryl A. Scott, Elaine Seto, Gary D. Clark, Hongzheng Dai, Hsiao-Tuan Chao, Ivan Chinn, James P. Orengo, Jennifer E. Posey, Jill A. Rosenfeld, Kim Worley, Lindsay C. Burrage, Lisa T. Emrick, Lorraine Potocki, Monika Weisz Hubshman, Richard A. Lewis, Ronit Marom, Seema R. Lalani, Shamika Ketkar, Tiphanie P. Vogel, William J. Craigen, Jared Sninsky, Lauren Blieden, Sandesh Nagamani, Hugo J. Bellen, Michael F. Wangler, Oguz Kanca, Shinya Yamamoto, Christine M. Eng, Patricia A. Ward, Pengfei Liu, Adeline Vanderver, Cara Skraban, Edward Behrens, Gonench Kilich, Kathleen Sullivan, Kelly Hassey, Ramakrishnan Rajagopalan, Rebecca Ganetzky, Vishnu Cuddapah, Anna Raper, Daniel J. Rader, Giorgio Sirugo, Vaidehi Jobanputra, Allyn McConkie-Rosell, Kelly Schoch, Mohamad Mikati, Nicole M. Walley, Rebecca C. Spillmann, Vandana Shashi, Alan H. Beggs, Calum A. MacRae, David A. Sweetser, Deepak A. Rao, Edwin K. Silverman, Elizabeth L. Fieg, Frances High, Gerard T. Berry, Ingrid A. Holm, J. Carl Pallais, Joan M. Stoler, Joseph Loscalzo, Lance H. Rodan, Laurel A. Cobban, Lauren C. Briere, Matthew Coggins, Melissa Walker, Richard L. Maas, Susan Korrick, Jessica Douglas, Cecilia Esteves, Emily Glanton, Isaac S. Kohane, Kimberly LeBlanc, Rachel Mahoney, Shamil R. Sunyaev, Shilpa N. Kobren, Brett H. Graham, Erin Conboy, Francesco Vetrini, Kayla M. Treat, Khurram Liaqat, Lili Mantcheva, Stephanie M. Ware, Breanna Mitchell, Brendan C. Lanpher, Devin Oglesbee, Eric Klee, Filippo Pinto e Vairo, Ian R. Lanza, Kahlen Darr, Lindsay Mulvihill, Lisa Schimmenti, Queenie Tan, Surendra Dasari, Abdul Elkadri, Brett Bordini, Donald Basel, James Verbsky, Julie McCarrier, Michael Muriello, Michael Zimmermann, Adriana Rebelo, Carson A. Smith, Deborah Barbouth, Guney Bademci, Joanna M. Gonzalez, Kumarie Latchman, LéShon Peart, Mustafa Tekin, Nicholas Borja, Stephan Zuchner, Stephanie Bivona, Willa Thorson, Herman Taylor, Rakale C. Quarells, Ayuko Iverson, Bruce Gelb, Charlotte Cunningham-Rundles, Eric Gayle, Joanna Jen, Louise Bier, Mafalda Barbosa, Manisha Balwani, Mariya Shadrina, Rachel Evard, Saskia Shuman, Susan Shin, Andrea Gropman, Barbara N. Pusey Swerdzewski, Camilo Toro, Colleen E. Wahl, Donna Novacic, Ellen F. Macnamara, John J. Mulvihill, Maria T. Acosta, Precilla D'Souza, Valerie V. Maduro, Ben Afzali, Ben Solomon, Cynthia J. Tifft, David R. Adams, Elizabeth A. Burke, Francis Rossignol, Heidi Wood, Jiayu Fu, Joie Davis, Leoyklang Petcharet, Lynne A. Wolfe, Margaret Delgado, Marie Morimoto, Marla Sabaii, MayChristine V. Malicdan, Neil Hanchard, Orpa Jean-Marie, Wendy Introne, William A. Gahl, Yan Huang, Andrew Stergachis, Danny Miller, Elisabeth Rosenthal, Elizabeth Blue, Elsa Balton, Emily Shelkowitz, Eric Allenspach, Fuki M. Hisama, Gail P. Jarvik, Ghayda Mirzaa, Ian Glass, Kathleen A. Leppig, Katrina Dipple, Mark Wener, Martha Horike-Pyne, Michael Bamshad, Peter Byers, Runjun Kumar, Seth Perlman, Sirisak Chanprasert, Virginia Sybert, Wendy Raskind, Nitsuh K. Dargie, Chun-Hung Chan, Dr. Francisco Bustos velasq, Isum Ward, Jason Schend, Jennifer Morgan, Megan Bell, Miranda Leitheiser, Mohamad Saifeddine, Paul Berger, Rachel Li, Taylor Beagle, Alexander Miller, Beatriz Anguiano, Beth A. Martin, Brianna Tucker, Chloe M. Reuter, Devon Bonner, Elijah Kravets, Hector Rodrigo Mendez, Holly K. Tabor, Jacinda B. Sampson, Jason Hom, Jennefer N. Kohler, Jennifer Schymick, John E. Gorzynski, Jonathan A. Bernstein, Kevin S. Smith, Laura Keehan, Laurens Wiel, Matthew T. Wheeler, Meghan C. Halley, Mia Levanto, Page C. Goddard, Paul G. Fisher, Rachel A. Ungar, Raquel L. Alvarez, Sara Emami, Shruti Marwaha, Stephen B Montgomery, Suha Bachir, Tanner D Jensen, Taylor Maurer, Terra R. Coakley, Euan A. Ashley, Ali Al-Beshri, Anna Hurst, Brandon M Wilk, Bruce Korf, Elizabeth A Worthey, Kaitlin Callaway, Martin Rodriguez, Tammi Skelton, Tarun KK Mamidi, Andrew B. Crouse, Jordan Whitlock, Mariko Nakano-Okuno, Matthew Might, William E. Byrd, Albert R. La Spada, Changrui Xiao, Elizabeth C. Chao, Eric Vilain, Jose Abdenur, Kirsten Blanco, Maija-Rikka Steenari, Rebekah Barrick, Richard Chang, Sanaz Attaripour, Suzanne Sandmeyer, Tahseen Mozaffar, Alden Huang, Andres Vargas, Bianca E. Russell, Brent L. Fogel, Esteban C. Dell'Angelica, George Carvalho, Julian A. Martínez-Agosto, Layal F. Abi Farraj, Manish J. Butte, Martin

G. Martin, Naghmeh Dorrani, Neil H. Parker, Rosario I. Corona, Stanley F. Nelson, Yigit Karasozen, Aaron Quinlan, Alistair Ward, Ashley Andrews, Corrine K. Welt, Dave Viskochil, Erin E. Baldwin, John Carey, Justin Alvey, Laura Pace, Lorenzo Botto, Nicola Longo, Paolo Moretti, Rebecca Overbury, Russell Butterfield, Steven Boyden, Thomas J. Nicholas, Matt Velinder, Gabor Marth, Pinar Bayrak-Toydemir, Rong Mao, Monte Westerfield, Brian Corner, John A. Phillips III, Kimberly Ezell, Lynette Rives, Rizwan Hamid, Serena Neumann, Ashley McMinn, Joy D. Cogan, Thomas Cassini, Alex Paul, Dana Kiley, Daniel Wegner, Erin McRoy, Jennifer Wambach, Kathy Sisco, Patricia Dickson, F. Sessions Cole, Dustin Baldridge, Jimann Shin, Lilianna Solnica-Krezel, Stephen C. Pak, Timothy Schedl, Allen Bale, Carol Oladele, Caroline Hendry, Emily Wang, Hua Xu, Hui Zhang, Lauren Jeffries, María José Ortuño Romero, Mark Gerstein, Michele Spencer-Manzon, Monkol Lek, Nada Derar, Odelya Kaufman, Shrikant Mane, Teodoro Jerves Serrano, Vasilis Vasiliou, Winston Halstead, Yong-Hui Jiang
