# Supplementary material for: Monoallelic PSMB8 variants cause PRAAS with immunodeficiency through impaired immunoproteasome assembly
Source: Am J Hum Genet. 2026 May 21;113(6):1214–32. doi: 10.1016/j.ajhg.2026.04.015 (PMC13277692; doi:10.1016/j.ajhg.2026.04.015)
Supplement: Document S3. Article plus supplemental information [file mmc3.pdf]

Monoallelic *PSMB8* variants cause PRAAS with immunodeficiency through impaired immunoproteasome assembly

Graphical abstract

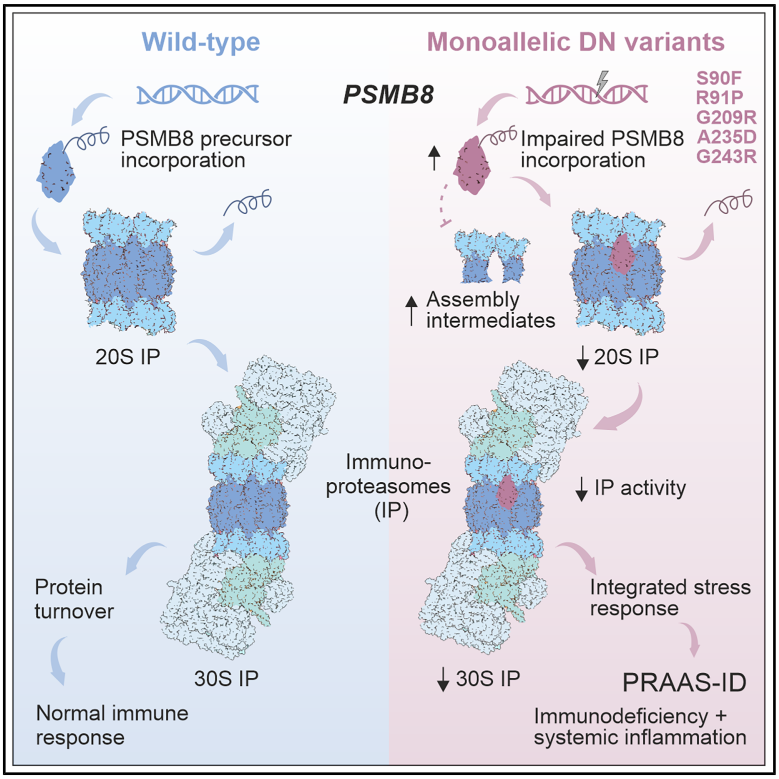

Authors

Robin Wijngaard,
Caspar I. van der Made,
Sema Kalkan Uçar, ...,
Saskia B. Wortmann,
Machteld M. Oud,
Sergio Guerrero-Castillo

Correspondence

machteld.oud@radboudumc.nl
(M.M.O.),
s.guerrerocastillo@uke.de (S.G.-C.)

Seven individuals from five families harboring monoallelic *PSMB8* variants presented with immunodeficiency and systemic inflammation. Mutant PSMB8 proteins are inefficiently incorporated into immunoproteasome complexes, impairing assembly and triggering cellular stress responses. This dominant-negative mechanism unifies PRAAS-IDs and highlights structural vulnerabilities in catalytic subunits.

Wijngaard et al., 2026, The American Journal of Human Genetics *113*, 1214–1232
June 4, 2026 © 2026 The Authors. Published by Elsevier Inc. on behalf of American Society of Human Genetics.
https://doi.org/10.1016/j.ajhg.2026.04.015

ARTICLE

Monoallelic *PSMB8* variants cause PRAAS with immunodeficiency through impaired immunoproteasome assembly

Robin Wijngaard,[1,29] Caspar I. van der Made,[1,2,29] Sema Kalkan Uçar,[3,30] Gayatri Ramakrishnan,[4,30] Man Wang,[5] Johannes Brand,[5] Jill A. Rosenfeld,[6] Tiphanie P. Vogel,[7] Sarah K. Nicholas,[8] Monika Weisz-Hubshman,[6,9] Undiagnosed Diseases Network, Clara D.M. van Karnebeek,[10] Eric J. Allenspach,[11,12] Taylor E. Gardiner,[12] Sumudu Perera Kimmantudawage,[13] Zornitza Stark,[13,14] Ruth K. Armstrong,[15] Janine Campbell,[14,16] Stefano Volpi,[17,18] Enrico Drago,[17,18,19] Marco Gattorno,[17] Alice Grossi,[20] Isabella Ceccherini,[20] Alfredo Cabrera-Orefice,[21] Bente Siebels,[22] Thomas Mair,[22] Hartmut Schlüter,[22] Ruben L. Smeets,[23,24] Ronald van Beek,[1] Ingrid Goebel,[25,26] Katrin Küchler,[25,26] Søren W. Gersting,[25,26] Alexander Hoischen,[1,2] Lisenka E.L.M. Vissers,[1] Ron A. Wevers,[27] Catherine Meyer-Schwesinger,[5] Saskia B. Wortmann,[28] Machteld M. Oud,[1,31,*] and Sergio Guerrero-Castillo[25,26,31,*]

Summary

Monoallelic variants in catalytic immunoproteasome subunits have recently been linked to proteasome-associated autoinflammatory syndromes with immunodeficiency (PRAAS-ID), yet their molecular mechanisms and clinical spectra are not fully defined. In this study, seven individuals from five unrelated families carrying five distinct monoallelic *PSMB8* variants were identified. Individuals presented with neonatal-onset immunodeficiency characterized by recurrent infections, B cell lymphopenia, and hypogammaglobulinemia requiring immunoglobulin replacement. Inflammatory manifestations of variable severity included enteropathy, hepatitis, myositis, and inflammatory lung disease. Additional findings included leukocyte vacuolization in blood and bone marrow. Pathogenic variants in immunoproteasome subunits were analyzed to identify structural features associated with dominant-negative behavior. Immunoproteasome assembly and activity were investigated using complexome profiling, immunoblotting, and in-gel activity assays in proband-derived fibroblasts and transfected HEK293T cells, with downstream effects assessed by proteomic and RT-qPCR analyses. Mutant PSMB8 subunits were inefficiently incorporated into immunoproteasome complexes, leading to impaired assembly, including reduced fully assembled complexes and accumulation of assembly intermediates. This defect was accompanied by activation of the integrated stress response alongside impaired immune signaling. Monoallelic pathogenic variants in *PSMB8*, *PSMB9*, and *PSMB10* associated with PRAAS-ID affected residues that are highly conserved and biophysically similar between the three immunoproteasome catalytic subunits. These shared structural features may help identify additional variants with similar disruptive effects on immunoproteasome assembly. Together, our data show that monoallelic *PSMB8* variants disrupt immunoproteasome assembly, resulting in clinically variable disease with immunodeficiency and systemic

[1]Department of Human Genetics, Radboud University Medical Center, Nijmegen, the Netherlands; [2]Department of Internal Medicine and Radboud Center for Infectious Diseases (RCI), Radboud University Medical Center, Nijmegen, the Netherlands; [3]Division of Pediatric Nutrition and Metabolism, Department of Pediatrics, Ege University Faculty of Medicine, İzmir, Turkey; [4]Bangalore, India; [5]Institute of Cellular and Integrative Physiology, University Medical Center Hamburg-Eppendorf, Hamburg, Germany; [6]Department of Molecular and Human Genetics, Baylor College of Medicine, Houston, TX, USA; [7]Division of Rheumatology, Department of Pediatrics, Baylor College of Medicine and Center for Human Immunobiology, Texas Children's Hospital, Houston, TX, USA; [8]Division of Immunology, Allergy, and Retrovirology, Department of Pediatrics, Baylor College of Medicine, Houston, TX, USA; [9]Genetics Department, Texas Children's Hospital, Houston, TX, USA; [10]Departments of Pediatrics and Human Genetics, Emma Center for Personalized Medicine, Amsterdam University Medical Centers, Amsterdam, the Netherlands; [11]Department of Pediatrics, Divisions of Immunology and Pediatric Rheumatology, University of Washington, Seattle, WA, USA; [12]Seattle Children's Hospital, Division of Immunology, Seattle, WA, USA; [13]Victorian Clinical Genetics Services, Murdoch Children's Research Institute, Melbourne, VIC, Australia; [14]Department of Paediatrics, University of Melbourne, Melbourne, VIC, Australia; [15]Department of Neonatal Medicine, The Royal Children's Hospital, Melbourne, VIC, Australia; [16]Department of Clinical Haematology, The Royal Children's Hospital, Melbourne, VIC, Australia; [17]UOC Clinical and Experimental Immunology, IRCCS Istituto Giannina Gaslini, Genoa, Italy; [18]Department of Neuroscience, Rehabilitation, Ophthalmology, Genetics, Maternal and Child Health (DINOGMI), Università Degli Studi di Genova, Genova, Italy; [19]Gene Therapy Program, Dana Farber/Boston Children's Cancer and Blood Disorders Center, Harvard Medical School, Boston, MA, USA; [20]UOSD Area Aggregazione Laboratori Della Ricerca, IRCCS Istituto Giannina Gaslini, Genoa, Italy; [21]Research Institute for Medical Innovation, Radboud University Medical Center, Nijmegen, the Netherlands; [22]Section Mass Spectrometry and Proteomics, University Medical Center Hamburg-Eppendorf, Hamburg, Germany; [23]Department of Laboratory Medicine, Laboratory of Medical Immunology, Radboud University Medical Center, Nijmegen, the Netherlands; [24]Department of Laboratory Medicine, Radboudumc Laboratory for Diagnostics, Radboud University Medical Center, Nijmegen, the Netherlands; [25]University Children's Research@Kinder-UKE, University Medical Center Hamburg-Eppendorf, Hamburg, Germany; [26]German Center for Child and Adolescent Health (DZKJ), Partner Site Hamburg, Hamburg, Germany; [27]Translational Metabolic Laboratory, Department of Human Genetics, Radboud University Medical Center, Nijmegen, the Netherlands; [28]University Children's Hospital, Paracelsus Medical University, Salzburg, Austria
[29]These authors contributed equally
[30]These authors contributed equally
[31]These authors contributed equally
*Correspondence: machteld.oud@radboudumc.nl (M.M.O.), s.guerrerocastillo@uke.de (S.G.-C.)
https://doi.org/10.1016/j.ajhg.2026.04.015.
© 2026 The Authors. Published by Elsevier Inc. on behalf of American Society of Human Genetics.
This is an open access article under the CC BY license (http://creativecommons.org/licenses/by/4.0/).

inflammation. Our findings support immunoproteasome assembly disruption as a unifying dominant-negative mechanism underlying PRAAS-ID.

Introduction

Proteasomes are large multiprotein complexes that mediate protein degradation within cells, primarily targeting ubiquitinated proteins but also processing non-ubiquitinated substrates.[1] The proteasome is composed of a catalytic 20S core particle combined with one or two regulatory subcomplexes that modulate its activity, including 19S or PA700, which, together with the 20S, form the 26S or 30S proteasome, the 11S or PA28, and PA200.[1–3] The 20S core particle is a cylindrical structure composed of four stacked rings: two outer rings of seven α subunits and two inner rings of seven β subunits.[4] In the constitutively expressed standard proteasome (SP), the β rings contain three catalytic subunits, PSMB6 (β1), PSMB7 (β2), and PSMB5 (β5), with caspase-, trypsin-, and chymotrypsin-like activity, respectively.[5] These subunits are synthesized as precursors and activated through N-terminal propeptide cleavage. In addition, tissue-specific forms exist, including the immunoproteasome (IP), the thymoproteasome, and the spermatoproteasome. In the IP, expressed in immune cells or in other cell types in response to pro-inflammatory cytokines such as interferon-γ (IFNγ), the SP catalytic subunits are replaced by PSMB9 (β1i), PSMB10 (β2i), and PSMB8 (β5i).[6–8] These specialized subunits provide the IP with peptide-cleavage properties optimized for antigen presentation via major histocompatibility complex (MHC) class I, supporting adaptive immunity.[9–11]

Assembly of the 20S proteasome core is a highly ordered and regulated process.[4,12–14] Formation begins with an α ring scaffold, followed by stepwise incorporation of β subunits to generate a half-mer or 13S intermediate, which subsequently dimerizes to form the mature 20S particle.[3,13,15] The assembly process is tightly regulated by assembly chaperones, such as PSMG1-4 or POMP, which disassociate as assembly progresses and are absent in the mature complexes.[13] Notably, the incorporation order of certain β subunits differs between the IP and the SP; for example, the inducible subunit PSMB9 can enter precursor complexes earlier than its constitutive homolog PSMB6.[3,8,15,16] These distinct incorporation kinetics promote preferential formation of homogeneous SP or IP particles, although mixed proteasomes containing combinations of inducible and constitutive catalytic subunits have also been demonstrated.[12,17,18]

Dysfunction of the IP gives rise to a group of inborn errors of immunity known as proteasome-associated autoinflammatory syndromes (PRAAS). PRAAS are characterized by sustained pathological expression of type I IFN cytokines and multisystemic hyperinflammation, including rash, fevers, and organ dysfunction.[19,20] Classical PRAAS follows autosomal-recessive or oligogenic inheritance involving two or more variants in IP catalytic subunits, assembly chaperones, or shared subunits between the SP and the IP (MIM: 256040, 617591, 619183, and 619175).[21–26] However, autosomal-dominant inheritance has been described for some subunits (MIM: 618048, 620796, and 620807). These disorders, often referred to as PRAAS with immunodeficiency (PRAAS-ID), are thought to act via dominant-negative mechanisms that disrupt proteasome assembly.[27–30]

Here, we show that multiple monoallelic *PSMB8* (MIM: 177046) variants impair IP assembly, leading to clinically variable immunodeficiency and inflammatory disease, with leukocyte vacuolization and inclusions observed in some individuals. The variants share structural, biophysical, and functional consequences with previously described monoallelic variants in *PSMB9* (MIM: 177045) and *PSMB10* (MIM: 176847), providing a unifying molecular explanation for PRAAS-ID and expanding its clinical and genetic spectrum.

Material and methods

Inclusion and ethical considerations

Seven individuals from five families were identified through research projects at their respective centers and connected via GeneMatcher.[31] All studies were approved by the relevant local institutional review boards or ethics committees, and written informed consent was obtained from participants or their caregivers, including consent for publication of clinical images if appropriate. Detailed information on recruitment and ethics approvals is provided in the supplemental material and methods.

Data collection and genetic analysis

Clinical and laboratory data were obtained during routine diagnostic evaluations at each center. Sequencing was performed locally using standard exome or genome sequencing protocols on DNA extracted from blood, buccal swabs, or frozen brain tissue. Additional family members were sequenced to determine the variant's mode of inheritance. Family-specific details are provided in the supplemental material and methods.

Structural biology analysis

We modeled the *PSMB8* variants in the crystal structures of the human 20S IP containing PSMB8 (PDB: 6E5B) and the human 26S SP using the paralogous PSMB5 subunit (PDB: 6MSB, 60.3% sequence identity), to model the PSMB8 variants.[32,33] Structural models were generated using RepairPDB and BuildModel functions, with five iterations of side-chain rotamer adjustments. Changes in protein stability were estimated using the FoldX energy function (v.5.0), with average free energy differences between wild-type (WT) and variant structures in kcal/mol.[34] Frustration index (FI) analysis was computed to account for shifts in energetic distributions in the variant environment using Frustratometer2.[35] Mutational and configurational FIs and residue contact interactions were calculated for residues within 5 Å centered at Cα of the variant site. A contact was considered minimally

frustrated if scores were >0.78, highly frustrated if they were <−1.00, and neutral in between.

To investigate whether paralogous positions across proteasome subunits show similar structural and evolutionary properties, 21 described pathogenic missense variants affecting the mature regions of PSMB8, PSMB9, and PSMB10 were manually curated from the literature and ClinVar (including those identified in this study) (Table S1). Variants were classified according to disease association as classical PRAAS for variants reported in the context of recessive disease and PRAAS-ID for variants reported in the context of dominant disease. The variants were modeled across all 17 paralogous proteasome subunits of the 20S core particle (PSMA1–7 and PSMB1–10) by introducing equivalent amino acid substitutions at aligned positions. Evolutionary conservation was assessed using Rate4Site scores derived from multiple sequence alignments built from protein sequences with ≥70% identity retrieved from UniProt.[36] The distribution of variant locations within the protein structure was assessed by calculating their relative solvent accessibility (RSA) using FreeSASA.[37] For each residue, RSA was calculated as the ratio of its absolute solvent-accessible surface area (in $Å^2$) to its corresponding maximum allowed solvent accessibilities. Residues were categorized as exposed when RSA was ≥20% and buried when RSA was <20%.

Cell lines

Human osteosarcoma 143B and human monocytic THP-1 cells, used as non-immune and immune cell models, respectively, as well as human skin fibroblasts from healthy control subjects and from individuals 1 and 5 carrying the p.Ser90Phe (c.269C>T) and p.Ala235Asp (c.704C>A) variants, were cultured under standard conditions (supplemental material and methods).

Transient expression of WT and mutant *PSMB8* constructs in HEK293T cells

The pcDNA3.1/V5-His TOPO vector encoding human WT *PSMB8*, kindly provided by Elke Krüger, University Medicine Greifswald, Germany, was used as a template to generate nine *PSMB8* variant constructs by site-directed mutagenesis (Table S2).[30] HEK293T cells were transiently transfected for 24 h using Lipofectamine 2000 (Thermo Fisher Scientific, Carlsbad, CA, USA) following the supplier's instructions.

RNA isolation and quantitative reverse-transcription PCR

RNA from six biological replicates from fibroblasts from individual 5 and eight biological replicates from unaffected family members was extracted using the NucleoSpin RNA kit (Macherey-Nagel, Düren, Germany) according to the manufacturer's instructions. cDNA synthesis was performed using the iScript cDNA synthesis kit (Bio-Rad, Hercules, CA, USA). Relative expression levels of *HSPA5*, *ATF4*, *DDIT3*, and spliced *XBP1* (*sXBP1*) with *SDHA* as a reference gene were determined by RT-qPCR using GoTaq Green Master Mix (Promega, Madison, WI, USA). Primers are listed in Table S3.

Protein extraction and immunoblotting

To maintain the activity of proteasomes, fibroblasts were lysed by freeze-thaw cycles, and the cytosolic fraction was used for immunoblotting and proteasome activity assays (supplemental material and methods).

In-gel proteasome proteolytic activity assay and determination of active proteasome subunit abundance

The chymotrypsin-like activity of the proteasome was assessed with an in-gel fluorescence technique (supplemental material and methods). Briefly, lysates were separated by native gel electrophoresis. Gels were incubated in the presence of proteasomal substrate Ac-ANW-AMC, releasing aminomethylcumarin (AMC), which becomes fluorescent after substrate cleavage. Additionally, activity-based probes (ABPs) were used to assess the amount of active proteasome catalytic subunits (supplemental material and methods).

Complexome profiling

Briefly, cells were mechanically disrupted, and homogenates were solubilized with digitonin and separated by blue native gel electrophoresis. Triplicate gel lanes from 143B and THP-1 cells were further processed. Fibroblasts stimulated with IFNγ from 2 healthy control subjects (one gel lane from each individual) and two gel lanes from individual 5 carrying the p.Ala235Asp variant were analyzed by complexome profiling.[38,39] In addition, fibroblasts from individual 1 carrying the p.Ser90Phe variant were analyzed in a separate batch for qualitative comparison of migration patterns and were therefore not included in quantitative analyses. Entire gel lanes were cut into 60 fractions and digested in gel with trypsin, and peptides were subjected to liquid chromatography-tandem mass spectrometry (LC-MS/MS) to analyze the protein composition and abundance in each gel fraction. Protein migration profiles were grouped together based on their similarities using agglomerative hierarchical clustering analysis and visualized as abundance heatmaps. A full description of the technique can be found in the supplemental material and methods.

Statistics

Protein complex quantifications from complexome profiling data were calculated as the mean protein abundance of fibroblasts from two healthy control subjects and from two replicate cultures from individuals 1 and 5. The sum of all protein abundance values across the 60 fractions was used to calculate the total protein intensities. Relative gene expression from quantitative reverse-transcription PCR (RT-qPCR) was calculated using the comparative Ct ($2^{-\Delta\Delta Ct}$) method. Differences between groups were analyzed using a two-sided Student's *t* test or Wilcoxon test, as appropriate, and corrected for multiple testing. Graph Prism 6 and R were used to visualize the results. Error bars represent the standard deviation.

Results

Characterization of individuals with early-onset immunodeficiency and variable systemic inflammation

We identified seven individuals from five unrelated families with multisystemic inflammation and signs of immunodeficiency from the first year of life (Figure 1A; Table 1). Detailed clinical descriptions are provided in the supplemental note (case reports) and Table S4. The phenotypic presentations of the affected individuals can be

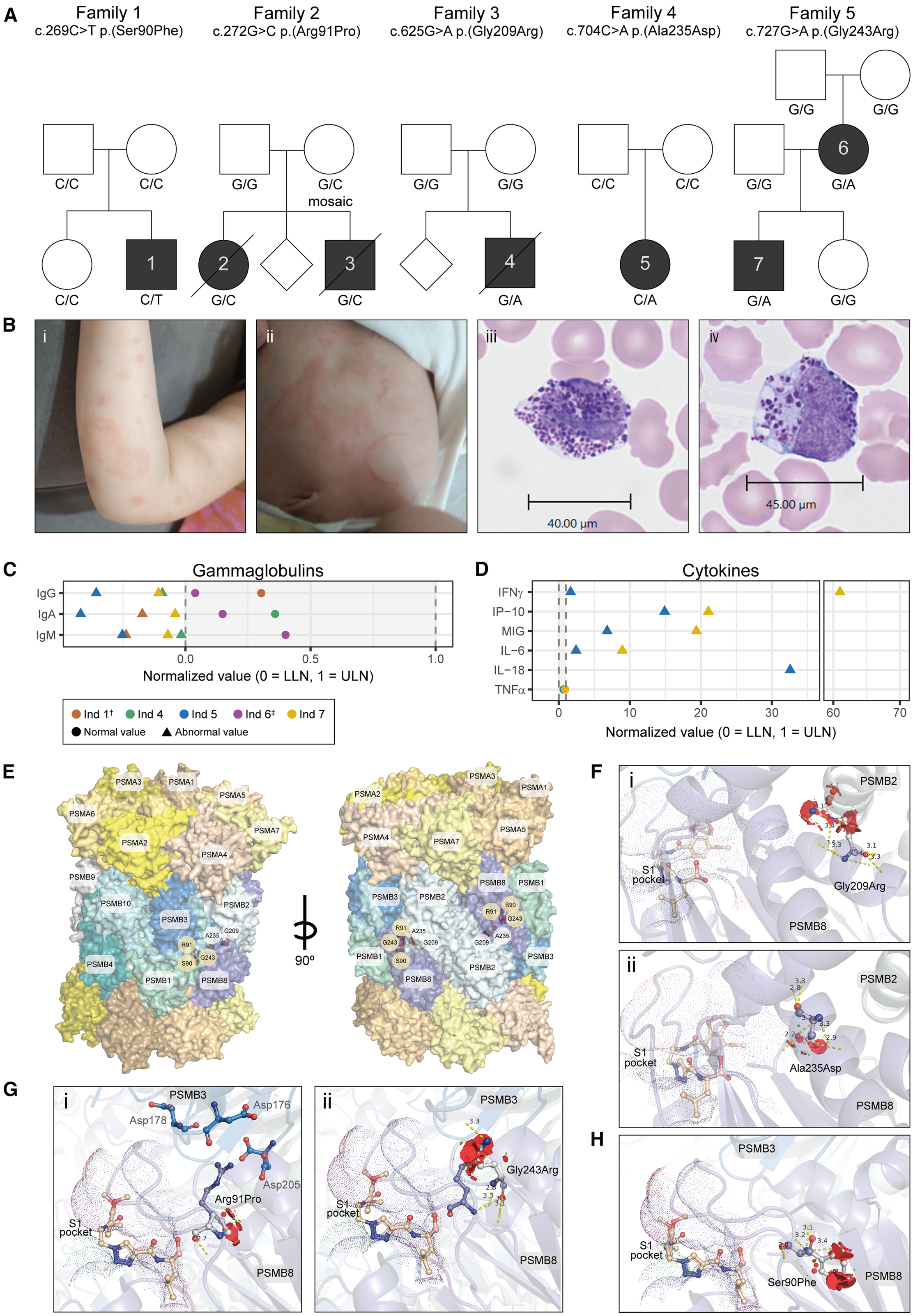

Figure 1. Clinical findings and structural impact of *PSMB8* variants

(A) Pedigrees of the five included families with monoallelic variants in *PSMB8* (GenBank: NM_148919.4). Affected individuals are numbered 1–7.

(B) Clinical signs and symptoms seen in individuals 4, 5, and 7. Erythematous, edematous plaques on the arm of individual 5 at age 3 years (i). Rash of individual 7 (ii). Peripheral blood cells from individual 4 showing a neutrophil (iii) and a monocyte (iv) with abnormal coarse, deep pink to purple staining inclusions.

(legend continued on next page)

placed in a clinical spectrum ranging from mild episodic infections to severe continuous multiorgan inflammation. Individual 6 presented with only skin eruption and did not develop febrile episodes, progressive interstitial lung disease, or bronchiectasis before adolescence. In contrast, individual 4 presented with very early-onset hydrops fetalis and severe pulmonary hypertension necessitating emergency Caesarian section and advanced respiratory support, leading to fatal multiorgan failure at just 2 months of age. The most prominent clinical findings were (inflammatory) lung disease (5/6), lymphopenia (5/5), enteropathy (5/7), transaminitis (4/7), myositis (4/7), and rhabdomyolysis (3/7). Infections were most often caused by viruses, including adenovirus, rhinovirus, enterovirus, parainfluenza virus, and respiratory syncytial virus (RSV), and bacteria, including *Streptococcus pneumoniae*, *Haemophilus influenzae*, and *Haemophilus haemolyticus*. Several individuals (4/7) suffered from periodic exacerbations with fever, recurrent infections with or without skin rash, and transaminitis (Figure 1B). Both severely affected children from family 2 died during infancy due to pneumonia and meningitis, respectively. Postmortem examination revealed basal ganglia calcification in both individuals. Individual 1 underwent lung transplantation due to devastating lung damage caused by necrotizing acute bronchitis and bronchiectasis.

Immunophenotyping in individuals 1, 4, 5, 6, and 7 revealed significant and repeated reductions in B cell percentages, accompanied by a reduced relative number of memory B cells in individuals 1 and 7 (Table S5). Natural killer (NK) cell percentages were variable across individuals, while T cell percentages remained normal. Additional immunological analyses demonstrated hypogammaglobulinemia (Figure 1C), necessitating ongoing intravenous immunoglobulin (IVIG) therapy. IFN scores were markedly elevated in individuals 4 and 5, mildly elevated in individual 1 (measurement was performed during immunosuppressive treatment following lung transplantation), and normal in individual 7 (supplemental material and methods; Figure S1). Cytokine profiling of individuals 5 and 7 during follow-up suggested marked activation of the IFN pathway, along with innate immune activation, including elevated serum levels of IL-6 and IL-18 (Figure 1D; supplemental material and methods; Table S6).

Hematological abnormalities, other than lymphopenia, included (intermittent) thrombocytopenia (5/7) and anemia (5/7). Strikingly, a bone marrow examination of individual 4 demonstrated moderate dyserythropoiesis, near-absent megakaryocytes, and occasional leukocytes in different developmental stages with vacuoles and coarse, deep pink to purple cytoplasmic inclusions (Figure 1B). Leukocyte vacuoles were also demonstrated in the peripheral blood of individual 1. Furthermore, individual 5 developed a combination of clinical features and laboratory findings that were suggestive of hemophagocytic lymphohistiocytosis, including fever, bicytopenia, hyperferritinemia, splenomegaly, and hypertriglyceridemia, and was treated with corticosteroids.

Genetic analysis identified heterozygous missense variants in *PSMB8*

Monoallelic missense variants in *PSMB8* were prioritized in all families: c.269C>T (p.Ser90Phe), c.272G>C (p.Arg91Pro), c.625G>A (p.Gly209Arg), c.704C>A (p.Ala235Asp), and c.727G>A (p.Gly243Arg) (Table 1; Figure S2). The variants were confirmed *de novo* in individuals 1, 4, and 5. Individuals 2 and 3 inherited the variant from the unaffected mother, in whom low-level mosaicism was detected in a buccal swab (4 of 143 reads; 3%). In family 5, the variant was confirmed *de novo* in the affected mother (individual 6) and transmitted to the affected child (individual 7). All variants were absent from gnomAD v.4.1.0, predicted to be deleterious by multiple *in silico* predictors, and classified as variants of uncertain significance or likely pathogenic following the ACMG criteria (Table 1).[40]

Given the possibility of digenic inheritance, all other proteasome subunit genes were screened for second hit variants. No rare exonic variants were identified in any family, and in those with genome sequencing, possible pathogenic intronic variants were also excluded. Other genetic findings in the families are included in Table S7. Based on the consistent identification of strong *PSMB8* variants and clinical overlap across families, *PSMB8* was considered the most likely genetic candidate in all cases.

The identified *PSMB8* variants destabilize the 20S and 26S complexes *in silico*

We investigated the structural impact of *PSMB8* variants within the 20S IP and 26S SP complexes through *in silico* modeling. All residues were retained in the mature protein and were highly conserved, as indicated by ConSurf scores (Figure S3).[41] Moreover, all variants exhibited increased free energy (ΔΔG) values compared with the WT, indicating a destabilizing effect on both the 20S and 26S complexes (Table S8).

Residues Gly209 and Ala235 are located near or at the PSMB8-PSMB2 β ring interface. Both residues are substituted by larger, charged amino acids, causing steric

(C and D) Normalized gammaglobulin and cytokine values. The gray area between the dashed lines represents the reference range, with 0 corresponding to the lower limit of normal (LLN) and 1 to the upper limit of normal (ULN). †, values measured during IVIG treatment; ‡, values of low IgG recorded during childhood.
(E) Crystal structure of the 20S immunoproteasome assembly with the variants indicated.
(F) Close-up view of the p.Gly209Arg (i) and the p.Ala235Asp (ii) variants located at the PSMB8-PSMB2 interface.
(G) Close-up view of the p.Arg91Pro (i) and p.Gly243Arg (ii) variants located at the PSMB8-PSMB3 interface.
(H) Close-up view of the p.Ser90Phe variant located near the S1 pocket of the active site.
Variants that introduce steric clashes in the neighborhood are indicated as red discs.

Table 1. Demographic, genetic, clinical, and immunological laboratory findings of included individuals

	Family 1, I1	Family 2, I2	Family 2, I3	Family 3, I4	Family 4, I5	Family 5, I6	Family 5, I7
**Demographics and genetics**							
Sex	male	female	male	male	female	female	male
PSMB8 variant (GenBank: NM_148919.4)	c.269C>T (p.Ser90Phe)	c.272G>C (p.Arg91Pro)	c.272G>C (p.Arg91Pro)	c.625G>A (p.Gly209Arg)	c.704C>A (p.Ala235Asp)	c.727G>A (p.Gly243Arg)	c.727G>A (p.Gly243Arg)
Inheritance	*de novo*	inherited from mother (mosaic 3%)	inherited from mother (mosaic 3%)	*de novo*	*de novo*	*de novo*	inherited from mother (I6)
CADD	29.6	29.1	29.1	29.1	29.7	29.7	29.7
AlphaMissense	0.973 (LP)	0.9864 (LP)	0.9864 (LP)	0.842 (LP)	0.987 (LP)	0.897 (LP)	0.897 (LP)
gnomAD AF v.4.1.0	absent	absent	absent	absent	absent	absent	absent
ACMG classification	LP (PS2, PM2_Supporting, PP3_Moderate)	VUS (PM2_Supporting, PP1_Moderate, PP3_Moderate)	VUS (PM2_Supporting, PP1_Moderate, PP3_Moderate)	LP (PS2, PM2_ Supporting, PP3_Moderate)	LP (PS2, PM2_ Supporting, PP3_Moderate)	LP (PS2, PM2_ Supporting, PP1_Moderate, PP3_Moderate)	LP (PS2, PM2_ Supporting, PP1_Moderate, PP3_Moderate)
**Clinical features**							
Age at							
Presentation	3 months	7 months	6 months	congenital	3 months	adolescence	2 days
Current	12 years	16 months*	11 months*	68 days*	4 years	34 years	4 years
Recurrent fever	–	+	+	–	+	+	–
Recurrent infections	+	+	+	–	+	+/–	–
Bronchitis (upper respiratory tract)	+	+	–	–	–	–	–
Otitis	+	+	+	–	–	–	–
Skin rash	–	–	–	+	+	+**	+
Systemic inflammation	–	+	+	+	+	–	–
Inflammatory lung disease	+	+	+	N/D	+	+	–
Pulmonary hypertension	–	–	–	+	+	–	–
Enteropathy	+	+	–	+	+	–	+
Liver dysfunction	+	–	–	+	+	–	+
Myositis/muscle atrophy	+	–	–	–	+	+	+
Rhabdomyolysis	–	–	–	–	+	+	+
Basal ganglia calcification	–	+	+	N/D	–	–	–

(*Continued on next page*)

Table 1. *Continued*

	Family 1, I1	Family 2, I2	Family 2, I3	Family 3, I4	Family 4, I5	Family 5, I6	Family 5, I7
Poor growth/short stature	+	–	–	+	–	+	+
Other	lung transplantation at age 10 years, bilateral conductive hearing loss, multiple skin warts	focal seizures (11 months)	–	prematurity (28 completed weeks' gestation), hydrops fetalis, cardiac hypertrophy, metaphyseal dysplasia (moth-eaten) long bones	hypothyroidism	late preterm, hypothyroidism	acute myo-pericarditis, hypothyroidism, multiple allergies
**Laboratory results**							
Dyslipidemia	+	N/D	N/D	N/D	+	N/D	–
Autoantibodies	N/D	N/D	N/D	–	–	–	–
Immunoglobulins	↓	N/D	N/D	↓	↓	↓ (transient)**	↓
Anemia	+	+	–	+	+	–	+
Thrombocytopenia	+	+	+	+	+	–	–
Lymphopenia	+	N/D	N/D	+	+	+	+
B cells	↓	N/D	N/D	↓	↓	↓	↓
T cells	normal	N/D	N/D	normal	normal	normal	normal
NK cells	↓	N/D	N/D	normal	variable	normal	normal
Eosinophilia	–	–	N/D	–	–	–	+
Leukocyte inclusions	+	N/D	N/D	+	–	N/D	N/D
IFN signature	↑	N/D	N/D	↑	↑	N/D	normal

ACMG, American College of Medical Genetics and Genomics; CADD, Combined Annotation Dependent Depletion; d, days; gnomAD AF, Genome Aggregation Database allele frequency; IFN, interferon; LP, likely pathogenic; N/D, not determined; VUS, variant of uncertain significance; y, years; +, present; –, absent; ↑, increased; ↓, decreased; *, deceased; **, started during the first year of life.

clashes that likely disrupt interactions with PSMB2 and are incompatible with the surrounding hydrophobic environment (Figures 1E and 1F). In contrast, Ser90, Arg91, and Gly243 are close to the S1 pocket of the active site and the interface with PSMB3 (Figure 1E). Arg91 forms stable salt bridges with aspartates in PSMB3, which are lost upon mutation (Figure 1G). The p.Gly243Arg substitution produces substantial shifts due to the long side chain of the Arg residue that may interfere with residue interactions at the PSMB8-PSMB3 interface (Figures 1G and S4). p.Ser90Phe is likely to exert allosteric effects on the S1 pocket, with the bulky, hydrophobic phenylalanine increasing rigidity of the active site loop, thereby altering local structure and function (Figure 1H).

Moreover, protein frustration analyses revealed that all variants induced changes in neighboring residues by altering local energy frustrations and/or changing local contact densities, rewiring the surrounding structure (Table S8; Figure S5). Together, these data suggest that the monoallelic variants exert their pathogenic effect through broad structural destabilization of the complex, in line with a dominant-negative disease mechanism.

Monoallelic variants lead to defective IP biogenesis with buildup of assembly intermediates

Complexome profiling, a technique that separates protein complexes under native conditions to analyze their composition and abundance by MS, was employed to assess proteasome assembly. Using a non-immune (143B) and an immune (THP-1) cell line, we first validated the specificity and sensitivity of this method to distinguish IP from SP through clear detection and quantification of IP-specific subunits (PSMB8-10) and SP-specific subunits (PSMB5-7) (supplemental note: additional complexome profiling findings; Figure S6). To enhance IP expression in fibroblasts, IFNγ stimulation was applied, which markedly increased the IP fraction from ~15% to ~65% of total proteasome abundance (Figure S7).

Under IFNγ stimulation conditions, fibroblasts carrying p.Ala235Asp showed an approximately 50% decrease in the abundance of the IP-specific 20S and 26S complexes compared with controls, accompanied by a significant reduction of all IP-specific catalytic subunits in both complexes (Figures 2A–2D). In contrast, SP-specific complexes and subunits remained unchanged (Figures 2A–2D). Consequently, the relative abundance of IP compared with SP was lower in the p.Ala235Asp line than in controls (1.4 versus 0.8) (Figure 2E). Fibroblasts carrying p.Ser90-Phe showed similarly low relative IP levels (0.7), most likely due to a reduced IP in this sample (Figures 2E and S8). Native immunoblotting under basal conditions confirmed an approximately 50% reduction in PSMB8-containing IP complexes in both variants, whereas PSMB5-containing SP assemblies were not significantly altered (Figures 2F and 2G). Upon IFNγ stimulation, IP formation was partially restored, with a more pronounced compensatory effect in the p.Ser90Phe line (Figure S9). This assembly defect resulted in reduced IP-dependent proteasome activity, with a ~40% decrease in ANW-AMC activity of 26S and 30S proteasomes after normalization to total proteasome abundance, whereas 20S-associated activity remained preserved (Figures 2F and 2G). Despite reduced activity, no accumulation of total ubiquitinated proteins was detected (Figures S9E and S9F).

Consistent with impaired IP assembly, mutant fibroblasts showed a pronounced ~440-kDa IP-associated peak that was nearly absent in controls, comprising all α subunits together with PSMB2, PSMB3, PSMB9, and PSMB10 (Figures 2A–2C, S8, 3A, and S10). Accumulation of an assembly intermediate was further supported by native immunoblotting of IFNγ-stimulated cells, revealing a band migrating below the 20S complex in mutant lines that contained PSMA3 and PSMB9 but lacked PSMB8 (marked as 440; Figure 3B). Despite the incorporation of PSMB9, no caspase-like activity was detected, indicating that the complex is likely catalytically inactive (Figure 3B). In the complexome profiling data, assembly chaperones PSMG1–4 and POMP co-migrated with the ~440-kDa intermediate in both mutants (Figures 3D, 3E, and S10). Moreover, an additional peak at ~560 kDa was detected in p.Ala235Asp, most likely representing a transitional assembly state in which the PSMG1–4 chaperones are displaced by the PA28 heptameric regulator (Figures 3D–3G).

Collectively, these findings indicate impaired IP biogenesis, including reduced formation of mature IP complexes and accumulation of assembly intermediates (Figure 3H). Regulatory particle subassemblies formed independently of the 20S core showed no alterations (Figure S11).

Accumulation of immature PSMB8 indicates defective proteasome incorporation

To further define the molecular basis of the assembly defect, we examined PSMB8 processing and incorporation. Complexome profiling showed similar overall PSMB8 abundance but a 4.5-fold enrichment of PSMB8-derived peptides at the gel front, including precursor peptides, and a 30% reduced incorporation into assembled complexes (Figures 4A–4D and S12). At ~440 kDa, a minor signal of PSMB8 precursor peptides suggested limited incorporation into this assembly intermediate (Figures 4A–4D). Notably, precursor peptide signals for PSMB9 and PSMB10 were also detected at this position, consistent with the presence of immature catalytic subunits at this assembly stage (Figure S13). Comparison of WT and mutant equivalent peptides showed that the abnormal migration pattern was attributable to mutant PSMB8, which was 4-fold reduced in 20S/26S complexes and accumulated at the gel front in the p.Ala235Asp line (Figures 4E and 4F). High-resolution SDS-PAGE confirmed the accumulation of the PSMB8 precursor with reduced mature protein and revealed an additional band slightly above the mature form detected only in mutant samples (Figure 4G). Accordingly, activity-based probing with

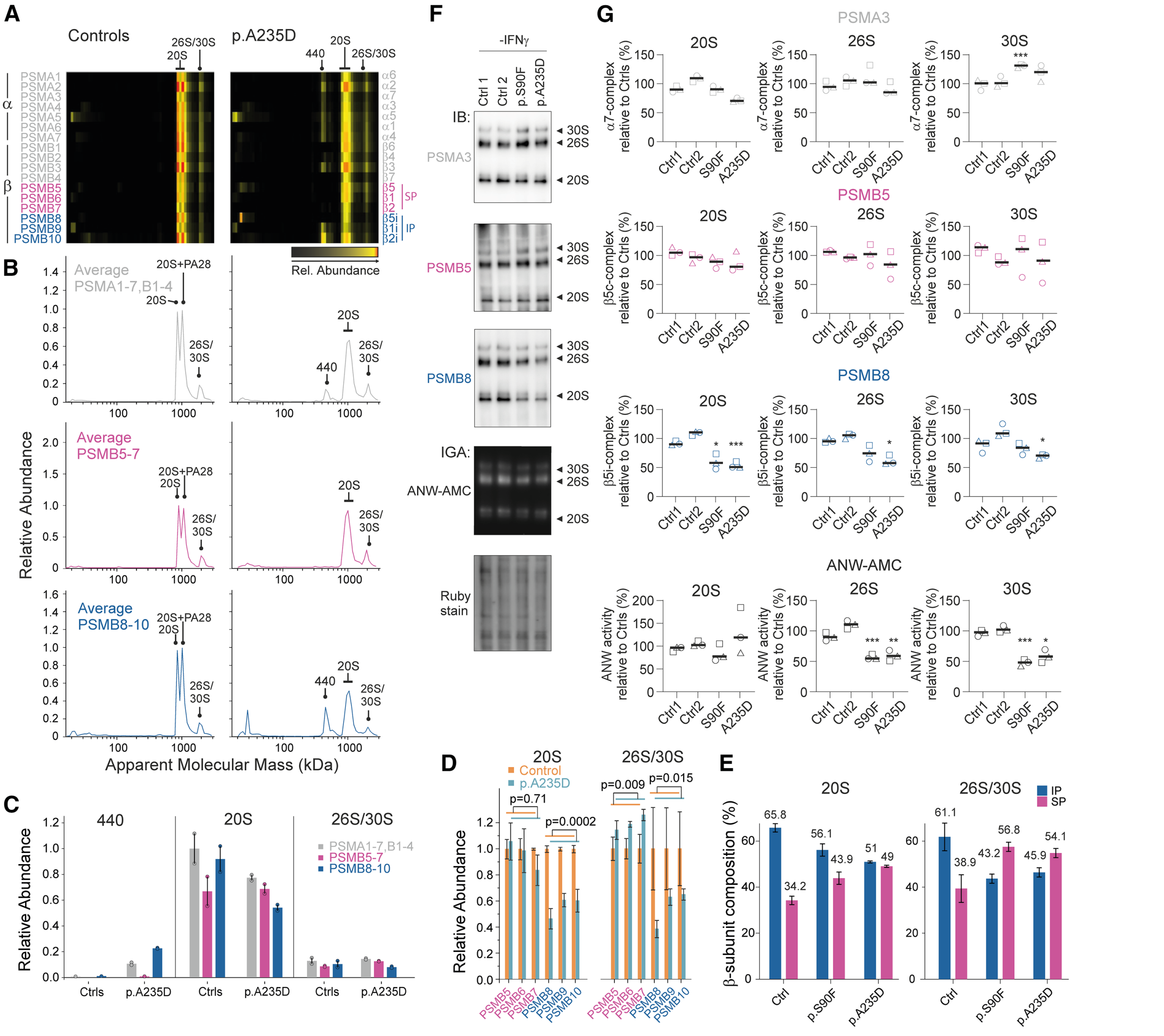

Figure 2. Impaired assembly of immunoproteasomes in fibroblasts carrying *PSMB8* variants

(A) Heatmap representation of the migration profiles of α and β subunits showing signals for 20S and 26S+30S proteasome complexes in control fibroblasts and in fibroblasts carrying variant p.Ala235Asp. An additional signal at ∼440 kDa is observed in the variants. For each individual, two lanes of a blue native gel were analyzed.

(B) Average migration patterns of α and β subunits shared between the SP and IP (gray), SP-specific β subunits (dark pink), and IP-specific β subunits (blue).

(C) Abundance of SP-specific, IP-specific, and shared subunits at the 440-kDa intermediate and 20S and 26S proteasomes* relative to the abundance of the 20S complex in the controls (mean ± standard deviation, $n = 2$).

(D) Relative quantification of SP- and IP-β subunits at 20S and 26S (mean ± standard deviation, $n = 2$).

(E) Percentage of SP and IP content in 20S and 26S proteasome complexes.

(F) Immunoblots (IBs) of PSMA3, PSMB5, and PSMB8 and in-gel activity (IGA), followed by cleavage of ANW-AMC, after native separation of fibroblast lysates. This is a representative image of three independent experiments.

(G) Quantification of the activity and the abundance in 20S, 26S, and 30S complexes of PSMA3, PSMB5, and PSMB8, as representatives of shared and SP- and IP-specific subunits, respectively ($n = 3$). Quantification of ANW-AMC activity at the 20S, 26S, and 30S proteasome complexes was normalized to total proteasome abundance measured as PSMA3 content.

Significance levels are indicated as ns, not significant, *$p \leq 0.05$, **$p \leq 0.01$, and ***$p \leq 0.001$.

Note: quantification of the 20S and 26S+30S proteasomes also includes assemblies with PA28 and PA200. Mass ranges used for quantification were as follows: 440-kDa peak, 403–514 kDa; 20S proteasome, 774–1,262 kDa; and 26S+30S proteasome, 1,898–2,059 kDa.

Cy5-epoxomicin showed a ∼50% reduction in PSMB8-associated catalytic activity, while PSMB5 activity remained unchanged (Figure 4H). To assess the effect of the remaining variants, we transiently expressed them in HEK293T cells, alongside four structurally proximal variants associated with classical PRAAS. WT and variants associated with classical PRAAS were normally assembled, as indicated by the presence of mature PSMB8 and their detection in proteasome complexes, whereas all monoallelic mutants lacked mature PSMB8, which was also absent in the complexes (Figures 4I and 4J).

Collectively, these data support defective incorporation of mutant PSMB8 into assembling proteasomes, leading to accumulation of free precursor subunits, a mechanism

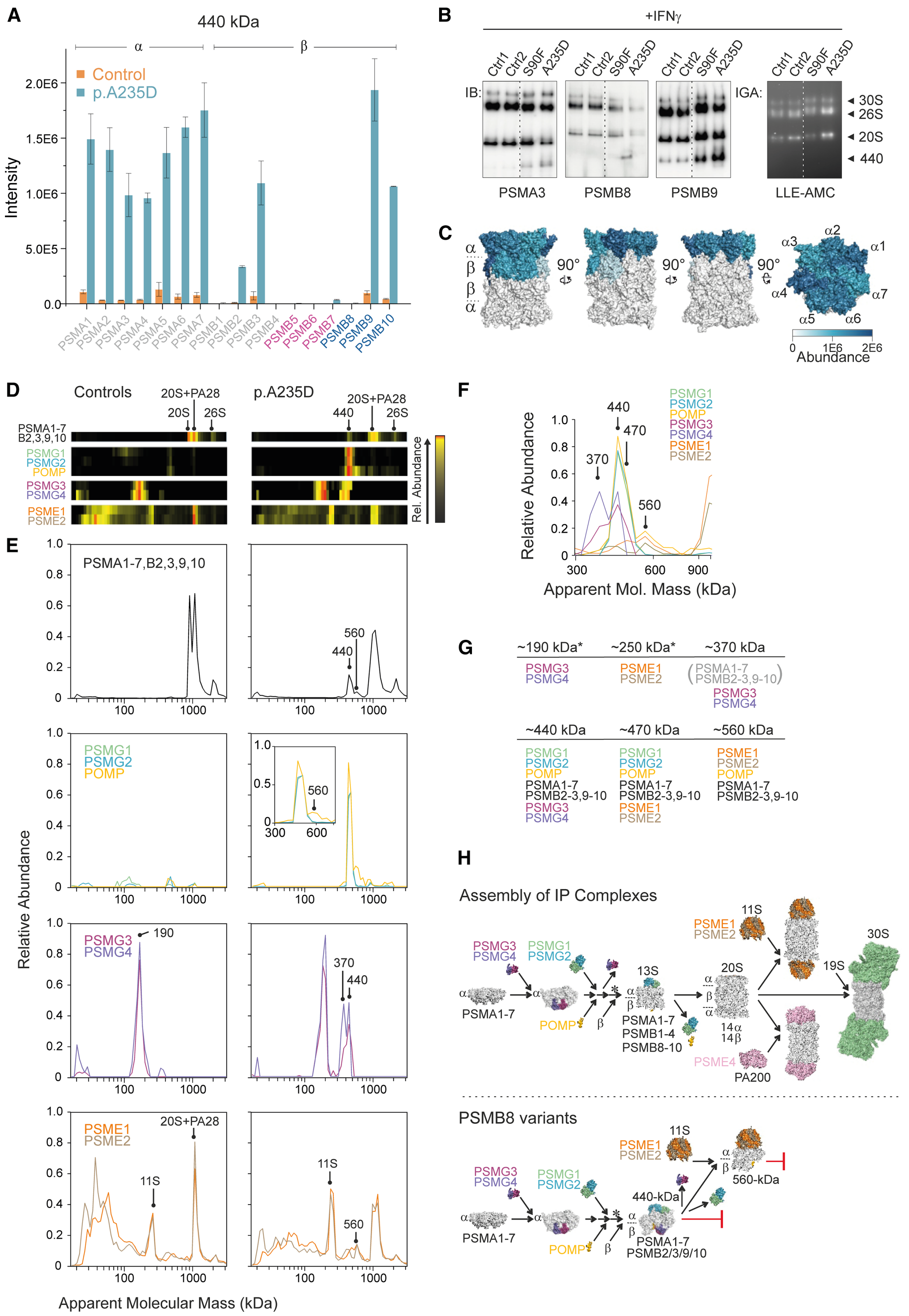

Figure 3. Assembly factors scaffold the accumulated IP intermediate
(A) Quantification of α and β subunits at the ∼440-kDa intermediate.
(B) Immunoblot (IB) of PSMA3, PSMB8, and PSMB9, showing the accumulation of PSMA3 and PSMB9 at ∼440 kDa, and proteasomal in-gel activity (IGA) developed with the β1 and β1i-specific substrate LLE-AMC.
(C) Cartoon representation of the 20S IP based on the cryoelectron microscopy (cryo-EM) structure of the human 20S IP (PDB: 6E5B[32]), where the upper half of the structure, comprising one α and one β ring, is colored by the average protein intensity values observed in the proband-derived samples ($n = 2$).

(legend continued on next page)

distinct from that observed for the tested classical PRAAS-associated variants.

Proteasome assembly factors, inflammatory markers, and ISR-related genes are dysregulated in affected fibroblasts

Differential protein abundance in fibroblasts with the p.Ala235Asp variant revealed a total of 182 significantly upregulated (fold change ≥ 2) and 157 significantly downregulated (fold change ≤ 0.5) proteins (Table S9). Enrichment analysis indicated upregulation of proteasome assembly factors (PSMG1, PSMG2, and POMP) and stress-related pathways, including components of the RNA exosome complex (EXOSC), as well as immune response genes predominantly related to type I/II IFN signaling (Figures 5A and S14; Table S10). Downregulated proteins were enriched for cell motility and adhesion, T cell responses, and the PI3K/AKT pathway. Previous studies have linked cellular stress to activation of the integrated stress response (ISR) through eIF2α phosphorylation mediated by PKR or GCN2 kinases.[30,42,43] In line with this, and consistent with the proteomics findings, significant upregulation of the downstream ISR targets *ATF4* and *DDIT3* was observed by RT-qPCR. In contrast, expression levels of endoplasmic reticulum stress-associated genes, *HSPA5* and *sXBP1*, remained unchanged (Figure 5B).

Variants at aligned positions across *PSMB8*, *PSMB9*, and *PSMB10* may exert similar functional effects

Because *PSMB8*, *PSMB9*, and *PSMB10* originated from paralogous gene copies, we aligned their sequences to compare positions of nine reported monoallelic variants (Table S1).[44] Interestingly, two of the *PSMB8* variants mapped to the same secondary structural positions as described variants in *PSMB9* and *PSMB10*, whereas the remaining variants localized to positions largely conserved among the three subunits (Figures 5C and S15).

To identify features underlying potentially dominant-negative behavior, we assessed structural and biophysical properties of monoallelic variants and compared them with thirteen missense variants associated with classical PRAAS. In general, all pathogenic variants affected evolutionarily conserved residues (Rate4Site score < 0). However, PRAAS-ID-associated variants showed significantly larger predicted energy shifts (ΔΔG), preferential localization to buried sites (RSA < 5%), and disruption of a greater number of contacts showing differences in frustration energies (Δ contacts) (Figure 5D). These features were subsequently evaluated across paralogous variants (equivalent amino acid substitutions at aligned positions) in the 19 subunits constituting the 20S SP and IP. Notably, the structural and evolutionary characteristics were largely conserved across the IP β subunits but substantially weaker across other β and α subunits (Figure S16). Thus, most modeled paralogous variants displayed similar structural and evolutionary properties, suggesting they may have similar functional impacts (Figures 5E and S17; Table S11). However, selected positions deviated from this pattern, including p.Gly190Arg in PSMB9, which is present in gnomAD v.4.1.0, consistent with an expected limited impact on IP assembly. Together, these findings suggest that variants at paralogous positions are susceptible to similar functionally relevant assembly disruption, with structural properties improving predictions of their impact (Figure 5E).

Discussion

Classical PRAAS caused by *PSMB8* variants is inherited in a recessive manner. Here, we expand this observation by describing five different monoallelic variants in seven individuals, presenting with variable degrees of immunodeficiency and multisystemic inflammation. These variants act through a dominant-negative disease mechanism, causing impaired PSMB8 incorporation, abnormal IP biogenesis, accumulation of dysfunctional assembly intermediates, and cellular stress. Our findings are in agreement with a recent publication that independently described a single dominant-negative *PSMB8* variant causing PRAAS.[30]

By modeling the variants into the IP structure, we found that all variants were associated with structural destabilization and altered residue contacts, supporting a shared mechanism of defective proteasome assembly underlying dominant-negative effects in multimeric protein complexes.[45] This prediction was experimentally validated, revealing defective IP biogenesis with reduced abundance

(D) Heatmap representation of the average of α and β subunits that form the ~440-kDa intermediate (PSMA1–7 and PSMB2–3/9–10), proteasome assembly chaperones PSMG1–4 and POMP, and the components of the 11S heptameric complex, PSME1 and PSME2. The average of two independent experiments is shown.
(E) Plots of the average migration profiles of the proteins from (A).
(F) Zoom-in of the protein migration profiles around ~440 kDa showing the stepwise incorporation and release of assembly chaperones.
(G) Composition of the accumulated (sub)complexes observed in control subject- and proband-derived fibroblasts.
(H) Schematic representation of the IP assembly pathway illustrating differences between controls and variants. In the controls, all β subunits are efficiently incorporated into assembly intermediates, leading to the formation of the 20S core particle and, subsequently, fully assembled proteasomes containing regulatory particles. In contrast, in the variants, defective incorporation of PSMB8 and of β subunits that are incorporated later stalls the assembly process. This leads to the accumulation of an aberrant ~440-kDa assembly intermediate that retains the assembly factors. In the variant p.Ala235Asp, a portion of this intermediate progresses to form a ~560-kDa complex, in which the assembly factors, except for POMP, have been replaced with the 11S regulatory particle. This model highlights impaired proteasome biogenesis as a potential pathogenic mechanism.

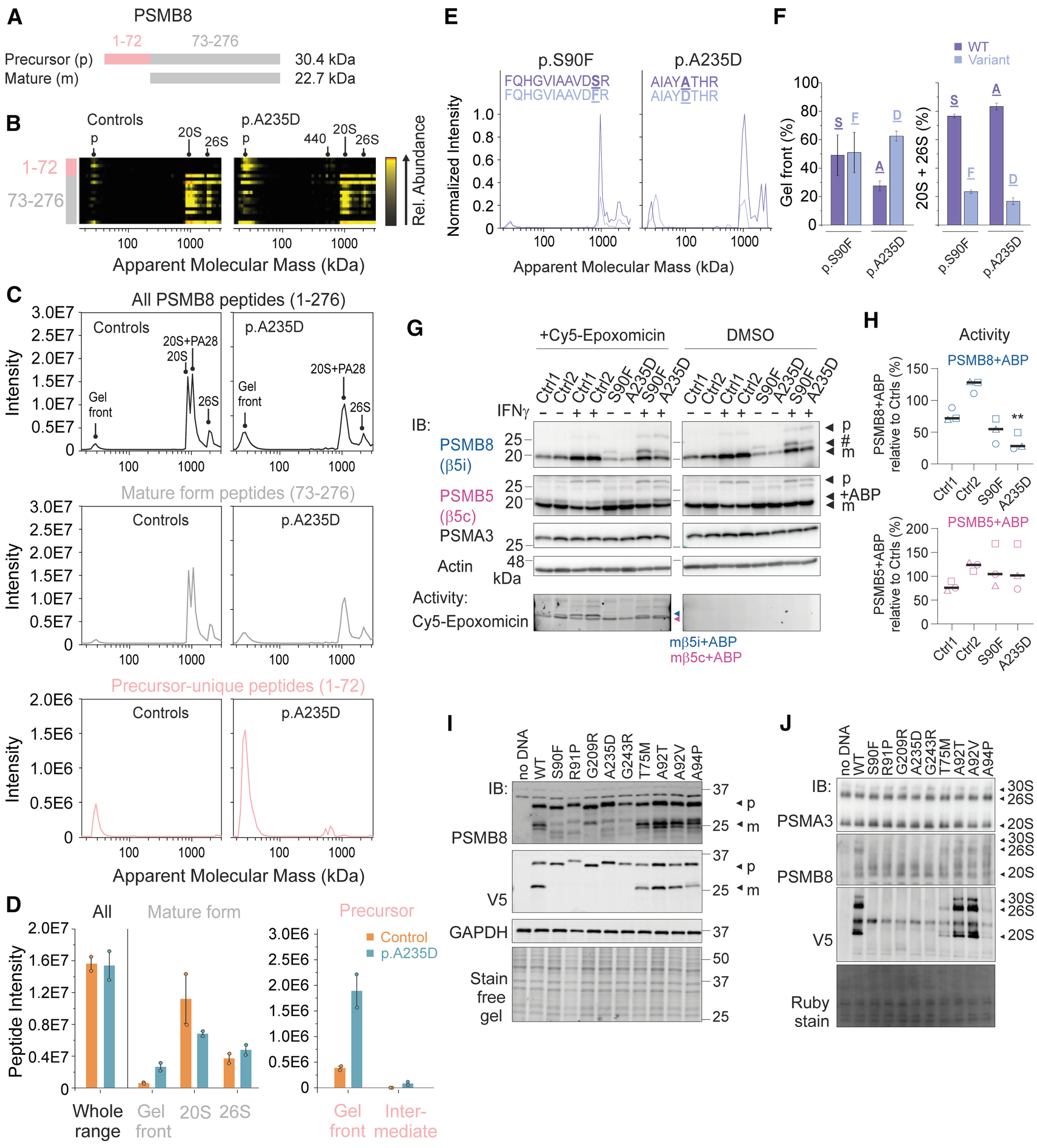

Figure 4. Peptide analysis reveals inefficient maturation and decreased incorporation of p.Alla235Asp variant to 20S and 26S complexes

(A) Schematic representation of PSMB8 cleavage site. Theoretical masses were calculated with the Expasy tool compute pI/Mw.
(B) Heatmap representation of average migration profiles of all PSMB8 peptides identified (Figures S12A and S12B).
(C) Average migration profiles based on the intensity values of all peptides (top), peptides of the mature form (middle), or precursor-specific peptides (bottom).
(D) Quantification of all peptides in the whole molecular mass range, mature-form peptide intensities across the gel front and 20S and 26S proteasome fractions, and of precursor-unique peptides at the gel front and at the 440-kDa intermediate.
(E) Quantification of wild-type and mutant-specific peptide intensities in controls and in fibroblasts carrying variants p.Ser90Phe and p.Ala235Asp.
(F) Percentage of free (migrating at the gel front) and assembled wild-type and mutant PSMB8 into 20S+26S complexes.
(G) Immunoblot (IB) analysis of fibroblasts with or without IFNγ stimulation using antibodies against PSMB8, PSMB5, PSMA3, and actin and proteasomal subunit activity. #, this band may reflect an incomplete maturation step.
(H) Quantification of PSMB8 and PSMB5 specific activity in samples without IFNγ stimulation.
(I) Immunoblot of PSMB8, V5-tag, and GAPDH in HEK293T cells transiently expressing *PSMB8* variants associated with PRAAS-ID and classical PRAAS.
(J) Native immunoblot analysis of transfected HEK293T cells from (I), using antibodies against PSMA3, PSMB8, and V5-tag. Data represent the average of two independent experiments.
Significance levels are indicated as ns, not significant, *$p \leq 0.05$, **$p \leq 0.01$, and ***$p \leq 0.001$.

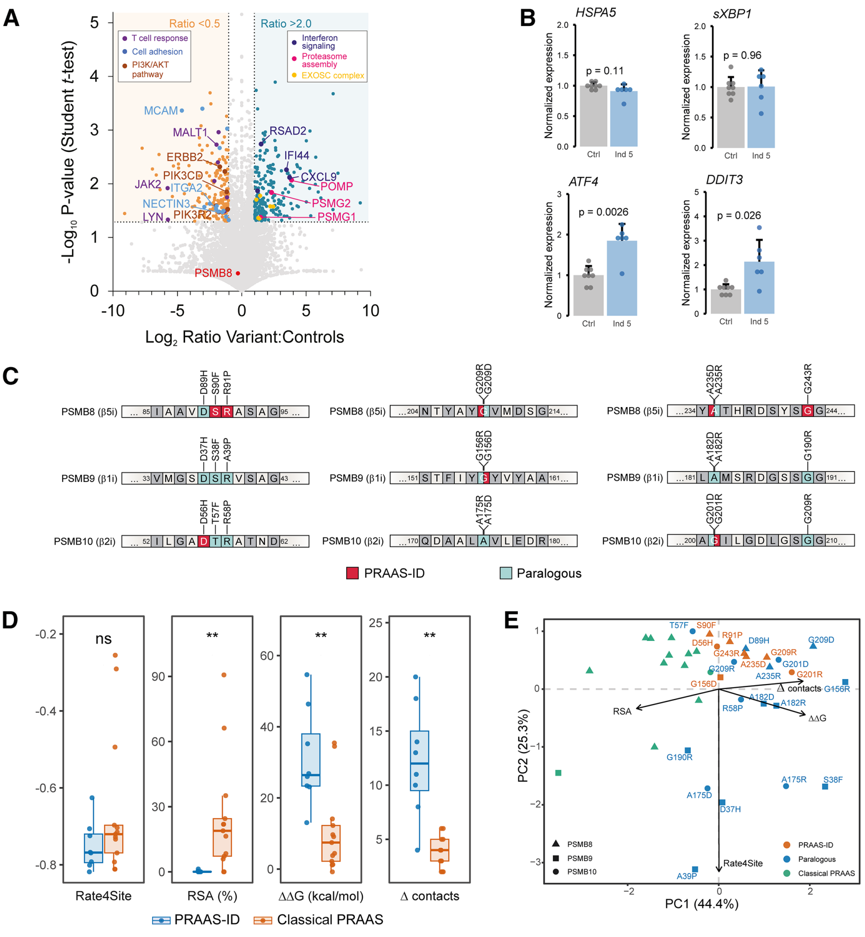

Figure 5. Expression analysis and *in silico* characterization of *PSMB8*, *PSMB9*, and *PSMB10* variants

(A) Volcano plot of differential protein abundance in fibroblast expressing the p.Ser90Phe and p.Ala235Asp variants compared with controls ($\log_2$ fold change versus $-\log_{10}$ Student's t test p value). Dashed vertical lines indicate expression ratios <0.33 and >3.0. Selected significantly dysregulated genes are highlighted and colored by functional category.

(B) RT-qPCR analysis of integrated stress response (ISR) and endoplasmic reticulum stress markers (*HSPA5*, *sXBP1*, *ATF4*, and *DDIT3*) in IFNγ-stimulated fibroblasts from control subjects and individual 5 (Ind 5). Bars show the mean ± SD; dots represent individual measurements. p values are shown (two-tailed t test).

(C) Sequence context of PRAAS-ID-associated variants and evaluated paralogous variants (equivalent amino acid substitutions at aligned positions) in *PSMB8*, *PSMB9*, and *PSMB10*.

(D) Comparison of *in silico* scores between PRAAS-ID-associated and classical PRAAS-associated variants for Rate4Site (evolutionary conservation), ΔΔG (predicted destabilization), RSA (relative solvent accessibility), and Δ contacts (number of contacts with changed frustration energies).

(E) Principal-component analysis (PCA) of structural and evolutionary features of PRAAS-ID and classical PRAAS variants and PRAAS-ID-derived paralogous variants. Symbols indicate the proteasome subunit, and colors indicate variant class. Arrows represent the contribution of structural and evolutionary variables (RSA, Δ contacts, ΔΔG, and Rate4Site).

Significance levels are indicated as ns, not significant, $*p \leq 0.05$, $**p \leq 0.01$, and $***p \leq 0.001$.

of fully assembled complexes and impaired maturation characterized by accumulation of stalled assembly intermediates. The primary defect appears to be inefficient incorporation of mutant PSMB8, leading to accumulation of unincorporated protein. Residual incorporation of mutant subunits may further perturb assembly by generating complexes that fail to mature properly or that retain compromised proteasome function. For example, the p.Ser90Phe variant is predicted to perturb the interaction network near the catalytic center and may therefore additionally affect catalytic activity. Several additional observations were consistent with current models of IP assembly. First, the reduction of fully assembled complexes was accompanied by a coordinated decrease of all three IP catalytic subunits, supporting the cooperative incorporation model favoring homogeneous IP formation over mixed complexes.[15,46–48] Second, the composition of the stalled assembly intermediate aligned with the reported sequential order of IP subunit incorporation. This intermediate was also detectable at low levels in controls, indicating a transient physiological assembly step that remains catalytically inactive with uncleaved precursor forms of PSMB9 and PSMB10.[6,47] Finally, compensatory SP upregulation, typically observed in knockout models, was not detected despite impaired IP assembly.[46,49,50] This likely reflects preserved PSMB8 expression, with the defect resulting from impaired subunit incorporation rather than its absence.

The mechanism we observed here parallels that proposed for reported monoallelic variants in genes encoding the IP catalytic subunits PSMB8, PSMB9, and PSMB10 and the proteasome assembly factor POMP.[27–30] Moreover, assembly defects have been independently demonstrated for the p.Gly209Arg variant in PSMB8 and the p.Gly156Asp variant in PSMB9, which similarly impair incorporation of the catalytic subunits and lead to accumulation of abnormal assembly intermediates.[28,30] More broadly, reported monoallelic variants in these catalytic subunits are missense changes that predominantly cluster at the β ring interface and share similar predicted structural consequences, including destabilization and perturbation of residue interactions. Several variants affect equivalent residues across these paralogous proteins, supporting observations that variants at paralogous positions can support the pathogenicity of newly identified variants.[51,52] However, some paralogous substitutions showed

milder or divergent predicted structural effects, indicating that computational analyses can help refine variant interpretation and guide prioritization for experimental validation. In contrast, classical PRAAS-associated variants were predicted to induce more localized effects, consistent with the normal incorporation of the mutant proteins observed in our transfection assays. Finally, variants affecting *POMP* represent a related but mechanistically broader category. These variants are frameshifts that escape nonsense-mediated decay, resulting in the production of a truncated protein that disrupts both SP and IP assembly pathways.[27] Taken together, monoallelic variants affecting either catalytic subunits or *POMP* likely act through a shared dominant-negative mechanism by interfering with proteasome assembly.

Clinically, the seven individuals show substantial overlap with PRAAS-ID caused by monoallelic variants in *PSMB8*, *PSMB9*, or *PSMB10*, characterized by neonatal-onset immunodeficiency with recurrent infections and systemic inflammation. In contrast, lipodystrophy or joint abnormalities typical of recessive forms of PRAAS are generally absent (Table 2).[28–30,53,54] The postmortem basal ganglia calcification in individuals 2 and 3 and the elevated IFN signatures and/or increased serum IFNγ levels in all tested individuals suggested IFN pathway activation, as observed in PRAAS, although less prominent and without clear accumulation of polyubiquitinated substrates.[55] Immunodeficiency, particularly B cell lymphopenia and hypogammaglobulinemia, is consistent across all monoallelic forms.[28–30] In contrast to reports of T cell abnormalities in individuals with monoallelic variants in *PSMB9* and *PSMB10*, T cell populations and mitogen responses were normal in our affected individuals. This difference may in part reflect the distinct composition of the thymoproteasome, in which PSMB8 is replaced by PSMB11 (β5t), the catalytic subunit mediating $CD8^+$ T cell selection, whereas PSMB9 and PSMB10 are components of both the IP and the thymoproteasome.[56,57] However, T cell lymphopenia and hampered function have also been described for the p.Gly209Arg monoallelic *PSMB8* variant, suggesting a thymus-independent mechanism. The recurrent viral infections in several individuals in our study might also indicate some degree of impaired T cell function. Additional studies are needed to further delineate specific lymphocyte effects. T and B cell abnormalities have also been described in individuals with variants in the proteasome assembly factor *POMP*, who additionally exhibit signs of immune dysregulation such as circulating autoantibodies and hypergammaglobulinemia.[27]

Of interest are the leukocyte vacuolization and inclusions seen in the blood and/or bone marrow of individuals 1 and 4. These inclusions might reflect accumulation of protein aggregates due to increased ISR activation and proteotoxic stress, reminiscent of lysosomal storage disorders and perhaps similar to the ubiquitin-rich inclusions observed in keratinocytes from individuals with PRAAS.[24] More studies are needed to unravel the origin and relevance of these leukocyte inclusions in individuals with monoallelic *PSMB8* variants. Nevertheless, vacuolization and the presence of inclusions in leukocytes could be sought as a supportive diagnostic feature.

Our results underscore the added value of complexome profiling relative to conventional methods in the evaluation of the assembly process. Complexome profiling provides high-resolution and comprehensive detail, enabling precise visualization of proteasome assembly intermediates, stoichiometric relationships between subunits, and variant-specific disruptions, especially relevant for assessing the clinical significance of missense variants. Moreover, our findings emphasize that alternative inheritance patterns should be considered when missense variants occur in components of multiprotein complexes, as dominant-negative effects may be involved.

To conclude, we describe seven individuals carrying monoallelic *PSMB8* variants that impair IP assembly, leading to clinically variable immunodeficiency and inflammatory disease, with leukocyte vacuolization and inclusions observed in some individuals. The overlap between clinical features of PRAAS-ID and the shared structural and functional effects caused by monoallelic variants supports IP assembly disruption as a unifying dominant-negative mechanism underlying this group of disorders.

Data and code availability

The MS data from IFNγ-stimulated fibroblasts are available at the ProteomeXchange Consortium via the PRIDE partner repository. The accession number for the MS data reported in this paper is PRIDE:PXD064505 (https://www.ebi.ac.uk/pride/archive). The complexome profiling dataset is available at the Complexome Profiling Data Resource (CEDAR). The accession number for the complexome profiling data reported in this paper is CEDAR:CRX49 (https://www3.cmbi.umcn.nl/cedar/browse).

Acknowledgments

The members of the Undiagnosed Diseases Network are listed in supplemental information. We thank the families for participating and for allowing this study to be conducted. We would like to thank Wim A. Dik, Sandra J. Posthumus-van Sluijs, Esther van Rijssen, Evelien Sprenkeler, Laura Lubbers, Laura Batlle Masó, and the Core Facility Mass Spectrometric Proteomics as part of the Technology Platform Mass Spectrometry (TPMS) at the University of Hamburg (UHH) and University Medical Center Hamburg-Eppendorf (UKE) for technical assistance. S.B.W. is a member of the European Reference Network for Rare Hereditary Metabolic Disorders (MetabERN). This work was supported by MetaKids and the United for Metabolic Diseases consortium (UMD-ZOE-2022-012); the Deutsche Forschungsgemeinschaft (DFG; INST 337/15-1, INST 337/16-1, INST 152/837-1, INST 152/947-1, FOR 5705, 523862973, and 518551069); the BMFTR (German Federal Ministry of Research, Technology, and Space) through the German Center for Child and Adolescent Health (DZKJ) (01GL2404A); the Ricerca Corrente Ministeriale (PerSAIDs project; ERAPerMed2021-262); the National Institute of Neurological

Table 2. Comparison of clinical features between monoallelic *PSMB8*-related disorder and other PRAAS syndromes

**Disease**	**Monoallelic**					**Biallelic, oligogenic**	
**Gene(s)**	***PSMB8***	***PSMB8***	***PSMB9***	***PSMB10***	***POMP***	***PSMB8***	***PSMB8, PSMB10, PSMB4, PSMG2, PSMA3, PSMA5, PSMC5***
Ref.	this study	Wolfgramm et al.[30]	Kanazawa et al.,[28] Kataoka et al.[53]	van der Made et al.[29]	Brehm et al.,[24] Poli et al.,[27] Meinhardt et al.[58]	Papendorf et al.,[25] Arima et al.,[59] Liu et al.,[60] Patel et al.[61]	de Jesus et al.,[22] Brehm et al.,[24] Papendorf et al.,[25] Arima et al.,[59] Liu et al.,[60] Verhoeven et al.[62]
Total individuals	7	8	3	6	4	20	13
**Clinical features**							
Growth deficiency	+	+	N/R	+	+/−	+	+
Recurrent fever	+	+	+	−	+/−	+	+
Skin rash	+	+	+	+	+	+	+
Systemic inflammation	+/−	+	+	+/−	+/−	+	+
Inflammatory lung disease	+	+/−	+	+/−	+/−	+/−	+/−
Pulmonary hypertension	+/−	N/R	+	−	N/R	N/R	N/R
Enteropathy	+	+/−	N/R	+	+/−	N/R	N/R
Liver dysfunction	+	+	+	+	−	+/−	+
Myositis/muscle atrophy	+	+/−	+	−	−	+/−	+
Rhabdomyolysis	+/−	N/R	+	−	−	+/−	+/−
Basal ganglia calcification	+/−	N/R	+	−	N/R	+/−	+/−
Lipodystrophy	−	+/−	−	−	+/−	+	+
Joint contractures	−	N/R	N/R	−	−	+/−	+
**Laboratory results**							
Dyslipidemia	+	+/−	−	N/R	N/R	+	+
Autoantibodies	−	N/R	−	N/R	+	+/−	+/−
Immunoglobulins	↓	↓	↓	↓	↑	↑ (most)	↑ (most), ↓ (some)
Anemia	+	+	+	N/R	+	+	+
Thrombocytopenia	+	+	+	N/R	+	+ (some ↑)	+ (some ↑)
B cells	↓	↓	↓ or normal	↓	↓	normal or N/R	normal or N/R
T cells	normal	↓ CD4 or normal	↓ or normal	↓, ↑ CD4/CD8	↑ CD4, ↑ CD4/CD8	normal or N/R	normal or N/R

(*Continued on next page*)

Table 2. *Continued*

Disease	Monoallelic					Biallelic, oligogenic	
**Gene(s)**	***PSMB8***	***PSMB8***	***PSMB9***	***PSMB10***	***POMP***	***PSMB8***	***PSMB8, PSMB10, PSMB4, PSMG2, PSMA3, PSMA5, PSMC5***
NK cells	variable	N/R	variable	variable	↓	normal or N/R	normal or N/R
IFN signature	↑	↑	↑	↑	↑	↑	↑
Other	hypothyroidism, eosinophilia	thyroid gland agenesis, eosinophilia, nephropathy	abnormal coagulation	eosinophilia	eosinophilia	low TSH	–

+, observed in most individuals; +/−, observed in some individuals; −, not observed; ↓, decreased; ↑, increased; N/R, not reported; normal, within reference range; TSH, thyroid-stimulating hormone.

Disorders and Stroke of the National Institutes of Health (U01HG007709 and U01HG007942); and the Clinical Translational Core of the Baylor College of Medicine IDDRC (P50HD103555) from the Eunice Kennedy Shriver National Institute of Child Health and Human Development. The content is solely the responsibility of the authors and does not necessarily represent the official views of the National Institutes of Health.

Author contributions

R.W., C.I.v.d.M., S.B.W., C.M.-S., R.A.W., M.M.O., and S.G.-C. contributed to the conception and design of the study. R.W., M.W.-H., E.J.A., A.G., I.C., Z.S., and M.M.O. performed the genetic data analysis. R.W., C.I.v.d.M., S.K.U., J.A.R., T.P.V., S.K.N., E.J.A., T.E.G., S.V., S.P.K., R.K.A., J.C., and E.D. followed up with the families and collected the clinical data and images. R.W., J.B., M.W., T.P.V., A.C.-O., B.S., T.M., I.G., K.K., R.v.B., and S.G.-C. performed the experimental work. G.R. performed structural biology analysis. R.L.S. performed the cytokine analysis. C.D.M.v.K., M.G., and L.E.L.M.V. provided funding. S.V., M.G., A.H., H.S., S.W.G., L.E.L.M.V., S.B.W., C.M.-S., R.A.W., and M.M.O. provided supervision, either of the study as a whole or of specific aspects such as experiments, clinical follow-up, or data analysis. R.W. and S.G.-C. wrote the initial draft of the manuscript. S.B.W., R.A.W., M.M.O., and S.G.-C. provided critical feedback and assisted with manuscript revisions. All authors read and approved the final version of the manuscript.

Declaration of interests

The Department of Molecular and Human Genetics at Baylor College of Medicine receives revenue from clinical genetic testing completed at Baylor Genetics Laboratories.

Supplemental information

Supplemental information can be found online at https://doi.org/10.1016/j.ajhg.2026.04.015.

Web resources

CEDAR, https://www3.cmbi.umcn.nl/cedar/browse
GenBank, https://www.ncbi.nlm.nih.gov/genbank/
OMIM, https://www.omim.org
PDB, https://www.rcsb.org
PRIDE, https://www.ebi.ac.uk/pride/archive

Received: October 13, 2025
Accepted: April 30, 2026
Published: May 21, 2026

References

1. Rousseau, A., and Bertolotti, A. (2018). Regulation of proteasome assembly and activity in health and disease. Nat. Rev. Mol. Cell Biol. *19*, 697–712. https://doi.org/10.1038/s41580-018-0040-z.
2. Kaneko, T., Hamazaki, J., Iemura, S.I., Sasaki, K., Furuyama, K., Natsume, T., Tanaka, K., and Murata, S. (2009). Assembly pathway of the Mammalian proteasome base subcomplex is

mediated by multiple specific chaperones. Cell *137*, 914–925. https://doi.org/10.1016/j.cell.2009.05.008.

3. Murata, S., Yashiroda, H., and Tanaka, K. (2009). Molecular mechanisms of proteasome assembly. Nat. Rev. Mol. Cell Biol. *10*, 104–115. https://doi.org/10.1038/nrm2630.
4. Tanaka, K. (2009). The proteasome: overview of structure and functions. Proc. Jpn. Acad. Ser. B Phys. Biol. Sci. *85*, 12–36. https://doi.org/10.2183/pjab.85.12.
5. Arendt, C.S., and Hochstrasser, M. (1997). Identification of the yeast 20S proteasome catalytic centers and subunit interactions required for active-site formation. Proc. Natl. Acad. Sci. USA *94*, 7156–7161. https://doi.org/10.1073/pnas.94.14.7156.
6. Ferrington, D.A., and Gregerson, D.S. (2012). Immunoproteasomes: structure, function, and antigen presentation. Prog. Mol. Biol. Transl. Sci. *109*, 75–112. https://doi.org/10.1016/B978-0-12-397863-9.00003-1.
7. Heink, S., Ludwig, D., Kloetzel, P.M., and Krüger, E. (2005). IFN-gamma-induced immune adaptation of the proteasome system is an accelerated and transient response. Proc. Natl. Acad. Sci. USA *102*, 9241–9246. https://doi.org/10.1073/pnas.0501711102.
8. Murata, S., Takahama, Y., Kasahara, M., and Tanaka, K. (2018). The immunoproteasome and thymoproteasome: functions, evolution and human disease. Nat. Immunol. *19*, 923–931. https://doi.org/10.1038/s41590-018-0186-z.
9. Kincaid, E.Z., Che, J.W., York, I., Escobar, H., Reyes-Vargas, E., Delgado, J.C., Welsh, R.M., Karow, M.L., Murphy, A.J., Valenzuela, D.M., et al. (2011). Mice completely lacking immunoproteasomes show major changes in antigen presentation. Nat. Immunol. *13*, 129–135. https://doi.org/10.1038/ni.2203.
10. Rock, K.L., and Goldberg, A.L. (1999). Degradation of cell proteins and the generation of MHC class I-presented peptides. Annu. Rev. Immunol. *17*, 739–779. https://doi.org/10.1146/annurev.immunol.17.1.739.
11. Sijts, E.J.A.M., and Kloetzel, P.M. (2011). The role of the proteasome in the generation of MHC class I ligands and immune responses. Cell. Mol. Life Sci. *68*, 1491–1502. https://doi.org/10.1007/s00018-011-0657-y.
12. Abi Habib, J., Lesenfants, J., Vigneron, N., and Van den Eynde, B.J. (2022). Functional Differences between Proteasome Subtypes. Cells *11*, 421. https://doi.org/10.3390/cells11030421.
13. Adolf, F., Du, J., Goodall, E.A., Walsh, R.M., Jr., Rawson, S., von Gronau, S., Harper, J.W., Hanna, J., and Schulman, B.A. (2024). Visualizing chaperone-mediated multistep assembly of the human 20S proteasome. Nat. Struct. Mol. Biol. *31*, 1176–1188. https://doi.org/10.1038/s41594-024-01268-9.
14. Budenholzer, L., Cheng, C.L., Li, Y., and Hochstrasser, M. (2017). Proteasome Structure and Assembly. J. Mol. Biol. *429*, 3500–3524. https://doi.org/10.1016/j.jmb.2017.05.027.
15. Watanabe, A., Yashiroda, H., Ishihara, S., Lo, M., and Murata, S. (2022). The Molecular Mechanisms Governing the Assembly of the Immuno- and Thymoproteasomes in the Presence of Constitutive Proteasomes. Cells *11*, 1580. https://doi.org/10.3390/cells11091580.
16. Nandi, D., Woodward, E., Ginsburg, D.B., and Monaco, J.J. (1997). Intermediates in the formation of mouse 20S proteasomes: implications for the assembly of precursor beta subunits. EMBO J. *16*, 5363–5375. https://doi.org/10.1093/emboj/16.17.5363.
17. Guillaume, B., Chapiro, J., Stroobant, V., Colau, D., Van Holle, B., Parvizi, G., Bousquet-Dubouch, M.P., Théate, I., Parmentier, N., and Van den Eynde, B.J. (2010). Two abundant proteasome subtypes that uniquely process some antigens presented by HLA class I molecules. Proc. Natl. Acad. Sci. USA *107*, 18599–18604. https://doi.org/10.1073/pnas.1009778107.
18. Klare, N., Seeger, M., Janek, K., Jungblut, P.R., and Dahlmann, B. (2007). Intermediate-type 20 S proteasomes in HeLa cells: “asymmetric” subunit composition, diversity and adaptation. J. Mol. Biol. *373*, 1–10. https://doi.org/10.1016/j.jmb.2007.07.038.
19. Mendonça, L.O., and Frémond, M.L. (2024). Interferonopathies: From concept to clinical practice. Best Pract. Res. Clin. Rheumatol. *38*, 101975. https://doi.org/10.1016/j.berh.2024.101975.
20. de Jesus, A.A., Hou, Y., Brooks, S., Malle, L., Biancotto, A., Huang, Y., Calvo, K.R., Marrero, B., Moir, S., Oler, A.J., et al. (2020). Distinct interferon signatures and cytokine patterns define additional systemic autoinflammatory diseases. J. Clin. Investig. *130*, 1669–1682. https://doi.org/10.1172/JCI129301.
21. Agarwal, A.K., Xing, C., DeMartino, G.N., Mizrachi, D., Hernandez, M.D., Sousa, A.B., Martínez de Villarreal, L., dos Santos, H.G., and Garg, A. (2010). PSMB8 encoding the beta5i proteasome subunit is mutated in joint contractures, muscle atrophy, microcytic anemia, and panniculitis-induced lipodystrophy syndrome. Am. J. Hum. Genet. *87*, 866–872. https://doi.org/10.1016/j.ajhg.2010.10.031.
22. de Jesus, A.A., Brehm, A., VanTries, R., Pillet, P., Parentelli, A.S., Montealegre Sanchez, G.A., Deng, Z., Paut, I.K., Goldbach-Mansky, R., and Krüger, E. (2019). Novel proteasome assembly chaperone mutations in PSMG2/PAC2 cause the autoinflammatory interferonopathy CANDLE/PRAAS4. J. Allergy Clin. Immunol. *143*, 1939–1943.e8. https://doi.org/10.1016/j.jaci.2018.12.1012.
23. Sarrabay, G., Méchin, D., Salhi, A., Boursier, G., Rittore, C., Crow, Y., Rice, G., Tran, T.A., Cezar, R., Duffy, D., et al. (2020). PSMB10, the last immunoproteasome gene missing for PRAAS. J. Allergy Clin. Immunol. *145*, 1015–1017.e6. https://doi.org/10.1016/j.jaci.2019.11.024.
24. Brehm, A., Liu, Y., Sheikh, A., Marrero, B., Omoyinmi, E., Zhou, Q., Montealegre, G., Biancotto, A., Reinhardt, A., Almeida de Jesus, A., et al. (2015). Additive loss-of-function proteasome subunit mutations in CANDLE/PRAAS patients promote type I IFN production. J. Clin. Investig. *125*, 4196–4211. https://doi.org/10.1172/JCI81260.
25. Papendorf, J.J., Ebstein, F., Alehashemi, S., Piotto, D.G.P., Kozlova, A., Terreri, M.T., Shcherbina, A., Rastegar, A., Rodrigues, M., Pereira, R., et al. (2023). Identification of eight novel proteasome variants in five unrelated cases of proteasome-associated autoinflammatory syndromes (PRAAS). Front. Immunol. *14*, 1190104. https://doi.org/10.3389/fimmu.2023.1190104.
26. Méchin, D., Tusseau, M., Broly, M., Sanlaville, D., Viel, S., Sarrabay, G., Belot, A., Forestier, E., Cottet, R., and Boursier, G. (2026). A new digenic inheritance of proteasome-associated autoinflammatory syndrome involving the PSMA6 gene. J. Allergy Clin. Immunol. Pract. *14*, 524–526.e1. https://doi.org/10.1016/j.jaip.2025.10.039.

27. Poli, M.C., Ebstein, F., Nicholas, S.K., de Guzman, M.M., Forbes, L.R., Chinn, I.K., Mace, E.M., Vogel, T.P., Carisey, A.F., Benavides, F., et al. (2018). Heterozygous Truncating Variants in POMP Escape Nonsense-Mediated Decay and Cause a Unique Immune Dysregulatory Syndrome. Am. J. Hum. Genet. *102*, 1126–1142. https://doi.org/10.1016/j.ajhg.2018.04.010.
28. Kanazawa, N., Hemmi, H., Kinjo, N., Ohnishi, H., Hamazaki, J., Mishima, H., Kinoshita, A., Mizushima, T., Hamada, S., Hamada, K., et al. (2021). Heterozygous missense variant of the proteasome subunit beta-type 9 causes neonatal-onset autoinflammation and immunodeficiency. Nat. Commun. *12*, 6819. https://doi.org/10.1038/s41467-021-27085-y.
29. van der Made, C.I., Kersten, S., Chorin, O., Engelhardt, K.R., Ramakrishnan, G., Griffin, H., Schim van der Loeff, I., Venselaar, H., Rothschild, A.R., Segev, M., et al. (2024). Expanding the PRAAS spectrum: De novo mutations of immunoproteasome subunit beta-type 10 in six infants with SCID-Omenn syndrome. Am. J. Hum. Genet. *111*, 791–804. https://doi.org/10.1016/j.ajhg.2024.02.013.
30. Wolfgramm, S., Alehashemi, S., Wendlandt, M., Thiel, F.G., de Jesus, A.A., Papendorf, J.J., Wolfgramm, H., Alvarez, F.L., Borngräber, E., Uss, K., et al. (2026). A de novo dominant-negative PSMB8 mutation causes severe CANDLE/PRAAS due to arrested proteasome biogenesis. Ann. Rheum. Dis. *85*, 715–729. https://doi.org/10.1016/j.ard.2025.10.021.
31. Sobreira, N., Schiettecatte, F., Valle, D., and Hamosh, A. (2015). GeneMatcher: a matching tool for connecting investigators with an interest in the same gene. Hum. Mutat. *36*, 928–930. https://doi.org/10.1002/humu.22844.
32. Ladi, E., Everett, C., Stivala, C.E., Daniels, B.E., Durk, M.R., Harris, S.F., Huestis, M.P., Purkey, H.E., Staben, S.T., Augustin, M., et al. (2019). Design and Evaluation of Highly Selective Human Immunoproteasome Inhibitors Reveal a Compensatory Process That Preserves Immune Cell Viability. J. Med. Chem. *62*, 7032–7041. https://doi.org/10.1021/acs.jmedchem.9b00509.
33. Dong, Y., Zhang, S., Wu, Z., Li, X., Wang, W.L., Zhu, Y., Stoilova-McPhie, S., Lu, Y., Finley, D., and Mao, Y. (2019). Cryo-EM structures and dynamics of substrate-engaged human 26S proteasome. Nature *565*, 49–55. https://doi.org/10.1038/s41586-018-0736-4.
34. Schymkowitz, J., Borg, J., Stricher, F., Nys, R., Rousseau, F., and Serrano, L. (2005). The FoldX web server: an online force field. Nucleic Acids Res. *33*, W382–W388. https://doi.org/10.1093/nar/gki387.
35. Parra, R.G., Schafer, N.P., Radusky, L.G., Tsai, M.Y., Guzovsky, A.B., Wolynes, P.G., and Ferreiro, D.U. (2016). Protein Frustratometer 2: a tool to localize energetic frustration in protein molecules, now with electrostatics. Nucleic Acids Res. *44*, W356–W360. https://doi.org/10.1093/nar/gkw304.
36. Pupko, T., Bell, R.E., Mayrose, I., Glaser, F., and Ben-Tal, N. (2002). Rate4Site: an algorithmic tool for the identification of functional regions in proteins by surface mapping of evolutionary determinants within their homologues. Bioinformatics *18*, S71–S77. https://doi.org/10.1093/bioinformatics/18.suppl_1.s71.
37. Mitternacht, S. (2016). FreeSASA: An open source C library for solvent accessible surface area calculations. F1000Res. *5*, 189. https://doi.org/10.12688/f1000research.7931.1.
38. Wessels, H.J.C.T., Vogel, R.O., van den Heuvel, L., Smeitink, J.A., Rodenburg, R.J., Nijtmans, L.G., and Farhoud, M.H. (2009). LC-MS/MS as an alternative for SDS-PAGE in blue native analysis of protein complexes. Proteomics *9*, 4221–4228. https://doi.org/10.1002/pmic.200900157.
39. Cabrera-Orefice, A., Potter, A., Evers, F., Hevler, J.F., and Guerrero-Castillo, S. (2021). Complexome Profiling-Exploring Mitochondrial Protein Complexes in Health and Disease. Front. Cell Dev. Biol. *9*, 796128. https://doi.org/10.3389/fcell.2021.796128.
40. Richards, S., Aziz, N., Bale, S., Bick, D., Das, S., Gastier-Foster, J., Grody, W.W., Hegde, M., Lyon, E., Spector, E., et al. (2015). Standards and guidelines for the interpretation of sequence variants: a joint consensus recommendation of the American College of Medical Genetics and Genomics and the Association for Molecular Pathology. Genet. Med. *17*, 405–424. https://doi.org/10.1038/gim.2015.30.
41. Ashkenazy, H., Abadi, S., Martz, E., Chay, O., Mayrose, I., Pupko, T., and Ben-Tal, N. (2016). ConSurf 2016: an improved methodology to estimate and visualize evolutionary conservation in macromolecules. Nucleic Acids Res. *44*, W344–W350. https://doi.org/10.1093/nar/gkw408.
42. Davidson, S., Yu, C.H., Steiner, A., Ebstein, F., Baker, P.J., Jarur-Chamy, V., Hrovat Schaale, K., Laohamonthonkul, P., Kong, K., Calleja, D.J., et al. (2022). Protein kinase R is an innate immune sensor of proteotoxic stress via accumulation of cytoplasmic IL-24. Sci. Immunol. *7*, eabi6763. https://doi.org/10.1126/sciimmunol.abi6763.
43. Ebstein, F., Poli Harlowe, M.C., Studencka-Turski, M., and Krüger, E. (2019). Contribution of the Unfolded Protein Response (UPR) to the Pathogenesis of Proteasome-Associated Autoinflammatory Syndromes (PRAAS). Front. Immunol. *10*, 2756. https://doi.org/10.3389/fimmu.2019.02756.
44. Kasahara, M., and Flajnik, M.F. (2019). Origin and evolution of the specialized forms of proteasomes involved in antigen presentation. Immunogenetics *71*, 251–261. https://doi.org/10.1007/s00251-019-01105-0.
45. Bergendahl, L.T., Gerasimavicius, L., Miles, J., Macdonald, L., Wells, J.N., Welburn, J.P.I., and Marsh, J.A. (2019). The role of protein complexes in human genetic disease. Protein Sci. *28*, 1400–1411. https://doi.org/10.1002/pro.3667.
46. Çetin, G., Studencka-Turski, M., Venz, S., Schormann, E., Junker, H., Hammer, E., Völker, U., Ebstein, F., and Krüger, E. (2022). Immunoproteasomes control activation of innate immune signaling and microglial function. Front. Immunol. *13*, 982786. https://doi.org/10.3389/fimmu.2022.982786.
47. Griffin, T.A., Nandi, D., Cruz, M., Fehling, H.J., Kaer, L.V., Monaco, J.J., and Colbert, R.A. (1998). Immunoproteasome assembly: cooperative incorporation of interferon gamma (IFN-gamma)-inducible subunits. J. Exp. Med. *187*, 97–104. https://doi.org/10.1084/jem.187.1.97.
48. De, M., Jayarapu, K., Elenich, L., Monaco, J.J., Colbert, R.A., and Griffin, T.A. (2003). Beta 2 subunit propeptides influence cooperative proteasome assembly. J. Biol. Chem. *278*, 6153–6159. https://doi.org/10.1074/jbc.M209292200.
49. de Freitas Chama, L.L., Ebstein, F., Wiesrecker, B., Wagh, P.R., Hammer, E., Weiss, F.U., Junker, H., Studencka-Turski, M., Lerch, M.M., Krüger, E., and Sendler, M. (2021). Immunoproteasome impairment via beta5i/LMP7-deletion leads to sustained pancreatic injury from experimental pancreatitis. J. Cell Mol. Med. *25*, 6786–6799. https://doi.org/10.1111/jcmm.16682.

50. Hewing, B., Ludwig, A., Dan, C., Pötzsch, M., Hannemann, C., Petry, A., Lauer, D., Görlach, A., Kaschina, E., Müller, D.N., et al. (2017). Immunoproteasome subunit ss5i/LMP7-deficiency in atherosclerosis. Sci. Rep. *7*, 13342. https://doi.org/10.1038/s41598-017-13592-w.
51. Brünger, T., Ivaniuk, A., Pérez-Palma, E., Montanucci, L., Cohen, S., Smith, L., Parthasarathy, S., Helbig, I., Nothnagel, M., May, P., and Lal, D. (2025). Conserved missense variant pathogenicity and correlated phenotypes across paralogous genes. Genome Biol. *26*, 197. https://doi.org/10.1186/s13059-025-03663-x.
52. Wiel, L., Baakman, C., Gilissen, D., Veltman, J.A., Vriend, G., and Gilissen, C. (2019). MetaDome: Pathogenicity analysis of genetic variants through aggregation of homologous human protein domains. Hum. Mutat. *40*, 1030–1038. https://doi.org/10.1002/humu.23798.
53. Kataoka, S., Kawashima, N., Okuno, Y., Muramatsu, H., Miwata, S., Narita, K., Hamada, M., Murakami, N., Taniguchi, R., Ichikawa, D., et al. (2021). Successful treatment of a novel type I interferonopathy due to a de novo PSMB9 gene mutation with a Janus kinase inhibitor. J. Allergy Clin. Immunol. *148*, 639–644. https://doi.org/10.1016/j.jaci.2021.03.010.
54. López-Cano, J., Monserrat-García, M.T., Goldbach-Mansky, R., Alehashemi, S., Rubio-Murillo, M., Montero-Valladares, C., Zulueta-Dorado, T., Boztug, K., Bernabeu-Wittel, J., and Neth, O. (2025). Neonatal-Onset CANDLE/PRAAS due to PSMB8 p.Gly209Arg: Clinical Course and Baricitinib Response. Int. J. Dermatol. https://doi.org/10.1111/ijd.70214.
55. Crow, Y.J., and Stetson, D.B. (2022). The type I interferonopathies: 10 years on. Nat. Rev. Immunol. *22*, 471–483. https://doi.org/10.1038/s41577-021-00633-9.
56. Nitta, T., Murata, S., Sasaki, K., Fujii, H., Ripen, A.M., Ishimaru, N., Koyasu, S., Tanaka, K., and Takahama, Y. (2010). Thymoproteasome shapes immunocompetent repertoire of CD8+ T cells. Immunity *32*, 29–40. https://doi.org/10.1016/j.immuni.2009.10.009.
57. Takahama, Y. (2023). The thymoproteasome in shaping the CD8(+) T-cell repertoire. Curr. Opin. Immunol. *83*, 102336. https://doi.org/10.1016/j.coi.2023.102336.
58. Meinhardt, A., Ramos, P.C., Dohmen, R.J., Lucas, N., Lee-Kirsch, M.A., Becker, B., de Laffolie, J., Cunha, T., Niehues, T., Salzer, U., et al. (2021). Curative Treatment of POMP-Related Autoinflammation and Immune Dysregulation (PRAID) by Hematopoietic Stem Cell Transplantation. J. Clin. Immunol. *41*, 1664–1667. https://doi.org/10.1007/s10875-021-01067-7.
59. Arima, K., Kinoshita, A., Mishima, H., Kanazawa, N., Kaneko, T., Mizushima, T., Ichinose, K., Nakamura, H., Tsujino, A., Kawakami, A., et al. (2011). Proteasome assembly defect due to a proteasome subunit beta type 8 (PSMB8) mutation causes the autoinflammatory disorder, Nakajo-Nishimura syndrome. Proc. Natl. Acad. Sci. USA *108*, 14914–14919. https://doi.org/10.1073/pnas.1106015108.
60. Liu, Y., Ramot, Y., Torrelo, A., Paller, A.S., Si, N., Babay, S., Kim, P.W., Sheikh, A., Lee, C.C.R., Chen, Y., et al. (2012). Mutations in proteasome subunit beta type 8 cause chronic atypical neutrophilic dermatosis with lipodystrophy and elevated temperature with evidence of genetic and phenotypic heterogeneity. Arthritis Rheum. *64*, 895–907. https://doi.org/10.1002/art.33368.
61. Patel, P.N., Hunt, R., Pettigrew, Z.J., Shirley, J.B., Vogel, T.P., and de Guzman, M.M. (2021). Successful treatment of chronic atypical neutrophilic dermatosis with lipodystrophy and elevated temperature (CANDLE) syndrome with tofacitinib. Pediatr. Dermatol. *38*, 528–529. https://doi.org/10.1111/pde.14517.
62. Verhoeven, D., Schonenberg-Meinema, D., Ebstein, F., Papendorf, J.J., Baars, P.A., van Leeuwen, E.M.M., Jansen, M.H., Lankester, A.C., van der Burg, M., Florquin, S., et al. (2022). Hematopoietic stem cell transplantation in a patient with proteasome-associated autoinflammatory syndrome (PRAAS). J. Allergy Clin. Immunol. *149*, 1120–1127.e8. https://doi.org/10.1016/j.jaci.2021.07.039.

The American Journal of Human Genetics, Volume *113*

Supplemental information

Monoallelic *PSMB8* variants cause PRAAS with immunodeficiency through impaired immunoproteasome assembly

Robin Wijngaard, Caspar I. van der Made, Sema Kalkan Uçar, Gayatri Ramakrishnan, Man Wang, Johannes Brand, Jill A. Rosenfeld, Tiphanie P. Vogel, Sarah K. Nicholas, Monika Weisz-Hubshman, Undiagnosed Diseases Network, Clara D.M. van Karnebeek, Eric J. Allenspach, Taylor E. Gardiner, Sumudu Perera Kimmantudawage, Zornitza Stark, Ruth K. Armstrong, Janine Campbell, Stefano Volpi, Enrico Drago, Marco Gattorno, Alice Grossi, Isabella Ceccherini, Alfredo Cabrera-Orefice, Bente Siebels, Thomas Mair, Hartmut Schlüter, Ruben L. Smeets, Ronald van Beek, Ingrid Goebel, Katrin Küchler, Søren W. Gersting, Alexander Hoischen, Lisenka E.L.M. Vissers, Ron A. Wevers, Catherine Meyer-Schwesinger, Saskia B. Wortmann, Machteld M. Oud, and Sergio Guerrero-Castillo

Supplemental Note: Case Reports

Supplemental Note: Additional complexome profiling findings

Supplemental Figures

Figure S1. Interferon signature score plots

Figure S2. Genetic evidence of identified variants

Figure S3. Variant positions and conservation along the linear protein sequence

Figure S4. Conformational changes due to p.Gly243Arg

Figure S5. Structural context of monoallelic variants identified in this study and their associated local frustration changes

Figure S6. Characterization of standard and immunoproteasome subunits in human osteosarcoma 143B cells and in macrophages derived from THP-1 cells

Figure S7. Enhanced expression of immunoproteasome-specific subunits in cytokine-stimulated human skin fibroblasts

Figure S8. Heatmaps and migration profiles of variant p.Ser90Phe.

Figure S9. Enhanced expression of immunoproteasome-specific subunits in fibroblasts from controls and variants p.S90F and p.SA235D after IFNg-stimulation.

Figure S10. Accumulation of 440-kDa intermediate in variant p.Ser90Phe.

Figure S11. Heatmaps of migration profiles of proteasome subunits and assembly factors in fibroblasts

Figure S12. PSMB8 peptide profiles

Figure S13. PSMB9 and PSMB10 peptide profiles

Figure S14. Gene ontology (GO) and Reactome enrichment analysis of differentially expressed proteins.

Figure S15. Clustal multiple sequence alignment of PSMB8, PSMB9, and PSMB10 protein sequences.

Figure S16. Comparison of structural and evolutionary features across paralogous positions in proteasome subunits for the same amino acid substitution.

Figure S17. Structural and evolutionary properties of monoallelic variants and at their paralogous positions.

Figure S18. Blue native electrophoresis of fibroblasts and molecular mass calibration

Supplemental Tables

Table S1. Variant curation for *PSMB8*, *PSMB9* and *PSMB10*

Table S2. Site-directed mutagenesis oligonucleotides

Table S3. Quantitative reverse transcription PCR primer sequences

Table S4. Clinical characteristics of included individuals

Table S5. Laboratory findings of included individuals

Table S6. Quantification of soluble serum factors in individual 5 and 7.

Table S7. Candidate gene variants and rare variants in proteasome subunits

Table S8. Frustration index and proteasomal contacts across wild-type and mutant variants

Table S9. List of significantly upregulated or downregulated proteins in the p.Ala235Asp cell line

Table S10. Enriched Gene Ontology (GO) terms and Reactome pathways in upregulated and downregulated protein sets

Table S11. Predicted structural and biophysical effects of paralogous variants in immunoproteasome β-subunits (*PSMB8*, *PSMB9* and *PSMB10*).

Table S12. Identified proteasome subunits and associated subcomplexes detected in control samples from THP1, 143B, and fibroblast cell lines

Supplemental Methods

Recruitment and ethics approvals

Cell culturing

Protein extraction and immunoblotting

In-gel proteasome proteolytic activity assay

Active proteasome subunit abundance assay

Complexome profiling

Genome sequencing and variant analysis

Cytokine measurements

Supplemental References

Members of the Undiagnosed Diseases Network (Version 3.31.25)

Supplemental Note: Case Reports

Family 1

The proband (individual 1) is the second child of non-consanguineous healthy Egyptian parents. He was born at 39 weeks' gestation following an uncomplicated pregnancy, and his neonatal course was unremarkable. His respiratory illnesses started at 3 months of age, requiring hospitalization. He subsequently had multiple episodes of pneumonia, at times at a monthly frequency, also requiring hospitalization. During early hospitalizations, there was concern for possible myopathy with hypotonia and elevated CK levels but normal EMG. Biopsy findings were suggestive of a muscular dystrophy (with focal loss and disarray of myofilaments and regenerating fibers with large/prominent nuclei). However, he does not currently have hypotonia, weakness, nor elevated CK levels. Growth/differentiation factor 15 (GDF15) was mildly elevated, which could be due to myopathy or chronic inflammation. Developmentally, there were early motor delays due to hypotonia, but he does not currently have developmental issues, only academic concerns likely related to multiple hospitalizations and ADHD. At 2-3 years of age, he presented with bloody diarrhea and was diagnosed with dysentery. Colonoscopy demonstrated ulcerative colitis. He has not required medications for symptom control since the age of 5 years. Poor growth remains a feature, requiring a G-tube for nighttime feeds. He was diagnosed with growth hormone deficiency at age 7 years. He has had intermittent elevations of AST/ALT/GGT. Given his multiple infections, he underwent immunology evaluations starting at age 5 years, which showed low IgG and poor vaccine responses, suggesting common variable immune deficiency (CVID). Further workup has shown a severe antibody defect with pan-hypogammaglobulinemia, low B cells with no switched memory cells, low NK cell numbers, and intermittent mild cytopenias. His NK cell subset distribution is roughly within the normal range, and he has normal NK cell cytotoxicity. He has normal T cells with normal function. A blood smear revealed vacuolated neutrophils. He does not have any history of malignancy, granulomatous-lymphocytic interstitial lung disease, or autoimmune disease. His cardiac evaluation has shown evidence of elevated right-sided pressures in the past, but these have normalized on repeat echocardiography assessments. By age 7 years, his lung damage was significant, with three lobes (right middle, right lower, and left lower) being nonfunctional. Administration of IVIG helped stabilize his lung disease. His airways were diffusely ectatic with mucosal irregularity. He underwent lung transplantation at age 10 years. Pathology evaluation of the explanted tissue revealed severe, variably necrotizing acute bronchitis and bronchiectasis of several medium and small airways, bronchiolectasis without inflammation, and very rare microscopic foci of small airway obstruction reminiscent of bronchiolitis obliterans, with no follicular bronchiolitis.

His history is also significant for bilateral conductive hearing loss and bilateral tympanic membrane perforations. Around 9 years of age he developed multiple warts on the face, neck, and torso. His lung transplant was complicated by respiratory failure requiring two days on extracorporeal membrane oxygenation (ECMO), with a possible stroke event identified on cerebral MRI. At age 11 years, 9 months, his height was 130.6cm (-2.39SD), weight was 29.4kg (-1.69SD); he is normocephalic. The interferon score was mildly elevated, although this measurement was performed during immunosuppressive treatment (Supplemental Methods, Figure S1A).

Family 2

Individual 2 is the first child of non-consanguineous parents of European descent in family 2. The father had no significant medical history. The mother of this family has a history of recurrent ear infections as a child and migraines but has otherwise been healthy with no invasive infection history, growth issues, or significant autoimmune manifestations. The female child (individual 2) was born at term gestation with an uncomplicated pregnancy and unremarkable neonatal course. At 7 months of age, she started to get recurrent ear infections and chronic diarrhea. At 11 months of age, she was admitted to the hospital for a seizure in the setting of viral URI symptoms and acute otitis media (AOM) following 3 days of Augmentin. She was afebrile with eye deviation to the left, clenched fists, and intermittent stiffening of the upper extremities. Infectious workup was unremarkable other than the AOM. Head CT demonstrated bilateral mastoid and middle ear effusions without bone destruction or soft tissue abscess. There was no evidence of intracranial infection, acute intracranial hemorrhage, transcortical infarction, or mass lesion. These findings were confirmed on cerebral MRI. EEG was consistent with focal seizures with central midline interictal discharges. Levitiracetam treatment was initiated, and she received ceftriaxone x2 days for the ear infection with clinical improvement. At 12 months of age, she underwent bilateral myringotomy with tube placement but continued to have persistent otorrhea with cultures including *Haemophilus influenza*. At the age of 16 months, she presented with dehydration, lethargy, tachycardia after one day of diarrhea, rash, fever to 102F and emesis. She rapidly decompensated with multiple seizures, depressed mental status, respiratory depression, and large variations in her heart rate and blood pressure. She required intubation, and despite active resuscitation, her neurological condition deteriorated, resulting in brain death. Life support was withdrawn with the provision of comfort care. At the time of death, respiratory virus PCR panel was positive for adenovirus and rhinovirus. Blood culture was positive *for Streptococcus pneumoniae*. Autopsy revealed acute neutrophilic meningitis suggestive of a bacterial infection. She had no evidence of pneumonia. She was noted to have bilateral basal ganglia calcifications.

Individual 3 is the third child of family 2. He was born at term gestation after an uncomplicated pregnancy. He started to get recurrent ear infections and frequent viral respiratory infections starting at 6 months of age and underwent bilateral myringotomy with ear tube placement at 9 months of age. However, he continued to have chronic otorrhea. Ear culture was positive for *Haemophilus haemolyticus* and treatment with amoxicillin resulted in resolution of the otorrhea. At 11 months of age, he presented with cough, rhinorrhea, fever, respiratory distress and an ear infection for which he had been taking amoxicillin. Respiratory viral PCR panel was positive for rhinovirus/enterovirus and adenovirus. Progressive increase in work of breathing and hypoxia prompted CXR which demonstrated vague opacities in the medial lung bases bilaterally. Emergency intubation occurred when he became unresponsive with agonal breathing following a seizure. He subsequently went into cardiac arrest and despite active resuscitation spontaneous circulation was unable to be achieved and he died. Postmortem bronchoalveolar lavage PCR was positive for adenovirus, rhinovirus, parainfluenza virus, *Streptococcus pneumoniae*, and *Haemophilus influenzae*. Autopsy revealed diffuse, bilateral, panlobar bronchopneumonia, likely bacterial with no evidence of infection elsewhere. Bilateral basal ganglia calcifications were also noted.

<u>Family 3</u>

The proband (individual 4) is the second child to non-consanguineous parents of European descent, delivered at 28+1 weeks' gestation by emergency caesarean section due to abnormal cardiotocography and oligohydramnios, birth weight 864g (9th percentile). The infant had hydrops fetalis and significant persistent pulmonary hypertension requiring advanced ventilation techniques in the early neonatal period. The neonatal period was further complicated by conjugated hyperbilirubinemia with evolving cholestasis and fluctuating transaminitis; hepatosplenomegaly with stable splenic infarct and ascites; biventricular cardiac hypertrophy; and strikingly, 'moth-eaten' long bone changes and metaphyseal splaying on X-ray imaging prompting an extensive (though unrevealing) search for congenital infections. The blood film showed infrequent leukocytes with coarse deep pink to purple staining cytoplasmic inclusions. Persistent cytopenias required near-daily platelet transfusions with more intermittent red cell transfusion. Immunological investigations revealed lymphopenia with a very low percentage of B-cells and low immunoglobulins. The infant was treated empirically with benzylpenicillin on radiographic grounds (though maternal syphilis serology was negative). Bone marrow aspirate demonstrated moderate dyserythropoiesis, near-absent megakaryocytes, and occasional leukocytes with coarse deep pink to purple cytoplasmic inclusions. He died having received the genetic diagnosis, aged 68 days (35+6 weeks' gestation) from multisystem organ failure, with acute cardiac decompensation, having received high dose steroid

therapy (modest improvement in thrombocytopenia) and shortly after starting immunomodulating agents in line with recommendations for treatment of interferonopathy.

Family 4

The proband (individual 5) is the only child of non-consanguineous Caucasian parents, the father has Multiple Sclerosis. The pregnancy, birth, anthropometric birth data, and postnatal adaptation were uneventful. No skin lesions were noted at birth. At the age of 3 months, following the live-attenuated rotavirus vaccination, she developed a gastroenteritis and was admitted to the pediatric intensive care unit with lactic acidosis, elevated transaminases, high triglycerides, increased ferritin, and pancytopenia as well as hypogammaglobulinemia. Hemophagocytic lymphohistiocytosis (HLH) was suspected and she was consequently started on steroid therapy with good response. Blood and urine amino acid analyses were additionally performed to investigate potential inherited metabolic diseases, showing mildly elevated lysine levels, and urinary organic acid analysis revealed dicarboxylic aciduria. Cranial MRI and MR spectroscopy showed no abnormalities. At 5 months of age, she was hospitalized with respiratory distress resulting from Respiratory Syncytial Virus (RSV) infection complicated by pneumonia. She additionally developed rhabdomyolysis and elevated liver transaminases without liver insufficiency. Due to persisting respiratory distress, a high-resolution chest CT and echocardiography were performed at the age of 6 months, detecting bronchiolitis obliterans and pulmonary arterial hypertension. She was started on monthly corticosteroids, intravenous immunoglobulin suppletion therapy (IVIG), and the PDE-5 inhibitor tildenafil. Moreover, ultrasonography and biopsy of the liver showed mild hepatomegaly with mild perisinusoidal and periductal fibrosis. At the age of 19 months she experienced a *Klebsiella pneumoniae* urinary tract infection, again complicated by rhabdomyolysis. Over the subsequent years, she experienced recurrent episodes of elevated transaminases, thrombocytopenia, and rhabdomyolysis, sometimes with diarrhea, during numerous milder episodes often triggered by respiratory tract infections. She had persistently low IgG levels requiring ongoing (IVIG) treatment. These episodes were often accompanied by skin lesions of the face, extremities or the whole body appearing as erythematous, edematous plaques with central blanching that resolved with brown discoloration and subsequent complete disappearance. Skin lesions resembling livedo reticularis were noted recurrently. Additionally, hypothyroidism was diagnosed at the age of 10 months and treated. Currently, at 4 years of age, she shows age adequate growth and development. She has received all vaccinations following the Turkish vaccination program and notably, has not suffered from invasive infections.

Immunological investigations starting from the first year of life showed neutropenia and mild lymphopenia with persistently low B cell and normal T and NK cell numbers. There was a reduction in

IgG, IgA and IgM production. No autoantibodies were detected. T cell populations were not notably different from controls and T cell proliferation in response to PHA stimulation was normal. A diagnostic interferon signature and soluble serum factors were additionally requested as a type I interferonopathy was suspected. Both showed evidence of an interferon type I immune response (Table S1).

Family 5

Family 5 includes two affected family members, mother (individual 6) and child (individual 7). During the first year of life the mother presented with recurrent pruritic polymorphous erythematous eruptions clinically described as erythema marginatum–like, with post-inflammatory hyperpigmentation. Hypogammaglobulinemia in the absence of recurrent infections was reported in the first year of life and diagnosed as transient hypogammaglobulinemia of infancy. In adolescence she developed progressive interstitial lung disease with basal bronchiectasis. Spirometry showed a very severe mixed ventilatory defect. Febrile, antibiotic-related episodes were associated with myalgia, tendinopathy, and marked creatine kinase (CK) elevations up to rhabdomyolysis. She exhibits adult short stature (<−2 SDS) and non-autoimmune primary hypothyroidism treated with levothyroxine.

The affected child was born late-preterm and small for gestational age (SGA) after intrauterine growth restriction (IUGR) and oligohydramnios. At 2 days of age, he developed a pruritic, serpiginous annular eruption consistent with erythema marginatum (Figure 1B), diarrhea and peripheral eosinophilia. Neuroimaging, chest imaging, and abdominal ultrasound were unremarkable.

During the first years, failure to thrive (FTT) persisted, affecting weight (≤−2 SDS) and height (≤-2 SDS), and a hypereosinophilic syndrome emerged with absolute eosinophil counts typically $1.5–4.0\times10^3/\mu L$ (peak $8.4\times10^3/\mu L$), involving the gastrointestinal tract, liver, and skin. Gastrointestinal disease manifested as chronic diarrhea, malabsorption, and iron-deficiency anemia, supported by endoscopic–histologic evidence of an eosinophil-predominant inflammatory colopathy (28 E/HPF). Hepatic involvement was characterized by eosinophilic hepatopathy with fluctuating transaminases. Liver biopsy demonstrated diffuse macrovesicular steatosis with portal eosinophils and reactive hepatocellular changes. Transient elastography showed no fibrosis, and flares were corticosteroid-responsive. Cutaneous disease comprised recurrent pruritic serpiginous annular erythema beginning in the neonatal period; dermatopathology reported spongiotic dermatitis with a dense eosinophilic infiltrate. In view of persistent eosinophilia and multiorgan involvement, mepolizumab (anti–IL-5) was started and associated with an early decline in eosinophils and aminotransferases, followed by later

transaminase rebounds without signs of liver failure. A 3-month course of oral steroid therapy only partially controlled liver enzymes.

At approximately 3½ years of age, after amoxicillin exposure, he developed a drug reaction compatible with eosinophilia and systemic symptoms (DRESS) but without eosinophilia (on mepolizumab treatment) complicated by acute myopericarditis with junctional rhythm, troponin elevation, and cardiac MRI consistent with myocardial–pericardial inflammation requiring admittance to our intensive care unit. He received intravenous immunoglobulin (IVIG) 2 g/kg, high-dose methylprednisolone, and milrinone with full clinical recovery and normalization of the electrocardiogram (EKG) and echocardiography. Across follow-up he also exhibited recurrent hyper–creatine kinase (CK) elevations, mostly during intercurrent infectious (of note these were not more severe or more frequent than in healthy peers of the same age) or inflammatory events.

The immunologic profile indicates a primary humoral immunodeficiency with persistent hypogammaglobulinemia, reduced IgG subclasses 1–3, and poor responses to T-dependent protein antigens; B-cell immunophenotyping shows marked memory reduction—low un-switched memory ($CD27^{+}IgD^{+}IgM^{+}$) and low switched memory ($CD27^{+}IgD^{-}IgM^{-}$)—with expansion of naïve cells and a low CD21low fraction for age (Table S2). Type I interferon signature was negative on several occasions.

Supplemental Note: Additional complexome profiling findings

Complexome profiling analysis on 143B and THP-1 cell lines

To determine whether IP 20S and 26S complexes were properly assembled in fibroblasts from individuals harboring a variant in *PSMB8*, complexome profiling was employed. This mass spectrometric technique separates protein complexes under native conditions to analyze their composition and abundance within a biological sample .

The ability of this technique to detect all proteasome subunits and to differentiate between the SP and the IP was first assessed using a non-immune cell line (143B) and an immune cell line (THP-1). The migration profiles in these cell lines showed two predominant peaks at ~750 kDa and ~2000 kDa, corresponding to the 20S and 26S complexes, respectively (Figure S6A). At the 20S peak, seven α and ten β-subunits were detected, including PSMB5-7 (SP-specific) and PSMB8-10 (IP-specific), indicating the presence of both proteasome types in immune and non-immune cell lines. The 26S complex additionally included ATPase and non-ATPase regulatory subunits from the 19S regulatory particle. A peak around ~1000 kDa, close to the 20S proteasome, was also detected, representing the 20S complex associated with the 11S or PA200 regulatory subunits. Additional peaks corresponding to proteasome subcomplexes were observed and are further detailed below ("Other proteasome assemblies detected in 143B, THP-1, and control fibroblast cell lines") and Table S11.

To differentiate the SP from the IP, we used abundances of PSMB5-7 and PSMB8-10 as SP-specific and IP-specific markers, respectively (Figure S6B). In the 143B cells, 95% of the proteasome corresponded to the SP, whereas in the THP-1 cells, the ratio was inverted, with 80% of the proteasome corresponding to the IP (Figure S6C). Based on these findings, although the apparent molecular masses of the 20S and 26S complexes of SP and IP were similar, we were able to distinguish between these two based on the quantification of the respective specific β-subunits.

Other proteasome assemblies detected in 143B, THP-1, and control fibroblast cell lines

To gain insight into the whole proteasome assembly process, additional patterns observed in the migration profiles and heatmaps were assessed.

In the complexome profiling data, we observed an intermediate of the 20S proteasome assembly in THP-1 and stimulated fibroblast cell lines at very low abundances. This structure contained a complete α-ring and included subunits PSMB2, PSMB3, PSMB9 and PSMB10. The presence of immunoproteasome (IP)-specific subunits PSMB9-10, and the absence of standard proteasome (SP)-specific subunits PSMB5–7, indicated that this intermediate is specific to the IP. The 20S assembly factors POMP and PSMG1–2 were also detected within this intermediate, consistent with their

known role early in proteasome biogenesis and their dissociation later in the assembly process. The abundance of this intermediate was substantially lower than that of the mature 20S and 26S complexes, and it was not detected in the 143B cell line, which is in line with the generally low expression of the IP in this non-immunological cell type.

Regarding the 19S regulatory particles, three distinct subassemblies were detected. Two of these corresponded to base subcomplexes: one composed of PSMC3, PSMC6, and PSMD9, and the other formed by PSMC1, PSMC2, PSMD2, and PSMD5. The third intermediate was part of the 19S lid and comprised subunits PSMD3, PSMD6, PSMD7, PSMD11, PSMD12, PSMD13 and PSMD14. Migration profiles of subunits PSMC1-6 and PSMD2 also showed another peak slightly smaller than the 26S, indicating that these subunits of the base were associated first to the 20S core particle. The complete 19S structure was only observed as part of the assembled 26S proteasome complex. This complex contained the PSMC1-6 subunits forming the ATPase module, as well as non-ATPase subunits previously identified in the subassemblies, along with PSMD1, PSMD4, and PSMD8. Notably, PSMD5, PSMD9 and PSMD10 were absent from the fully assembled 26S complex, consistent with their known roles as assembly chaperones rather than structural components. The only subunit known to be part of the human 26S complex (PDB: 6MSB) that was not identified by LC-MS/MS was SEM1.[1] This protein was not efficiently detected by mass spectrometry due to its small size and the lack of tryptic peptides of a detectable length.

Finally, we also detected subassembly structures of the 11S regulatory particles. At a similar molecular mass, we observed the presence of PSME1, PSME2, and PSME3. These signals corresponded to two distinct 11S complexes: the 11S αβ heteroheptamer formed by PSME1 and PSME2, and the 11S γ homoheptamer formed by PSME3. At the same molecular mass, we also detected the PA200 regulatory particle, formed by a single molecule of PSME4. Both the 11S αβ complex and PA200 were also observed in association with the 20S core particle, alone or as part of hybrid proteasomes incorporating the 20S core particle with the 19S and either 11S αβ or PA200. In contrast, the 11S γ complex was not detected in association with the 20S core particle.

All proteasome structures identified in this study through complexome profiling are presented in Figure S11B and summarized in Table S12.

Supplemental Figures

Figure S1. Interferon signature score plots

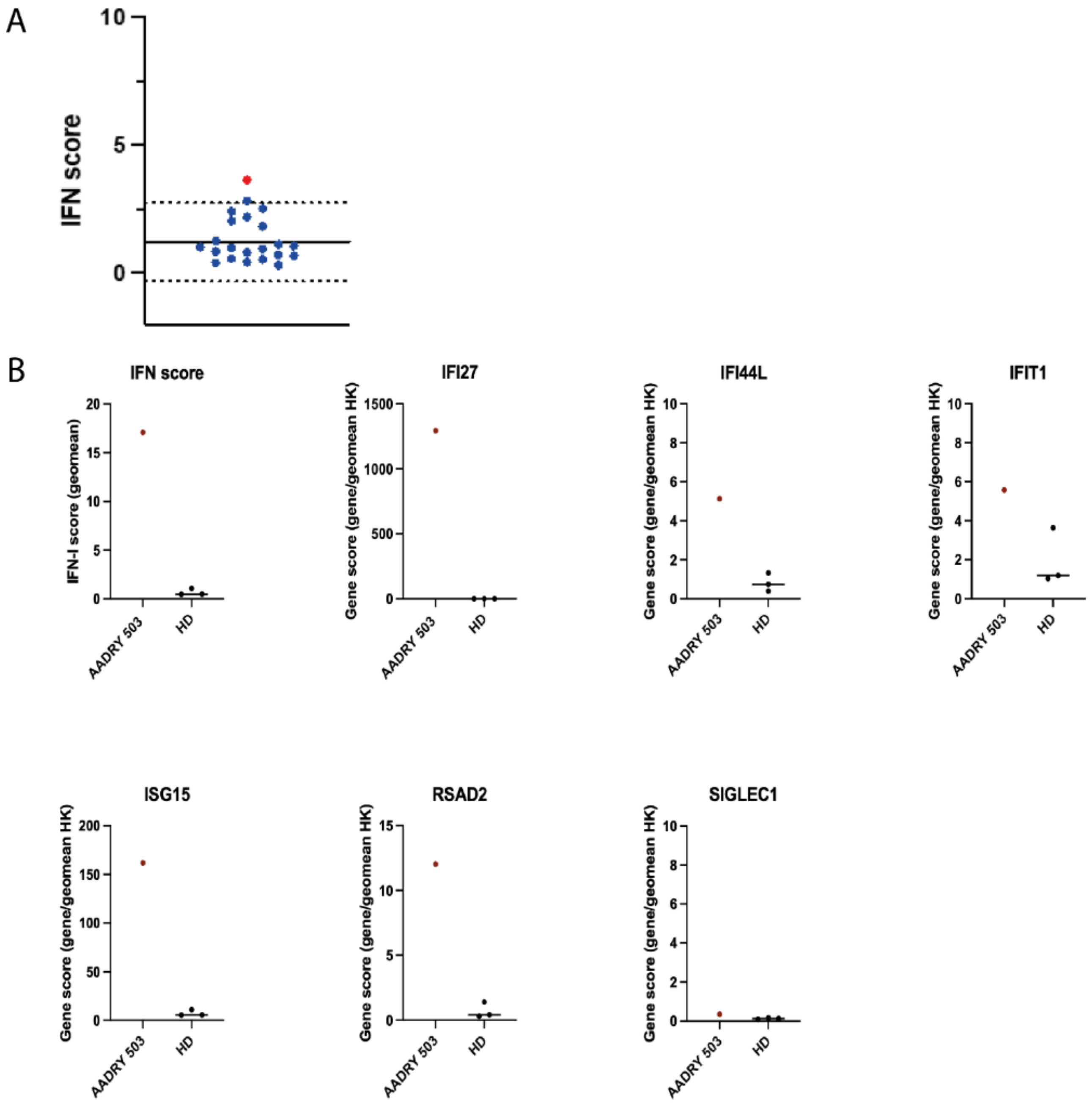

A: Blue are interferon (IFN) scores from healthy controls and red is the IFN score from individual 1, from a post-lung transplant specimen (pre-transplant samples were not available). Solid line represents the mean of healthy controls, dotted lines represent 2 standard deviations from the mean. **B**: IFN score and individual gene scores for individual 4.

Figure S2. Genetic evidence of identified variants

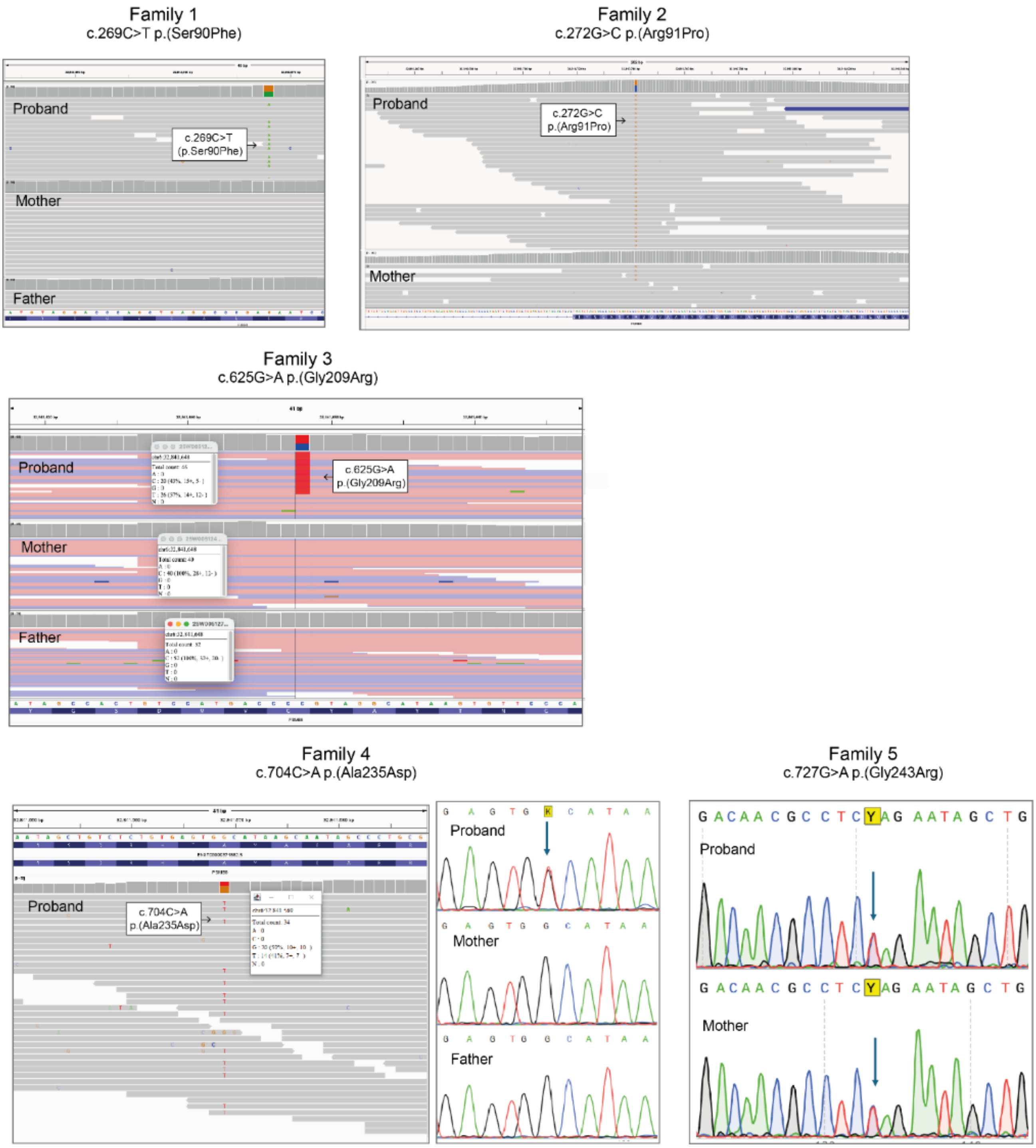

Visualization of the variant in genome sequencing data and/or Sanger sequencing traces in the proband and parents in the five included families.

Figure S3. Variant positions and conservation along the linear protein sequence

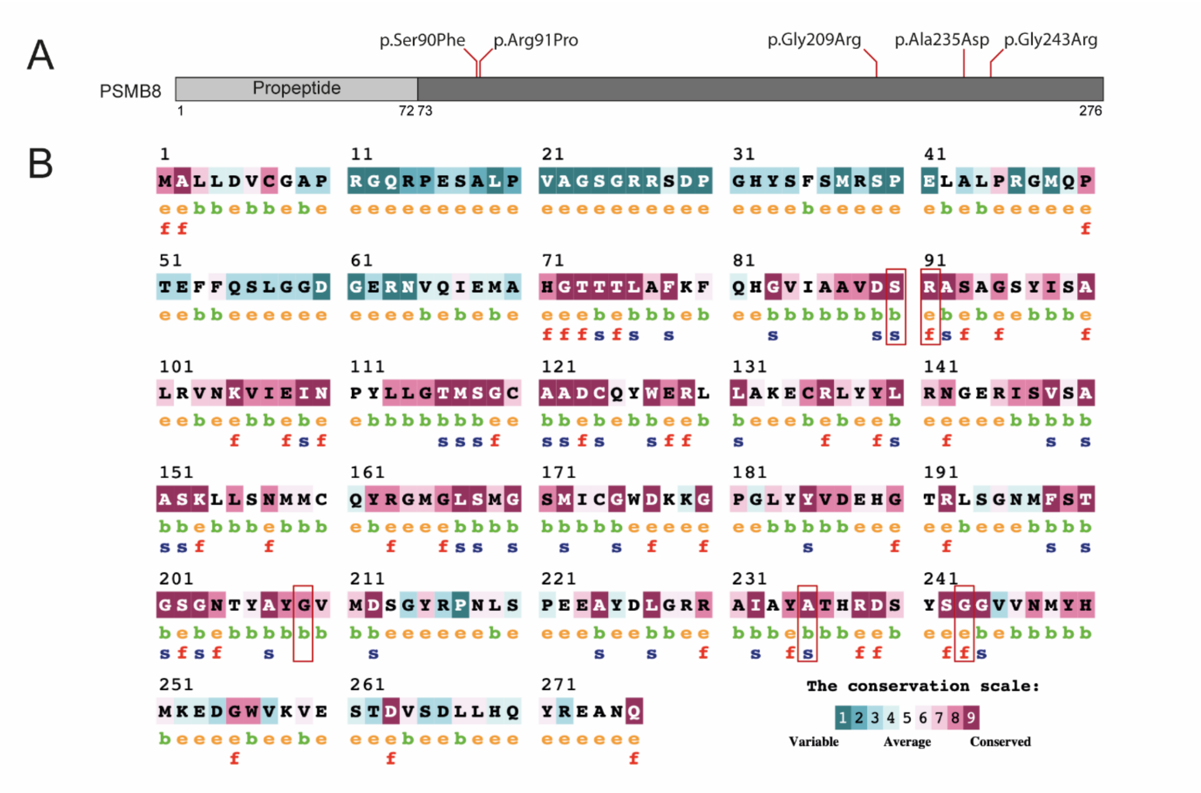

A: Linear protein structure with variant positions. **B**: ConSurf conservation scores with variants highlighted (red boxes). Letters below amino acids indicate: e = solvent-exposed; b = buried; f = functional residue (highly conserved and exposed); s = structural residue (highly conserved and buried).

Figure S4. Conformational changes due to p.Gly243Arg

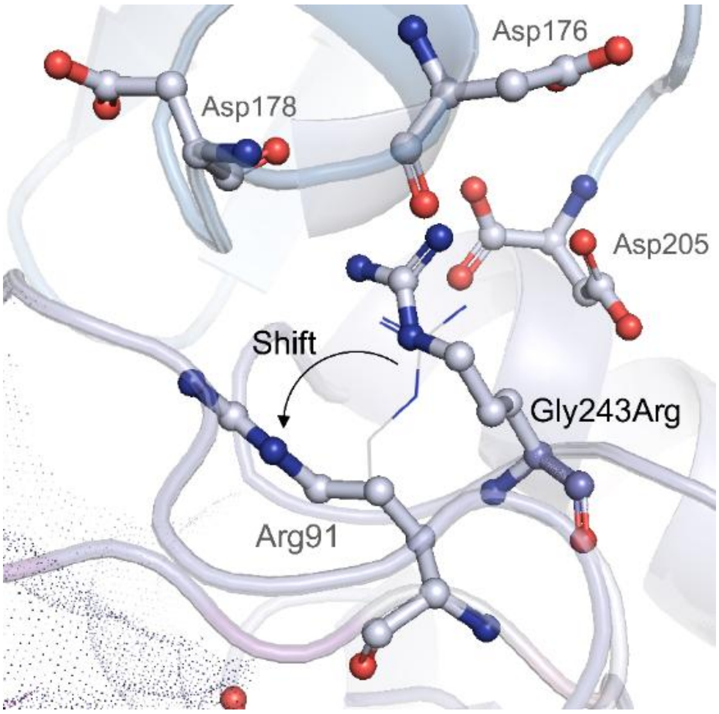

The p.Gly243Arg substitution causes substantial conformational changes in the surrounding residues, resulting in the loss of interactions between Arg91 and the PSMB3 subunit. In place of Arg91, the variant Arg243 establishes salt-bridges with aspartates in PSMB3, while repelling the positively charged sidechain of Arg91. This necessitates structural rewiring in the neighborhood which may be insufficient to fully accommodate the extra positive charge, resulting in significant protein instability.

Figure S5. Structural context of monoallelic variants identified in this study and their associated local frustration changes

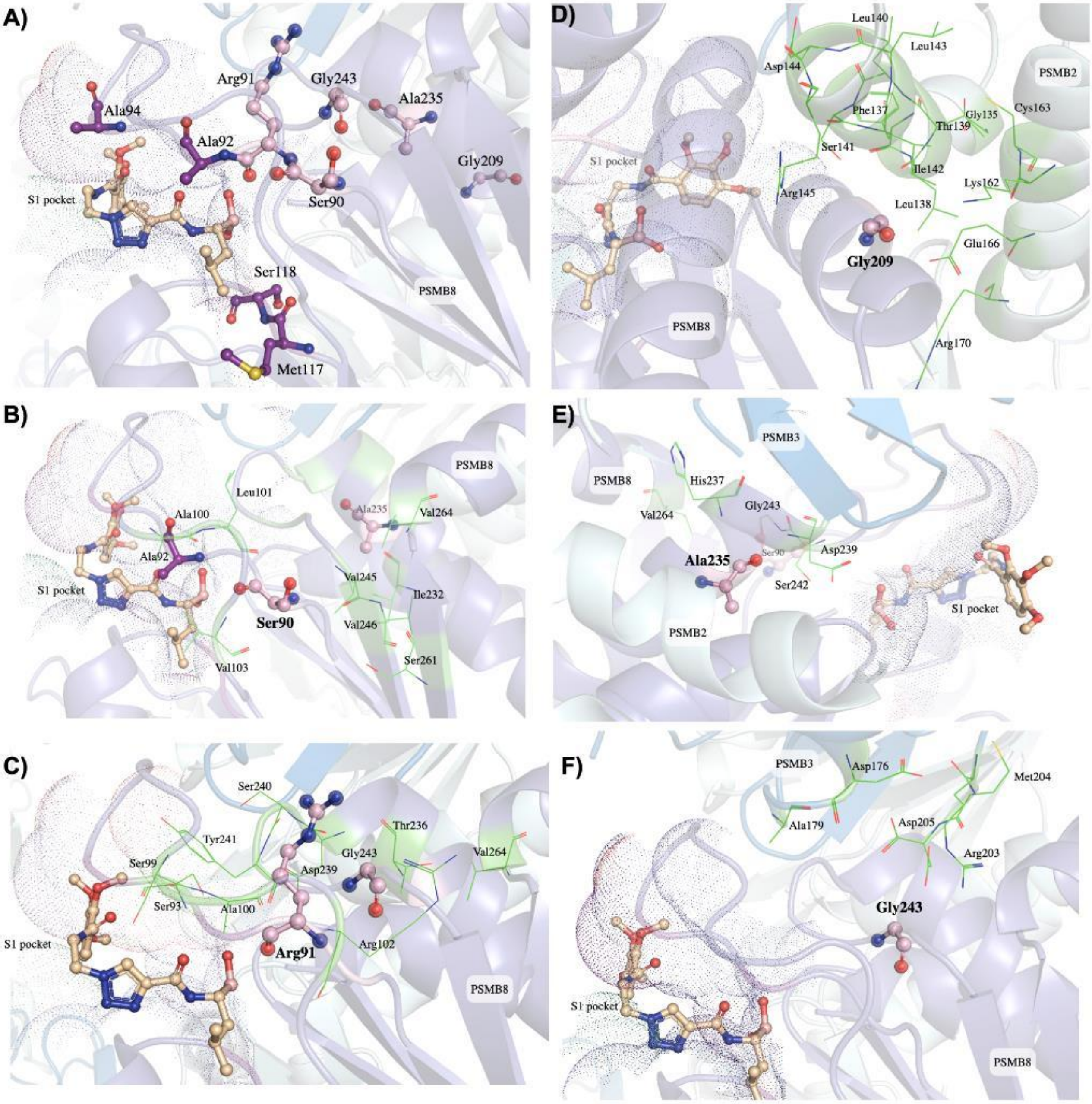

A: Variants assessed in this study are highlighted as ball and sticks. PRAAS-ID-associated variants are colored pink while classical PRAAS-associated variants are shown in violet. Immunoproteasome inhibitor (PDB ID: 6E5B) at S1 pocket is shown and retained in all panels as a reference location point.[2] **B**: Residues that undergo change in frustration energies in the variant p.(Ser90Phe) (neutral/high to minimally frustrated) located 5Å around Ser90 are shown in green. This set also includes the autosomal classical PRAAS-associated variant of interest Ala92 shown in purple. **C-F:** Similarly, residues in the neighborhood around p.(Arg91Pro), p.(Gly209Arg), p.(Ala235Asp), and p.(Gly243Arg) that exhibit changes in frustration energies are shown in green. Table S6 lists the residues in each of the variants' neighborhood.

Figure S6. Characterization of standard and immunoproteasome subunits in human osteosarcoma 143B cells and in macrophages derived from THP-1 cells

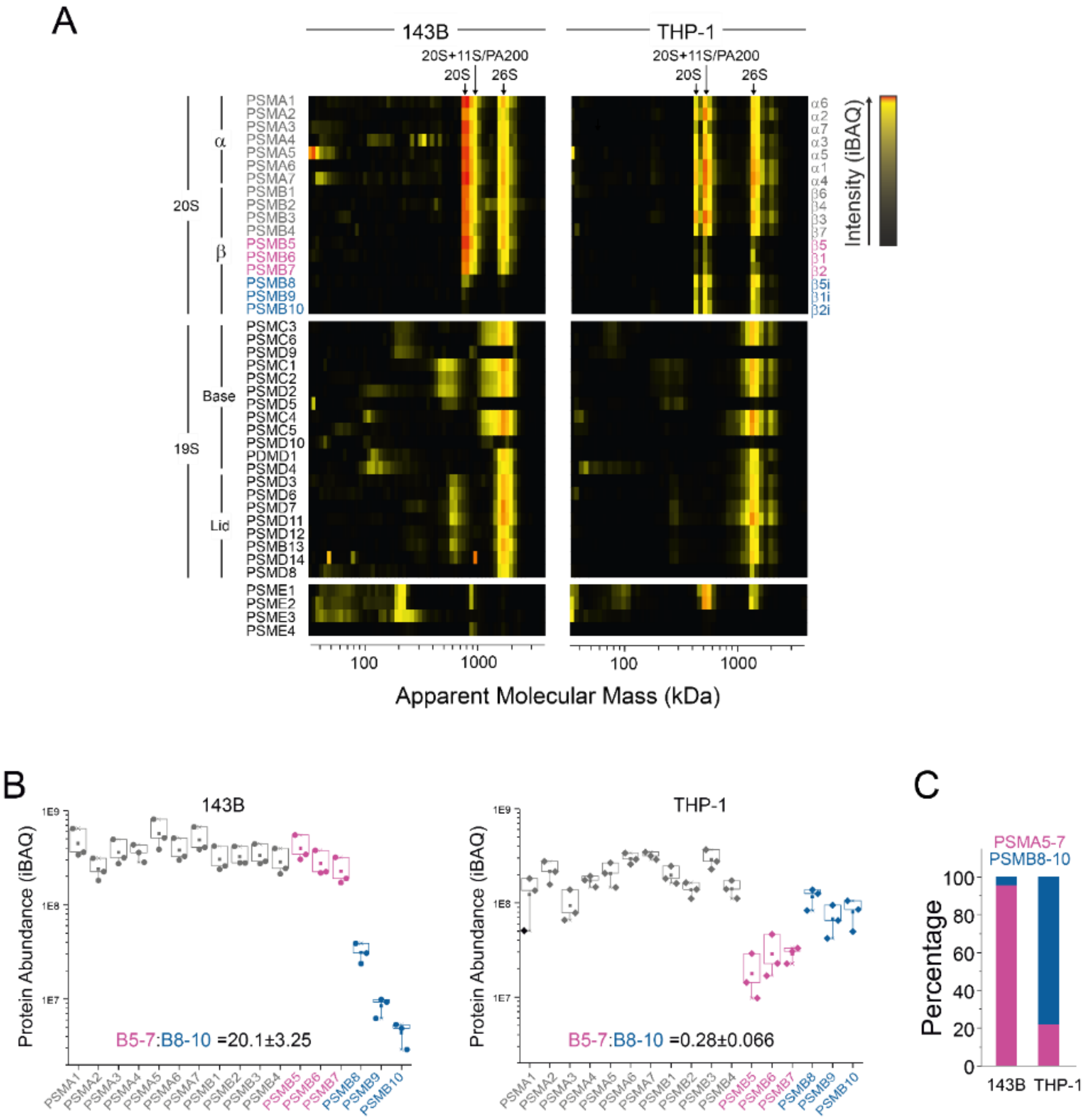

A: Heatmap representation of the migration profiles of 20S proteasome α- and β-subunits and 19S, 11S (PSME1-2) and PA200 (PSME4) proteasome regulatory proteins reflecting total abundances. Average of three independent experiments. **B:** Quantification of α- and β-subunits. Standard proteasomes predominated in 143B cells whereas in THP-1, immunoproteasomes were more prevalent. iBAQ, intensity-based abundance quantification. **C:** Quantification of the percentage of immunoproteasome content in both cell lines.

Figure S7. Enhanced expression of immunoproteasome-specific subunits in cytokine-stimulated human skin fibroblasts

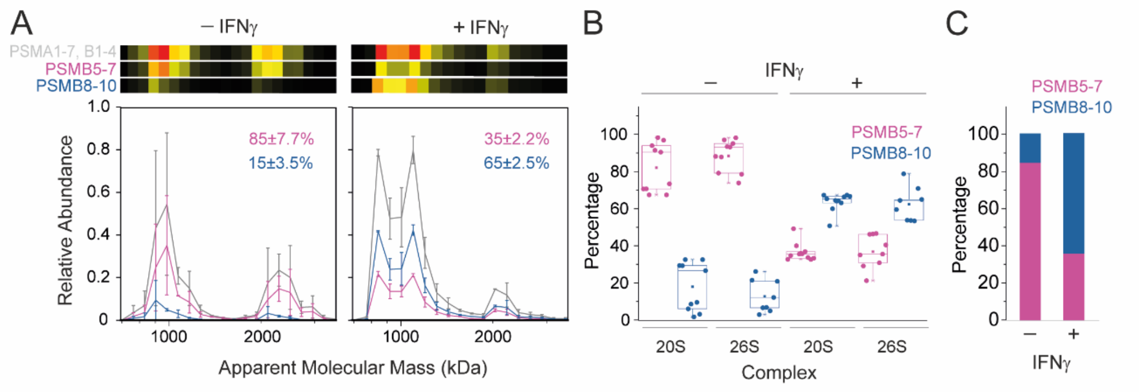

A: Heatmaps of the average migration profiles of shared (PSMA1-7, PSMB1-4), standard proteasome-specific (PSMB5-7) and immunoproteasome-specific (PSMB8-10) subunits in control fibroblasts without and after 48 h incubation with 250 U/mL human recombinant IFNγ prior to harvesting. Average of two independent experiments. **B:** Quantification of the immunoproteasome content in 20S and 26S inferred from SP-specific subunits:IP-specific subunits abundance ratios in gel fractions with the highest intensity values of 20S and 26S complexes. **C**: Quantification of the percentage of immunoproteasome content after IFNγ stimulation.

Figure S8. Heatmaps and migration profiles of variant p.Ser90Phe.

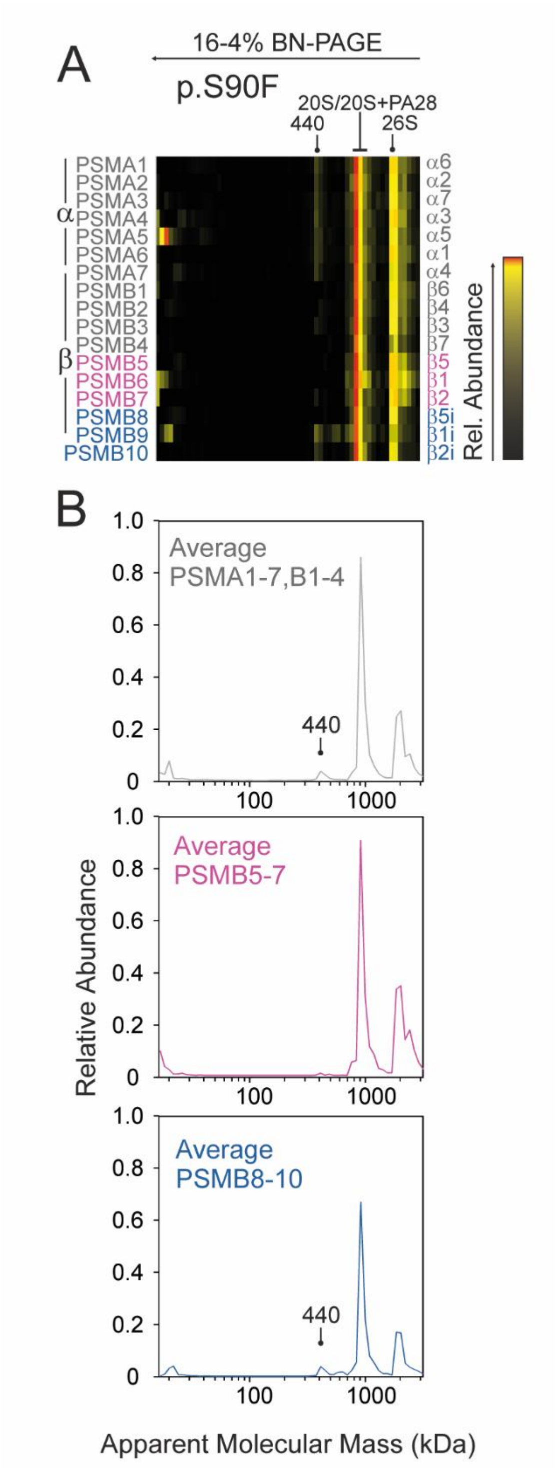

A: heatmap representation of migration profiles of α- and β-subunits showing signals for the 440-kDa intermediate, 20S+PA28 and 26S proteasome complexes in fibroblasts carrying *PSMB8* variant p.(S90F). **B**: Average migration profiles of shared subunits (gray), SP-specific β-subunits (pink), and IP-specific β-subunits (blue).

Figure S9. Enhanced expression of immunoproteasome-specific subunits in fibroblasts from controls and variants p.S90F and p.SA235D after IFNγ-stimulation.

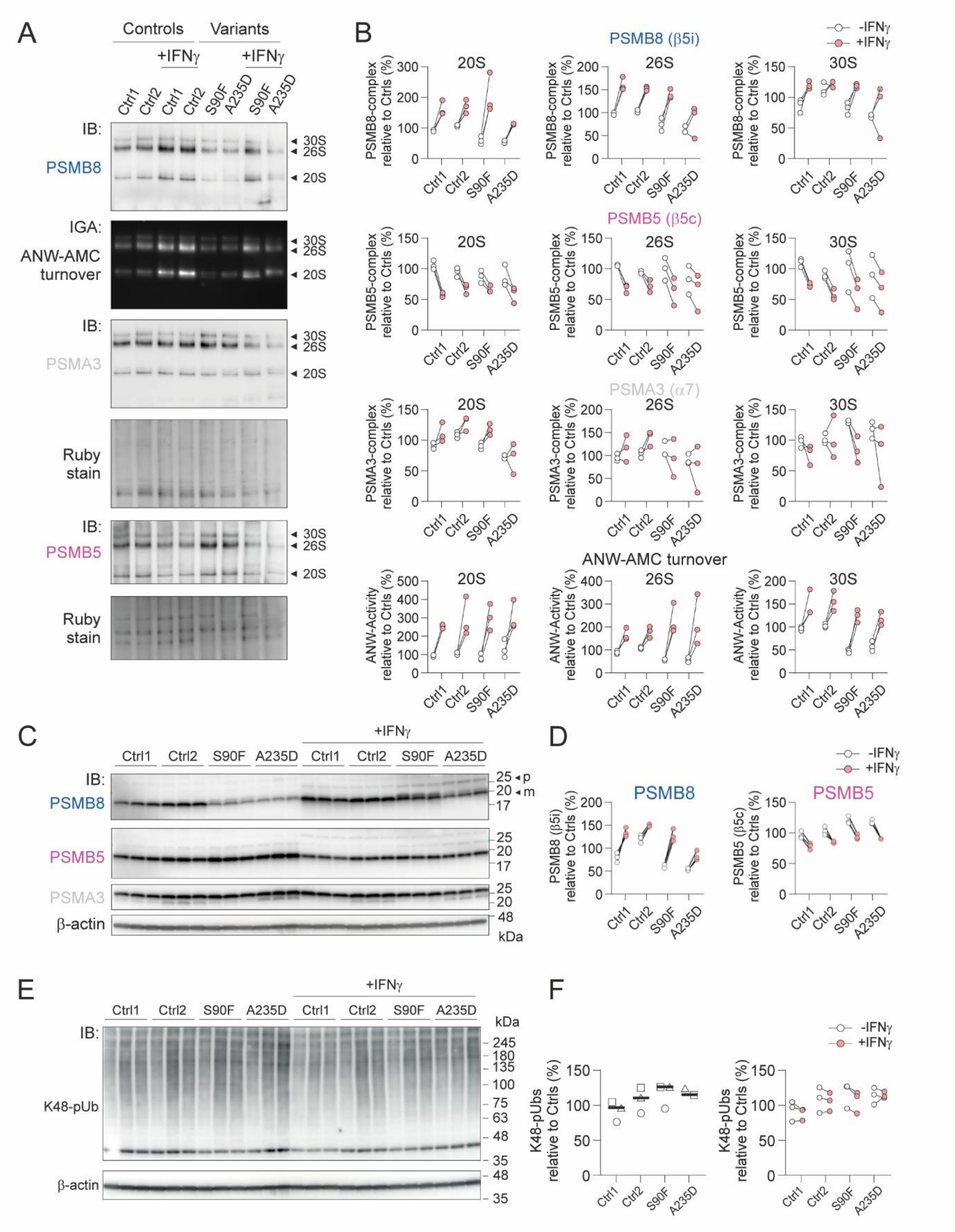

A: Immunoblots (IB) of PSMB8, PSMA3 and PSMB5 and in-gel activity (IGA) after native separation of non-induced and induced fibroblasts with IFNγ for 48 h. Representative blot (n=3). **B**: densitometric quantification of PSMB8 and PSMB5 signals at 20S and 26S complexes relative to non-induced

control fibroblasts. **C**: Immunoblots of PSMB8, PSMB5, PSMA3 and actin after denaturing separation of non-induced and induced fibroblasts with IFNγ for 48 h. Three independent cultures of each individual were induced or not with IFNγ. Precursor (p) and mature (m) PSMB8 forms are indicated. **D**: densitometric quantification of mature PSMB8 and PSMB5 after IFNγ induction for 48 h, relative to non-induced control fibroblasts. E: Protein polyubiquitination assessed by immunoblotting K48-polyubiquitin. **F:** Quantification of K48-poly-ubiquitin across variants and after IFNγ treatment.

Figure S10. Accumulation of 440-kDa intermediate in variant p.Ser90Phe.

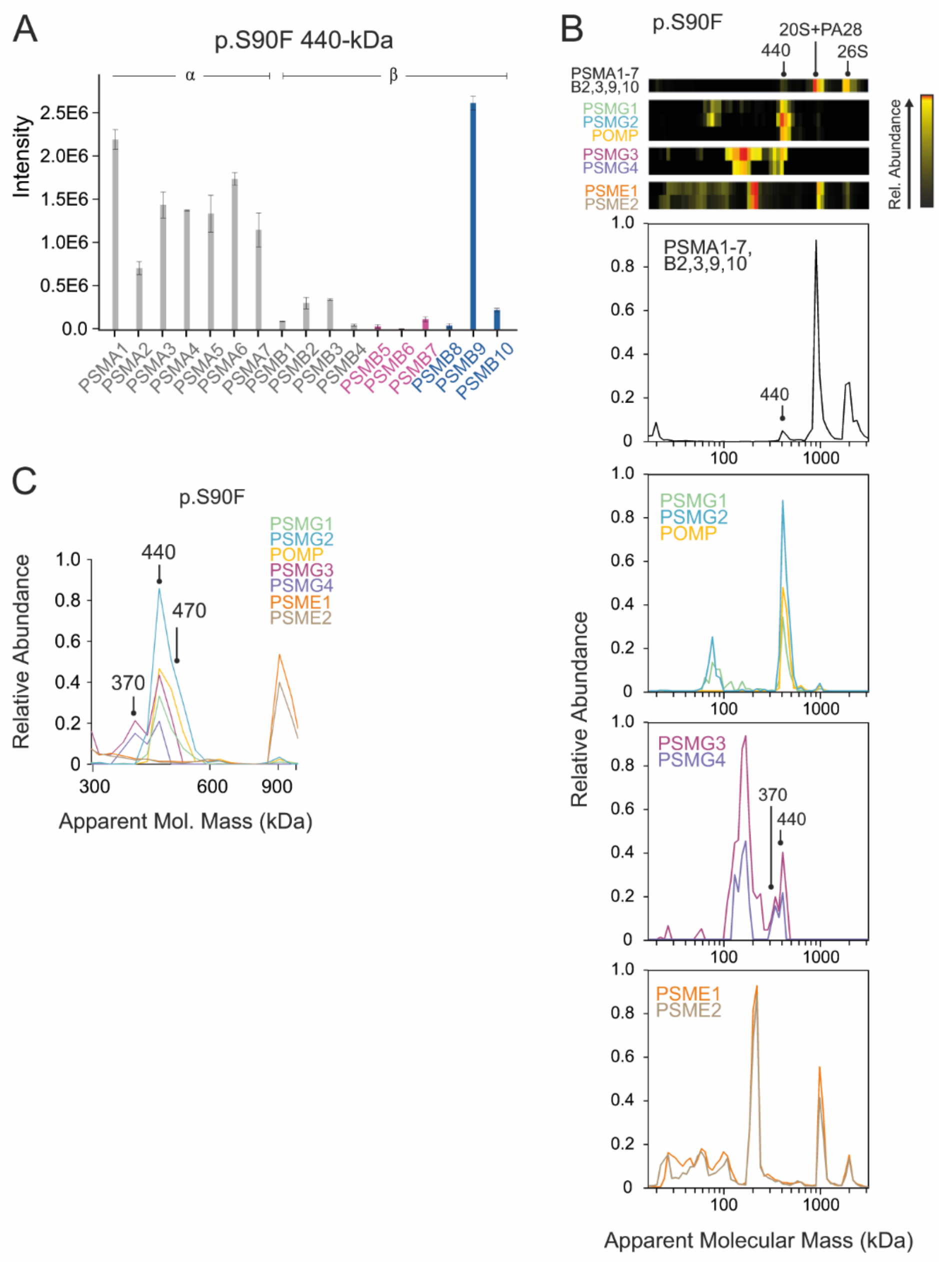

A: Quantification of α- and β-subunits at 440 kDa. **B**: Heatmap representation and migration plots of the average of α- and β-subunits integrating the ~440-kDa intermediate (PSMA1-7, PSMB2-3/9-10), proteasome assembly chaperones PSMG1-4 and POMP, and components of the 11S regulatory particle, PSME1 and PSME2. Average of two independent experiments. **C**: Zoom-in of the protein migration profiles around ~440 kDa showing the stepwise incorporation and release of assembly chaperones.

Figure S11. Heatmaps of migration profiles of proteasome subunits and assembly factors in fibroblasts

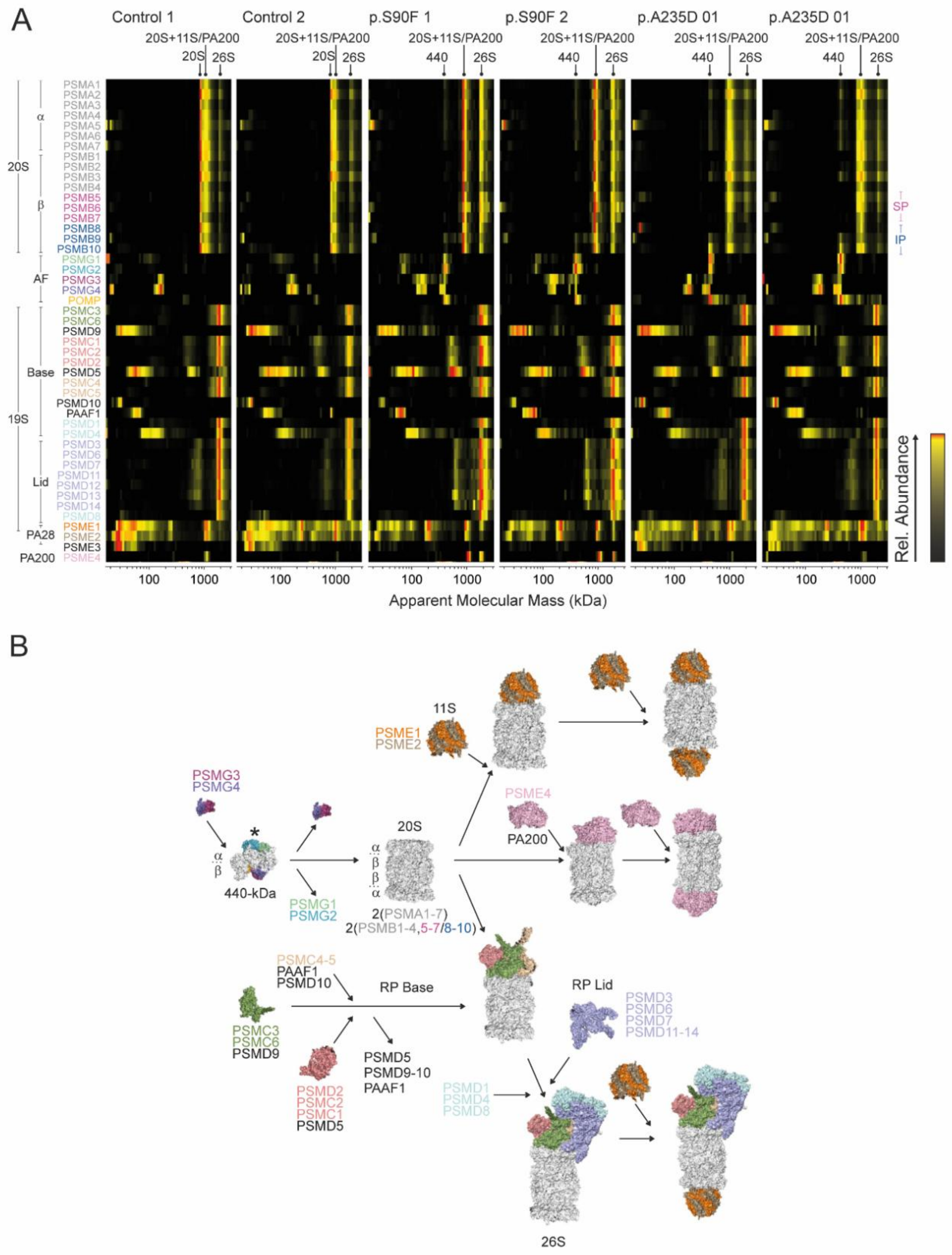

A: Heatmap representation of the migration profiles of proteasome subunits and assembly factors in control fibroblast and from individuals carrying variants p.S90F and p.A235D. Each protein's values were normalized independently, with 0 representing the lowest and 1 the highest value observed for that protein. **B**: Model of proteasome assembly based on intermediates and fully assembled proteasome complexes evidenced by complexome profiling. Subunits forming assembly intermediates are represented as cartoon illustrations, based on cryo-EM structures (PDB: 5GJR, 6E5B and 8QYJ).[1-3] AF: assembly factors.

Figure S12. PSMB8 peptide profiles

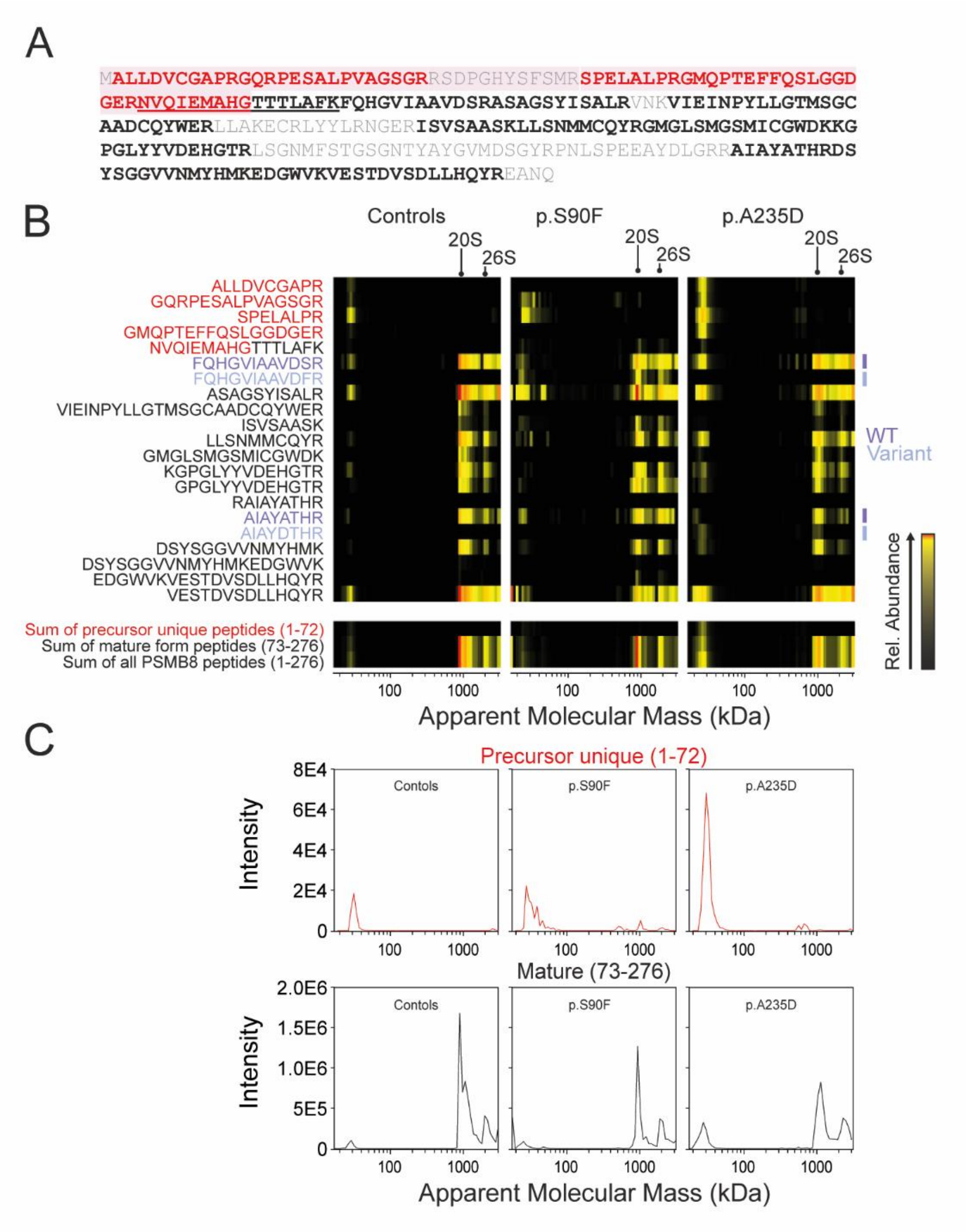

A: PSMB8 protein sequence with the propeptide sequence highlighted in light pink. Detected peptides corresponding to the propeptide are shown in red, while those from the mature form are shown in black. Identified peptide containing the cleavage site is underscored. **B**: Heatmap representation of the peptides identified in the controls and in variants p.Ser90Phe and p.Ala235Asp. **C:** Average migration profiles of precursor-unique peptides and of peptides covering the mature form of PSMB8. Average of two independent experiments.

Figure S13. PSMB9 and PSMB10 peptide profiles

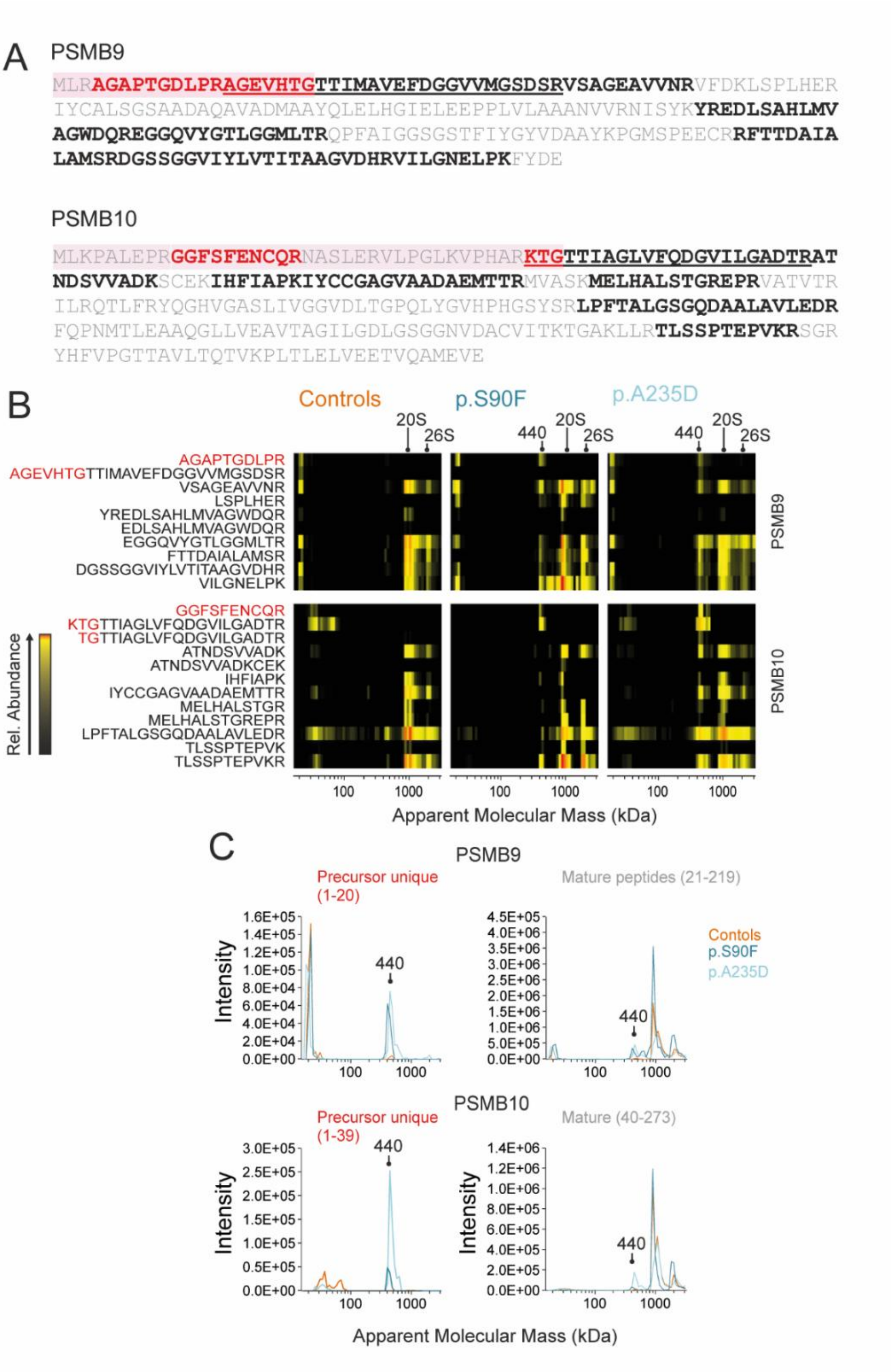

A: PSMB9 and PSMB10 protein sequences with the propeptide sequences highlighted in light pink. Detected premature-unique peptides are shown in red, while those from the mature proteins are shown in black. **B**: Heatmap representation of the PSMB9 and PSMB10 peptides present in the control and in variants p.Ser90Phe and p.Ala235Asp. Average of two independent experiments. **C**: Average migration profiles from precursor-unique peptides and mature PSMB9 and PSMB10 peptides.

Figure S14. Gene ontology (GO) and Reactome enrichment analysis of differentially expressed proteins.

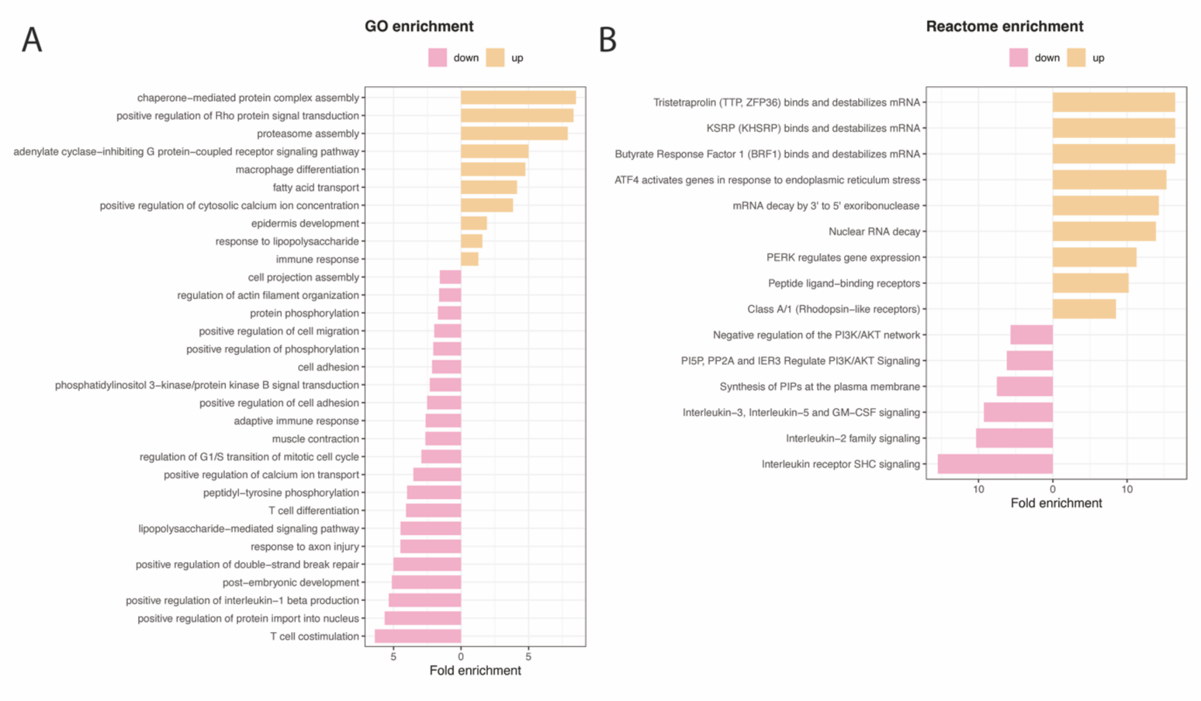

A: GO terms and **B:** Reactome pathways enriched among upregulated and downregulated proteins. Bar length represents fold enrichment (observed/expected).

Figure S15. Clustal multiple sequence alignment of PSMB8, PSMB9, and PSMB10 protein sequences.

```
PSMB10      ------------------------------------MLKPALEPRGGFSFENCQRNASLE      24
PSMB8       MALLDVCGAPRGQRPESALPVAGSGRRSDPGHYSFSMRSPELALPRGMQPTEFFQSLG--      58
PSMB9       ----------------------------------------------------MLRAGA--      6
                                                                  :  .

PSMB10      RVLPGLKVPHARKTGTTIAGLVFQDGVILGADTRATNDSVVADKSCEKIHFIAPKIYCCG      84
PSMB8       -GDGERNVQIEMAHGTTTLAFKFQHGVIAAVDSRASAGSYISALRVNKVIEINPYLLGTM      117
PSMB9       -PTGDLPRAGEVHTGTTIMAVEFDGGVVMGSDSRVSAGEAVVNRVFDKLSPLHERIYCAL      65
                          ***  .. *: **: . *:*.: .. :     :*:  :   :

PSMB10      AGVAADAEMTTRMVASKMELHALSTGREPRVATVTRILRQTLFRYQG-HVGASLIVGGVD      143
PSMB8       SGCAADCQYWERLLAKECRLYYLRNGERISVSAASKLLSNMMCQYRGMGLSMGSMICGWD      177
PSMB9       SGSAADAQAVADMAAYQLELHGIELEEPPLVLAAANVVRNISYKYR-EDLSAHLMVAGWD      124
            :* ***.:    : * : .*: :   .   * :.:.:: :   :*:   :.   :: * *

PSMB10      LTGPQLYGVHPHGSYSRLPFTALGSGQDAALAVLEDRFQPNMTLEAAQGLLVEAVTAGIL      203
PSMB8       KKGPGLYYVDEHGTRLSGNMFSTGSGNTYAYGVMDSGYRPNLSPEEAYDLGRRAIAYATH      237
PSMB9       QREGGQVYGTLGGMLTRQPFAIGGSGSTFIYGYVDAAYKPGMSPEECRRFTTDAIALAMS      184
                        *      :   ***.    . ::  ::*.:: * .  :   *:: .

PSMB10      GDLGSGGNVDACVITKTGAKLLRTLSSPTEPVKRSGRYHFVPGTTAVLTQTVKPLTLELV      263
PSMB8       RDSYSGGVVNMYHMKEDGWVKVESTDVSDL-------LHQYREANQ--------------      276
PSMB9       RDGSSGGVIYLVTITAAGVDHRVI-LGNEL-------PKFYDE-----------------      219
             *  *** :    :.  *                    :

PSMB10      EETVQAMEVE      273
PSMB8       ----------      276
PSMB9       ----------      219
```

Residues reported to harbor PRAAS-ID-associated variants are highlighted in blue. Highlighted positions correspond to Ser90, Arg91, Gly209, Ala235, and Gly243 in PSMB8, Gly156 in PSMB9, and Asp56 and Gly201 in PSMB10.

Figure S16. Comparison of structural and evolutionary features across paralogous positions in proteasome subunits for the same amino acid substitution.

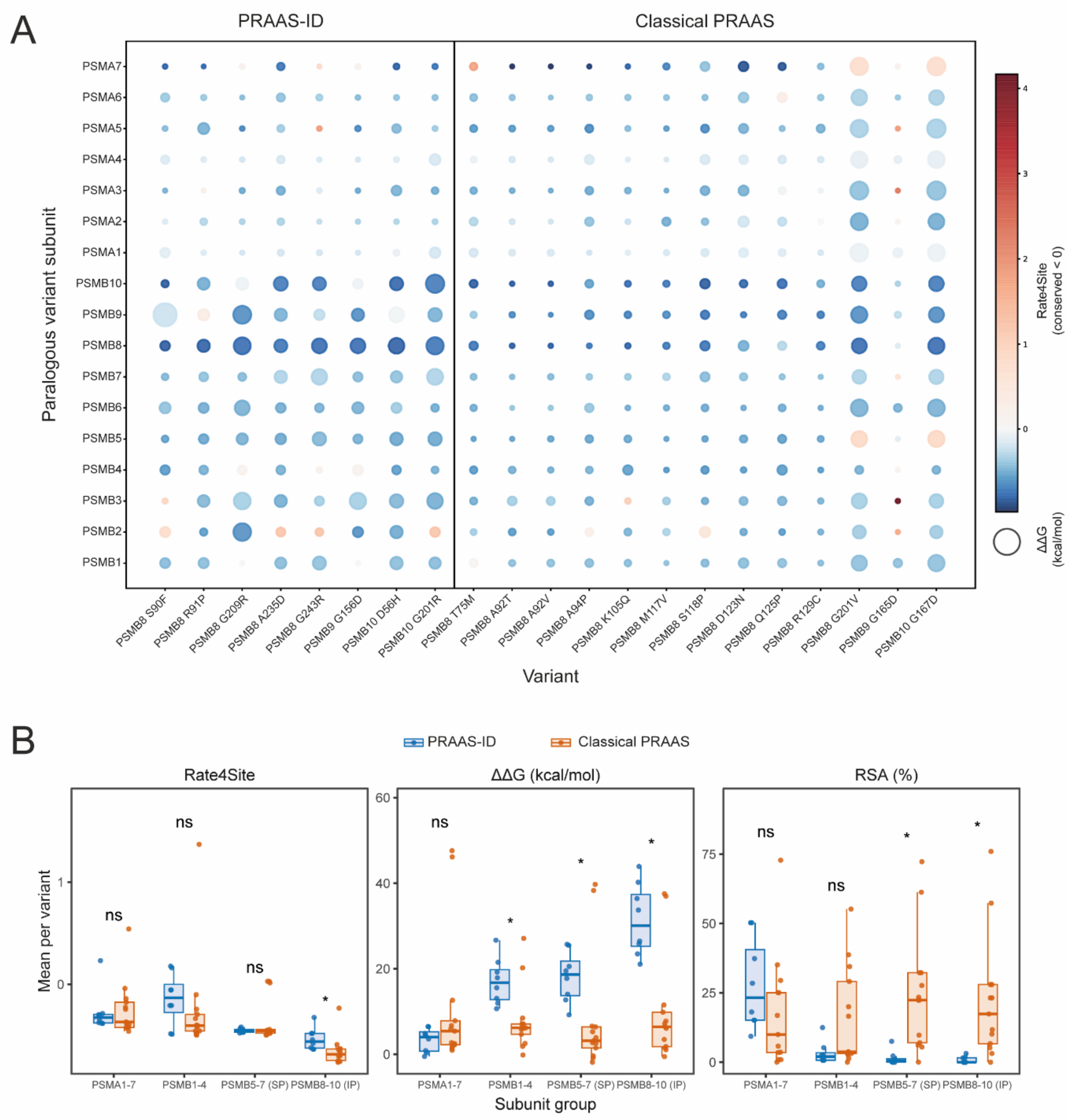

A: Bubble plot of variants, classified as PRAAS-ID or classical PRAAS, showing evolutionary conservation and predicted structural impact of the same amino acid substitution at paralogous positions across proteasome subunits. Bubble color indicates the Rate4Site score (lower values reflect greater conservation), and bubble size indicates the predicted FoldX ΔΔG (kcal/mol). **B:** Distribution of Rate4Site, FoldX ΔΔG (kcal/mol), and RSA (%) values per variant, averaged across subunits within each subunit group.

Significance levels are indicated as ns = non-significant, * = p ≤ 0.05, ** = p ≤ 0.01, and *** = p ≤ 0.001

Figure S17. Structural and evolutionary properties of monoallelic variants and at their paralogous positions.

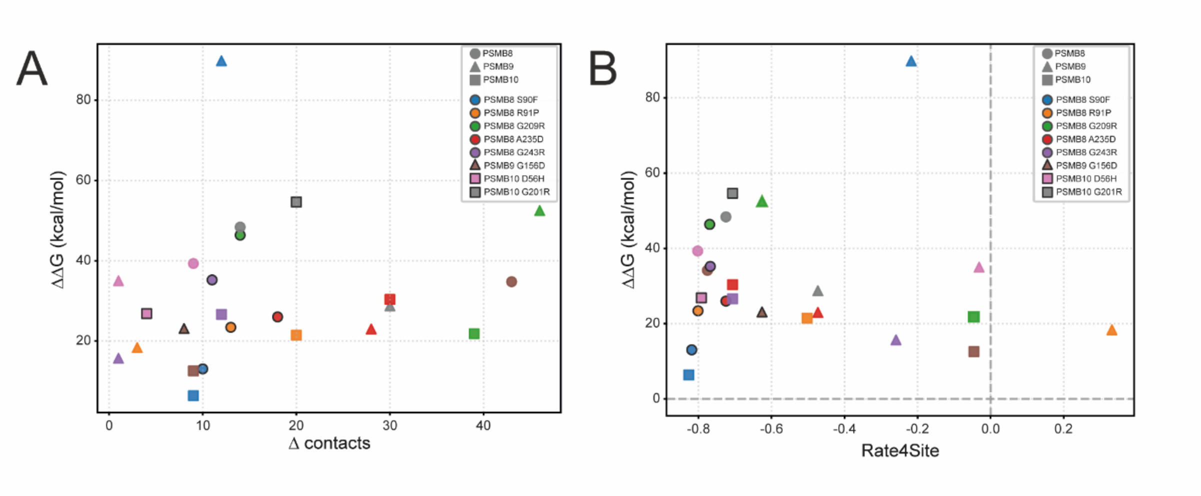

Scatter plots showing predicted structural effects of PRAAS-ID variants and paralogous immunoproteasome β-subunits (*PSMB8*, *PSMB9*, *PSMB10*). Points are colored by variant position. Variants reported in patients are highlighted with a black outline, whereas paralogous variants carrying the same amino acid substitution at equivalent positions lack the outline. **A:** Relationship between predicted destabilization (FoldX ΔΔG) and intermolecular contact perturbation (Δ contacts). **B:** Relationship between predicted destabilization (FoldX ΔΔG) and evolutionary conservation (Rate4Site).

Figure S18. Blue native electrophoresis of fibroblasts and molecular mass calibration

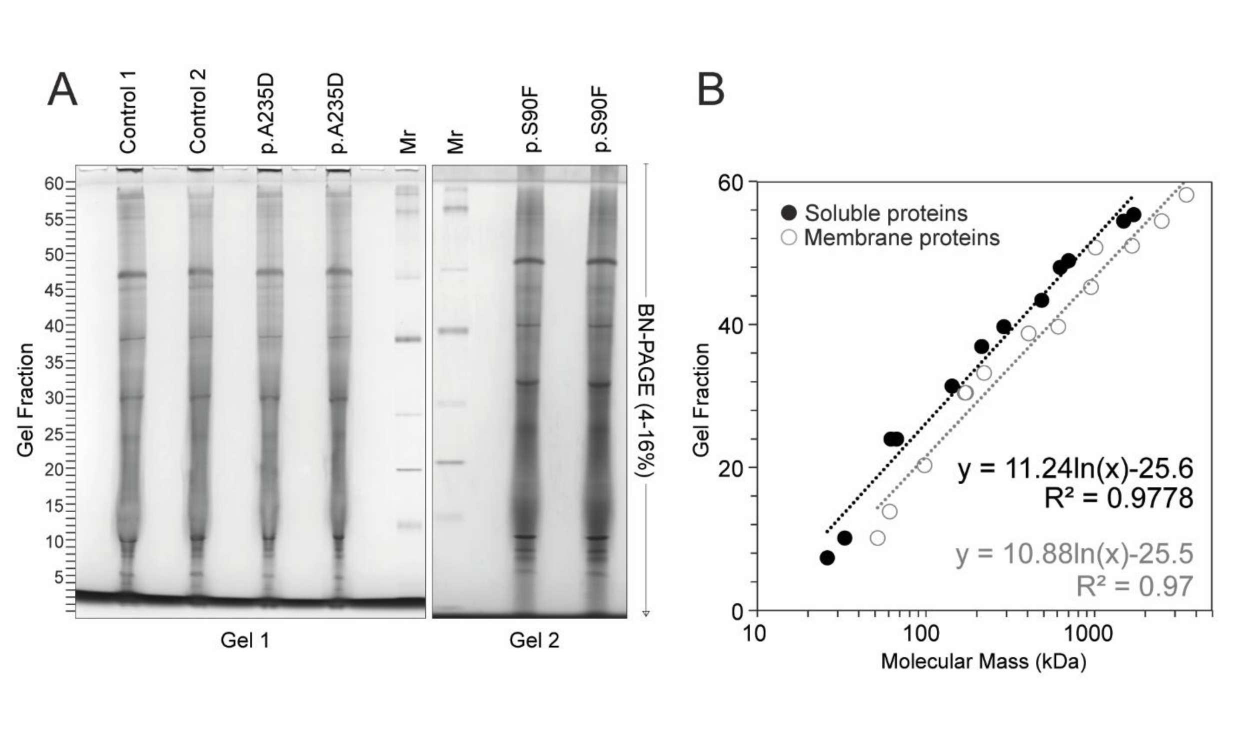

A: Gel images showing protein separation by blue native electrophoresis and Coomassie staining. **B:** Molecular mass calibration using a reference set of soluble and membrane protein complexes with known molecular masses and stoichiometries. The following proteins and complexes were used to calculate the apparent molecular masses of globular (hydrophilic) protein complexes in each gel fraction: monomeric ATP synthase subunit beta (ATP5F1B, 51.7 kDa), tetrameric single-stranded DNA-binding protein (SSBP1, 15.2×4=61 kDa), dimeric citrate synthase (CS, 49×2=98 kDa), complex I (CI) Q-module (NDUFS2, NDUFS3, NDUFS7, NDUFS8, NDUFA5, NDUFAF3, NDUFAF4, 170 kDa), UDP-glucose:glycoprotein glucosyltransferase 1 (UGGT1, 173 kDa), aldehyde dehydrogenase X tetramer (ALDH1B1, 55.3×4=221 kDa), 60 kDa heat shock protein heptamer (HSPD1, 58×7=406 kDa), isocitrate dehydrogenase [NAD] (IDH3A, IDH3B, IDH3G heterooctamer 300 kDa), propionyl-CoA carboxylase (PCCA, PCCB heterododecamer, 832 kDa), TRiC chaperonin (TCP1, CCT2-5, CCT6A/B, CCT7-8 heterohexadecamer, 944 kDa), alpha ketoglutarate dehydrogenase complex (OGDH, DLST, DLD, MRPS36, ca. 3500 kDa). The following transmembrane protein complexes were used as standards for hydrophobic protein complexes: MIC27 monomer (26 kDa), SLC25A5 (33 kDa), VDAC1-2 dimer (62 kDa), TOMM70 (67 kDa), succinate dehydrogense complex (143 kDa), cytochrome *c* oxidase (CIV, 214 kDa), VDAC1-3 nonamers (290 kDa), cytochrome bc_1 complex dimer ($CIII_2$, 485 kDa), ATP synthase (626 kDa), supercomplex $CIII_2$-CIV (700 kDa), supercomplex CI-$CIII_2$ (1485 kDa), respirasome CI-$CIII_2$-CIV (1708 kDa).

Supplemental Tables

Table S1. Variant curation for *PSMB8*, *PSMB9* and *PSMB10*

Gene	gDNA	cDNA	Protein	ClinVar[a]	PMID	Phenotype
PSMB8	chr6:g.32843013G>A	NM_148919.4:c.224C>T	p.(Thr75Met)	Pathogenic 2* (Variation ID: 659832)	21129723; 21953331; 26524591; 28895430; 29115062; 31874111; 37600812	Classical PRAAS
PSMB8	chr6:g.32842968G>A	NM_148919.4:c.269C>T	p.(Ser90Phe)	VUS 2* (Variation ID: 1308452)	This study	PRAAS-ID
PSMB8	chr6:g.32842965C>G	NM_148919.4:c.272G>C	p.(Arg91Pro)	VUS 1* (Variation ID: 2581896)	This study	PRAAS-ID
PSMB8	chr6:g.32842963C>T	NM_148919.4:c.274G>A	p.(Ala92Thr)	-	26524591; 28895430	Classical PRAAS
PSMB8	chr6:g.32842962G>A	NM_148919.4:c.275C>T	p.(Ala92Val)	-	31046790	Classical PRAAS
PSMB8	chr6:g.32842957C>G	NM_148919.4:c.280G>C	p.(Ala94Pro)	-	26567544	Classical PRAAS
PSMB8	chr6:g.32842766T>G	NM_148919.4:c.313A>C	p.(Lys105Gln)	Pathogenic 0* (Variation ID: 548954)	26524591; 31046790	Classical PRAAS
PSMB8	chr6:g.32842730T>C	NM_148919.4:c.349A>G	p.(Met117Val)	-	24001180; 26524591	Classical PRAAS
PSMB8	chr6:g.32842727A>G	NM_148919.4:c.352T>C	p.(Ser118Pro)	Pathogenic 0* (Variation ID: 3220941)	31874111; 33512037; 37600812	Classical PRAAS
PSMB8	chr6:g.32842712C>T	NM_148919.4:c.367G>A	p.(Asp123Asn)	VUS 1* (Variation ID: 870500)	32513120	Classical PRAAS
PSMB8	chr6:g.32842705T>G	NM_148919.4:c.374A>C	p.(Gln125Pro)	-	30387862	Classical PRAAS
PSMB8	chr6:g.32842706G>A	NM_148919.4:c.385C>T	p.(Arg129Cys)	VUS 2* (Variation ID: 870499)	32513120	Classical PRAAS
PSMB8	chr6:g.32841671C>A	NM_148919.4:c.602G>T	p.(Gly201Val)	Pathogenic 0* (Variation ID: 29860)	21852578; 21881205; 23942189; 23942189; 26524591; 36211342	Classical PRAAS
PSMB8	chr6:g.32841648C>G	NM_148919.4:c.625G>C	p.(Gly209Arg)	VUS 2* (Variation ID: 1034913)	This study; 41253591	PRAAS-ID
PSMB8	chr6:g.32841569G>T	NM_148919.4:c.704C>A	p.(Ala235Asp)	-	This study	PRAAS-ID
PSMB8	chr6:g.32841546C>T	NM_148919.4:c.727G>A	p.(Gly243Arg)	VUS 1* (Variation ID: 1431256)	This study	PRAAS-ID
PSMB9	chr6:g.32858440G>A	NM_002800.5:c.467G>A	p.(Gly156Asp)	Pathogenic 0* (Variation ID: 1299361)	34819510	PRAAS-ID
PSMB9	chr6:g.32858467G>A	NM_002800.5:c.494G>A	p.(Gly165Asp)	Conflicting classifications of pathogenicity 1* (Variation ID: 548995)	26524591	Classical PRAAS
PSMB10	chr16:g.67936291C>G	NM_002801.4:c.166G>C	p.(Asp56His)	Pathogenic 0* (Variation ID: 3241970)	38503300	PRAAS-ID
PSMB10	chr16:g.67935478C>T	NM_002801.4:c.500G>A	p.(Gly167Asp)	Pathogenic 0* (Variation ID: 3241965)	37600812	Classical PRAAS
PSMB10	chr16g.67934906C>G	NM_002801.4:c.601G>C	p.(Gly201Arg)	Pathogenic 0* (Variation ID: 3241969)	38503300	PRAAS-ID

a: ClinVar: assessed on 24 October 2025

Table S2. Site-directed mutagenesis oligonucleotides

	Name	Sequence	Length bp	Tm °C
1	1PSMB8_c.269C>T_S90F_for	GCA GTG GAT TTT CGG GCC TCA G	22	67.2
	1PSMB8_c.269C>T_S90F-rev	CTG AGG CCC GAA AAT CCA CTG C	22	67.2
2	2PSMB8_c.272G>C_R91P-for	GTG GAT TCT CCG GCC TCA GCT G	22	68.2
	2PSMB8_c.272G>C_R91P-rev	CAG CTG AGG CCG GAG AAT CCA C	22	68.2
3	3PSMB8_c.625G>A_G209R-for	CTT ATG CCT ACA GGG TCA TGG AC	23	64
	3PSMB8_c.625G>A_G209R-rev	GTC CAT GAC CCT GTA GGC ATA AG	23	64
4	4PSMB8_c.704C>A_A235D-for	CTA TTG CTT ATG ACA CTC ACA GAG AC	26	62.9
	4PSMB8_c.704C>A_A235D-rev	GTC TCT GTG AGT GTC ATA AGC AAT AG	26	62.9
5	5PSMB8_c.727G>A_G243R-for	GAC AGC TAT TCT AGA GGC GTT GTC	24	64.2
	5PSMB8_c.727G>A_G243R_rev	GAC AAC GCC TCT AGA ATA GCT GTC	24	64.2
6	6PSMB8_c.224C>T_T75M-for	CATGGCACCACCATGCTCGCCTTCAAG	27	69.5
	6PSMB8_c.224C>T_T75M-rev	CTTGAAGGCGAGCATGGTGGTGCCATG	27	69.5
7	7PSMB8_c.274G>A_A92T-for	GTGGATTCTCGG**A**CCTCAGCTG	22	64
	7PSMB8_c.274G>A_A92T-rev	CAGCTGAGG**T**CCGAGAATCCAC	22	64
8	8PSMB8_c.275C>T_A92V-for	GTGGATTCTCGGG**T**CTCAGCTG	22	64
	8PSMB8_c.275C>T_A92V-rev	CAGCTGAG**A**CCCGAGAATCCAC	22	64
9	9PSMB8_c.280G>C_A94P-for	GATTCTCGGGCCTCA**C**CTGGGTCC	24	69.6
	9PSMB8_c.280G>C_A94P-rev	GGACCCAG**G**TGAGGCCCGAGAATC	24	69.6

Table S3. Quantitative reverse transcription PCR primer sequences

Target	Alternative names	Forward primer	Reverse primer
SDHA		CTGTCTTCATACGCTTCTGCACTC	CCAGCCACTAGGTGCCAATC
HSPA5	BiP, GRP78	TGTTCAACCAATTATCAGCAAACTC	TTCTGCTGTATCCTCTTCACCAGT
sXBP1	Spliced XBP1	CTGAGTCCGAATCAGGTGCAG	ATCCATGGGGAGATGTTCTGG
ATF4		GTTCTCCAGCGACAAGGCTA	ATCCTGCTTGCTGTTGTTGG
DDIT3	CHOP	AGAACCAGGAAACGGAAACAGA	TCTCCTTCATGCGCTGCTTT
HSP90B1	GRP94	GAAACGGATGCCTGGTGG	GCCCCTTCTTCCTGGGTC

Table S4. Clinical characteristics of included individuals

In separate file

Table S5. Laboratory findings of included individuals

In separate file

Table S6. Quantification of soluble serum factors in individual 5 and 7.

Analyte	Individual 5				Individual 7	
	Detection range (pg/mL)	Healthy controls median (pg/mL)	Healthy controls range (pg/mL)	Value (pg/mL)	Reference range (pg/mL)	Value (pg/ml)
BCA1 (CXCL13)	0.64-7236	12.9	5.37-20.8	**81.6**	–	–
MIP-4 (CCL18)	1.58-29290	5751	2820-13276	**>29290**	–	–
GM-CSF	1.20-21428	3.39	<1.20-59.9	2.46	–	–
IFNγ	1.98-43870	38.7	2.20-152	**252**	0-0.2	**12.2**
IL-1β	1.88-25072	<1.88	<1.88-25.6	<1.88	0-5.7	**8.6**
IL-2	0.57-9931	<0.57	<0.57-61.0	<0.57	–	–
sIL-2Rα	31.9-499286	285	141-671	**3256**	–	–
IL-4	0.70-10000	27.3	21.2-83.1	24.9	–	–
IL-5	0.64-9961	1.52	<0.64-20.1	1.52	0-0.3	**4**
IL-6	0.67-9988	<0.67	<0.67-8.93	**20.8**	0.5-2.2	**15.7**
IL-7	0.64-10249	2.85	<0.64-11.2	4.18	–	–
IL-8	0.70-9298	5.38	1.68-10.2	**51.2**	–	–
sVEGFR-2	156-2526362	6010	4763-7791	**10012**	–	–
sVCAM-1	7.72-124288	31252	22808-51553	**>124288**	–	–
IL-17A	3.44-10010	<3.44	<3.44	<3.44	–	–
IL-21	60.8-999925	154	<60.8-12874	182	–	–
sTNFR-2	16.0-216001	2618	1488-4492	**7245**	–	–
IL-18	0.59-10027	19.3	8.94-46.0	**1214**	–	–
VEGF-D	45.4-25000	235	47.0-343	82.8	–	–
IP-10 (CXCL10)	1.26-3953	29.9	13.8-63.4	**752**	0-104	**2190**
MIP-3 (CCL23)	0.30-4685	35.0	16.0-80.0	**>4685**	–	–
MIG (CXCL9)	1.62-19669	66.1	22.2-151	**897**	23-220	**3839**
RANTES (CCL5)	0.53-9707	1917	843-4136	933	–	–
TNFα	3.21-48196	13.3	5.58-33.2	24.5	0.2-5.6	5
PF4 (CXCL4)	1480-1057120	9615	2388-17091	5279	–	–
Endostatin	1.00-4997	103	85-159	100	–	–
TIMP-1	239-181547	94292	76761-148602	90945	–	–
Eotaxin	–	–	–	–	55-220	50.5

Substantial differences of soluble factors in the serum are presented as >1.5x upper limit of normal and highlighted in bold.

[a]Serum of healthy controls was used as reference (n=13). Quantification is expressed as mean value (pg/mL) and range of the lower and upper limit of normal (min-max).

Table S7. Candidate gene variants and rare variants in proteasome subunits

Family	Individual	Gene	HGVSg (hg38)	HGVSc	HGVSp	Zygosity	Segregation	CADD v1.6	AF GnomAD v4.0.0	Comment
Family 1	Individual 1	*PSMB8*	chr6:g.32842968G>T	NM_148919.4:c.269C>T	p.(Ser90Phe)	Heterozygous	*De novo*	28.2	Absent	
		IL25	chr14:g.23375644C>T	NM_022789.3:c.298C>T	p.(Arg100Trp)	Homozygous	PV MV	30	0.00056322 (1x homozygote)	
		GMPPB	chr3:g.49722056C>T	NM_013334.3:c.860G>A	p.(Arg287Gln)	Heterozygous	PV	20.9	0.00017912 (0x homozygote)	No second variant on other allele
		HS6ST2	chrX:g.132958353C>G	NM_001077188.1:c.250G>C	p.(Ala84Pro)	Hemizygous	MV	21.7	Absent	Does not fit phenotype
Family 2	Individual 2 and 3	*PSMB8*	chr6:g.32842965C>G	NM_148919.4:c.272G>C	p.(Arg91Pro)	Heterozygous	MV (mosaic)	32	Absent	
Family 3	Individual 4	*PSMB8*	chr6:g.32841648G>A	NM_148919.4:c.625G>A	p.(Gly209Arg)	Heterozygous	*De novo*	29.1	Absent	
Family 4	Individual 5	*PSMB8*	chr6:g.32841569G>T	NM_148919.4:c.704C>A	p.(Ala235Asp)	Heterozygous	*De novo*	29.7	Absent	
		CLPB	chr11:g.72372971C>T	NM_030813.6:c.690G>A	p.(Trp230*)	Heterozygous	PV	38	0.00001115 (0x homozygote)	No second variant on other allele
Family 5	Individual 6	*PSMB8*	chr6:g.32841546C>T	NM_148919.2:c.727G>A	p.(Gly243Arg)	Heterozygous	*De novo*	31	Absent	
		PAPPA	chr9:g.116187628G>C	NM_002581.5:c.890G>C	p.(Trp297Ser)	Heterozygous	*De novo*	29.2	0.0000006195	Present in unaffected child
	Individual 7	*PSMB8*	chr6:g.32841546C>T	NM_148919.2:c.727G>A	p.(Gly243Arg)	Heterozygous	MV	31	Absent	
		PAPPA	chr9:g.116187628G>C	NM_002581.5:c.890G>C	p.(Trp297Ser)	Heterozygous	MV	29.2	0.0000006195	Present in unaffected sibling

LP: likely pathogenic; MV: maternal variant; P: pathogenic; PV: paternal variant

Table S8. Frustration index and proteasomal contacts across wild-type and mutant variants

In separate file

Table S9. List of significantly upregulated or downregulated proteins in the p.Ala235Asp cell line

In separate file

Table S10. Enriched Gene Ontology (GO) terms and Reactome pathways in upregulated and downregulated protein sets

In separate file

Table S11. Predicted structural and biophysical effects of paralogous variants in immunoproteasome β-subunits (*PSMB8*, *PSMB9* and *PSMB10*).

Gene	Variant	Described pathogenic	gnomAD v4.1.0	RSA	Rate4Site	ΔΔG	Δ contacts	FI switch
PSMB8	**p.Asp89His**	No	Absent	0	-0.802	39.3	9	Neutral
PSMB9	**p.Asp37His**	No	Absent	0	-0.032	35.0	1	Neutral
PSMB10	**p.Asp56His**	Yes	Absent	0	-0.792	26.8	4	High to neutral
PSMB8	**p.Ser90Phe**	Yes	Absent	0.4	-0.818	13.1	10	Neutral to minimal
PSMB9	**p.Ser38Phe**	No	Absent	0	-0.217	89.8	12	Neutral to minimal
PSMB10	**p.Thr57Phe**	No	Absent	4.3	-0.826	6.4	9	Neutral to minimal
PSMB8	**p.Arg91Pro**	Yes	Absent	1.3	-0.801	23.4	13	Neutral to high
PSMB9	**p.Arg39Pro**	No	Absent	5.9	0.332	18.4	3	Neutral to high
PSMB10	**p.Arg58Pro**	No	Absent	2.3	-0.502	21.4	20	Neutral to high
PSMB8	**p.Gly209Arg**	Yes	Absent	0	-0.769	46.4	14	Neutral
PSMB9	**p.Gly156Arg**	No	Absent	0	-0.626	52.5	46	Neutral
PSMB10	**p.Ala175Arg**	No	Absent	0	-0.046	21.8	39	Neutral to minimal
PSMB8	**p.Gly209Asp**	No	Absent	0	-0.769	34.8	43	Neutral to high
PSMB9	**p.Gly156Asp**	Yes	Absent	0	-0.626	23.1	8	Neutral to high
PSMB10	**p.Ala175Asp**	No	Absent	0	-0.046	12.6	9	Neutral to high
PSMB8	**p.Ala235Asp**	Yes	Absent	0	-0.725	26.0	18	Neutral to high
PSMB9	**p.Ala182Asp**	No	Absent	0	-0.473	23.0	28	Neutral to high
PSMB10	**p.Gly201Asp**	No	Absent	0	-0.706	30.4	30	Neutral to high
PSMB8	**p.Ala235Arg**	No	Absent	0	-0.725	48.4	14	Neutral
PSMB9	**p.Ala182Arg**	No	Absent	0	-0.473	28.7	30	Neutral
PSMB10	**p.Gly201Arg**	Yes	Absent	0	-0.706	54.6	20	Neutral
PSMB8	**p.Gly243Arg**	Yes	Absent	0	-0.767	35.2	11	Neutral
PSMB9	**p.Gly190Arg**	No	74x	5.1	-0.259	15.7	1	Neutral
PSMB10	**p.Gly209Arg**	No	Absent	0	-0.706	26.6	12	Neutral

RSA, relative solvent accessibility; Rate4Site, evolutionary conservation score; ΔΔG, predicted change in protein stability (FoldX); Δ contacts, change in residue interaction network; FI, frustration index; FI switch, change in local frustration state; gnomAD v4.1.0, Genome Aggregation Database.

Table S12. Identified proteasome subunits and associated subcomplexes detected in control samples from THP1, 143B, and fibroblast cell lines

In separate file

Supplemental Methods

Recruitment and ethics approvals

Family 1 was identified in the Undiagnosed Diseases Network (UDN) study, a multicenter study approved by the National Institutes of Health IRB (15HG0130).

Family 2 was enrolled under the Immunology Biorepository #667 protocol for research genomic sequencing and formal case reporting of their medical course which was approved by Seattle Children's Hospital Institutional Review board.

Family 3 was tested clinically through the Victorian Clinical Genetics Services, Melbourne, Victoria.

Family 4 was part of the Radboud Data- and Biobank for genetics and rare diseases and was enrolled in an international research study to diagnose the undiagnosed at the Radboud University Medical Center (Radboudumc) under a protocol approved by the Institutional Review Board of CMO Radboudumc and METC East Nijmegen, the Netherlands (2018-4985 and 2019-5554).

Family 5 was recruited under the whole exome and whole genome sequencing program for undiagnosed diseases of Gaslini Children's Hospital and Italian Institute of Technology under the protocol approved by the Regione Liguria Institutional Review Board.

Cell culturing

Human osteosarcoma 143B cells (cell line GM05887, Coriell Institute for Medical Research) were cultured in Dulbecco's Modified Eagle's Medium (DMEM, Gibco 41-965-039) supplemented with 10% fetal bovine serum (FBS-11A, Capricorn scientific) and 1% antibiotic/antimycotic solution (30-004-CL, Corning) in a humidified, 5% CO_2 atmosphere at 37°C.

Human monocytic cell line THP-1 (ATCC® TIB202™) was cultured in RPMI-1640 supplemented with 10% FBS-11A, 100 U/ml penicillin, 100 µg/ml streptomycin, 250 n/ml amphotericin B and 0.05 mM β-mercaptoethanol in a humidified, 5% CO_2 atmosphere at 37°C. THP-1 monocytes were differentiated with 10 ng/ml phorbol 12-myristate 13-acetate for 48 h and macrophages were harvested by trypsinization.

Human skin fibroblasts were cultured in DMEM (Gibco) supplemented with 10% FCS (Capricorn scientific) and antibiotic/antimycotic solution in a humidified, 5% CO_2 atmosphere at 37 °C. To enhance the expression of immunoproteaseome-specific subunits, cells were stimulated for 48 h with 250 U/ml human recombinant IFNγ (ImmunoTools, Friesoythe, Germany). Cells from four 175-cm^2 flasks were harvested by trypsinization, washed with PBS and centrifuged at 1000 *g* for 5 min at 4°C.

Protein extraction and immunoblotting

To maintain the activity of proteasomes, cells were lysed in TSDG buffer (10 mM Tris pH7.5, 10 mM NaCl, 25 mM KCl, 1 mM $MgCl_2$, 0.1 mM EDTA, 10% glycerol, 1 mM DTT, 2mM ATP in H_2O) with seven freeze-thaw cycles consisting of rapid freezing in an ethanol/dry ice bath for 3 minute followed by thawing in a 37 °C water bath for 3 minutes, ensuring complete thawing in each cycle. Afterwards, a centrifugation for 10 minutes with 16000 *g* at 4 °C was performed, and supernatants were used for immunoblotting and proteasome activity assays.

Cell lysates containing 10 µg proteins were denatured in 5x reducing loading buffer (250 mM Tris pH6.8, 0.5 M DTT, 10% SDS, 50% glycerol, 0.25% bromophenol blue in H_2O) for 10 minutes at 95 °C. Afterwards, samples were separated on 4-15% TGX stain-free gels (Bio-Rad, 5678085) in SDS-PAGE running buffer (25 mM Tris, 192 mM glycine, 0.1 % SDS in H_2O, pH8.6). After electrophoresis, proteins were visualized by stain-free staining under UV-light as loading control. Proteins were then transferred on ethanol-activated PVDF membranes with the TransBlot Turbo System. The transfer was carried out at constant 25 V for 30 minutes in transfer buffer (25 mM Tris, 192 mM glycine, 20% ethanol in H_2O, pH8.6). After the transfer, total protein staining was performed by staining membranes in 0.2% Ponceau-S staining solution for 10 minutes. Membranes were then blocked in 3% non-fat dry milk (NFDM)/TBS-T for 1 hour at RT, followed by primary antibody incubation overnight at 4 °C. Antibodies against PSMB5 (Thermo Fisher Scientific PA1-977), PSMB8 (Abcam ab3329), PSMA3 (Santa Cruz Biotechnology Inc. Sc-166205), PSMB9 (Abcam ab184172), Ubiquitin (linkage-specific K48; Abcam 140601), V5 tag (Thermo Fisher Scientific, R960-25) and GAPDH (Santa Cruz Biotechnology, sc-47724) were used. Subsequent incubation with HRP-coupled secondary antibodies (1:10,000) in 3% NFDM/TBS-T was performed at RT for 1 hour. Membranes were then incubated in ECL SuperSignal for 5 minutes at RT, and the signal was developed with an imager (Fusion FX7, Vilber). When reprobing was needed, HRP activity of the former secondary antibody was inhibited by incubating membranes in 0.01 M para-toluene sulfonic acid (PTSA) twice for 5 minutes before the next primary antibody incubation.

In-gel proteasome proteolytic activity assay

The chymotrypsin-like activity of proteasome was assessed with an in-gel fluorescence technique. Native cell lysates with 15 µg protein were mixed with 5x native loading buffer (250 mM BisTris pH6.5, 250 mM NaCl, 50% glycerol, 0.25% bromophenol blue in H_2O). Without heating, the mixture was resolved using a 3-12% Bis-Tris native gel (Thermo Fisher, #BN1003BOX). After 3-hour electrophoresis run at constantly 150-200 V in native running buffer (50 mM BisTris, 50 mM Tricin, 0.4 mM ATP, 2 mM $MgCl_2$, 0.5 mM DTT in H_2O), gels were incubated with proteasome activity assay buffer (20 mM Tris pH7.4, 5 mM $MgCl_2$, 2 mM ATP, 100 µM Ac-ANW-AMC in H_2O) for 20 minutes at

37 °C. The chymotrypsin-like activity of proteasomal complexes is reflected by the cleavage of the Suc-LLVY-AMC substrate and the release of fluorescent free aminomethylcumarin (AMC), which could be measured by an imager (Fusion FX7, Vilber) using excitation at 365 nm and detection at 450 nm. The gel was subsequently blotted on a PVDF membrane, which was further stained with RubyStain total protein stain (Thermo Fisher Scientific, V10309) for loading control. The proteasome α7 subunit (PSMA3) was detected as a measure of proteasome abundance for normalization of proteolytic activity. PSMB5 and PSMB8 were detected as a measure of constitutive and immunoproteasome abundance, respectively.

Active proteasome subunit abundance assay

The amount of active proteasome catalytic subunits was assessed with activity-based probes (ABPs), which are composed of a reactive group, a recognition element, and a reporter tag. ABPs covalently attach to the active sites of proteasomal catalytic subunits, enabling their detection via the reporter. Pan-ABPs (cy5-epoxomicin) targeting all proteolytic β-subunits (kindly provided by Dr. B.I. Florea) were used in this study. Specifically, 10 µg native cell lysates were incubated with 0.5 µM cy5-epoxomicin for 1 hour at 37 °C. All reactions were performed in TSDG buffer. After incubation with ABPs, samples were solubilized with the 5x loading buffer (250 mM Tris pH6.8, 0.5 M DTT, 10% SDS, 50% glycerol, 0.25% bromophenol blue in H_2O) for 10 minutes at 70 °C. Samples were subsequently resolved by SDS-PAGE on 12.5% tris-glycine gels with 3.3% crosslinker (acrylamide:bisacrylamide 29:1). Electrophoresis was carried out at 120-150 V until the 17 kDa band of the protein ladder reached the gel bottom to get the optimal separation of proteasome catalytic subunits. After separation, fluorescence detection was performed at at Ex/Em = 650/670 nm for cy5-epoxomicin using the imager (Fusion FX7, Vilber) and was followed by subsequent blotting of the gel to determine total expression of proteasome subunits of interest for normalization.

Complexome profiling

<u>Cell fractionation</u>

Fibroblast, 143B and THP-1 cell pellets were resuspended in 4 ml ice-cold homogenization buffer (250 mM sucrose, 1 mM EDTA, 20 mM Tris/HCl, pH 7.4) and disrupted mechanically by 15 strokes, passing the cell suspension through a 20 G needle fixed to a 5-ml syringe on ice.

Fibroblasts cell homogenates were centrifuged at 1000 *g* for 10 min at 4°C and the supernatants were centrifuged at 21000 *g* for 10 min at 4°C. Pellets were resuspended in homogenization buffer and the protein concentration was determined by Lowry. Suspension aliquots containing 200 µg protein were centrifuged at 21000 *g* for 20 min at 4°C. Supernatants were discarded and the pellets were shock frozen in liquid nitrogen and stored at -80°C.

143B cell homogenates were centrifuged at 1000 *g* for 10 min at 4°C and the supernatant was centrifuged at 6000 *g* for 10 min at 4°C. Pellets were resuspended in homogenization buffer and the protein concentration was determined by Lowry.

THP-1 cell homogenates were centrifuged at 1000 *g* for 10 min at 4°C and the protein concentration was determined from the supernatants.

<u>Blue-Native PAGE and cutting</u>

Samples containing 200 µg of protein were thawed and resuspended at 10 mg protein/ml in solubilization buffer (0.5 M 6-aminohexanoic acid, 1 mM EDTA, 50 mM imidazole/HCl, pH 7.0) and solubilized with 6 g digitonin/g protein. Protein solutions were centrifuged at 22000 *g* for 20 min at 4°C and protein concentration of the supernatant was determined by Lowry. An aliquot containing 0.1 mg protein was mixed with 5% Coomassie blue (Serva Blue G) in 0.5 M aminohexanoic acid and loaded on a 4-16% polyacrylamide gradient gel. Proteins were separated by gel electrophoresis at 4°C as described previously.[4] After electrophoresis, gels were fixed in 50% methanol, 10% acetic acid, 10 mM ammonium acetate and stained with Coomassie blue (Figure S18A). Gels were destained in 10% acetic acid, washed with water, documented and each lane was cut into 60 even pieces and transferred to a 96-well MultiScreen-BV. 1.2 µm filter plate (Millipore).

<u>In-gel digestion</u>

Gel pieces were destained for 30 min in 50% methanol, 50 mM ammonium bicarbonate (ABC) at room temperature and centrifuged at 600 *g* for 2 min. This step was repeated 3-4 times until the Coomassie blue dye was removed entirely. Then, gel pieces were incubated for 1 h in 5 mM dithiothreitol, 50 mM ABC followed by 45 min incubation with 15 mM 2-chloroacetamide, 50 mM ABC. After washing once with 50% methanol, 50 mM ABC, gel pieces were let dry at room temperature for 30 min and rehydrated with 20 µl of 5 µg trypsin/ml in 50 mM ABC for 30 min at 4°C. After adding 50 µl 50 mM ABC, plates were sealed and incubated overnight at 37°C. Peptide solutions were transferred to a PCR plate by centrifugation at 600 *g* for 2 min. The gel pieces were washed once with 50% acetonitrile, 5% formic acid and the filtrates were collected into the same PCR plate. Peptide solutions were dried for 2.5 h at 45°C in a centrifuge concentrator (Concentrator plus, Eppendorf). Peptides were dissolved in 20 µl 0.1% formic acid.

<u>LC-MS/MS</u>

From fibroblasts samples, 2 µl were injected and subjected to liquid chromatography tandem mass spectrometry (LC-MS/MS) on a quadrupole-orbitrap hybrid orbitrap mass spectrometer (Exploris 480, Thermo Fisher Scientific) coupled at the front end to an ultra-high pressure liquid

chromatography system (Vanquish neo UHPLC System, Thermo Fisher). Attached to the UHPLC was a peptide trap (100 µm x 20 mm, 100 Å pore size, 5 µm particle size, C18, Nano Viper, Thermo Fisher) for online desalting and purification, followed by a 25 cm C18 reversed-phase column (75 µm x 250 mm, 130 Å pore size, 1.7 µm particle size, peptide BEH C18, nanoEase, Waters). Peptides were separated using a 35 min method with linearly increasing ACN concentration from 2% to 30% ACN over 25 minutes.

MS/MS measurements from fibroblasts samples were performed on a quadrupole-orbitrap hybrid mass spectrometer (Exploris 480, Thermo Fisher Scientific). Eluting peptides were ionized using a nano-electrospray ionization source (nano-ESI) with a spray voltage of 1,800 V and analyzed in data-independent acquisition (DIA) mode. For each MS1 scan, ions were accumulated for a maximum of 240 ms or until a charge density of 3 x 10^6 ions (AGC Target) was reached. Fourier-transformation based mass analysis of the data from the orbitrap mass analyzer was performed covering a mass range of m/z 400 – 1,400 with a resolution of 120,000 at m/z 200. Within a precursor mass range of m/z 380-980 fragmentation in DIA-mode with m/z 12 isolation windows and m/z 1 window overlaps was performed. Fragmentation was performed at normalized collision energy of 28% using higher energy collisional dissociation (HCD). An AGC target of 2 x 10^6 ions or a maximum of 54 ms was set. Orbitrap resolution was set to 30,000 with a scan range from m/z 350-2000.

From 143B and THP-1 samples, 5 µl of the peptide solutions were analyzed by liquid chromatography electrospray ionization tandem mass spectrometry (LC-ESI-MS/MS) in a Q-Exactive mass spectrometer (Thermo Fisher Scientific) equipped with an Easy nLC1000 nano-flow high-performance liquid chromatography system at the front end. Peptide separation was performed with an emitter column (15cm L x 100 µm ID x 360 µm OD x 15 µm orifice; MSWil, CoAnn Technologies, LLC) filled with ReproSil-Pur C18-AQ reverse phase beads (3 µm particle size, 120 Å pore size; Dr. Maisch GmbH) using a 30 min linear gradient of 5 to 35% acetonitrile with 0.1% formic acid. The mass spectrometer operated in positive ion switching automatically between MS and data dependent MS/MS, fragmenting the twenty most intense ions per precursor scan. Full scan MS mode (400 to 1400 m/z) was operated with automatic gain control target of 1×10^6 ions, 70000 resolution and a maximum ion transfer of 20 ms. Selected ions for MS/MS were analyzed using the following parameters: resolution 17,500; AGC target of 1×10^5; maximum ion transfer of 50 ms; 4.0 m/z isolation window and dynamic exclusion of 30.0 s was used.

Proteomics data analysis

LC-MS/MS data from fibroblasts were searched with the CHIMERYS DIA algorithm integrated into the Proteome Discoverer software (v3.1.0.638, Thermo Fisher Scientific) against a reviewed human

Swissprot database (obtained November 2023) using Inferys 3.0 fragmentation as prediction model. Carbamidomethylation was set as a fixed modification for cysteine residues. The oxidation of methionine was allowed as a variable modification. A maximum number of one missing tryptic cleavage was set. Peptides between 7 and 30 amino acids were considered. A strict cutoff (FDR < 0.01) was set for peptide identification. Quantification was performed by CHIMERYS based on fragment ions. The mass spectrometry proteomics data have been deposited to the ProteomeXchange Consortium via the PRIDE partner repository with the dataset identifier PXD064505.[5]

A new database search was performed to identify the PSMB8 variant A235D and S90F. To this end, the raw data were searched with the DIA-NN algorithm (Version 1.9.1) against a human database (obtained November 2023) which included the PSMB8 variants.[6] The oxidation of methionine, the N-terminal methionine excision, and the acetylation of the protein N-terminus were allowed as variable modifications. A maximum number of two missing tryptic cleavages was set. Peptides between 7 and 30 amino acids were considered. A strict cutoff (FDR < 0.01) was set for peptide identification in double pass mode. Match between runs was activated.

LC-MS/MS raw files from 143B and THP-1 samples were analyzed using MaxQuant 1.5.0.25, and 1.6.17.0, respectively. 143B spectra were matched against the human NCBI Reference Sequence Database release 55 with reverse decoy and a false discovery rate of 0.01. THP-1 spectra were marched against the Uniprot database of canonical isoforms downloaded on May 2021.

Protein groups text files containing the abundance values of each protein across all gel fractions were subjected to hierarchical clustering analysis by uncentered Pearson correlation with average linkage using Cluster 3.0.[7] Protein migration profiles were visualized using NOVA v0.5.7 and the differences of the protein migration profiles between controls and patient fibroblasts were scored by Hausdorff distance calculations using COPAL as described previously.[8,9] Protein migration profiles of control and patient fibroblasts were uploaded to the complexome profiling data resource, CEDAR, accession number (CRX49).[10] A set of soluble and membrane protein complexes with established molecular mass and stoichiometry were used as standards to estimate the molecular masses of globular (hydrophilic) and transmembrane (hydrophobic) protein complexes in each gel fraction, respectively (Figure S18B).

Genome sequencing and variant analysis

Family 1

Family 1 underwent trio genome sequencing through the UDN Sequencing Core at Baylor Genetics as previously described, and research reanalysis was performed at the BCM UDN site.[11] The research genome reanalyzes prioritized rare, *de novo* and biallelic variants that had an allele frequency of < 1% in gnomAD v4.0.0 and in the BCM UDN internal sequencing database (> 1200 samples). Codified Genomics was used for variant filtering and prioritization.

Family 2

The exome sequencing was clinically performed using enrichment capture kit from IDT xGen Exome v1 and v2 from extracted genomic DNA isolated from frozen brain tissue for individual 4 and buccal swabs for the unaffected parents. The enriched targets were simultaneously sequenced with paired-end reads on an Illumina platform. Bi-directional sequence reads were assembled and aligned to reference sequences based on NCBI RefSeq transcripts and human genome build GRCh37/UCSC hg19. Initial analysis was performed using custom-developed proprietary analysis tool GeneDx's XomeAnalyzer (a variant annotation, filtering, and viewing interface for WES data). Further analysis of the proteosome genes were also performed manually using the Integrative Genomics Viewer (IGV).

Family 3

Clinically accredited trio genome sequencing was performed at the Victorian Clinical Genetics Services, Melbourne, Australia as previously described and identified a *de novo* heterozygous missense variant in *PSMB8*, NM_148919.4(PSMB8): c.625G>A; p.(Gly209Arg).[12]

Family 4

Genome sequencing for the proband was outsourced to the Beijing Genomics Institute (BGI) and performed on a BGISeq500 platform. Paired-end 100 bp reads were generated, achieving a median coverage of 30-fold. Data processing was carried out at the Radboud University Medical Center. Reads were aligned to the GRCh38 reference genome using Bwa-mem2 v2.2.1, and quality control was performed with Qualimap v2.2.1. Single nucleotide variants (SNVs) were called using GATK HaplotypeCaller v3.8. Structural variants (SVs) were detected using Manta v1.1.0 (Illumina), and copy number variants (CNVs) were identified with Canvas v1.40.0 (Illumina). Short tandem repeats (STRs) were analyzed using ExpansionHunter v3.1.2 with default parameters. All variant types (SNVs, SVs, CNVs) were annotated using an in-house pipeline. Rare candidate SNVs were prioritized based on a gnomAD v3.1 and internal frequency of <1%. CNVs and SVs were filtered using a <1% frequency

threshold in the 1000 Genomes database and an internal reference set, requiring a minimum reciprocal overlap of 90%. Additional annotations, including CADD scores, SpliceAI, phyloP, and AlphaMissense, along with the patient's phenotype, were used to prioritize potentially disease-causing variants. Candidate variants were segregated in parental DNA with Sanger sequencing, allowing *de novo* assessment.

Family 5

Genome sequencing was generated on an Illumina NovaSeq 6000 at the Genomics Facility of the Istituto Italiano di Tecnologia (Genoa, Italy) and analyzed by the Clinical Bioinformatics Unit, Istituto Giannina Gaslini (Genoa, Italy). We first interrogated the coding regions of all known autoinflammatory disease (AID) genes (~60, IUIS 2024). No pathogenic variants were identified. Variant filtering used standard criteria: high quality and depth, rarity in population databases (e.g., gnomAD <1%), predicted protein-altering or canonical splice-site effect, inheritance consistency, and exclusion of known benign/common variants.

Interferon (IFN) type I signature

Family 1

The IFN score calculation was adapted with the following modifications.[13] RNA was extracted from cryopreserved peripheral blood mononuclear cells (PBMCs) using the Quick RNA Miniprep Kit (Zymo Research) per manufacturer's instructions, and 33ng converted to cDNA using the GoScript Reverse Transcription System (Promega). Real time PCR was performed using TaqMan Fast Advanced Master Mix and commercially available primer/probe sets (both Applied Biosystems) on a Roche LightCycler 96. The relative abundance of each target transcript was normalized to GAPDH, and data are expressed relative to a single control patient.

Family 3

An IFN-score was calculated based on six IFN-related genes: IF127, IF144L, IFIT1, ISG15, RSAD2, SIGLEC1.

Family 4

Based on the expression levels quantified from PAXgene Blood RNA tubes by RT-PCR of five IFN-related genes (IFI44, IFI44L, IFIT1, IFIT3, and MX1) and a reference gene (ABL) an interferon-type 1 (IFN-1) gene signature score was calculated for each sample, as previously described.[14,15] A value ≥ 9.4 was considered positive.

Family 5

IFN induced gene expression analysis was performed as described by Tesser et al.[16]

Cytokine measurements

Family 4

For cytokine, chemokine and soluble receptor production measurements, serum was collected. Concentrations (pg/mL) of human BCA-1, MIP-3, MIP-4, GM-CSF, IFNγ, IL-1β, IL-2, sIL-2Rα, IL-4, IL-5, IL-6, IL-7, IL-8, sVEGFR-2, sVCAM-1, IL-17A, IL-21, sTNFR2, IL-18, VEGF-D, IP-10, MIG, RANTES, TNFα, PF4, Endostatin, and TIMP-1 were measured according to the manufacturer's instructions. For all factors, measured values below the lower limit of detection are represented by this lowest detection value. Serum analysis was performed using MILLIPLEX® Multiplex Assays (Merck Millipore) using a Flexmap 3D system.

Supplemental References

1. Dong, Y., Zhang, S., Wu, Z., Li, X., Wang, W.L., Zhu, Y., Stoilova-McPhie, S., Lu, Y., Finley, D., and Mao, Y. (2019). Cryo-EM structures and dynamics of substrate-engaged human 26S proteasome. Nature *565*, 49-55. 10.1038/s41586-018-0736-4.
2. Ladi, E., Everett, C., Stivala, C.E., Daniels, B.E., Durk, M.R., Harris, S.F., Huestis, M.P., Purkey, H.E., Staben, S.T., Augustin, M., et al. (2019). Design and Evaluation of Highly Selective Human Immunoproteasome Inhibitors Reveal a Compensatory Process That Preserves Immune Cell Viability. J Med Chem *62*, 7032-7041. 10.1021/acs.jmedchem.9b00509.
3. Huang, X., Luan, B., Wu, J., and Shi, Y. (2016). An atomic structure of the human 26S proteasome. Nat Struct Mol Biol *23*, 778-785. 10.1038/nsmb.3273.
4. Wittig, I., Braun, H.P., and Schagger, H. (2006). Blue native PAGE. Nat Protoc *1*, 418-428. 10.1038/nprot.2006.62.
5. Perez-Riverol, Y., Bandla, C., Kundu, D.J., Kamatchinathan, S., Bai, J., Hewapathirana, S., John, N.S., Prakash, A., Walzer, M., Wang, S., and Vizcaino, J.A. (2025). The PRIDE database at 20 years: 2025 update. Nucleic Acids Res *53*, D543-D553. 10.1093/nar/gkae1011.
6. Demichev, V., Messner, C.B., Vernardis, S.I., Lilley, K.S., and Ralser, M. (2020). DIA-NN: neural networks and interference correction enable deep proteome coverage in high throughput. Nat Methods *17*, 41-44. 10.1038/s41592-019-0638-x.
7. de Hoon, M.J., Imoto, S., Nolan, J., and Miyano, S. (2004). Open source clustering software. Bioinformatics *20*, 1453-1454. 10.1093/bioinformatics/bth078.
8. Giese, K.P., Aziz, W., Kraev, I., and Stewart, M.G. (2015). Generation of multi-innervated dendritic spines as a novel mechanism of long-term memory formation. Neurobiol Learn Mem *124*, 48-51. 10.1016/j.nlm.2015.04.009.
9. Van Strien, J., Guerrero-Castillo, S., Chatzispyrou, I.A., Houtkooper, R.H., Brandt, U., and Huynen, M.A. (2019). COmplexome Profiling ALignment (COPAL) reveals remodeling of mitochondrial protein complexes in Barth syndrome. Bioinformatics *35*, 3083-3091. 10.1093/bioinformatics/btz025.
10. van Strien, J., Haupt, A., Schulte, U., Braun, H.P., Cabrera-Orefice, A., Choudhary, J.S., Evers, F., Fernandez-Vizarra, E., Guerrero-Castillo, S., Kooij, T.W.A., et al. (2021). CEDAR, an online resource for the reporting and exploration of complexome profiling data. Biochim Biophys Acta Bioenerg *1862*, 148411. 10.1016/j.bbabio.2021.148411.
11. Keehan, L., Jiang, M.M., Li, X., Marom, R., Dai, H., Murdock, D., Liu, P., Hunter, J.V., Heaney, J.D., Robak, L., et al. (2021). A novel de novo intronic variant in ITPR1 causes Gillespie syndrome. Am J Med Genet A *185*, 2315-2324. 10.1002/ajmg.a.62232.
12. Lunke, S., Bouffler, S.E., Patel, C.V., Sandaradura, S.A., Wilson, M., Pinner, J., Hunter, M.F., Barnett, C.P., Wallis, M., Kamien, B., et al. (2023). Integrated multi-omics for rapid rare disease diagnosis on a national scale. Nat Med *29*, 1681-1691. 10.1038/s41591-023-02401-9.
13. Rice, G.I., Forte, G.M., Szynkiewicz, M., Chase, D.S., Aeby, A., Abdel-Hamid, M.S., Ackroyd, S., Allcock, R., Bailey, K.M., Balottin, U., et al. (2013). Assessment of interferon-related biomarkers in Aicardi-Goutieres syndrome associated with mutations in TREX1, RNASEH2A, RNASEH2B, RNASEH2C, SAMHD1, and ADAR: a case-control study. Lancet Neurol *12*, 1159-1169. 10.1016/S1474-4422(13)70258-8.
14. Huijser, E., Bodewes, I.L.A., Lourens, M.S., van Helden-Meeuwsen, C.G., van den Bosch, T.P.P., Grashof, D.G.B., van de Werken, H.J.G., Lopes, A.P., van Roon, J.A.G., van Daele, P.L.A., et al. (2022). Hyperresponsive cytosolic DNA-sensing pathway in monocytes from primary Sjogren's syndrome. Rheumatology (Oxford) *61*, 3491-3496. 10.1093/rheumatology/keac016.
15. Bodewes, I.L.A., Al-Ali, S., van Helden-Meeuwsen, C.G., Maria, N.I., Tarn, J., Lendrem, D.W., Schreurs, M.W.J., Steenwijk, E.C., van Daele, P.L.A., Both, T., et al. (2018). Systemic interferon

type I and type II signatures in primary Sjogren's syndrome reveal differences in biological disease activity. Rheumatology (Oxford) *57*, 921-930. 10.1093/rheumatology/kex490.
16. Tesser, A., Bocca, P., Ulivi, M., Pin, A., Pastorino, C., Cangelosi, D., Santori, E., Drago, E., Caorsi, R., Candotti, F., et al. (2025). Type I interferon signature: a quantitative standardized method for clinical application. Clin Exp Immunol *219*. 10.1093/cei/uxaf018.

Members of the Undiagnosed Diseases Network (Version 3.31.25)

Alyssa A. Tran, Arjun Tarakad, Ashok Balasubramanyam, Brendan H. Lee, Carlos A. Bacino, Daryl A. Scott, Elaine Seto, Gary D. Clark, Hongzheng Dai, Hsiao-Tuan Chao, Ivan Chinn, James P. Orengo, Jennifer E. Posey, Jill A. Rosenfeld, Kim Worley, Lindsay C. Burrage, Lisa T. Emrick, Lorraine Potocki, Monika Weisz Hubshman, Richard A. Lewis, Ronit Marom, Seema R. Lalani, Shamika Ketkar, Tiphanie P. Vogel, William J. Craigen, Jared Sninsky, Lauren Blieden, Sandesh Nagamani, Hugo J. Bellen, Michael F. Wangler, Oguz Kanca, Shinya Yamamoto, Christine M. Eng, Patricia A. Ward, Pengfei Liu, Adeline Vanderver, Cara Skraban, Edward Behrens, Gonench Kilich, Kathleen Sullivan, Kelly Hassey, Ramakrishnan Rajagopalan, Rebecca Ganetzky, Vishnu Cuddapah, Anna Raper, Daniel J. Rader, Giorgio Sirugo, Vaidehi Jobanputra, Allyn McConkie-Rosell, Kelly Schoch, Mohamad Mikati, Nicole M. Walley, Rebecca C. Spillmann, Vandana Shashi, Alan H. Beggs, Calum A. MacRae, David A. Sweetser, Deepak A. Rao, Edwin K. Silverman, Elizabeth L. Fieg, Frances High, Gerard T. Berry, Ingrid A. Holm, J. Carl Pallais, Joan M. Stoler, Joseph Loscalzo, Lance H. Rodan, Laurel A. Cobban, Lauren C. Briere, Matthew Coggins, Melissa Walker, Richard L. Maas, Susan Korrick, Jessica Douglas, Cecilia Esteves, Emily Glanton, Isaac S. Kohane, Kimberly LeBlanc, Rachel Mahoney, Shamil R. Sunyaev, Shilpa N. Kobren, Brett H. Graham, Erin Conboy, Francesco Vetrini, Kayla M. Treat, Khurram Liaqat, Lili Mantcheva, Stephanie M. Ware, Breanna Mitchell, Brendan C. Lanpher, Devin Oglesbee, Eric Klee, Filippo Pinto e Vairo, Ian R. Lanza, Kahlen Darr, Lindsay Mulvihill, Lisa Schimmenti, Queenie Tan, Surendra Dasari, Abdul Elkadri, Brett Bordini, Donald Basel, James Verbsky, Julie McCarrier, Michael Muriello, Michael Zimmermann, Adriana Rebelo, Carson A. Smith, Deborah Barbouth, Guney Bademci, Joanna M. Gonzalez, Kumarie Latchman, LéShon Peart, Mustafa Tekin, Nicholas Borja, Stephan Zuchner, Stephanie Bivona, Willa Thorson, Herman Taylor, Rakale C. Quarells, Ayuko Iverson, Bruce Gelb, Charlotte Cunningham-Rundles, Eric Gayle, Joanna Jen, Louise Bier, Mafalda Barbosa, Manisha Balwani, Mariya Shadrina, Rachel Evard, Saskia Shuman, Susan Shin, Andrea Gropman, Barbara N. Pusey Swerdzewski, Camilo Toro, Colleen E. Wahl, Donna Novacic, Ellen F. Macnamara, John J. Mulvihill, Maria T. Acosta, Precilla D'Souza, Valerie V. Maduro, Ben Afzali, Ben Solomon, Cynthia J. Tifft, David R. Adams, Elizabeth A. Burke, Francis Rossignol, Heidi Wood, Jiayu Fu, Joie Davis, Leoyklang Petcharet, Lynne A. Wolfe, Margaret Delgado, Marie Morimoto, Marla Sabaii, MayChristine V. Malicdan, Neil Hanchard, Orpa Jean-Marie, Wendy Introne, William A. Gahl, Yan Huang, Andrew Stergachis, Danny Miller, Elisabeth Rosenthal, Elizabeth Blue, Elsa Balton, Emily Shelkowitz, Eric Allenspach, Fuki M. Hisama, Gail P. Jarvik, Ghayda Mirzaa, Ian Glass, Kathleen A. Leppig, Katrina Dipple, Mark Wener, Martha Horike-Pyne, Michael Bamshad, Peter Byers, Runjun Kumar, Seth Perlman, Sirisak Chanprasert, Virginia Sybert, Wendy Raskind, Nitsuh K. Dargie, Chun-Hung Chan, Dr. Francisco Bustos velasq, Isum Ward, Jason Schend, Jennifer Morgan, Megan Bell, Miranda Leitheiser, Mohamad Saifeddine, Paul Berger, Rachel Li, Taylor Beagle, Alexander Miller, Beatriz Anguiano, Beth A. Martin, Brianna Tucker, Chloe M. Reuter, Devon Bonner, Elijah Kravets, Hector Rodrigo Mendez, Holly K. Tabor, Jacinda B. Sampson, Jason Hom, Jennefer N. Kohler, Jennifer Schymick, John E. Gorzynski, Jonathan A. Bernstein, Kevin S. Smith, Laura Keehan, Laurens Wiel, Matthew T. Wheeler, Meghan C. Halley, Mia Levanto, Page C. Goddard, Paul G. Fisher, Rachel A. Ungar, Raquel L. Alvarez, Sara Emami, Shruti Marwaha, Stephen B Montgomery, Suha Bachir, Tanner D Jensen, Taylor Maurer, Terra R. Coakley, Euan A. Ashley, Ali Al-Beshri, Anna Hurst, Brandon M Wilk, Bruce Korf, Elizabeth A Worthey, Kaitlin Callaway, Martin Rodriguez, Tammi Skelton, Tarun KK Mamidi, Andrew B. Crouse, Jordan Whitlock, Mariko Nakano-Okuno, Matthew Might, William E. Byrd, Albert R. La Spada, Changrui Xiao, Elizabeth C. Chao, Eric Vilain, Jose Abdenur, Kirsten Blanco, Maija-Rikka Steenari, Rebekah Barrick, Richard Chang, Sanaz Attaripour, Suzanne Sandmeyer, Tahseen Mozaffar, Alden Huang, Andres Vargas, Bianca E. Russell, Brent L. Fogel, Esteban C. Dell'Angelica, George Carvalho, Julian A. Martínez-Agosto, Layal F. Abi Farraj, Manish J. Butte, Martin

G. Martin, Naghmeh Dorrani, Neil H. Parker, Rosario I. Corona, Stanley F. Nelson, Yigit Karasozen, Aaron Quinlan, Alistair Ward, Ashley Andrews, Corrine K. Welt, Dave Viskochil, Erin E. Baldwin, John Carey, Justin Alvey, Laura Pace, Lorenzo Botto, Nicola Longo, Paolo Moretti, Rebecca Overbury, Russell Butterfield, Steven Boyden, Thomas J. Nicholas, Matt Velinder, Gabor Marth, Pinar Bayrak-Toydemir, Rong Mao, Monte Westerfield, Brian Corner, John A. Phillips III, Kimberly Ezell, Lynette Rives, Rizwan Hamid, Serena Neumann, Ashley McMinn, Joy D. Cogan, Thomas Cassini, Alex Paul, Dana Kiley, Daniel Wegner, Erin McRoy, Jennifer Wambach, Kathy Sisco, Patricia Dickson, F. Sessions Cole, Dustin Baldridge, Jimann Shin, Lilianna Solnica-Krezel, Stephen C. Pak, Timothy Schedl, Allen Bale, Carol Oladele, Caroline Hendry, Emily Wang, Hua Xu, Hui Zhang, Lauren Jeffries, María José Ortuño Romero, Mark Gerstein, Michele Spencer-Manzon, Monkol Lek, Nada Derar, Odelya Kaufman, Shrikant Mane, Teodoro Jerves Serrano, Vasilis Vasiliou, Winston Halstead, Yong-Hui Jiang
